# EnzML: multi-label prediction of enzyme classes using InterPro signatures

**DOI:** 10.1186/1471-2105-13-61

**Published:** 2012-04-25

**Authors:** Luna De Ferrari, Stuart Aitken, Jano van Hemert, Igor Goryanin

**Affiliations:** 1 Computational Systems Biology and Bioinformatics, School of Informatics, University of Edinburgh, Informatics Forum, 10 Crichton Street, UK; 2 Artificial Intelligence Applications Institute, Centre for Intelligent Systems and their Applications, School of Informatics, University of Edinburgh, Appleton Tower, 11 Crichton Street, UK; 3 Data Intensive Research, Centre for Intelligent Systems and their Applications, School of Informatics, University of Edinburgh, Informatics Forum, 10 Crichton Street, UK; 4 Biological Systems Unit, Okinawa Institute of Science and Technology, 1919-1 Tancha, Onna-son, Japan

## Abstract

**Background:**

Manual annotation of enzymatic functions cannot keep up with automatic genome sequencing. In this work we explore the capacity of InterPro sequence signatures to automatically predict enzymatic function.

**Results:**

We present EnzML, a multi-label classification method that can efficiently account also for proteins with multiple enzymatic functions: 50,000 in UniProt. EnzML was evaluated using a standard set of 300,747 proteins for which the manually curated Swiss-Prot and KEGG databases have agreeing Enzyme Commission (EC) annotations. EnzML achieved more than 98% subset accuracy (exact match of *all* correct Enzyme Commission classes of a protein) for the entire dataset and between 87 and 97% subset accuracy in reannotating eight entire proteomes: human, mouse, rat, mouse-ear cress, fruit fly, the *S. pombe* yeast, the *E. coli* bacterium and the *M. jannaschii* archaebacterium. To understand the role played by the dataset size, we compared the cross-evaluation results of smaller datasets, either constructed at random or from specific taxonomic domains such as archaea, bacteria, fungi, invertebrates, plants and vertebrates. The results were confirmed even when the redundancy in the dataset was reduced using UniRef100, UniRef90 or UniRef50 clusters.

**Conclusions:**

InterPro signatures are a compact and powerful attribute space for the prediction of enzymatic function. This representation makes multi-label machine learning feasible in reasonable time (30 minutes to train on 300,747 instances with 10,852 attributes and 2,201 class values) using the Mulan Binary Relevance Nearest Neighbours algorithm implementation (BR-kNN).

## Background

Assigning enzymatic function to the proteins in a genome is one of the first essential steps of metabolic reconstruction, important for biology, medicine, industrial production and environmental studies. Without precise annotation of the reactions a protein can perform, the subsequent pathway assembly and verification becomes problematic [1]. Metabolic flux studies that aim to understand diseased states or biomass production become almost impossible.

Unfortunately, at the current rate of genome sequencing and manual annotation, manual curation will never complete the functional annotation of all available proteomes [2]. Hence in this work we propose and evaluate a method to automatically predict the enzymatic functions of a protein. Previously, Tetko et al. [3] used component analysis to show that the highest contributor to the performance of various protein function prediction methods were InterPro signatures. InterPro is an extensive database of conserved sequence signatures and domains [4] that can be computed from sequence data alone and for any sequence using the publicly available InterProScan algorithm [4,5]. Through the use of InterPro signatures, we demonstrate that it is possible to predict Enzyme Commission (EC) numbers [6] with high accuracy, recall (sensitivity) and precision (specificity), using the information contained in the protein sequence *exclusively*.

Despite some known limitations, such as some inconsistencies between the rules set by the nomenclature committee and the actual class definitions [7], we use the NC-IUBMB Enzyme Commission (EC) nomenclature to define enzymatic reactions, as it is the current standard for enzyme function classification. The EC nomenclature uses a four digit code, such as EC 1.2.3.4, to represent an enzymatic class. The first three digits represent an increasingly detailed definition of reaction class, while the last digit represents the accepted substrates.

Our approach is widely applicable as it uses exclusively information contained in the protein sequence, in contrast with methods that also require existing or computationally inferred structural information [8]. Further, our method supports *multi-label classification*, that is, the direct association of *multiple* enzymatic functions to each protein. A single enzyme can perform different reactions, either due to the presence of multiple catalytic sites or by regulation of a single site, and can hence be associated with multiple EC numbers. Multi-label learning can take multiple EC numbers, and their hierarchical relation, into account more coherently and effectively than creating an individual classifier for each class. It can also leverage the information contained in proteins annotated with incomplete EC numbers (about 2% of UniProt and 9% of Swiss-Prot annotations), such as EC 1.-.-.-, EC 1.2.-.- or EC 1.2.3.-.

Sequence based methods for the prediction of EC numbers include EFICAz [9], ModEnzA [10] and PRIAM [11]. PRIAM uses a set of position-specific scoring matrices (profiles) specific for each EC number to predict the existence of a given EC function somewhere in a fully sequenced genome. EnzML, ModEnzA and EFICAz try to assign EC numbers to individual protein sequences or fragments. ModEnzA builds Hidden Markov model profiles of positive and negative sequences specific for each four digits EC numbers, partial or multiple EC numbers cannot be assigned.

EFICAz can assign multiple EC numbers of exactly three or four digits by weighting information from four sequence based predictions methods using functionally discriminating residues for enzyme families, pairwise sequence comparison, Pfam enzyme families and Prosite patterns (EFICAz2 [12] is enhanced using Support Vector Machine learning). EFICAz, ModEnzA and PRIAM are further discussed and quantitatively compared with EnzML in the Discussion section and Additional file  1: methods_comparison.pdf.

Multi-label learning has been successfully applied to predict FunCat protein functions in yeast [13], GO functions in yeast [14], CYGD functions in yeast [15], FunCat and GO functions in yeast and plants [16] and other species [17], but has not yet been extensively applied to the prediction of enzyme functionality. A multi-label support vector machines methodology was used in the past to predict EC numbers but only up to the second EC digit (e.g.. EC 1.2.-.-) and only on 8,291 enzymes [18]. Hierarchical classification was also applied to about 6,000 enzymes from KEGG, obtaining over 85% accuracy in predicting four digits EC numbers [19]. However, here we demonstrate that bigger datasets can cause dramatic improvement in performance. We make use of Mulan [20,21], an open-source software infrastructure for evaluation and prediction based on the Weka framework [22], to improve the potential for extension and reuse of this work. In addition to the effect of dataset size, we report on how predictions depend on species content and sequence redundancy. We also obtain very good computational performance over a real-life size set of 1,099,321 protein entries.

## Methods

### Data sources

The protein sequence and EC annotation data was taken from UniProt Knowledge Base [23] release 2010_12 (Nov-2010) consisting of Swiss-Prot release 2010_12 and TrEMBL release 2010_12, InterPro release 30.0 (Dec 2010), KEGG [24] release 57.0 (Jan 2011). The InterPro release used contains 21,591 signatures, 21,178 of which present in UniProt. The complete set of 5,222 EC numbers and their status (active, deleted or transferred) was downloaded from ExPASy ENZYME database (11-Jan-2011 release) [25]. All annotations using deleted EC numbers were removed from the data; transferred EC numbers were substituted with their newly assigned EC number(s). The data was further processed using Ondex [26,27] and MySQL. The data sources content of EC and InterPro annotation is summarised in Additional file  2: ec_interpro_stats.pdf.

**Figure 1:**
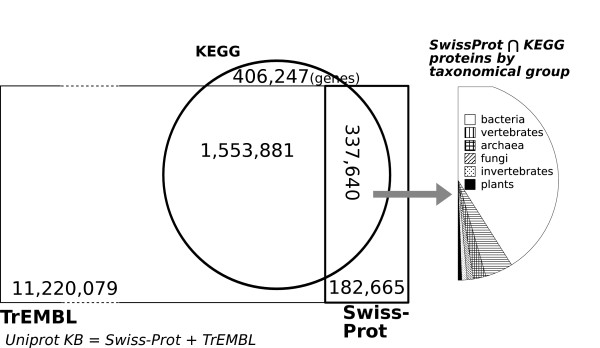
**The shared protein content of UniProt and KEGG.** The circle represents KEGG, the right rectangle represents Swiss-Prot (manually curated), while the left rectangle represents TrEMBL (mostly automatically curated). The two rectangles together compose the UniProt Knowledge Base. The intersection between Swiss-Prot and KEGG has been further expanded to show the distribution of taxonomic groups. For legibility, the areas in the pseudo Venn diagram are not exactly proportional to the number of proteins

The overlap between UniProt and KEGG is schematically represented in Figure 1, which shows that the manually curated section of the UniProt Knowledge Base (Swiss-Prot) only contains about half a million entries, versus the over twelve million entries awaiting manual annotation in TrEMBL. The taxonomic breakdown shows an overall dominance of bacterial annotation, in addition to a certain over representation of vertebrates and under representation of invertebrates, considering their estimated number of species in the tree of life. This distribution is not an artefact of the intersection, it is due to the underlying distribution of Swiss-Prot and KEGG data.

### Datasets

**Figure 2:**
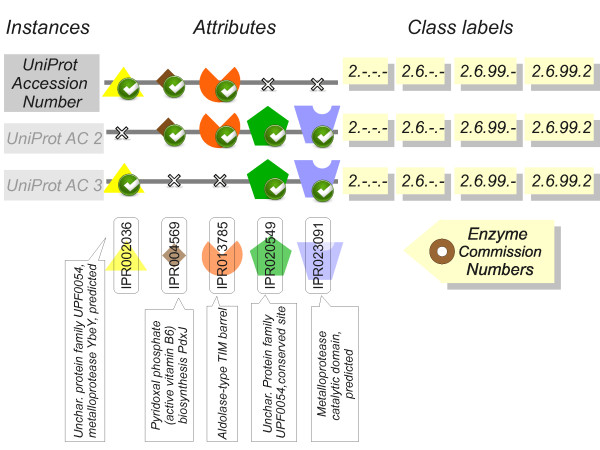
**Data schema: protein instances, InterPro attributes, EC classes.** In the data schema used each row represents one UniProt protein. An attribute value is the presence or absence of an InterPro signature, here shown as a geometrical shape. The class labels are one or more EC numbers, either accessible to the learning algorithm (for training) or invisible (for testing and predicting). The example shows the InterPro signatures associated with EC number 2.6.99.2 in UniProt (Pyridoxine 5-phosphate synthase, vitamin B6 pathway). These three combinations of five signatures compactly represent the 1,108 UniProt proteins having function 2.6.99.2

The EnzML data schema is shown in Figure 2, where each instance represents a protein identified by a UniProt Accession Number. Each protein can have zero or more class labels in the form of Enzyme Commission (EC) numbers. Each instance can also have zero or more attributes (features), each representing the presence or absence of one or more InterPro signatures (protein domains, catalytic sites, sequence repeats etc.).

In order to execute the different evaluations presented in the Results section, a number of datasets have been created. The main dataset is indicated from now on as *SwissProt* >*KEGG*. The join symbol () represents the fact that this set contains only annotation that is equal in the two databases. *SwissProt**KEGG* consists of all EC annotations *agreeing* in both Swiss-Prot and KEGG, an annotation being a couple in the form [UniProt Accession Number, EC number]. The set includes 300,747 proteins, 55% enzymes and 45% non enzymes (see below for a definition of non enzyme). The *SwissProt**KEGG* dataset has thus been submitted to *two manual curations*, in which none of the authors were involved. In the same way, the *TrEMBL**KEGG* dataset includes all annotations agreeing between UniProt TrEMBL and KEGG. The *TrEMBL**KEGG* dataset is very extensive and varied, but it has not been manually curated in TrEMBL. This dataset has been included in the analysis not for the purpose of method evaluation, but to review EnzML performance on a large dataset and to judge the internal consistency of *TrEMBL**KEGG* itself. The protein instances have surprisingly few features, having an average of 3.55 InterPro signatures (attribute values) and 3.97 EC numbers (class labels, including incomplete EC numbers) per protein.

The proportion of proteins with no EC annotations ranges from 45% of the *SwissProt**KEGG* dataset to 69% of the *TrEMBL**KEGG* dataset. These sets include proteins that have been extensively studied and do not carry enzymatic activity (especially in the *SwissProt**KEGG* dataset) as well as proteins not yet characterised as enzymes or belonging to still unknown enzymatic classes (more probable in the *TrEMBL**KEGG* dataset). Due to the difficulty of distinguishing between these cases, the non and not yet EC proteins are treated as one class. This allows EnzML to emit a cumulative no EC prediction as an alternative to the prediction of one or more EC numbers. A protein predicted as no EC could thus be either a non-enzyme or a not yet characterised enzyme or belonging to a not yet characterised enzyme class. For simplicity we refer to this class as non enzyme from now on. The EnzML method can accept instances with an empty set of attributes, which account for 0.3% of the *SwissProt**KEGG* dataset and 1.7% of the *TrEMBL**KEGG* dataset. These proteins are processed normally, but they are generally predicted as non enzymes due to the fact that most proteins without InterPro signatures also do not have EC annotations. The datasets used also include (and hence the method predicts) incomplete EC classes, such as EC 1.-.-.- , EC 1.2.-.- or EC 1.2.3.-.

The independence of the UniProt and KEGG curation cannot be determined by the annotations alone due to a lack of provenance meta-data. Curators in both institutions use a variety of primary (experimental data and literature) and secondary (other databases) sources to assign an EC annotation. However, out of the 1.8 million proteins annotated in both Uniprot and KEGG, 31% have a disagreeing annotation (20% for Swiss-Prot vs. KEGG and 33% for TrEMBL vs. KEGG), showing that the two knowledge bases curators have different scientific opinions in many cases.

In order to evaluate the impact of the dataset size and taxonomic content on EnzML performance, the *SwissProt*>*KEGG* dataset has been partitioned into taxonomic domains: archaea, bacteria and eukaria, further divided into fungi, invertebrates, plants and vertebrates. For each taxonomic domain we have investigated the individual proteome having most proteins in the *SwissProt**KEGG* set: *Methanocaldococcus jannaschii* for archaea, *Escherichia coli* (all strains) for bacteria, *Schizosaccharomyces pombe* for fungi, *Drosophila melanogaster* for invertebrates, *Arabidopsys thaliana* for plants, *Homo sapiens* for vertebrates. We also considered *Mus musculus* and *Rattus norvegicus* as second and third most represented species overall (the first is *Homo sapiens*).

To examine the performance on each EC main class, the Escherichia coli dataset was further divided into seven datasets each containing exclusively either the no enzyme annotation (*Ecoli_NoEC*) or EC annotations starting with a different main EC class (*Ecoli_EC1*, *Ecoli_EC2*, , *Ecoli_EC6*).

As an alternative to machine learning, EC labels could be directly assigned from InterPro domains: the InterPro2GO file associates individual InterPro signatures with GO terms, which in turn are mapped to EC numbers in the EC2GO file. To understand if EnzML is more accurate than this simple transitive assignment, a dataset was created containing all the *SwissProt**KEGG* entries annotated using the InterPro2GO and EC2GO lists provided by the UniProt FTP website (*InterPro2GO2EC*).

We have also created a separate set (named *SwissProt_2011_2012*) for proteins that were added to Swiss-Prot between Jan 2011 and March 2012 (16,938 proteins: 7,507 enzymes and 9,431 non-enzymes). The data was taken from BioMart Central UniProt. Of these proteins, an interesting subset consists of those 503 proteins (491 enzymes and 12 non-enzymes) which already existed in *TrEMBL**KEGG* as of Jan 2011 but acquired a new or different label (or lost their EC label) upon incorporation into Swiss-Prot (named *TrEMBL_2011_now_in_SwissProt_2012*).

The data format consists of a sparse Weka ARFF (Attribute-Relation File Format) file supplemented by a Mulan XML file containing the class labels hierarchy. Examples of ARFF and XML file formats are available in Additional file  3: arff_and_xml_file_examples.tar.gz. The *SwissProt**KEGG* and *TrEMBL**KEGG* data files used for evaluation are also available (Additional file  4: swiss-join-kegg_trembl-join-kegg_files.tar.gz) and so is the Java code used to format the data files and run the experiments (Additional file  5: enzml_java_code.tar.gz).

### Sequence redundancy

To analyse the performance of EnzML at different levels of sequence similarity we generated other datasets using UniRef clusters. UniRef100 is a database of clusters of UniProt proteins that are 100% identical in sequence (UniRef90 90% similar, UniRef50 50% similar in sequence). Each cluster has a representative (reference) protein sequence and a group of other sequences similar to it. To measure the effect of sequence redundancy on the method, the *SwissProt**KEGG* dataset was reduced to only its UniRef representative sequences (UniRef100 from *SwissProt**KEGG*, UniRef90 from  and UniRef50 from *SwissProt**KEGG* datasets) and cross-evaluated.

### EC numbers distribution

**Figure 3:**
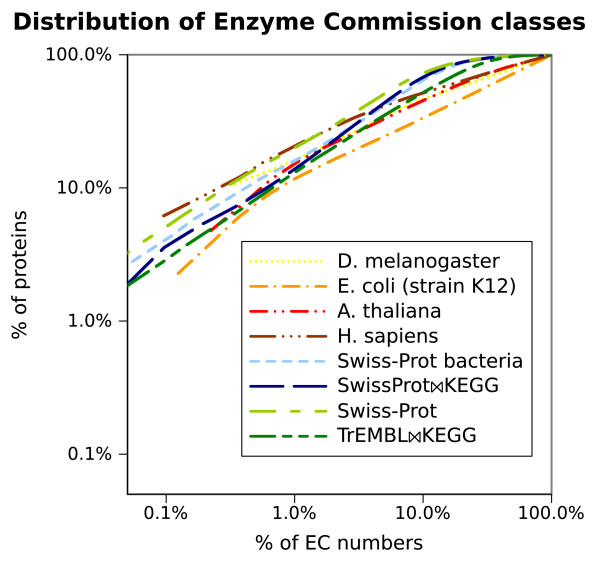
**Distribution of Enzyme Commission numbers among proteins.** To compare datasets of different sizes, the distribution is represented as cumulative percentage, starting with the most frequent EC number. The x and y axis are logarithmic. The datasets in the legend are in descending order of size. If each EC number were to annotate exactly the same proportion of proteins, the distribution would follow a diagonal from the bottom left to the top right corner of the plot

It is important to note that enzymatic classes are long-tail distributed in the main data sources, that is, some EC numbers are very frequent among proteins while most EC numbers only rarely occur. The distribution is very skewed (Figure 3), with roughly a 80-10 ratio: 80% of EC classes annotate only about 10% of UniProt enzymes, while the remaining 20% most common EC classes annotate 90% of UniProt enzymes (excluding the 45% of proteins with no EC annotation). The 2,825 most rare EC classes (80% of the total) only annotate 185,634 enzymes (about 10% of UniProt), and 731 EC classes have less than 5 protein examples in UniProt (277 EC classes only have one protein example in UniProt).

### Algorithm

The algorithm used throughout this work is BR-kNN [28]. BR-kNN is a multi-label adaptation of the traditional K-Nearest Neighbour using Binary Relevance. Binary Relevance transforms the original dataset into as many datasets as the existing labels, each example being labelled as label  true if the label existed in the original example and label  false otherwise (also called one-against-all or one-versus-rest approach). The Mulan version 1.2.0 implementation of BR-kNN [28] used in EnzML makes sure the (Euclidean) distance between neighbours is calculated only once, with considerable time savings on large datasets.

The best choice for the number of neighbours was k  1 (see Additional file  6: number_of_neighbours.pdf). BR-kNN is fast on the data used: less than 30 minutes per fold of a 10-fold cross-evaluation of 300,747 instances, on a dedicated machine with 2 GHz CPU and 4 GB RAM (14 hours to predict over a million instances). As baseline we used the Zero Rule algorithm, which assigns the majority class (non-enzyme) to every instance.

### Evaluation metrics

The evaluation metrics are either based on a single round of evaluation (train-test) or, for cross-evaluation, they are the average of a number of cross-evaluation rounds. After examining the standard deviations, we submitted datasets smaller than 40,000 proteins to two rounds of 10-fold cross evaluation, training on 9/10 of the data and testing on the remaining unseen 1/10 (one round of cross evaluation for bigger samples). We present the average value of *subset accuracy*, a strict measure of prediction success, as it requires the predicted set of class labels to be an *exact match* of the true set of labels [29]. For example, if a protein has these four EC class labels: [EC 1.-.-.-, EC 1.2.-.-, EC 1.2.3.- and EC 1.2.3.4], and it is assigned as prediction only the three first labels: [EC 1.-.-.-, EC 1.2.-.-, EC 1.2.3.-], this prediction would be considered as *completely* incorrect, because it misses the last label.

Where computable, we also report *micro* and *macro *metrics. In this context *micro* averaging (averaging over the entire confusion matrix) favours more frequent EC classes, while *macro* averaging gives equal relevance to both rare and frequent EC classes. Hence a protein will affect the macro-averaged metrics more if it belongs to a rare EC class. *Example-based* metrics consider how many correct EC predictions have been given to each individual protein example. The full mathematical form of all metrics is defined in [20] and [29]. The best achievable value of all these measures is 100% when all instances are correctly classified. Where averaged, the metrics are presented with plus and minus standard deviation marks.

### Statistical significance

To judge the difference between sets of results, the p-value at 5% confidence was used and calculated as follows. If the t-statistic is:

(1) $$t=\frac{\frac{X-M}{\mathrm{sd}}}{\sqrt{n}}$$

where *X* is the average (and *sd* the standard deviation) of the reference set of samples, *M* is the average of the other set of samples to be compared and *n* is the number of samples in both sets, the p-value becomes:

(2) p−value=tdist(abs(t),r,tails)

where *r* are the degrees of freedom (equal to n−1). Here we consider a two tailed hypothesis, so *tails* equals 2. *tdist* returns the probability density function for the t-distribution, calculating:

(3) $$\frac{\Gamma ((r+1)/2)}{\sqrt{r}\Gamma (r/2)}{\left(1+\frac{t^{2}}{r}\right)}^{\frac{-r+1}{2}}$$

where  is the Gamma function and *r* are the degrees of freedom. If the  is lower than 5%, the confidence that the samples come from different underlying distribution is higher than 95% and hence the two samples are declared significantly different.

## Results

### Whole, taxonomic and random datasets

**Figure 4:**
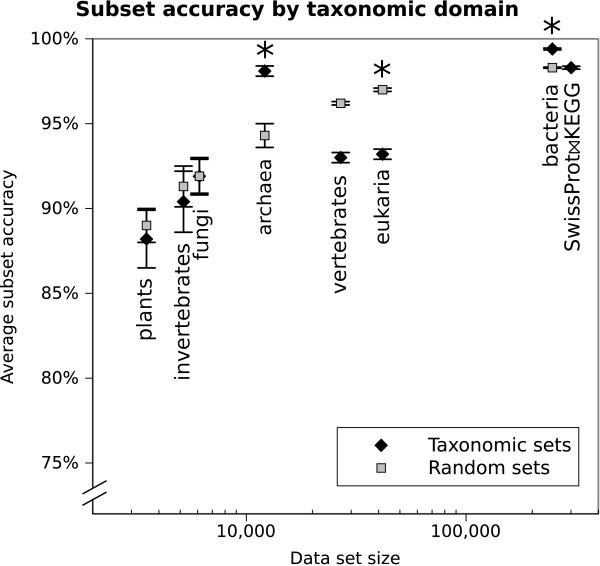
**Cross-evaluation results.** The plot compares the subset accuracy between taxonomic datasets and random sets of the same size. The rightmost point of the diagram is the whole *SwissProt**KEGG* dataset. The y axis (accuracy and recall) starts at 70%. An asterisk indicates significant difference in accuracy (with p-value at 5%) between the taxonomic and random datasets below. The full data is available in Additional file  7: all_cross_evaluation_results.csv

**Figure 5:**
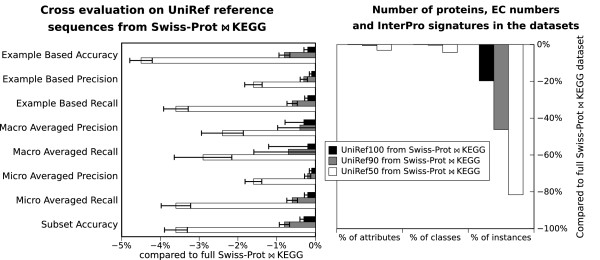
**Comparison with InterPro2GO2EC and testing on TrEMBL.** Left panel: The results of the internal cross-evaluation of the entire *SwissProt**KEGG* and Swiss-Prot datasets are compared with the direct transitive annotation using InterPro2GO and EC2GO lists. The results of training on the *SwissProt**KEGG* dataset and testing on the *TrEMBL**KEGG* dataset are also included. The x axis (accuracy, precision, recall) starts at 50%. Right panel: Comparison of the EC digits in the predicted and actual EC numbers for the *TrEMBL**KEGG* dataset. *All predictions* all the EC annotations emitted by training on *SwissProt**KEGG* and predicting the unlabelled *TrEMBL**KEGG* (true positives, true negatives, false positives, false negatives). Correct predictions  only the predictions corresponding to true, correct annotations existing in *TrEMBL**KEGG* (true positives and true negatives). Wrong predictions  false positives and false negatives. The data files used (*SwissProt**KEGG* and *TrEMBL**KEGG*) are available as Additional file  3. The full cross evaluation results are available in Additional file  7: all_cross_evaluation_results.csv

The first set of experiments assesses by cross evaluation the ability of EnzML to predict EC numbers using InterPro signatures. The cross evaluation results are summarised in Figure 4 (Additional metrics in Figure 5). The total dataset *SwissProt**KEGG* achieves 98% (0.1% standard deviation) subset accuracy (perfect match of all enzymatic classes of a protein). For comparison, the Zero Rule algorithm achieves 45%  0.2% subset accuracy.

To understand whether taxonomically related proteins were better at predicting proteins in their own taxa, the *SwissProt**KEGG* dataset has been subdivided into archaea, bacteria and eukarya (further divided into fungi, invertebrates, plants or vertebrates). The average classification accuracy after cross-evaluation of each taxonomic dataset was then compared with sets of the same size as each taxonomic set, but comprising proteins picked at random from *SwissProt**KEGG*.

The results in Figure 4 show that the predictions accuracy generally increases as the dataset size increases. Excluding far-related species does not seem to dramatically improve results: only the archaea and bacteria sets significantly outperform a random set of the same size, but they cover a reduced set of enzymatic functions compared to the full set. The plants, invertebrates, fungi and vertebrates sets are not significantly different from a random set of the same size, while the eukarya dataset accuracy is significantly different but lower.

### Sequence redundancy reduction

**Figure 6:**
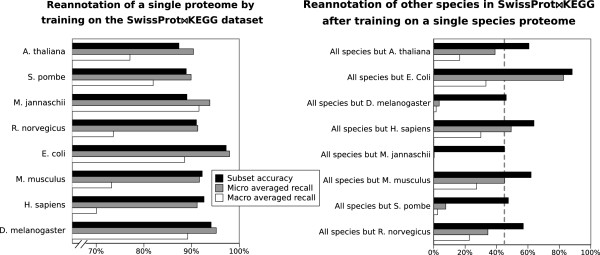
**Cross-evaluation on the UniRef reference sequences.** Left panel: the reference sequences are derived from *SwissProt**KEGG* using UniRef100, UniRef90 or UniRef50 clusters. Right panel: number of protein instances, InterPro attributes and EC classes when the *SwissProt**KEGG* dataset is reduced to its UniRef representative sequences. The values in both panels are shown as difference to the corresponding value for the entire *SwissProt**KEGG* dataset. The full data is available in Additional file  7: all_cross_evaluation_results.csv

To evaluate the impact of the sequence redundancy reduction on the method, a cross evaluation was executed on the three sets of proteins derived from *SwissProt**KEGG* by keeping only the UniRef reference entries (*SwissProt**KEGG* from UniRef100, *SwissProt**KEGG* from UniRef90 and *SwissProt**KEGG* from UniRef50). Hence the *SwissProt**KEGG* UniRef50 dataset contains only one representative sequence per each 50% similarity cluster. When the dataset is submitted to 10-fold cross-evaluation, the nine tenth of sequences that make up the training set are all less than 50% similar to the sequences in the test set (the remaining 10th). The results, shown in Figure 6, are robust and not particularly affected by the reduction to UniRef sequences, not even when clustering at 50% of sequence similarity, despite losing 80% of the sequences, as shown in the right panel of Figure 6. This is because, in spite of the dramatic sequence reduction and reduced overall sequence similarity, only 4% of the EC classes and 3% of the InterPro signatures are lost.

### Proteome reannotation

**Figure 7:**
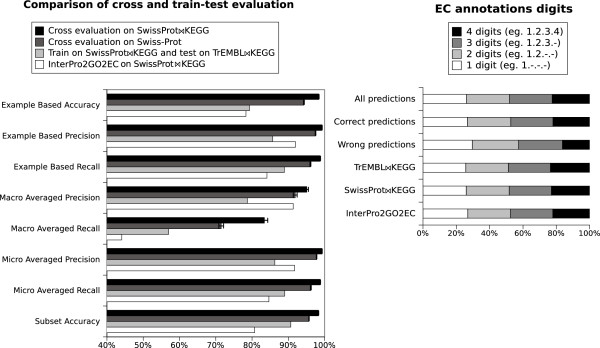
**Reannotation of proteomes.** Left panel: reannotation of individual species proteomes. The classifier is trained on the *SwissProt**KEGG* dataset (minus the species to be predicted) and then used to predict each species proteome. The x axis (accuracy and recall) starts at 65%. Right panel: reannotation of the entire *SwissProt**KEGG* dataset starting from a single species proteome. The classifier is trained on a single proteome and then used to predict all the other species. The dashed line at 45% represents the baseline of subset accuracy than would be obtained if all proteins were simply classified as non-enzyme. There are no standard deviation bars since no randomisation is involved: each value represents one experiment (one species excluded or all other species excluded)

The performance obtained by cross evaluating the entire *SwissProt**KEGG* dataset is representative of the success that can be expected on a metagenomic sample, especially one with a high bacterial content, as suggested by the high bacterial content in Figure 1. We hence executed another set of experiments to evaluate the performance of EnzML on annotating *individual* proteomes. Each experiment: 1. excluded the chosen species from the *SwissProt**KEGG* dataset, 2. trained on the remaining data, 3. re-annotated that species proteome (as if it were from a newly sequenced genome), and 4. compared the predictions with the existing annotations (sometimes referred to as jackknife evaluation). Figure 7 shows that EnzML can re-annotate an entire proteome with subset accuracy starting at 87% for *A. thaliana* and reaching 97% for *E. coli*.

To gauge the predictive power or a single species, the inverse was also attempted: to re-annotate the entire *SwissProt**KEGG* dataset based on a single proteome. This inverse exercise (Figure 7) shows that up to 88% of proteins, and more than a third of the EC classes, can be reannotated correctly in the *SwissProt**KEGG* dataset (minus *E. coli*) if the training occurs on possibly the most studied species in Molecular Biology, *E. coli*. This suggests a high level of evolutionary conservation of core metabolism across species.

### Comparison with InterPro2GO2EC and TrEMBL

EC labels could also be directly assigned from InterPro domains using the InterPro2GO and EC2GO lists. As shown in Figure 5, this method has much lower accuracy (80%) than EnzML (97%) on the same *SwissProt**KEGG* dataset. To assess computational performance, EnzML was also trained on *SwissProt**KEGG* (the right semicircle in Figure 1) and tested on the diverse and extensive, but not intensively manually curated, *TrEMBL**KEGG* dataset (the left semicircle in Figure 1). The loss of accuracy on the *TrEMBL**KEGG* dataset is not due to a limitation in EnzML, but more to the sheer variety and low internal consistency of *TrEMBL**KEGG*. The *SwissProt**KEGG* - the training set - only contains half of the InterPro domains existing in the *TrEMBL**KEGG* test set (see Additional file  2).

Figure 5 also shows the number of EC digits for the predictions and the correct EC number annotations. The higher the number of digits, the more specific the prediction, for example: EC 1.-.-.- only provides a generic enzymatic classification (oxidoreductases), while EC 1.2.3.4 defines the catalytic functionality down to the class of substrates (oxalate oxidase, with oxygen as acceptor). The proportion of predicted four digits EC numbers appears to be in line with their proportion in the true dataset.

As the predictions emitted by EnzML trained on *SwissProt**KEGG* for the *TrEMBL**KEGG* set are of interest for scientists working on non-model organisms, they are available as Additional file  8: TrEMBL_join_KEGG_true_and_predicted_EC_numbers.tar.gz.

A more detailed analysis of the prediction errors (using the E. coli dataset as example) is contained in Additional file  9: predictions.pdf. The additional file includes a table with the most common errors and the accuracy for each of the six main EC classes.

### Predicting recent Swiss-Prot entries

EnzML trained on *SwissProt**KEGG* (Jan 2011) can correctly predict most of the entries incorporated into Swiss-Prot in the following year (*SwissProt_2011_2012* set) and does so with 79% subset accuracy, 89% micro averaged precision and 64% macro averaged recall. EnzML performance is limited by the fact that 13% of the entries are annotated with new EC numbers that did not exist in the *SwissProt**KEGG* set of Jan 2011 and so cannot be predicted by the classifier. For comparison, a 10 fold cross-evaluation over the same *SwissProt_2011_2012* set achieves much better results (subset accuracy 92%  0.6%, micro averaged precision 96%  0.6%, macro averaged recall 79%  1.7%) because the probability of a class existing in the test set but not in the training set is low.

Also, EnzML trained on *SwissProt**KEGG* can correctly predict 69% of the new labels given to TrEMBL proteins upon their incorporation into Swiss-Prot (*TrEMBL_2011_now_in_SwissProt_2012* set). This suggests that many of the mistakes in the *TrEMBL**KEGG* predictions could actually become correct labels after manual curation. Here as well the performance is limited because 15% of the EC classes used in these new annotations did not exist in the *SwissProt**KEGG* set of Jan 2011 the classifier is trained on.

## Discussion

### Effects of EC distribution

The long-tail shape of the EC distribution is conserved even when the data is further categorised, often the case with long-tail distributions, and can be seen in the similarity of distributions for single species and full databases (Figure 3). This could be caused by evolutionary conservation of certain metabolic functions. Individual species, even compact bacterial genomes such as *E. coli*, have redundancy in certain enzymatic functions, and these functions seem to be common across species, leading to very frequent EC numbers such as Cytochrome-c oxidase (EC 1.9.3.1, mitochondrial respiration pathway) representing alone 12% of all UniProt enzymes.

The rare EC numbers do not impact on most evaluation measures as they affect a small number of proteins, but in Figure 4 we can note that the macro-averaged recall, a measure affected by the misprediction of rare classes is generally the lowest and more unpredictable metric for this method, as shown also by the wider standard deviation in Figures 5 and 6. Also, the macro-averaged recall of *SwissProt**KEGG* cross evaluation is lower than expected at 83%, despite only 20% of its EC numbers being very rare (having less than 3 proteins) versus 63% in invertebrates and 22% in bacteria. However, the measure improves (from 83% to 88%) if 20 fold cross evaluation is used instead of 10 fold, hence raising the probability of having in the training set more examples of rare and very rare EC classes (data not shown).

### Method applicability

The proposed method is applicable to any partial or complete protein sequence or metagenomic sample, since any genetic sequence can be scanned *in silico* for the presence of InterPro signatures using the InterProScan algorithm, also available as web service [4,5].

The overall success of EnzML is due to the fact that InterPro signatures provide a very compact representation of protein functionality. The 13.5 million proteins in UniProt are described by only 154,583 (unordered) sets of InterPro signatures (attributes). Many of these sets are subsets of other longer signature sets. InterPro subsets in UniProt have an average length of 2.77 signatures, while InterPro super-sets have an average length of 4.78 signatures. 58,697 super-sets completely describe the possible combinations of InterPro signatures found in all UniProt proteins. To give a comparison, 1,582 billion combinations of three unordered elements could be obtained from 21,178 InterPro signatures (8.4 E+15 combinations of four elements).

In relation to the method application and evaluation, it must be noted that the distribution of annotation in metabolic databases tends, by definition, to be more enriched in enzymes than in non-enzymes. Even highly-populated databases such as UniProt are biased, with more accurate annotation (and Swiss-Prot status) going to widely studied biological functions. Using only annotations that agree in two manually curated databases (such as Swiss-Prot and KEGG in this work) increases trust, but decreases the number of EC classes that can be predicted. Swiss-Prot contains 2,850 distinct EC classes, and KEGG contains 2,636 EC classes, but the set of annotations agreeing in both databases only contains 2,051 EC classes. Rare EC classes can easily be lost in case of disagreement among the data sources.

The accuracy of the predictions generally increases as the dataset size increases which, combined with the efficiency of the algorithm, is a good case for using a bigger training set whenever possible. Training the classifier on more data from non-manually curated databases, such as UniProt-TrEMBL, might reduce the bias and increase the number of predictable classes, but will also decrease trust. Alternative biocuration scenarios might call for a different balance between coverage and trust, to increase the probability of recognising rare Enzyme Commission classes in newly sequenced genomes.

Although the highest possible level of accuracy is clearly desirable, the high accuracy of EnzML, combined with the measure of confidence that the method emits for each prediction, enables the curators to focus their work. The majority of erroneous annotations have low confidence (results not shown), so curators could tackle the more error prone annotations first. However, active learning research has shown that simply correcting low-confidence annotations is rarely the best strategy, as the representativeness and informative content of each instance also has an impact. A strength of fast re-training systems such as EnzML is the potential to incrementally improve overall accuracy when incorrect annotations are spotted by curators. The authors are currently researching active learning strategies to improve enzyme annotation accuracy in a mixed human-machine learning curation workflow.

### Comparison with other EC prediction methods

PRIAM [11] was designed predict the overall metabolism for an organism, indicating whether particular enzyme functionalities were encoded in the genome, rather than assign functions to individual genes. A gene-oriented version of PRIAM was introduced in 2006 to address this task. In contrast, EnzML is designed to associate EC numbers to individual genes or gene fragments. EnzML improves on ModEnzA [10] by supporting the prediction of multiple EC numbers for a protein, and on EFICAz [9] by being able to assign multiple EC numbers of any number of digits. EFICAz2 [12] improves the precision of EFICAz on test sequences having less than 30% similarity to the training set, and has not been evaluated separately from EFICAz.

However, it is possible to compare EFICAz2 results at MTTSI  50% (maximal test to training sequence identity) in Figure 4-C and 4-D of [12] with those obtained by EnzML (Figure 6 of this article). In more detail, EFICAz2 reports a maximum recall of 47%  49% of standard deviation (for MTTSI  30%), 78%  33% (for MTTSI 30-40%) and 86%  34% (for MTTSI 40-50%). EFICAz2 precision reaches a maximum of 74%  44% of standard deviation (for MTTSI  30%), 82%  36% (for MTTSI 30-40%) and 91%  27% (for MTTSI 40-50%). In a similar range of protein similarity (MTTSI  50%) EnzML obtains generally more accurate results and within 0.7% of standard deviation, thanks also to its extensive dataset. In particular, EnzML results on *SwissProt**KEGG* UniRef50 are 80-95% recall (micro, macro, example based) and 93-98% precision (micro, macro, example based), all within less than  1% of standard deviation.

A comparison between EnzML on the four genomes used for evaluation in [10] (see Additional file  1: methods_comparison.pdf) shows that our method achieves greater sensitivity and specificity on a greater number of sequences, as our method uses more recent data. The data used for the comparison is available in Mulan ARFF format as Additional file  10: methods_comparison_arff_data.tar.gz and in comma-separated format as Additional file  11: methods_comparison_csv_data.tar.gz (including all the *SwissProt**KEGG* data).

## Conclusions

The EnzML method can be applied to any sequenced protein, without need for existing annotation or protein structures and it can provide quick, accurate and complete results on extensive datasets. EnzML leverages the evolutionary similarity of metabolic function yet without loosing performance when sequences redundancy is reduced. Thanks to the Mulan Binary Relevance Nearest Neighbours implementation (BR-kNN) this is possible in reasonable time even for millions of sequences, showing clear potential for meta-genomic analysis. Our approach demonstrates the potential of InterPro signatures in predicting enzymatic function and easing the backlog of manual curation of enzymatic function.

We plan to couple EnzML with pool-based active learning to further reduce the number of annotated instances needed, saving precious annotators time while further speeding up the method. The goal is to create a virtuous cycle between automatic and manual annotation, that is able to keep up with high-throughput sequencing. In the future, EnzML could also be extended to learning all protein functionalities, for example in the form of Gene Ontology terms.

## Competing interests

The authors declare that they have no competing interests.

## Authors contributions

LDF and SA designed the study, analysed the results and wrote the manuscript. LDF collected the data and wrote the EnzML code. JVH and IG helped conceive the study, participated in its design and coordination and helped to draft the manuscript. All authors read and approved the final manuscript.

## Supplementary Material

Addtional file 1 Comparison between EnzML and EFICAz, ModEnzA and PRIAM. File methods_comparison.pdf contains a comparison of the predictive performance of EFICAz, ModEnzA and EnzML over three bacterial genomes (*E. Coli*, *B. Aphidicola* and *M. Pneumoniae*) and one eukaryoti cgenome (*P. Falciparum*), and comparison of EnzML and PRIAM over two additional bacterial genomes (*Haemophilus influenzae* and *Mycoplasma genitalium*). The data used for the comparison is also available as Additional files  10 and 11. Click here for file

Addtional file 2 Table summary of EC and InterPro annotations in UniProt, KEGG and derived datasets. A summary of the EC and InterPro content of UniProt, KEGG and other datasets used in this work is presented in file ec_interpro_stats.pdf. Click here for file

Addtional file 3 Examples of sparse Weka ARFF and Mulan XML file formats. An example of sparse Weka ARFF and its corresponding Mulan XML file is available in the file arff_and_xml_file_examples.tar.gz. Click here for file

Addtional file 4The *SwissProt**KEGG* and *TrEMBL**KEGG* ARFF and XML files. The *SwissProt**KEGG* and *TrEMBL**KEGG* ARFF and XML files used for train-test (jackknife) evaluation in Figure 5 are provided in: swiss-join-kegg_trembl-join-kegg_files.tar.gz.Click here for file

Addtional file 5 The Java code to format the data files, evaluate and predict. The file enzml_java_code.tar.gz contains the Java code used to format database data to ARFF and XML formats, to execute cross and train-test (jackknife) evaluations and to record evaluation results to database. More information is included in the readme.txt file and the Javadoc files. The code can be used with a MySQL database. To use a different database software, other JDBC drivers might be required. Click here for file

Addtional file 6 Figure of the relation between accuracy and number of neighbours for the nearest neighbours algorithm. The Figure in file number_of_neighbours.pdf shows the degradation in accuracy when the number of neighbours is increased above 1. Click here for file

Addtional file 7 All cross evaluation results. The file all_cross_evaluation_results.csv contains, in comma separated format, all the cross evaluation results summarised in Figures 4, 5 and 6. Click here for file

Addtional file 8EC predictions emitted by EnzML for the *TrEMBL**KEGG* set. The compressed file TrEMBL_join_KEGG_true_and_predicted_EC_numbers.tar.gz contains, in comma separated format: (1) the file TrEMBLJoinKEGG_EC_predicted_by_EnzML.csv with the full set of EC predictions emitted by EnzML (trained on *SwissProt**KEGG*) for the *TrEMBL**KEGG* set (proteins not listed were predicted as non enzymes); (2) the file TrEMBL_KEGG_agreeing_EC_annotations.csv containing the (agreeing) annotations attributed to the *TrEMBL**KEGG* set by Uniprot-TrEMBL and KEGG (an empty EC number signifies the protein is not an enzyme).Click here for file

Addtional file 9Prediction errors analysis. The PDF file predictions.pdf contains a brief analysis of the most common prediction errors when training on *SwissProt**KEGG* and testing on *E. coli* (all strains). It also contains separate accuracy results for each main EC class.Click here for file

Addtional file 10 Methods comparison: data files in Mulan ARFF format. The compressed file methods_comparison_arff_data.tar.gz contains the Mulan ARFF and XML files used for jackknife evaluation on the full proteomes of *E. Coli*, *B. Aphidicola*, *M. Pneumoniae*, *P. Falciparum*, *Haemophilus influenzae* and *Mycoplasma genitalium*. Click here for file

Addtional file 11*SwissProt**KEGG* data in comma separated format. The compressed file swissprot_join_kegg_csv_data.tar.gz includes comma separated files containing: the list of all Uniprot accession numbers in the *SwissProt**KEGG* set and their (1) EC numbers (swissprot_kegg_proteins_ec.csv), (2) species (swissprot_kegg_species.csv), (3) InterPro signatures identifiers (swissprot_kegg_interpro.csv), (4) InterPro sets (swissprot_kegg_interproset.csv, signatures identifiers separated by a double dash). It also contains all the jackknife (train-test) evaluation results used to compare EnzML with other methods in Additional file  1 (as methods_comparison_all_evaluation_results.csv).Click here for file
