# Supplementary material for: EnzML: multi-label prediction of enzyme classes using InterPro signatures
Source: BMC Bioinformatics. 2012 Apr 25;13:61. doi: 10.1186/1471-2105-13-61 (PMC3483700; doi:10.1186/1471-2105-13-61)
Supplement: Addtional file 1 — Comparison between EnzML and EFICAz, ModEnzA and PRIAM. File methods_comparison.pdf contains a comparison of the predictive performance of EFICAz, ModEnzA and EnzML over three bacterial genomes (E. Coli, B. Aphidicola and M. Pneumoniae) and one eukaryoti cgenome (P. Falciparum), and comparison of EnzML and PRIAM over two additional bacterial genomes (Haemophilus influenzae and Mycoplasma genitalium). The data used for the comparison is also available as Additional files 10 and 11. [file 1471-2105-13-61-S1.pdf]

# Additional File 1

**Article title:** EnzML: Multi-label prediction of enzyme classes using InterPro signatures

**Authors:** Luna De Ferrari, Stuart Aitken, Jano van Hemert and Igor Goryanin.

## 1 Comparison between EFICAz, ModEnzA and EnzML

Table 1 and 2 compares the recall (sensitivity) and true negative rate (specificity) of various methods in predicting EC numbers and assigning EC numbers to sequences. The prediction performance published in [1] for three bacterial and one eukaryotic genomes are compared to the corresponding EnzML results.

EnzML shows higher recall (sensitivity) for all genomes presented (with the only exception of the EC numbers recall in E. coli where ModEnzA Tier I+II = 91.1%, EnzML = 88.5%). As an example, for *E. coli* sequences: EFICAz 86%, ModEnzA 89-92%, EnzML 97%. EnzML also exhibits a very high true negative rate (specificity). For *E. coli* sequences: EFICAz 81%, ModEnzA 85-87%, EnzML 99.9%, in addition to high overall precision (98%) and accuracy (97%).

## 2 Comparison between PRIAM and EnzML

Table 3 compares the performance of PRIAM and EnzML in recognising EC classes in a data set. EnzML improves on PRIAM results on both recall (sensitivity) and true negative rate (specificity), for all genomes but *E. coli*, where specificity is higher, but recall is the same as PRIAM and lower than the KEGG Orthology.

### Bibliography

*[1] Desai, D. K.; Nandi, S.; Srivastava, P. K. & Lynn, A. M. ModEnzA: Accurate Identification of Metabolic Enzymes Using Function Specific Profile HMMs with Optimised Discrimination Threshold and Modified Emission Probabilities. Adv Bioinformatics, Biological Oceanography Division, Leibniz Institute of Marine Sciences (IFM-GEOMAR), Düsternbrooker Weg 20, 24105 Kiel, Germany., 2011, 2011, 743782*

*[2] Claudel-Renard, C.; Chevalet, C.; Faraut, T. & Kahn, D. Enzyme-specific profiles for genome annotation: PRIAM. Nucleic Acids Res, 2003, 31, 6633-6639*

| ***Methods*** | ***EFICAz (from [1])*** | ***ModEnzA (Tier I) (from [1])*** | ***ModEnzA (Tier I+II) (from [1])*** | ***EnzML*** |
|---|---|---|---|---|
| ***Annotation Benchmark*** | KEGG (Nov 2010) | KEGG (Nov 2010) | KEGG (Nov 2010) | Swiss-Prot (Dec 2010) ⋈ KEGG (Jan 2011) |
| ***Training Data*** | Swiss-Prot, TrEMBL, KEGG (2004) | Swiss-Prot, ENZYME (Jan 2010) | Swiss-Prot, ENZYME (Jan 2010) | Swiss-Prot (Dec 2010) ⋈ KEGG (Jan 2011) |
| ***E. Coli*** | | | | |
| ***Sequences*** | *856 (1051)* | *892(1021)* | *919 (1082)* | *13,800* |
| Sensitivity | 86.11 | 89.73 | 92.45 | **97.8 (A)** |
| Specificity | 81.44 | 87.36 | 84.93 | **99.9 (B)** |
| Precision | | | | 98.1 (C) |
| Accuracy | | | | 97.3 (D) |
| ***EC numbers*** | *647 (728)* | *648 (697)* | *683 (775)* | *927* |
| Sensitivity | 86.26 | 86.4 | **91.06** | 88.5 (E) |
| Specificity | 88.87 | 92.96 | 88.12 | **99.9 (F)** |
| Precision | | | | 93.0 (G) |
| ***B. Aphidicola*** | | | | |
| ***Sequences*** | *258 (273)* | *262 (271)* | *263 (273)* | *1,514* |
| Sensitivity | 93.81 | 95.27 | 95.63 | **99.5 (A)** |
| Specificity | 94.5 | 96.67 | 96.33 | **99.9 (B)** |
| Precision | | | | 99.5 (C) |
| Accuracy | | | | 98.8 (D) |
| ***EC numbers*** | *227 (238)* | *220 (229)* | *220 (233)* | *368* |
| Sensitivity | 91.53 | 88.7 | 88.7 | **97.5 (E)** |
| Specificity | 95.37 | 96.06 | 94.42 | **99.9 (F)** |
| Precision | | | | 98.0 (G) |

Table 1: *Comparison between methods to predict EC numbers. Data for columns 1-4 was taken from Supplementary Table 3 in [1], original caption: "genome-wide enzyme identification for three bacterial genomes (E. Coli , B. Aphidicola and M. Pneumoniae) and one eukaryotic genome (P. Falciparum) by ModEnzA and EFICAz using KEGG annotations as benchmark. Numbers within parentheses indicate the total number of sequences or EC numbers identified by each method." The EnzML column contains data from leave-one-proteome-out experiments (jackknife). For example, for the E. Coli rows, all E. Coli proteins belonging to all strains were excluded from the* $Swiss-Prot \bowtie KEGG$ *training set and used as test set. The metrics presented are: (A) Example based recall (sensitivity by sequence), (B) Example based specificity (specificity by sequence), (C) Example based precision, (D) Subset accuracy, (E) Macro-averaged recall (sensitivity by EC class), (F) Macro-averaged specificity (specificity by EC class), (G) Macro-averaged precision. The highest value for each row is highlighted in bold.*

| ***Methods*** | ***EFICAz (from [1])*** | ***ModEnzA (Tier I) (from [1])*** | ***ModEnzA (Tier I+II) (from [1])*** | ***EnzML*** |
|---|---|---|---|---|
| ***Annotation Benchmark*** | KEGG (Nov 2010) | KEGG (Nov 2010) | KEGG (Nov 2010) | Swiss-Prot (Dec 2010) ⋈ KEGG (Jan 2011) |
| ***Training Data*** | Swiss-Prot, TrEMBL, KEGG (2004) | Swiss-Prot, ENZYME (Jan 2010) | Swiss-Prot, ENZYME (Jan 2010) | Swiss-Prot (Dec 2010) ⋈ KEGG (Jan 2011) |
| ***M. Pneumoniae*** | | | | |
| ***Sequences*** | *112 (149)* | *114 (139)* | *114 (139)* | *297* |
| Sensitivity | 84.84 | 86.36 | 86.36 | **97.7 (A)** |
| Specificity | 75.16 | 82.01 | 82.01 | **99.9 (B)** |
| Precision | | | | 94.3 (C) |
| Accuracy | | | | 95.2 (D) |
| ***EC numbers*** | *91 (122)* | *102 (122)* | *102 (122)* | *191* |
| Sensitivity | 79.82 | 89.47 | 89.47 | **95.6 (E)** |
| Specificity | 74.59 | 83.6 | 83.6 | **99.9 (F)** |
| Precision | | | | 92.8 (G) |
| ***P. Falciparum*** | | | | |
| ***Sequences*** | *296 (480)* | *321 (415)* | *327 (431)* | *1,975* |
| Sensitivity | 54.91 | 59.55 | 60.66 | **97.9 (A)** |
| Specificity | 61.66 | 77.34 | 75.87 | **99.9 (B)** |
| Precision | | | | 99.9 (C) |
| Accuracy | | | | 96.0 (D) |
| ***EC numbers*** | *186 (247)* | *207 (234)* | *210 (242)* | *368* |
| Sensitivity | 62.2 | 69.23 | 70.23 | **96.7 (E)** |
| Specificity | 75.3 | 88.46 | 86.77 | **99.9 (F)** |
| Precision | | | | 99.9 (G) |

Table 2: *Same as Table 1. The highest value for each row is highlighted in bold.*

| | **KEGG Orthology (from [2])** | | **PRIAM jackknife (from [2])** | | ***EnzML jackknife*** | | |
|---|---|---|---|---|---|---|---|
| *Genome* | *Specificity* | *Sensitivity* | *Specificity* | *Sensitivity* | *Macro-Averaged Specificity* | *Macro-Averaged Recall (Sensitivity)* | *Macro-Averaged Precision* |
| *B. aphidicola* | 87% | 80% | 86% | 91% | 99% | **98%** | 98% |
| *E. coli* | 89% | **91%** | 92% | 88% | 99% | 88% | 93% |
| *H. influenzae* | 88% | 93% | 84% | 91% | 99% | **95%** | 97% |
| *M. genitalium* | 93% | 95% | 86% | 87% | 99% | **96%** | 94% |
| *M. pneumoniae* | 91% | 95% | 85% | 87% | 99% | **96%** | 93% |

Table 3: *Comparison between PRIAM, KEGG Orthology and EnzML. The data for columns 1-5 (PRIAM jackknife and KEGG Orthology) are taken from [2], original caption: "Specificity and sensitivity of PRIAM-based enzyme detection in five complete genomes, using SWISS-PROT annotation as a standard. The RPS-BLAST E-value was set at 10–30. Jackknife analysis was performed with PRIAM profiles in which sequences from the corresponding genome were omitted. Specificity and sensitivity of KEGG Orthology assignments (retrieved from http://www.genome.ad.jp/kegg/kegg2.html; 10,25) were calculated similarly against SWISS-PROT for comparison." Columns 6-7 contain EnzML results from leave-one-proteome-out experiments (jackknife) as detailed in Table 1 with the addition of Haemophilus influenzae and Mycoplasma genitalium data. The highest specificity value for the row is underlined. The highest sensitivity value is in bold.*
