# Supplementary material for: EnzML: multi-label prediction of enzyme classes using InterPro signatures
Source: BMC Bioinformatics. 2012 Apr 25;13:61. doi: 10.1186/1471-2105-13-61 (PMC3483700; doi:10.1186/1471-2105-13-61)
Supplement: Addtional file 2 — Table summary of EC and InterPro annotations in UniProt, KEGG and derived datasets. A summary of the EC and InterPro content of UniProt, KEGG and other datasets used in this work is presented in file ec_interpro_stats.pdf. [file 1471-2105-13-61-S2.pdf]

Statistics of EC and InterPro annotations in UniProt, KEGG and derived datasets

	Proteins (Uniprot Accession Numbers)	***EC classes (1)***	***% of the EC numbers existing in Expasy***	***% of proteins having an EC annotation (enzymes) (2)***	***% of non enzymes***	***InterPro signatures***	***% of proteins having an InterPro signature***
**Uniprot (Swiss-Prot + TrEMBL)**	13,294,255	3,549	75%	13%	87%	21,178	79.4%
**Swiss-Prot**	520,305	2,957	63%	46%	54%	17,907	95.0%
**TrEMBL**	12,773,950	3,145	67%	12%	88%	21,079	78.8%
**KEGG (3)**	1,891,521	2,697	57%	51%	49%	13,574	98.8%
**Swiss-Prot⋈KEGG**	300,747	2,064	44%	55%	45%	10,852	99.7%
**TrEMBL⋈KEGG**	1099337	2,088	44%	31%	69%	12,569	98.3%

Notes:

(1) the number of distinct EC classes including sub-classes. The Expasy ENZYME database lists 4409 EC classes, 4717 if including subclasses.

(2) at least one complete or incomplete EC number.

(3) for KEGG genes that could be converted to Uniprot AC (x out of y genes could not be converted).

(4) Proteins having the same EC number annotation in both KEGG and Swiss-Prot.
