# Supplementary material for: EnzML: multi-label prediction of enzyme classes using InterPro signatures
Source: BMC Bioinformatics. 2012 Apr 25;13:61. doi: 10.1186/1471-2105-13-61 (PMC3483700; doi:10.1186/1471-2105-13-61)
Supplement: Addtional file 5 — The Java code to format the data files, evaluate and predict. The file enzml_java_code.tar.gz contains the Java code used to format database data to ARFF and XML formats, to execute cross and train-test (jackknife) evaluations and to record evaluation results to database. More information is included in the readme.txt file and the Javadoc files. The code can be used with a MySQL database. To use a different database software, other JDBC drivers might be required. [file 1471-2105-13-61-S5.gz › java_code/enzml2011/doc/overview-frame.html]

Overview List


|  |
| --- |

|  |
| --- |
| All Classes Packages   test   test.dataharness   test.mulan   test.mulan.attributesfilter   test.mulan.learn   test.mulan.learn.database   test.mulan.learn.traintest   test.mulan.predict   test.weka   uk.ac.ed.inf.enzml   uk.ac.ed.inf.enzml.mulan   uk.ac.ed.inf.enzml.mulan.attributesfilter   uk.ac.ed.inf.enzml.mulan.database   uk.ac.ed.inf.enzml.mulan.learn   uk.ac.ed.inf.enzml.mulan.learn.traintest   uk.ac.ed.inf.enzml.mulan.predict   uk.ac.ed.inf.enzml.weka |
