# Supplementary material for: EnzML: multi-label prediction of enzyme classes using InterPro signatures
Source: BMC Bioinformatics. 2012 Apr 25;13:61. doi: 10.1186/1471-2105-13-61 (PMC3483700; doi:10.1186/1471-2105-13-61)
Supplement: Addtional file 5 — The Java code to format the data files, evaluate and predict. The file enzml_java_code.tar.gz contains the Java code used to format database data to ARFF and XML formats, to execute cross and train-test (jackknife) evaluations and to record evaluation results to database. More information is included in the readme.txt file and the Javadoc files. The code can be used with a MySQL database. To use a different database software, other JDBC drivers might be required. [file 1471-2105-13-61-S5.gz › java_code/enzml2011/doc/allclasses-frame.html]

All Classes


**All Classes**
  

|  |
| --- |
| AllArffTests   AllDataTests   AllMulanArffTests   AllMulanLearningTests   AllMulanPredictionTests   AllMulanTests   AllPreliminaryTests   AllTests   AllTrainTestsTests   AllUtilsTests   Arff   ArffGeneratorForFilterTests   ArffProperties   ArffPropsFilesTest   ArffPropsOneTest   ArffPropsQueriesOneTest   ArffPropsQueriesTest   ArffPropsQueriesTwoTest   ArffPropsTable   ArffPropsTableManager   ArffPropsTableManagerTest   ArffPropsTableReader   ArffPropsTableReaderTest   ArffPropsTableTest   ArffPropsTwoTest   ArffTest   AttributeFactory   AttributeFactoryTest   AttributesFilter   AttributesFilteredArff   AttributesFilteredArffTest   AttributesFilteredAttributeFactory   AttributesFilteredDataSetGenerator   AttributesFilteredDataSetGeneratorTest   AttributesFilteredDataSetManager   AttributesFilterTest   AttributeUtils   CreateDataTable   CrossEvaluatorTest   DatabaseTest   DataOne   DataSetChecker   DataSetCheckerTest   DataSetDbLoader   DataSetDbLoaderTest   DataSetGenerator   DataSetGeneratorTest   DataSetManager   DataSetManagerTest   DataSetWriter   DataSetWriterTest   DataTableOneTest   DataTableThreeTest   DataTableTwoTest   DataTwo   EvaluationMetricsTest   EvaluationParameters   EvaluationParametersTest   EvaluatorNoAUC   ExperimenterTest   ExperimentTable   ExperimentTableTest   IdentifiedSparseInstance   InstancesFiller   InstancesFillerTest   InstanceUtils   LearnerTest   MulanArff   MulanArffProperties   MulanArffRecord   MulanArffRecordTest   MulanArffTest   MulanAttributeFactory   MulanCrossEvaluator   MulanCrossExperimenter   MulanDataSetDbLoader   MulanDataSetDbLoaderTest   MulanDataSetGenerator   MulanDataSetGeneratorTest   MulanDataSetManager   MulanDataSetManagerTest   MulanDataSetWriterTest   MulanDbCreator   MulanDbCreatorTest   MulanDbManager   MulanDbManagerTest   MulanDbReader   MulanDbReaderTest   MulanDbWriter   MulanDbWriterTest   MulanEmitPredictions   MulanInstancesFiller   MulanInstancesFillerTest   MulanLearner   MulanLearners   MulanPredict   MulanPredictTest   MulanPredictWithTrainedModel   MulanSerializer   MulanTrain   ProjectParameters   ResultCheckerTest   ResultsFormatter   ResultsFormatterTest   ResultsSaver   ResultsSaverTest   SerializerTest   TestProjectParameters   TestProjectParametersTest   TrainTestEvaluator   TrainTestEvaluatorSerialised   TrainTestEvaluatorTest   TrainTestExperimenter   TrainTestExperimenterSerialized   TrainTestExperimenterTest   TrainTestFullRun   WekaTest |
