# Supplementary material for: EnzML: multi-label prediction of enzyme classes using InterPro signatures
Source: BMC Bioinformatics. 2012 Apr 25;13:61. doi: 10.1186/1471-2105-13-61 (PMC3483700; doi:10.1186/1471-2105-13-61)
Supplement: Addtional file 5 — The Java code to format the data files, evaluate and predict. The file enzml_java_code.tar.gz contains the Java code used to format database data to ARFF and XML formats, to execute cross and train-test (jackknife) evaluations and to record evaluation results to database. More information is included in the readme.txt file and the Javadoc files. The code can be used with a MySQL database. To use a different database software, other JDBC drivers might be required. [file 1471-2105-13-61-S5.gz › java_code/enzml2011/doc/constant-values.html]

Constant Field Values


---


|  |  |  |  |  |  |  |  |  |  |  |
| --- | --- | --- | --- | --- | --- | --- | --- | --- | --- | --- |
| |  |  |  |  |  |  |  |  | | --- | --- | --- | --- | --- | --- | --- | --- | | **Overview** | Package | Class | Use | **Tree** | **Deprecated** | **Index** | **Help** | | |  |
| PREV   NEXT | **FRAMES**    **NO FRAMES**     **All Classes** |


---


# Constant Field Values


---

**Contents**

- test.dataharness.\*- test.mulan.\*- uk.ac.\*

| test.dataharness.\* |
| --- |

| test.dataharness.DataOne | | |
| --- | --- | --- |
| `public static final java.lang.String` | `arffEmptyInstance` | `"{}"` |
| `public static final java.lang.String` | `arffInstance1` | `"{0 y,1 y,5 class1}"` |
| `public static final java.lang.String` | `arffInstance1b` | `"{0 y,1 y,5 class2}"` |
| `public static final java.lang.String` | `arffInstance2` | `"{2 y,5 class3}"` |
| `public static final java.lang.String` | `arffInstance4` | `"{3 y,5 class2}"` |
| `public static final java.lang.String` | `arffInstanceAttVoid` | `"{5 class4}"` |
| `public static final java.lang.String` | `arffInstanceClassVoid` | `"{4 y}"` |
| `public static final java.lang.String` | `ATT1` | `"att1"` |
| `public static final java.lang.String` | `ATT1_TO_STRING` | `"@attribute att1 {n,y}"` |
| `public static final java.lang.String` | `ATT2` | `"att2"` |
| `public static final java.lang.String` | `ATT2_TO_STRING` | `"@attribute att2 {n,y}"` |
| `public static final java.lang.String` | `ATT3` | `"att3"` |
| `public static final java.lang.String` | `ATT3_TO_STRING` | `"@attribute att3 {n,y}"` |
| `public static final java.lang.String` | `ATT4` | `"att4"` |
| `public static final java.lang.String` | `ATT4_TO_STRING` | `"@attribute att4 {n,y}"` |
| `public static final java.lang.String` | `ATT5` | `"att5"` |
| `public static final java.lang.String` | `ATT5_TO_STRING` | `"@attribute att5 {n,y}"` |
| `public static final java.lang.String` | `CLASS_TO_STRING` | `"@attribute class_name {dummy,class1,class2,class3,class4}"` |
| `public static final java.lang.String` | `CLASS1` | `"class1"` |
| `public static final java.lang.String` | `CLASS2` | `"class2"` |
| `public static final java.lang.String` | `CLASS3` | `"class3"` |
| `public static final java.lang.String` | `CLASS4` | `"class4"` |
| `public static final java.lang.String` | `DATASET_NAME` | `"test_data_1"` |
| `public static final java.lang.String` | `INST1` | `"inst1"` |
| `public static final java.lang.String` | `INST2` | `"inst2"` |
| `public static final java.lang.String` | `INST3` | `"inst3"` |
| `public static final java.lang.String` | `INST4` | `"inst4"` |
| `public static final java.lang.String` | `INSTATTVOID` | `"inst_att_void"` |
| `public static final java.lang.String` | `INSTCLASSATTVOID` | `"inst_class_att_void"` |
| `public static final java.lang.String` | `INSTCLASSVOID` | `"inst_class_void"` |

| test.dataharness.DataTwo | | |
| --- | --- | --- |
| `public static final java.lang.String` | `arffEmptyInstance` | `"{}"` |
| `public static final java.lang.String` | `arffInstance1` | `"{0 y,1 y,5 class1}"` |
| `public static final java.lang.String` | `arffInstance1b` | `"{0 y,1 y,5 class2}"` |
| `public static final java.lang.String` | `arffInstance2` | `"{2 y,5 class3}"` |
| `public static final java.lang.String` | `arffInstance4` | `"{3 y,5 class2}"` |
| `public static final java.lang.String` | `arffInstanceAttVoid` | `"{5 class4}"` |
| `public static final java.lang.String` | `arffInstanceClassVoid` | `"{4 y}"` |
| `public static final java.lang.String` | `DATASET_NAME` | `"test_ec_data"` |
| `public static final java.lang.String` | `INST1` | `"inst1"` |
| `public static final java.lang.String` | `INST2` | `"inst2"` |
| `public static final java.lang.String` | `INST3` | `"inst3"` |
| `public static final java.lang.String` | `INST4` | `"inst4"` |

| test.dataharness.TestProjectParameters | | |
| --- | --- | --- |
| `public static final java.lang.String` | `FILTER_OLD_TEST_SET` | `"src/test/data/properties_files/arff_props/testset_arff_for_filtering.props"` |
| `public static final java.lang.String` | `FILTER_TRAIN_SET` | `"src/test/data/properties_files/arff_props/trainset_arff_for_filtering.props"` |
| `public static final java.lang.String` | `INSTANCE_1_2` | `"src/test/data/properties_files/arff_props/instance_1_2.props"` |
| `public static final java.lang.String` | `INSTANCE_1_2_EMPTY` | `"src/test/data/properties_files/arff_props/instance_1_2_empty.props"` |
| `public static final java.lang.String` | `INSTANCE_3` | `"src/test/data/properties_files/arff_props/instance_3.props"` |
| `public static final java.lang.String` | `TEST_ARFF_DB_PROPS` | `"src/test/data/properties_files/arff_props/arff_table_db_connection.props"` |
| `public static final java.lang.String` | `TEST_ARFF_FILES_PATH` | `"src/test/data/arff/"` |
| `public static final java.lang.String` | `TEST_ARFF_PROPS_1` | `"src/test/data/properties_files/arff_props/test_arff_1.props"` |
| `public static final java.lang.String` | `TEST_ARFF_PROPS_2` | `"src/test/data/properties_files/arff_props/test_arff_2.props"` |
| `public static final java.lang.String` | `TEST_ARFF_PROPS_NO_XML` | `"src/test/data/properties_files/arff_props/test_arff_no_xml.props"` |
| `public static final java.lang.String` | `TEST_ARFF_PROPS_PATH` | `"src/test/data/properties_files/arff_props/"` |
| `public static final java.lang.String` | `TEST_BASE_DATA_PATH` | `"src/test/data/"` |
| `public static final java.lang.String` | `TEST_BASE_PATH` | `"src/test/"` |
| `public static final java.lang.String` | `TEST_DATA_DIR` | `"data/"` |
| `public static final java.lang.String` | `TEST_DB_CONN_PROPERTIES` | `"src/test/test_database_connection.props"` |
| `public static final java.lang.String` | `TEST_MACHINE_LEARNING_PROPS` | `"src/test/data/properties_files/machine_learning_props/mulan_test_results.props"` |
| `public static final java.lang.String` | `TEST_ML_PROPS_PATH` | `"src/test/data/properties_files/machine_learning_props/"` |
| `public static final java.lang.String` | `TEST_MODEL_FOLDER` | `"src/test/data/model/"` |
| `public static final java.lang.String` | `TEST_PREDICTIONS_FOLDER` | `"src/test/data/predictions/"` |
| `public static final java.lang.String` | `TEST_PROPS_PATH` | `"src/test/data/properties_files/"` |
| `public static final java.lang.String` | `TEST_RESULTS_FOLDER` | `"src/test/data/results/"` |

| test.mulan.\* |
| --- |

| test.mulan.MulanArffTest | | |
| --- | --- | --- |
| `public static final java.lang.String` | `arff_EC_Instance1` | `"{0 1,1 1,2 1,3 1,8 1}"` |
| `public static final java.lang.String` | `arffInstance1` | `"{0 1,1 1,5 1,6 1}"` |
| `public static final java.lang.String` | `arffInstance2` | `"{2 1,7 1}"` |
| `public static final java.lang.String` | `arffInstance4` | `"{3 1,6 1}"` |
| `public static final java.lang.String` | `arffInstanceAttributeVoid` | `"{8 1}"` |
| `public static final java.lang.String` | `arffInstanceClassAttributeVoid` | `"{}"` |
| `public static final java.lang.String` | `arffInstanceClassVoid` | `"{4 1}"` |
| `public static final java.lang.String` | `ATT_EC_1_TO_STRING` | `"@attribute 1.-.-.- {0,1}"` |
| `public static final java.lang.String` | `ATT_EC_11_TO_STRING` | `"@attribute 1.1.-.- {0,1}"` |
| `public static final java.lang.String` | `ATT_EC_111_TO_STRING` | `"@attribute 1.1.1.- {0,1}"` |
| `public static final java.lang.String` | `ATT_EC_1111_TO_STRING` | `"@attribute 1.1.1.1 {0,1}"` |
| `public static final java.lang.String` | `ATT_EC_2_TO_STRING` | `"@attribute 2.-.-.- {0,1}"` |
| `public static final java.lang.String` | `ATT_EC_22_TO_STRING` | `"@attribute 2.2.-.- {0,1}"` |
| `public static final java.lang.String` | `ATT_EC_222_TO_STRING` | `"@attribute 2.2.2.- {0,1}"` |
| `public static final java.lang.String` | `ATT_EC_2222_TO_STRING` | `"@attribute 2.2.2.2 {0,1}"` |
| `public static final java.lang.String` | `ATT1_TO_STRING` | `"@attribute att1 {0,1}"` |
| `public static final java.lang.String` | `ATT2_TO_STRING` | `"@attribute att2 {0,1}"` |
| `public static final java.lang.String` | `ATT3_TO_STRING` | `"@attribute att3 {0,1}"` |
| `public static final java.lang.String` | `ATT4_TO_STRING` | `"@attribute att4 {0,1}"` |
| `public static final java.lang.String` | `ATT5_TO_STRING` | `"@attribute att5 {0,1}"` |

| test.mulan.attributesfilter.AttributesFilteredArffTest | | |
| --- | --- | --- |
| `public static final int` | `TEST_ROLE` | `1` |
| `public static final int` | `TRAIN_ROLE` | `0` |

| uk.ac.\* |
| --- |

| uk.ac.ed.inf.enzml.ProjectParameters | | |
| --- | --- | --- |
| `public static final java.lang.String` | `ARFF_PROPS_ARCHAEA` | `"data/properties_files/arff_props/archaea.props"` |
| `public static final java.lang.String` | `ARFF_PROPS_BACTERIA` | `"data/properties_files/arff_props/bacteria.props"` |
| `public static final java.lang.String` | `ARFF_PROPS_EUKARIA` | `"data/properties_files/arff_props/eukaria.props"` |
| `public static final java.lang.String` | `ARFF_PROPS_FUNGI` | `"data/properties_files/arff_props/fungi.props"` |
| `public static final java.lang.String` | `ARFF_PROPS_HUMAN` | `"data/properties_files/arff_props/humans.props"` |
| `public static final java.lang.String` | `ARFF_PROPS_INVERTEBRATES` | `"data/properties_files/arff_props/invertebrates.props"` |
| `public static final java.lang.String` | `ARFF_PROPS_NOT_HUMAN` | `"data/properties_files/arff_props/not_human.props"` |
| `public static final java.lang.String` | `ARFF_PROPS_PATH` | `"data/properties_files/arff_props/"` |
| `public static final java.lang.String` | `ARFF_PROPS_PLANTS` | `"data/properties_files/arff_props/plants.props"` |
| `public static final java.lang.String` | `ARFF_PROPS_RANDOM_100` | `"data/properties_files/arff_props/swiss_kegg_random_100.props"` |
| `public static final java.lang.String` | `ARFF_PROPS_RANDOM_ARCHAEA` | `"data/properties_files/arff_props/swiss_kegg_random_archaea.props"` |
| `public static final java.lang.String` | `ARFF_PROPS_RANDOM_BACTERIA` | `"data/properties_files/arff_props/swiss_kegg_random_bacteria.props"` |
| `public static final java.lang.String` | `ARFF_PROPS_RANDOM_EUKARIA` | `"data/properties_files/arff_props/swiss_kegg_random_eukaria.props"` |
| `public static final java.lang.String` | `ARFF_PROPS_RANDOM_FUNGI` | `"data/properties_files/arff_props/swiss_kegg_random_fungi.props"` |
| `public static final java.lang.String` | `ARFF_PROPS_RANDOM_INVERTEBRATES` | `"data/properties_files/arff_props/swiss_kegg_random_invertebrates.props"` |
| `public static final java.lang.String` | `ARFF_PROPS_RANDOM_PLANTS` | `"data/properties_files/arff_props/swiss_kegg_random_plants.props"` |
| `public static final java.lang.String` | `ARFF_PROPS_RANDOM_VERTEBRATES` | `"data/properties_files/arff_props/swiss_kegg_random_vertebrates.props"` |
| `public static final java.lang.String` | `ARFF_PROPS_SWISSKEGG` | `"data/properties_files/arff_props/swisskegg_agree.props"` |
| `public static final java.lang.String` | `ARFF_PROPS_TREMBL_KEGG` | `"data/properties_files/arff_props/tremblkegg_agree.props"` |
| `public static final java.lang.String` | `ARFF_PROPS_VERTEBRATES` | `"data/properties_files/arff_props/vertebrates.props"` |
| `public static final java.lang.String` | `ARFF_TABLE` | `"arff"` |
| `public static final java.lang.String` | `AUTHOR` | `"Luna De Ferrari - luna.deferrari[at]ed.ac.uk"` |
| `public static final java.lang.String` | `MACHINE_LEARNING_LOCAL_DB_PROPS` | `"data/properties_files/machine_learning_props/mulan_results_localhost.props"` |
| `public static final java.lang.String` | `MACHINE_LEARNING_PROPS` | `"data/properties_files/machine_learning_props/mulan_results_remote_server.props"` |
| `public static final java.lang.String` | `MACHINE_LEARNING_REMOTE_DB_PROPS` | `"data/properties_files/machine_learning_props/mulan_results_remote_server.props"` |
| `public static final java.lang.String` | `MODELS_PATH` | `"data/model/"` |
| `public static final java.lang.String` | `PREDICTIONS_PATH` | `"data/predictions/"` |
| `public static final java.lang.String` | `PROJECT_NAME` | `"enzml"` |
| `public static final java.lang.String` | `RESULTS_DIRECTORY` | `"data/results/"` |
| `public static final java.lang.String` | `SOFTWARE_PROJECT_NAME` | `"EnzML"` |
| `public static final java.lang.String` | `VERSION` | `"2011_01"` |
| `public static final java.lang.String` | `WEB_PAGE` | `" http://mook.inf.ed.ac.uk/twiki/bin/view.cgi/PublicCSB/TaWeka"` |

| uk.ac.ed.inf.enzml.mulan.MulanArffProperties | | |
| --- | --- | --- |
| `public static final java.lang.String` | `XML_QUERY_PROP` | `"xml_query"` |

| uk.ac.ed.inf.enzml.mulan.MulanAttributeFactory | | |
| --- | --- | --- |
| `public static final double` | `ABSENT_OPTION` | `0.0` |
| `public static final double` | `PRESENT_OPTION` | `1.0` |

| uk.ac.ed.inf.enzml.mulan.database.MulanDbCreator | | |
| --- | --- | --- |
| `public static final java.lang.String` | `MULAN_DATABASE_NAME` | `"mulan"` |

| uk.ac.ed.inf.enzml.mulan.learn.ExperimentTable | | |
| --- | --- | --- |
| `public static final java.lang.String` | `ALGORITHM` | `"algorithm"` |
| `public static final java.lang.String` | `END_TIMESTAMP` | `"end_time"` |
| `public static final java.lang.String` | `EXPERIMENTS_TABLE_NAME` | `"mulan_results"` |
| `public static final java.lang.String` | `RUN` | `"run"` |
| `public static final java.lang.String` | `START_TIMESTAMP` | `"start_time"` |
| `public static final java.lang.String` | `TEST_ARFF_ID` | `"test_arff_id"` |
| `public static final java.lang.String` | `TRAIN_ARFF_ID` | `"train_arff_id"` |

| uk.ac.ed.inf.enzml.mulan.predict.MulanPredict | | |
| --- | --- | --- |
| `public static final java.lang.String` | `ATTRIBUTE_ID` | `"attribute_id"` |
| `public static final java.lang.String` | `ATTRIBUTE_NAME` | `"attribute"` |
| `public static final java.lang.String` | `CONFIDENCE` | `"confidence"` |
| `public static final java.lang.String` | `INSTANCE_ID` | `"instance_id"` |
| `public static final java.lang.String` | `INSTANCE_NAME` | `"instance"` |

| uk.ac.ed.inf.enzml.weka.ArffProperties | | |
| --- | --- | --- |
| `public static final java.lang.String` | `ARFF_FILE_PATH_PROP` | `"arff_file_path"` |
| `public static final java.lang.String` | `CLASS_NAME_PROP` | `"class_name"` |
| `public static final java.lang.String` | `DATA_PROPERTIES_FILE_PATH_PROP` | `"properties_file_path"` |
| `public static final java.lang.String` | `DATASET_NAME_PROP` | `"dataset"` |
| `public static final java.lang.String` | `DESCRIPTION_PROP` | `"description"` |
| `public static final java.lang.String` | `INSTANCE_ATTRIBUTE_QUERY_PROP` | `"instance_attribute_query"` |
| `public static final java.lang.String` | `INSTANCE_CLASS_QUERY_PROP` | `"instance_class_query"` |
| `public static final java.lang.String` | `NUMBER_OF_ATTRIBUTES` | `"attributes"` |
| `public static final java.lang.String` | `NUMBER_OF_CLASS_VALUES` | `"class_values"` |
| `public static final int` | `NUMBER_OF_CORE_PROPERTIES` | `9` |
| `public static final java.lang.String` | `NUMBER_OF_INSTANCES` | `"instances"` |
| `public static final int` | `NUMBER_OF_STATISTICS_PROPERTIES` | `3` |
| `public static final java.lang.String` | `TIMESTAMP_FIELD` | `"timestamp"` |

| uk.ac.ed.inf.enzml.weka.ArffPropsTable | | |
| --- | --- | --- |
| `public static final java.lang.String` | `ARFF_ID_COLUMN` | `"id"` |
| `public static final java.lang.String` | `TABLE_NAME` | `"arff"` |

| uk.ac.ed.inf.enzml.weka.AttributeFactory | | |
| --- | --- | --- |
| `public static final java.lang.String` | `ABSENT_OPTION` | `"n"` |
| `public static final java.lang.String` | `PRESENT_OPTION` | `"y"` |

| uk.ac.ed.inf.enzml.weka.DataSetManager | | |
| --- | --- | --- |
| `public static final java.lang.String` | `DUMMY_VALUE` | `"dummy"` |

---


|  |  |  |  |  |  |  |  |  |  |  |
| --- | --- | --- | --- | --- | --- | --- | --- | --- | --- | --- |
| |  |  |  |  |  |  |  |  | | --- | --- | --- | --- | --- | --- | --- | --- | | **Overview** | Package | Class | Use | **Tree** | **Deprecated** | **Index** | **Help** | | |  |
| PREV   NEXT | **FRAMES**    **NO FRAMES**     **All Classes** |


---
