# Supplementary material for: EnzML: multi-label prediction of enzyme classes using InterPro signatures
Source: BMC Bioinformatics. 2012 Apr 25;13:61. doi: 10.1186/1471-2105-13-61 (PMC3483700; doi:10.1186/1471-2105-13-61)
Supplement: Addtional file 5 — The Java code to format the data files, evaluate and predict. The file enzml_java_code.tar.gz contains the Java code used to format database data to ARFF and XML formats, to execute cross and train-test (jackknife) evaluations and to record evaluation results to database. More information is included in the readme.txt file and the Javadoc files. The code can be used with a MySQL database. To use a different database software, other JDBC drivers might be required. [file 1471-2105-13-61-S5.gz › java_code/enzml2011/doc/overview-tree.html]

Class Hierarchy


---


|  |  |  |  |  |  |  |  |  |  |  |
| --- | --- | --- | --- | --- | --- | --- | --- | --- | --- | --- |
| |  |  |  |  |  |  |  |  | | --- | --- | --- | --- | --- | --- | --- | --- | | **Overview** | Package | Class | Use | **Tree** | **Deprecated** | **Index** | **Help** | | |  |
| PREV   NEXT | **FRAMES**    **NO FRAMES**     **All Classes** |


---


## Hierarchy For All Packages

**Package Hierarchies:**: test, test.dataharness, test.mulan, test.mulan.attributesfilter, test.mulan.learn, test.mulan.learn.database, test.mulan.learn.traintest, test.mulan.predict, test.weka, uk.ac.ed.inf.enzml, uk.ac.ed.inf.enzml.mulan, uk.ac.ed.inf.enzml.mulan.attributesfilter, uk.ac.ed.inf.enzml.mulan.database, uk.ac.ed.inf.enzml.mulan.learn, uk.ac.ed.inf.enzml.mulan.learn.traintest, uk.ac.ed.inf.enzml.mulan.predict, uk.ac.ed.inf.enzml.weka

---

## Class Hierarchy

- java.lang.Object
  - weka.core.AbstractInstance (implements weka.core.Instance, weka.core.RevisionHandler, java.io.Serializable)
    - weka.core.SparseInstance
      - uk.ac.ed.inf.enzml.weka.**IdentifiedSparseInstance**- test.**AllArffTests**- test.dataharness.**AllDataTests**- test.**AllMulanArffTests**- test.**AllMulanLearningTests**- test.**AllMulanPredictionTests**- test.**AllMulanTests**- test.**AllPreliminaryTests**- test.**AllTests**- test.**AllTrainTestsTests**- test.**AllUtilsTests**- test.mulan.attributesfilter.**ArffGeneratorForFilterTests**- junit.framework.Assert
                            - junit.framework.TestCase (implements junit.framework.Test)
                              - test.dataharness.**ArffPropsFilesTest**- test.dataharness.**ArffPropsOneTest**- test.dataharness.**ArffPropsQueriesOneTest**- test.dataharness.**ArffPropsQueriesTest**- test.dataharness.**ArffPropsQueriesTwoTest**- test.weka.**ArffPropsTableManagerTest**- test.weka.**ArffPropsTableReaderTest**- test.weka.**ArffPropsTableTest**- test.dataharness.**ArffPropsTwoTest**- test.weka.**ArffTest**- test.weka.**AttributeFactoryTest**- test.mulan.attributesfilter.**AttributesFilteredArffTest**- test.mulan.attributesfilter.**AttributesFilterTest**- test.dataharness.**CreateDataTable**
                                                          - test.dataharness.**DataTableOneTest**- test.dataharness.**DataTableThreeTest**- test.dataharness.**DataTableTwoTest**- test.mulan.learn.**CrossEvaluatorTest**- test.dataharness.**DatabaseTest**- test.weka.**DataSetCheckerTest**- test.weka.**DataSetDbLoaderTest**- test.weka.**DataSetGeneratorTest**- test.weka.**DataSetManagerTest**- test.weka.**DataSetWriterTest**- test.mulan.learn.traintest.**EvaluationMetricsTest**- test.mulan.learn.**EvaluationParametersTest**- test.mulan.learn.**ExperimenterTest**- test.mulan.learn.**ExperimentTableTest**- test.weka.**InstancesFillerTest**- test.mulan.learn.**LearnerTest**- test.mulan.**MulanArffRecordTest**- test.mulan.**MulanArffTest**- test.mulan.**MulanDataSetDbLoaderTest**- test.mulan.**MulanDataSetGeneratorTest**- test.mulan.**MulanDataSetManagerTest**- test.mulan.**MulanDataSetWriterTest**- test.mulan.learn.database.**MulanDbCreatorTest**- test.mulan.learn.database.**MulanDbManagerTest**- test.mulan.learn.database.**MulanDbReaderTest**- test.mulan.learn.database.**MulanDbWriterTest**- test.mulan.**MulanInstancesFillerTest**- test.mulan.predict.**MulanPredictTest**- test.mulan.learn.**ResultCheckerTest**- test.mulan.learn.**ResultsFormatterTest**- test.mulan.learn.**ResultsSaverTest**- test.mulan.learn.**SerializerTest**- test.dataharness.**TestProjectParametersTest**- test.mulan.learn.traintest.**TrainTestEvaluatorTest**- test.mulan.learn.traintest.**TrainTestExperimenterTest**- test.weka.**WekaTest**- uk.ac.ed.inf.enzml.weka.**AttributeFactory**
                              - uk.ac.ed.inf.enzml.mulan.**MulanAttributeFactory**
                                - uk.ac.ed.inf.enzml.mulan.attributesfilter.**AttributesFilteredAttributeFactory**- test.mulan.attributesfilter.**AttributesFilteredDataSetGeneratorTest**- uk.ac.ed.inf.enzml.mulan.**AttributeUtils**- test.dataharness.**DataOne**- uk.ac.ed.inf.enzml.weka.**DataSetChecker**- uk.ac.ed.inf.enzml.weka.**DataSetDbLoader**
                                        - uk.ac.ed.inf.enzml.mulan.**MulanDataSetDbLoader**- uk.ac.ed.inf.enzml.weka.**DataSetGenerator**
                                          - uk.ac.ed.inf.enzml.mulan.**MulanDataSetGenerator**
                                            - uk.ac.ed.inf.enzml.mulan.attributesfilter.**AttributesFilteredDataSetGenerator**- uk.ac.ed.inf.enzml.weka.**DataSetWriter**- test.dataharness.**DataTwo**- uk.ac.ed.inf.utils.database.DbManaged
                                                - uk.ac.ed.inf.utils.database.DbCreator
                                                  - uk.ac.ed.inf.enzml.mulan.database.**MulanDbCreator**- uk.ac.ed.inf.utils.database.DbReader<T,U>
                                                    - uk.ac.ed.inf.enzml.mulan.database.**MulanDbReader**- uk.ac.ed.inf.utils.database.DbWriter
                                                      - uk.ac.ed.inf.enzml.mulan.database.**MulanDbWriter**- uk.ac.ed.inf.utils.database.TableManager
                                                        - uk.ac.ed.inf.enzml.weka.**ArffPropsTableManager**- uk.ac.ed.inf.enzml.weka.**ArffPropsTableReader**- uk.ac.ed.inf.enzml.mulan.learn.**EvaluationParameters**- mulan.evaluation.Evaluator
                                                    - uk.ac.ed.inf.enzml.mulan.learn.**EvaluatorNoAUC**
                                                      - uk.ac.ed.inf.enzml.mulan.learn.**MulanCrossEvaluator**
                                                        - uk.ac.ed.inf.enzml.mulan.learn.traintest.**TrainTestEvaluator**
                                                          - uk.ac.ed.inf.enzml.mulan.learn.traintest.**TrainTestEvaluatorSerialised**- uk.ac.ed.inf.utils.Initialised (implements uk.ac.ed.inf.utils.Initialisable)
                                                      - uk.ac.ed.inf.enzml.weka.**Arff**
                                                        - uk.ac.ed.inf.enzml.mulan.**MulanArff**
                                                          - uk.ac.ed.inf.enzml.mulan.attributesfilter.**AttributesFilteredArff**- uk.ac.ed.inf.enzml.weka.**ArffProperties**
                                                          - uk.ac.ed.inf.enzml.mulan.**MulanArffProperties**- uk.ac.ed.inf.enzml.mulan.attributesfilter.**AttributesFilter**- uk.ac.ed.inf.enzml.mulan.**MulanArffRecord**- uk.ac.ed.inf.enzml.mulan.learn.**MulanCrossExperimenter**
                                                                - uk.ac.ed.inf.enzml.mulan.learn.traintest.**TrainTestExperimenter**
                                                                  - uk.ac.ed.inf.enzml.mulan.learn.traintest.**TrainTestExperimenterSerialized**- uk.ac.ed.inf.enzml.mulan.predict.**MulanPredict**
                                                                  - uk.ac.ed.inf.enzml.mulan.predict.**MulanPredictWithTrainedModel**- uk.ac.ed.inf.enzml.weka.**InstancesFiller**
                                                        - uk.ac.ed.inf.enzml.mulan.**MulanInstancesFiller**- uk.ac.ed.inf.enzml.mulan.**InstanceUtils**- uk.ac.ed.inf.utils.database.Manager
                                                            - uk.ac.ed.inf.enzml.weka.**DataSetManager**
                                                              - uk.ac.ed.inf.enzml.mulan.**MulanDataSetManager**
                                                                - uk.ac.ed.inf.enzml.mulan.attributesfilter.**AttributesFilteredDataSetManager**- uk.ac.ed.inf.utils.database.DbManager
                                                                - uk.ac.ed.inf.enzml.mulan.database.**MulanDbManager**- test.mulan.predict.**MulanEmitPredictions**- uk.ac.ed.inf.enzml.mulan.learn.**MulanLearner**- uk.ac.ed.inf.enzml.mulan.learn.**MulanLearners**- uk.ac.ed.inf.enzml.mulan.learn.**MulanSerializer**- uk.ac.ed.inf.enzml.mulan.learn.traintest.**MulanTrain**- uk.ac.ed.inf.enzml.**ProjectParameters**
                                                                        - test.dataharness.**TestProjectParameters**- uk.ac.ed.inf.enzml.mulan.learn.**ResultsFormatter**- uk.ac.ed.inf.enzml.mulan.learn.**ResultsSaver**- uk.ac.ed.inf.utils.database.Table
                                                                              - uk.ac.ed.inf.enzml.weka.**ArffPropsTable**- uk.ac.ed.inf.enzml.mulan.learn.**ExperimentTable**- uk.ac.ed.inf.enzml.mulan.learn.traintest.**TrainTestFullRun**

---


|  |  |  |  |  |  |  |  |  |  |  |
| --- | --- | --- | --- | --- | --- | --- | --- | --- | --- | --- |
| |  |  |  |  |  |  |  |  | | --- | --- | --- | --- | --- | --- | --- | --- | | **Overview** | Package | Class | Use | **Tree** | **Deprecated** | **Index** | **Help** | | |  |
| PREV   NEXT | **FRAMES**    **NO FRAMES**     **All Classes** |


---
