# Supplementary material for: EnzML: multi-label prediction of enzyme classes using InterPro signatures
Source: BMC Bioinformatics. 2012 Apr 25;13:61. doi: 10.1186/1471-2105-13-61 (PMC3483700; doi:10.1186/1471-2105-13-61)
Supplement: Addtional file 5 — The Java code to format the data files, evaluate and predict. The file enzml_java_code.tar.gz contains the Java code used to format database data to ARFF and XML formats, to execute cross and train-test (jackknife) evaluations and to record evaluation results to database. More information is included in the readme.txt file and the Javadoc files. The code can be used with a MySQL database. To use a different database software, other JDBC drivers might be required. [file 1471-2105-13-61-S5.gz › java_code/utils/doc/overview-summary.html]

Overview


---


|  |  |  |  |  |  |  |  |  |  |  |
| --- | --- | --- | --- | --- | --- | --- | --- | --- | --- | --- |
| |  |  |  |  |  |  |  |  | | --- | --- | --- | --- | --- | --- | --- | --- | | **Overview** | Package | Class | Use | **Tree** | **Deprecated** | **Index** | **Help** | | |  |
| PREV   NEXT | **FRAMES**    **NO FRAMES**     **All Classes** |


---

| **Packages** | |
| --- | --- |
| **cern.colt** |  |
| **cern.colt.function** |  |
| **cern.jet.random** |  |
| **cern.jet.random.engine** |  |
| **edu.cornell.lassp.houle.RngPack** |  |
| **test** |  |
| **test.database** |  |
| **test.maputils** |  |
| **test.setutils** |  |
| **uk.ac.ed.inf.utils** |  |
| **uk.ac.ed.inf.utils.database** |  |
| **uk.ac.ed.inf.utils.diff** |  |
| **uk.ac.ed.inf.utils.guiutils** |  |
| **uk.ac.ed.inf.utils.maputils** |  |
| **uk.ac.ed.inf.utils.setutils** |  |
| **uk.ac.ed.inf.utils.stats** |  |
| **uk.ac.ed.inf.utils.stats.tests** |  |
| **uk.ac.ed.inf.utils.webutils** |  |
| **uk.ac.ed.inf.utils.webutils.simpledomparser** |  |

---


|  |  |  |  |  |  |  |  |  |  |  |
| --- | --- | --- | --- | --- | --- | --- | --- | --- | --- | --- |
| |  |  |  |  |  |  |  |  | | --- | --- | --- | --- | --- | --- | --- | --- | | **Overview** | Package | Class | Use | **Tree** | **Deprecated** | **Index** | **Help** | | |  |
| PREV   NEXT | **FRAMES**    **NO FRAMES**     **All Classes** |


---
