# Supplementary material for: EnzML: multi-label prediction of enzyme classes using InterPro signatures
Source: BMC Bioinformatics. 2012 Apr 25;13:61. doi: 10.1186/1471-2105-13-61 (PMC3483700; doi:10.1186/1471-2105-13-61)
Supplement: Addtional file 5 — The Java code to format the data files, evaluate and predict. The file enzml_java_code.tar.gz contains the Java code used to format database data to ARFF and XML formats, to execute cross and train-test (jackknife) evaluations and to record evaluation results to database. More information is included in the readme.txt file and the Javadoc files. The code can be used with a MySQL database. To use a different database software, other JDBC drivers might be required. [file 1471-2105-13-61-S5.gz › java_code/utils/doc/allclasses-frame.html]

All Classes


**All Classes**
  

|  |
| --- |
| AbstractContinousDistribution   AbstractDistribution   AllDatabaseUtilsTests   AllStatsUtilsTests   AllUtilsTests   ArrayUtils   ClientHttpRequestUtils   CollectionUtils   CollectionUtilsTest   CommandLineMenu   CommandOption   Data   DbConn   DbConnPropsTest   DbCreator   DbCreatorTest   DbManaged   DbManager   DbManagerTest   DbReader   DbReaderTest   DbUtils   DbUtilsTest   DbWriter   DbWriterTest   Diff   Difference   *DoubleFunction*   EntrezUtils   FileUtils   FileUtilsTest   GuiUtils   IndexedOneToManyMap   IndexedOneToManyMapTest   *Initialisable*   Initialised   *IntFunction*   ListUtils   ListUtilsTest   LoggerCreator   LogUtils   Managed   Manager   MapUtils   MapUtilsTest   MersenneTwister   NumberUtils   NumberUtilsTest   OneToManyMap   OneToManyMapTest   OptionUtils   Pareto   ParetoTest   PathUtils   PersistentObject   PropertiesUtils   PseudoTruncatedPareto   PseudoTruncatedParetoSingleton   PseudoTruncatedParetoTest   RandomApp   RandomElement   RandomEngine   RandomJava   RandomSeedable   RandomShuffle   RandomUtilsTest   Ranecu   Ranlux   Ranmar   ReflectionUtils   ReflectionUtilsTest   RegExpUtils   RegExpUtilsTest   ResultSetUtils   Set   SetTest   SetUtils   SimpleDOMParser   SimpleDOMParserTest   SimpleRadioButtonPanel   SqlUtils   SqlUtilsTest   StatUtils   StatUtilsTest   StringUtils   StringUtilsTest   SupersetsManager   SupersetsManagerTest   Table   TableColumn   TableCreator   TableCreatorTest   TableManaged   TableManager   TableManagerTest   TableMap   TableMapTest   TableReader   TableReaderTest   TableRow   TableRowTest   TableTest   TableWriter   TableWriterTest   TimeUtils   TimeUtilsTest   TruncatedPareto   TruncatedParetoTest   UniformRandomSingleton   UniformRandomSingletonTest   UniformRandomUtils   UniformRandomUtilsTest   Utils   UtilsTest   VectorUtils   WebUtils   WebUtilsTest   XmlNode   XmlNodeTest   XmlSearcher   XmlSearcherTest   XMLUtils   XmlUtilsTest |
