# Supplementary material for: EnzML: multi-label prediction of enzyme classes using InterPro signatures
Source: BMC Bioinformatics. 2012 Apr 25;13:61. doi: 10.1186/1471-2105-13-61 (PMC3483700; doi:10.1186/1471-2105-13-61)
Supplement: Addtional file 5 — The Java code to format the data files, evaluate and predict. The file enzml_java_code.tar.gz contains the Java code used to format database data to ARFF and XML formats, to execute cross and train-test (jackknife) evaluations and to record evaluation results to database. More information is included in the readme.txt file and the Javadoc files. The code can be used with a MySQL database. To use a different database software, other JDBC drivers might be required. [file 1471-2105-13-61-S5.gz › java_code/utils/doc/serialized-form.html]

Serialized Form


---


|  |  |  |  |  |  |  |  |  |  |  |
| --- | --- | --- | --- | --- | --- | --- | --- | --- | --- | --- |
| |  |  |  |  |  |  |  |  | | --- | --- | --- | --- | --- | --- | --- | --- | | **Overview** | Package | Class | Use | **Tree** | **Deprecated** | **Index** | **Help** | | |  |
| PREV   NEXT | **FRAMES**    **NO FRAMES**     **All Classes** |


---


# Serialized Form


---

| **Package** **cern.colt** |
| --- |

| **Class cern.colt.PersistentObject extends java.lang.Object implements Serializable** | |
| --- | --- |

---

| **Package** **cern.jet.random** |
| --- |

| **Class cern.jet.random.AbstractContinousDistribution extends AbstractDistribution implements Serializable** | |
| --- | --- |

| **Class cern.jet.random.AbstractDistribution extends PersistentObject implements Serializable** | |
| --- | --- |

| **Serialized Fields** |
| --- |

### randomGenerator

```
RandomElement randomGenerator
```

| **Class cern.jet.random.Pareto extends AbstractContinousDistribution implements Serializable** | |
| --- | --- |

| **Serialized Fields** |
| --- |

### pwr

```
double pwr
```

---

### scale

```
double scale
```

---

### shape

```
double shape
```

---

| **Package** **cern.jet.random.engine** |
| --- |

| **Class cern.jet.random.engine.MersenneTwister extends RandomEngine implements Serializable** | |
| --- | --- |

| **Serialized Fields** |
| --- |

### mt

```
int[] mt
```

---

### mti

```
int mti
```

| **Class cern.jet.random.engine.RandomEngine extends RandomSeedable implements Serializable** | |
| --- | --- |

---

| **Package** **edu.cornell.lassp.houle.RngPack** |
| --- |

| **Class edu.cornell.lassp.houle.RngPack.RandomElement extends PersistentObject implements Serializable** | |
| --- | --- |

| **Serialized Fields** |
| --- |

### BMoutput

```
double BMoutput
```

---

### BMoutputAvailable

```
boolean BMoutputAvailable
```

| **Class edu.cornell.lassp.houle.RngPack.RandomJava extends RandomElement implements Serializable** | |
| --- | --- |

| **Class edu.cornell.lassp.houle.RngPack.RandomSeedable extends RandomElement implements Serializable** | |
| --- | --- |

| **Class edu.cornell.lassp.houle.RngPack.RandomShuffle extends RandomElement implements Serializable** | |
| --- | --- |

| **Serialized Fields** |
| --- |

### deck

```
double[] deck
```

---

### decksize

```
int decksize
```

---

### generatorA

```
RandomElement generatorA
```

---

### generatorB

```
RandomElement generatorB
```

| **Class edu.cornell.lassp.houle.RngPack.Ranecu extends RandomSeedable implements Serializable** | |
| --- | --- |

| **Serialized Fields** |
| --- |

### iseed1

```
int iseed1
```

---

### iseed2

```
int iseed2
```

| **Class edu.cornell.lassp.houle.RngPack.Ranlux extends RandomSeedable implements Serializable** | |
| --- | --- |

| **Serialized Fields** |
| --- |

### diagOn

```
boolean diagOn
```

---

### in24

```
int in24
```

---

### kount

```
int kount
```

---

### mkount

```
int mkount
```

---

### i24

```
int i24
```

---

### j24

```
int j24
```

---

### iseeds

```
int[] iseeds
```

---

### isdext

```
int[] isdext
```

---

### next

```
int[] next
```

---

### luxlev

```
int luxlev
```

---

### nskip

```
int nskip
```

---

### inseed

```
int inseed
```

---

### jseed

```
int jseed
```

---

### seeds

```
float[] seeds
```

---

### carry

```
float carry
```

---

### twom24

```
float twom24
```

---

### twom12

```
float twom12
```

| **Class edu.cornell.lassp.houle.RngPack.Ranmar extends RandomSeedable implements Serializable** | |
| --- | --- |

| **Serialized Fields** |
| --- |

### c

```
double c
```

---

### cd

```
double cd
```

---

### cm

```
double cm
```

---

### u

```
double[] u
```

---

### uvec

```
double[] uvec
```

---

### i97

```
int i97
```

---

### j97

```
int j97
```

---

| **Package** **uk.ac.ed.inf.utils.guiutils** |
| --- |

| **Class uk.ac.ed.inf.utils.guiutils.GuiUtils extends javax.swing.JFrame implements Serializable** | |
| --- | --- |

---

| **Package** **uk.ac.ed.inf.utils.stats** |
| --- |

| **Class uk.ac.ed.inf.utils.stats.PseudoTruncatedPareto extends TruncatedPareto implements Serializable** | |
| --- | --- |

| **Serialized Fields** |
| --- |

### m\_pseudoRandomNumbers

```
java.lang.Integer[] m_pseudoRandomNumbers
```

| **Class uk.ac.ed.inf.utils.stats.TruncatedPareto extends Pareto implements Serializable** | |
| --- | --- |

| **Serialized Fields** |
| --- |

### m\_offset

```
int m_offset
```

:   Offset to match other numbering systems. For example, the Java arrays/map
    indexing system starts at 0, while the Pareto extracted integers start at
    2. This conversion will need a -2 offset.

---

### m\_truncationThreshold

```
int m_truncationThreshold
```

:   the truncation threshold, no numbers extracted can be above it

---


|  |  |  |  |  |  |  |  |  |  |  |
| --- | --- | --- | --- | --- | --- | --- | --- | --- | --- | --- |
| |  |  |  |  |  |  |  |  | | --- | --- | --- | --- | --- | --- | --- | --- | | **Overview** | Package | Class | Use | **Tree** | **Deprecated** | **Index** | **Help** | | |  |
| PREV   NEXT | **FRAMES**    **NO FRAMES**     **All Classes** |


---
