# Supplementary material for: EnzML: multi-label prediction of enzyme classes using InterPro signatures
Source: BMC Bioinformatics. 2012 Apr 25;13:61. doi: 10.1186/1471-2105-13-61 (PMC3483700; doi:10.1186/1471-2105-13-61)
Supplement: Addtional file 5 — The Java code to format the data files, evaluate and predict. The file enzml_java_code.tar.gz contains the Java code used to format database data to ARFF and XML formats, to execute cross and train-test (jackknife) evaluations and to record evaluation results to database. More information is included in the readme.txt file and the Javadoc files. The code can be used with a MySQL database. To use a different database software, other JDBC drivers might be required. [file 1471-2105-13-61-S5.gz › java_code/utils/doc/constant-values.html]

Constant Field Values


---


|  |  |  |  |  |  |  |  |  |  |  |
| --- | --- | --- | --- | --- | --- | --- | --- | --- | --- | --- |
| |  |  |  |  |  |  |  |  | | --- | --- | --- | --- | --- | --- | --- | --- | | **Overview** | Package | Class | Use | **Tree** | **Deprecated** | **Index** | **Help** | | |  |
| PREV   NEXT | **FRAMES**    **NO FRAMES**     **All Classes** |


---


# Constant Field Values


---

**Contents**

- cern.jet.\*- edu.cornell.\*- test.\*- uk.ac.\*

| cern.jet.\* |
| --- |

| cern.jet.random.engine.MersenneTwister | | |
| --- | --- | --- |
| `public static final int` | `DEFAULT_SEED` | `4357` |

| edu.cornell.\* |
| --- |

| edu.cornell.lassp.houle.RngPack.Ranlux | | |
| --- | --- | --- |
| `public static final int` | `lxdflt` | `3` |
| `public static final int` | `maxlev` | `4` |

| test.\* |
| --- |

| test.Data | | |
| --- | --- | --- |
| `public static final java.lang.String` | `KEY1` | `"key1"` |
| `public static final java.lang.String` | `KEY2` | `"key2"` |
| `public static final java.lang.String` | `KEY3` | `"key3"` |
| `public static final java.lang.String` | `KEYA` | `"keyA"` |
| `public static final java.lang.String` | `VAL1` | `"value1"` |
| `public static final java.lang.String` | `VAL2` | `"value2"` |
| `public static final java.lang.String` | `VAL3` | `"value3"` |
| `public static final java.lang.String` | `VALA` | `"valueA"` |
| `public static final java.lang.String` | `VALB` | `"valueB"` |

| test.database.\* |
| --- |

| test.database.TableCreatorTest | | |
| --- | --- | --- |
| `public static final java.lang.String` | `TABLE1` | `"table1"` |

| uk.ac.\* |
| --- |

| uk.ac.ed.inf.utils.TimeUtils | | |
| --- | --- | --- |
| `public static final java.lang.String` | `DAY` | `"day"` |
| `public static final java.lang.String` | `HOUR` | `"hour"` |
| `public static final java.lang.String` | `MILLISECONDS` | `"milliseconds"` |
| `public static final java.lang.String` | `MINUTE` | `"minute"` |
| `public static final java.lang.String` | `MONTH` | `"month"` |
| `public static final java.lang.String` | `SECOND` | `"second"` |
| `public static final java.lang.String` | `YEAR` | `"year"` |

| uk.ac.ed.inf.utils.database.DbConn | | |
| --- | --- | --- |
| `public static final java.lang.String` | `DATABASE_NAME_PROP` | `"database_name"` |
| `public static final java.lang.String` | `DATABASE_TYPE_PROP` | `"database_type"` |
| `public static final java.lang.String` | `HOST_PROP` | `"host"` |
| `public static final java.lang.String` | `JDBC_DRIVER_PROP` | `"jdbc_driver"` |
| `public static final java.lang.String` | `PASSWORD_PROP` | `"pswd"` |
| `public static final java.lang.String` | `PORT_PROP` | `"port"` |
| `public static final java.lang.String` | `USER_PROP` | `"user"` |

| uk.ac.ed.inf.utils.database.DbUtils | | |
| --- | --- | --- |
| `public static final java.lang.String` | `AUTO_INCREMENT_SQL_DATATYPE` | `"INT AUTO_INCREMENT"` |
| `public static final java.lang.String` | `BOOLEAN_SQL_DATATYPE` | `"BOOLEAN"` |
| `public static final java.lang.String` | `CHAR_2_SQL_DATATYPE` | `"CHAR(2)"` |
| `public static final java.lang.String` | `CHAR_4_SQL_DATATYPE` | `"CHAR(4)"` |
| `public static final java.lang.String` | `DOUBLE_QUOTE_CHARACTER` | `"\""` |
| `public static final java.lang.String` | `DOUBLE_SQL_DATATYPE` | `"DOUBLE"` |
| `public static final java.lang.String` | `END_TIMESTAMP` | `"end_time"` |
| `public static final java.lang.String` | `ID` | `"id"` |
| `public static final java.lang.String` | `INTEGER_SQL_DATATYPE` | `"INT"` |
| `public static final java.lang.String` | `REVERSED_PRIME_CHARACTER` | `` "`" `` |
| `public static final java.lang.String` | `SINGLE_QUOTE_CHARACTER` | `"\'"` |
| `public static final java.lang.String` | `START_TIMESTAMP` | `"start_time"` |
| `public static final java.lang.String` | `TIMESTAMP_DOWNLOADED` | `"time_downloaded"` |
| `public static final java.lang.String` | `TIMESTAMP_SQL_DATATYPE` | `"TIMESTAMP"` |
| `public static final java.lang.String` | `VARCHAR_90_SQL_DATATYPE` | `"VARCHAR(90)"` |

| uk.ac.ed.inf.utils.database.SqlUtils | | |
| --- | --- | --- |
| `public static final int` | `DOUBLE` | `2` |
| `public static final int` | `INTEGER` | `1` |
| `public static final int` | `MAX_BIGINT_LENGTH` | `18` |
| `public static final int` | `MAX_CHAR_LENGTH` | `255` |
| `public static final int` | `MAX_INT_LENGTH` | `9` |
| `public static final int` | `NULL` | `0` |
| `public static final int` | `STRING` | `3` |

| uk.ac.ed.inf.utils.diff.Difference | | |
| --- | --- | --- |
| `public static final int` | `NONE` | `-1` |

| uk.ac.ed.inf.utils.setutils.SupersetsManager | | |
| --- | --- | --- |
| `public static final boolean` | `m_debug` | `false` |

| uk.ac.ed.inf.utils.stats.tests.TruncatedParetoTest | | |
| --- | --- | --- |
| `public static final int` | `THRESHOLD_100` | `100` |

---


|  |  |  |  |  |  |  |  |  |  |  |
| --- | --- | --- | --- | --- | --- | --- | --- | --- | --- | --- |
| |  |  |  |  |  |  |  |  | | --- | --- | --- | --- | --- | --- | --- | --- | | **Overview** | Package | Class | Use | **Tree** | **Deprecated** | **Index** | **Help** | | |  |
| PREV   NEXT | **FRAMES**    **NO FRAMES**     **All Classes** |


---
