# Supplementary material for: EnzML: multi-label prediction of enzyme classes using InterPro signatures
Source: BMC Bioinformatics. 2012 Apr 25;13:61. doi: 10.1186/1471-2105-13-61 (PMC3483700; doi:10.1186/1471-2105-13-61)
Supplement: Addtional file 5 — The Java code to format the data files, evaluate and predict. The file enzml_java_code.tar.gz contains the Java code used to format database data to ARFF and XML formats, to execute cross and train-test (jackknife) evaluations and to record evaluation results to database. More information is included in the readme.txt file and the Javadoc files. The code can be used with a MySQL database. To use a different database software, other JDBC drivers might be required. [file 1471-2105-13-61-S5.gz › java_code/utils/doc/overview-tree.html]

Class Hierarchy


---


|  |  |  |  |  |  |  |  |  |  |  |
| --- | --- | --- | --- | --- | --- | --- | --- | --- | --- | --- |
| |  |  |  |  |  |  |  |  | | --- | --- | --- | --- | --- | --- | --- | --- | | **Overview** | Package | Class | Use | **Tree** | **Deprecated** | **Index** | **Help** | | |  |
| PREV   NEXT | **FRAMES**    **NO FRAMES**     **All Classes** |


---


## Hierarchy For All Packages

**Package Hierarchies:**: cern.colt, cern.colt.function, cern.jet.random, cern.jet.random.engine, edu.cornell.lassp.houle.RngPack, test, test.database, test.maputils, test.setutils, uk.ac.ed.inf.utils, uk.ac.ed.inf.utils.database, uk.ac.ed.inf.utils.diff, uk.ac.ed.inf.utils.guiutils, uk.ac.ed.inf.utils.maputils, uk.ac.ed.inf.utils.setutils, uk.ac.ed.inf.utils.stats, uk.ac.ed.inf.utils.stats.tests, uk.ac.ed.inf.utils.webutils, uk.ac.ed.inf.utils.webutils.simpledomparser

---

## Class Hierarchy

- java.lang.Object
  - test.**AllDatabaseUtilsTests**- test.**AllUtilsTests**- uk.ac.ed.inf.utils.**ArrayUtils**- junit.framework.Assert
          - junit.framework.TestCase (implements junit.framework.Test)
            - uk.ac.ed.inf.utils.stats.tests.**AllStatsUtilsTests**- test.**CollectionUtilsTest**- test.**Data**- test.database.**DbConnPropsTest**- test.database.**DbCreatorTest**- test.database.**DbManagerTest**- test.database.**DbReaderTest**- test.database.**DbUtilsTest**- test.database.**DbWriterTest**- test.maputils.**IndexedOneToManyMapTest**- test.**ListUtilsTest**- test.maputils.**MapUtilsTest**- test.**NumberUtilsTest**- test.maputils.**OneToManyMapTest**- uk.ac.ed.inf.utils.stats.tests.**ParetoTest**- uk.ac.ed.inf.utils.stats.tests.**PseudoTruncatedParetoTest**- uk.ac.ed.inf.utils.stats.tests.**RandomUtilsTest**- test.**ReflectionUtilsTest**- test.**RegExpUtilsTest**- test.setutils.**SetTest**- test.**SimpleDOMParserTest**- test.database.**SqlUtilsTest**- uk.ac.ed.inf.utils.stats.tests.**StatUtilsTest**- test.**StringUtilsTest**- test.setutils.**SupersetsManagerTest**- test.database.**TableCreatorTest**- test.database.**TableManagerTest**- test.maputils.**TableMapTest**- test.database.**TableReaderTest**- test.database.**TableRowTest**- test.database.**TableTest**- test.database.**TableWriterTest**- test.**TimeUtilsTest**- uk.ac.ed.inf.utils.stats.tests.**TruncatedParetoTest**- uk.ac.ed.inf.utils.stats.tests.**UniformRandomSingletonTest**- uk.ac.ed.inf.utils.stats.tests.**UniformRandomUtilsTest**- test.**UtilsTest**- test.**WebUtilsTest**- test.**XmlNodeTest**- test.**XmlSearcherTest**- test.**XmlUtilsTest**- uk.ac.ed.inf.utils.webutils.**ClientHttpRequestUtils**- uk.ac.ed.inf.utils.**CollectionUtils**- uk.ac.ed.inf.utils.guiutils.**CommandLineMenu**- uk.ac.ed.inf.utils.guiutils.**CommandOption**- java.awt.Component (implements java.awt.image.ImageObserver, java.awt.MenuContainer, java.io.Serializable)
                    - java.awt.Container
                      - java.awt.Window (implements javax.accessibility.Accessible)
                        - java.awt.Frame (implements java.awt.MenuContainer)
                          - javax.swing.JFrame (implements javax.accessibility.Accessible, javax.swing.RootPaneContainer, javax.swing.WindowConstants)
                            - uk.ac.ed.inf.utils.guiutils.**GuiUtils**- uk.ac.ed.inf.utils.database.**DbConn**- uk.ac.ed.inf.utils.database.**DbManaged**
                        - uk.ac.ed.inf.utils.database.**DbCreator**- uk.ac.ed.inf.utils.database.**DbReader**<T,U>- uk.ac.ed.inf.utils.database.**DbWriter**- uk.ac.ed.inf.utils.database.**TableManaged**
                                - uk.ac.ed.inf.utils.database.**TableCreator**- uk.ac.ed.inf.utils.database.**TableReader**- uk.ac.ed.inf.utils.database.**TableWriter**- uk.ac.ed.inf.utils.database.**TableManager**- uk.ac.ed.inf.utils.database.**TableRow**- uk.ac.ed.inf.utils.database.**DbUtils**- uk.ac.ed.inf.utils.diff.**Diff**- uk.ac.ed.inf.utils.diff.**Difference**- uk.ac.ed.inf.utils.**EntrezUtils**- uk.ac.ed.inf.utils.**FileUtils**- test.**FileUtilsTest**- uk.ac.ed.inf.utils.**Initialised** (implements uk.ac.ed.inf.utils.Initialisable)- uk.ac.ed.inf.utils.**ListUtils**- uk.ac.ed.inf.utils.**LoggerCreator**- uk.ac.ed.inf.utils.**LogUtils**- uk.ac.ed.inf.utils.database.**Managed**- uk.ac.ed.inf.utils.database.**Manager**
                                                - uk.ac.ed.inf.utils.database.**DbManager**- uk.ac.ed.inf.utils.maputils.**MapUtils**- uk.ac.ed.inf.utils.**NumberUtils**- uk.ac.ed.inf.utils.maputils.**OneToManyMap**<T,U>
                                                      - uk.ac.ed.inf.utils.maputils.**IndexedOneToManyMap**<T,U>- uk.ac.ed.inf.utils.guiutils.**OptionUtils**- uk.ac.ed.inf.utils.**PathUtils**- cern.colt.**PersistentObject** (implements java.lang.Cloneable, java.io.Serializable)
                                                            - cern.jet.random.**AbstractDistribution** (implements cern.colt.function.DoubleFunction, cern.colt.function.IntFunction)
                                                              - cern.jet.random.**AbstractContinousDistribution**
                                                                - cern.jet.random.**Pareto**
                                                                  - uk.ac.ed.inf.utils.stats.**TruncatedPareto**
                                                                    - uk.ac.ed.inf.utils.stats.**PseudoTruncatedPareto**- edu.cornell.lassp.houle.RngPack.**RandomElement** (implements java.lang.Cloneable)
                                                                - edu.cornell.lassp.houle.RngPack.**RandomJava**- edu.cornell.lassp.houle.RngPack.**RandomSeedable**
                                                                    - cern.jet.random.engine.**RandomEngine** (implements cern.colt.function.DoubleFunction, cern.colt.function.IntFunction)
                                                                      - cern.jet.random.engine.**MersenneTwister**- edu.cornell.lassp.houle.RngPack.**Ranecu**- edu.cornell.lassp.houle.RngPack.**Ranlux**- edu.cornell.lassp.houle.RngPack.**Ranmar**- edu.cornell.lassp.houle.RngPack.**RandomShuffle**- uk.ac.ed.inf.utils.**PropertiesUtils**- uk.ac.ed.inf.utils.stats.**PseudoTruncatedParetoSingleton**- edu.cornell.lassp.houle.RngPack.**RandomApp**- uk.ac.ed.inf.utils.**ReflectionUtils**- uk.ac.ed.inf.utils.**RegExpUtils**- uk.ac.ed.inf.utils.database.**ResultSetUtils**- uk.ac.ed.inf.utils.setutils.**Set** (implements java.lang.Comparable<T>)- uk.ac.ed.inf.utils.**SetUtils**- uk.ac.ed.inf.utils.webutils.simpledomparser.**SimpleDOMParser**- uk.ac.ed.inf.utils.guiutils.**SimpleRadioButtonPanel**- uk.ac.ed.inf.utils.database.**SqlUtils**- uk.ac.ed.inf.utils.stats.**StatUtils**- uk.ac.ed.inf.utils.**StringUtils**- uk.ac.ed.inf.utils.setutils.**SupersetsManager**- uk.ac.ed.inf.utils.database.**Table**- uk.ac.ed.inf.utils.database.**TableColumn** (implements java.lang.Comparable<T>)- uk.ac.ed.inf.utils.maputils.**TableMap**- uk.ac.ed.inf.utils.**TimeUtils**- uk.ac.ed.inf.utils.stats.**UniformRandomSingleton**- uk.ac.ed.inf.utils.stats.**UniformRandomUtils**- uk.ac.ed.inf.utils.**Utils**- uk.ac.ed.inf.utils.**VectorUtils**- uk.ac.ed.inf.utils.webutils.**WebUtils**- uk.ac.ed.inf.utils.webutils.simpledomparser.**XmlNode**- uk.ac.ed.inf.utils.webutils.simpledomparser.**XmlSearcher**- uk.ac.ed.inf.utils.webutils.**XMLUtils**

## Interface Hierarchy

- cern.colt.function.**DoubleFunction**- uk.ac.ed.inf.utils.**Initialisable**- cern.colt.function.**IntFunction**

---


|  |  |  |  |  |  |  |  |  |  |  |
| --- | --- | --- | --- | --- | --- | --- | --- | --- | --- | --- |
| |  |  |  |  |  |  |  |  | | --- | --- | --- | --- | --- | --- | --- | --- | | **Overview** | Package | Class | Use | **Tree** | **Deprecated** | **Index** | **Help** | | |  |
| PREV   NEXT | **FRAMES**    **NO FRAMES**     **All Classes** |


---
