# Supplementary material for: EnzML: multi-label prediction of enzyme classes using InterPro signatures
Source: BMC Bioinformatics. 2012 Apr 25;13:61. doi: 10.1186/1471-2105-13-61 (PMC3483700; doi:10.1186/1471-2105-13-61)
Supplement: Addtional file 5 — The Java code to format the data files, evaluate and predict. The file enzml_java_code.tar.gz contains the Java code used to format database data to ARFF and XML formats, to execute cross and train-test (jackknife) evaluations and to record evaluation results to database. More information is included in the readme.txt file and the Javadoc files. The code can be used with a MySQL database. To use a different database software, other JDBC drivers might be required. [file 1471-2105-13-61-S5.gz › java_code/ecmulan/doc/constant-values.html]

Constant Field Values


---


|  |  |  |  |  |  |  |  |  |  |  |
| --- | --- | --- | --- | --- | --- | --- | --- | --- | --- | --- |
| |  |  |  |  |  |  |  |  | | --- | --- | --- | --- | --- | --- | --- | --- | | **Overview** | Package | Class | Use | **Tree** | **Deprecated** | **Index** | **Help** | | |  |
| PREV   NEXT | **FRAMES**    **NO FRAMES**     **All Classes** |


---


# Constant Field Values


---

**Contents**

- uk.ac.\*

| uk.ac.\* |
| --- |

| uk.ac.ed.inf.ec.EcDbWriter | | |
| --- | --- | --- |
| `public static final java.lang.String` | `ANCESTOR_FIELD_NAME` | `"ancestor"` |
| `public static final java.lang.String` | `EC_DATA_TYPE` | `"VARCHAR(13)"` |
| `public static final java.lang.String` | `EC_FIELD_NAME` | `"ec"` |
| `public static final java.lang.String` | `EC_TABLE_NAME` | `"ec_ancestors_no0_nodash"` |

| uk.ac.ed.inf.ec.EcNumberGenerator | | |
| --- | --- | --- |
| `public static final java.lang.String` | `DASH` | `"-"` |
| `public static final java.lang.String` | `DOT` | `"."` |
| `public static final int` | `FULL_HIERARCHY_LENGHT` | `4` |
| `public static final java.lang.String` | `MAX_LEVEL1_CLASS` | `"6"` |
| `public static final java.lang.String` | `MAX_LEVEL1_REGEXP` | `"[1-6]\\."` |
| `public static final java.lang.String` | `MAX_LEVEL2_CLASS` | `"99"` |
| `public static final java.lang.String` | `MAX_LEVEL2_REGEXP` | `"[0-9]{1,2}\\."` |
| `public static final java.lang.String` | `MAX_LEVEL3_CLASS` | `"99"` |
| `public static final java.lang.String` | `MAX_LEVEL3_REGEXP` | `"[0-9]{1,2}\\."` |
| `public static final java.lang.String` | `MAX_LEVEL4_CLASS` | `"999"` |
| `public static final java.lang.String` | `MAX_LEVEL4_REGEXP` | `"n?[0-9]{1,3}"` |

| uk.ac.ed.inf.ec.MulanXml | | |
| --- | --- | --- |
| `public static final java.lang.String` | `LABEL_NAME_ATTRIBUTE` | `"name"` |
| `public static final java.lang.String` | `LABEL_XML_TAG` | `"label"` |
| `public static final java.lang.String` | `MULAN_XML_ROOT_TAG` | `"labels"` |
| `public static final java.lang.String` | `MULAN_XML_ROOT_TAG_ATTRIBUTE_NAME` | `"xmlns"` |
| `public static final java.lang.String` | `MULAN_XML_ROOT_TAG_ATTRIBUTE_VALUE` | `"http://mulan.sourceforge.net/labels"` |

| uk.ac.ed.inf.ec.test.EcDbReaderTest | | |
| --- | --- | --- |
| `public static final java.lang.String` | `GET_EC_QUERY_1` | `"SELECT distinct ec FROM ec_status where status = \'ok\' "` |
| `public static final java.lang.String` | `GET_EC_QUERY_2` | `"SELECT distinct ancestor FROM ec_ancestors_no0_nodash"` |
| `public static final java.lang.String` | `TEST_DB_CONN_PATH` | `"src/uk/ac/ed/inf/ec/dbconnection_test.props"` |

---


|  |  |  |  |  |  |  |  |  |  |  |
| --- | --- | --- | --- | --- | --- | --- | --- | --- | --- | --- |
| |  |  |  |  |  |  |  |  | | --- | --- | --- | --- | --- | --- | --- | --- | | **Overview** | Package | Class | Use | **Tree** | **Deprecated** | **Index** | **Help** | | |  |
| PREV   NEXT | **FRAMES**    **NO FRAMES**     **All Classes** |


---
