# Supplementary material for: EnzML: multi-label prediction of enzyme classes using InterPro signatures
Source: BMC Bioinformatics. 2012 Apr 25;13:61. doi: 10.1186/1471-2105-13-61 (PMC3483700; doi:10.1186/1471-2105-13-61)
Supplement: Addtional file 5 — The Java code to format the data files, evaluate and predict. The file enzml_java_code.tar.gz contains the Java code used to format database data to ARFF and XML formats, to execute cross and train-test (jackknife) evaluations and to record evaluation results to database. More information is included in the readme.txt file and the Javadoc files. The code can be used with a MySQL database. To use a different database software, other JDBC drivers might be required. [file 1471-2105-13-61-S5.gz › java_code/ecmulan/doc/overview-tree.html]

Class Hierarchy


---


|  |  |  |  |  |  |  |  |  |  |  |
| --- | --- | --- | --- | --- | --- | --- | --- | --- | --- | --- |
| |  |  |  |  |  |  |  |  | | --- | --- | --- | --- | --- | --- | --- | --- | | **Overview** | Package | Class | Use | **Tree** | **Deprecated** | **Index** | **Help** | | |  |
| PREV   NEXT | **FRAMES**    **NO FRAMES**     **All Classes** |


---


## Hierarchy For All Packages

**Package Hierarchies:**: uk.ac.ed.inf.ec, uk.ac.ed.inf.ec.test

---

## Class Hierarchy

- java.lang.Object
  - uk.ac.ed.inf.ec.test.**AllTests**- junit.framework.Assert
      - junit.framework.TestCase (implements junit.framework.Test)
        - uk.ac.ed.inf.ec.test.**EcDbReaderTest**- uk.ac.ed.inf.ec.test.**EcDbWriterTest**- uk.ac.ed.inf.ec.test.**EcFullXmlCreatorTest**- uk.ac.ed.inf.ec.test.**EcMulanXmlCreatorTest**- uk.ac.ed.inf.ec.test.**EcNumberGeneratorTest**- uk.ac.ed.inf.ec.test.**EcNumberTest**- uk.ac.ed.inf.ec.test.**MulanLabelTest**- uk.ac.ed.inf.ec.test.**MulanXmlTest**- uk.ac.ed.inf.utils.database.DbManaged
        - uk.ac.ed.inf.utils.database.DbReader<T,U>
          - uk.ac.ed.inf.ec.**EcDbReader**- uk.ac.ed.inf.ec.**EcDbWriter**- uk.ac.ed.inf.ec.**EcFullXmlCreator**
          - uk.ac.ed.inf.ec.**EcMulanXmlCreator**- uk.ac.ed.inf.ec.**EcNumber** (implements java.lang.Comparable<T>)- uk.ac.ed.inf.ec.**EcNumberGenerator**- uk.ac.ed.inf.ec.**MulanXml**- uk.ac.ed.inf.utils.webutils.simpledomparser.XmlNode
                  - uk.ac.ed.inf.ec.**MulanLabel**

---


|  |  |  |  |  |  |  |  |  |  |  |
| --- | --- | --- | --- | --- | --- | --- | --- | --- | --- | --- |
| |  |  |  |  |  |  |  |  | | --- | --- | --- | --- | --- | --- | --- | --- | | **Overview** | Package | Class | Use | **Tree** | **Deprecated** | **Index** | **Help** | | |  |
| PREV   NEXT | **FRAMES**    **NO FRAMES**     **All Classes** |


---
