# Supplementary material for: EnzML: multi-label prediction of enzyme classes using InterPro signatures
Source: BMC Bioinformatics. 2012 Apr 25;13:61. doi: 10.1186/1471-2105-13-61 (PMC3483700; doi:10.1186/1471-2105-13-61)
Supplement: Addtional file 5 — The Java code to format the data files, evaluate and predict. The file enzml_java_code.tar.gz contains the Java code used to format database data to ARFF and XML formats, to execute cross and train-test (jackknife) evaluations and to record evaluation results to database. More information is included in the readme.txt file and the Javadoc files. The code can be used with a MySQL database. To use a different database software, other JDBC drivers might be required. [file 1471-2105-13-61-S5.gz › java_code/enzml2011/doc/index-files/index-17.html]

U-Index


---


|  |  |  |  |  |  |  |  |  |  |  |
| --- | --- | --- | --- | --- | --- | --- | --- | --- | --- | --- |
| |  |  |  |  |  |  |  |  | | --- | --- | --- | --- | --- | --- | --- | --- | | **Overview** | Package | Class | Use | **Tree** | **Deprecated** | **Index** | **Help** | | |  |
| **PREV LETTER**   **NEXT LETTER** | **FRAMES**    **NO FRAMES**     **All Classes** |


A B C D E F G I K L M N P R S T U V W X 

---


## **U**

**uk.ac.ed.inf.enzml** - package uk.ac.ed.inf.enzml: **uk.ac.ed.inf.enzml.mulan** - package uk.ac.ed.inf.enzml.mulan: **uk.ac.ed.inf.enzml.mulan.attributesfilter** - package uk.ac.ed.inf.enzml.mulan.attributesfilter: **uk.ac.ed.inf.enzml.mulan.database** - package uk.ac.ed.inf.enzml.mulan.database: **uk.ac.ed.inf.enzml.mulan.learn** - package uk.ac.ed.inf.enzml.mulan.learn: **uk.ac.ed.inf.enzml.mulan.learn.traintest** - package uk.ac.ed.inf.enzml.mulan.learn.traintest: **uk.ac.ed.inf.enzml.mulan.predict** - package uk.ac.ed.inf.enzml.mulan.predict: **uk.ac.ed.inf.enzml.weka** - package uk.ac.ed.inf.enzml.weka

---


|  |  |  |  |  |  |  |  |  |  |  |
| --- | --- | --- | --- | --- | --- | --- | --- | --- | --- | --- |
| |  |  |  |  |  |  |  |  | | --- | --- | --- | --- | --- | --- | --- | --- | | **Overview** | Package | Class | Use | **Tree** | **Deprecated** | **Index** | **Help** | | |  |
| **PREV LETTER**   **NEXT LETTER** | **FRAMES**    **NO FRAMES**     **All Classes** |


A B C D E F G I K L M N P R S T U V W X 

---
