# Supplementary material for: EnzML: multi-label prediction of enzyme classes using InterPro signatures
Source: BMC Bioinformatics. 2012 Apr 25;13:61. doi: 10.1186/1471-2105-13-61 (PMC3483700; doi:10.1186/1471-2105-13-61)
Supplement: Addtional file 5 — The Java code to format the data files, evaluate and predict. The file enzml_java_code.tar.gz contains the Java code used to format database data to ARFF and XML formats, to execute cross and train-test (jackknife) evaluations and to record evaluation results to database. More information is included in the readme.txt file and the Javadoc files. The code can be used with a MySQL database. To use a different database software, other JDBC drivers might be required. [file 1471-2105-13-61-S5.gz › java_code/enzml2011/doc/index-files/index-1.html]

A-Index


---


|  |  |  |  |  |  |  |  |  |  |  |
| --- | --- | --- | --- | --- | --- | --- | --- | --- | --- | --- |
| |  |  |  |  |  |  |  |  | | --- | --- | --- | --- | --- | --- | --- | --- | | **Overview** | Package | Class | Use | **Tree** | **Deprecated** | **Index** | **Help** | | |  |
| PREV LETTER   **NEXT LETTER** | **FRAMES**    **NO FRAMES**     **All Classes** |


A B C D E F G I K L M N P R S T U V W X 

---


## **A**

**ABSENT\_OPTION** - Static variable in class uk.ac.ed.inf.enzml.mulan.MulanAttributeFactory: **ABSENT\_OPTION** - Static variable in class uk.ac.ed.inf.enzml.weka.AttributeFactory: **addEvaluationResultsToTableRow(TableRow, Evaluation)** - Static method in class uk.ac.ed.inf.enzml.mulan.learn.ResultsFormatter: adds the evaluation results to a table row object **ALGORITHM** - Static variable in class uk.ac.ed.inf.enzml.mulan.learn.ExperimentTable: the name for the algorithm table column **AllArffTests** - Class in test: Class **AllArffTests()** - Constructor for class test.AllArffTests: **AllDataTests** - Class in test.dataharness: Data harness for arff file generation and learning tests. **AllDataTests()** - Constructor for class test.dataharness.AllDataTests: **AllMulanArffTests** - Class in test: Class **AllMulanArffTests()** - Constructor for class test.AllMulanArffTests: **AllMulanLearningTests** - Class in test: Class **AllMulanLearningTests()** - Constructor for class test.AllMulanLearningTests: **AllMulanPredictionTests** - Class in test: Class **AllMulanPredictionTests()** - Constructor for class test.AllMulanPredictionTests: **AllMulanTests** - Class in test: Class **AllMulanTests()** - Constructor for class test.AllMulanTests: **AllPreliminaryTests** - Class in test: Class **AllPreliminaryTests()** - Constructor for class test.AllPreliminaryTests: **AllTests** - Class in test: All tests **AllTests()** - Constructor for class test.AllTests: **AllTrainTestsTests** - Class in test: Class **AllTrainTestsTests()** - Constructor for class test.AllTrainTestsTests: **AllUtilsTests** - Class in test: **AllUtilsTests()** - Constructor for class test.AllUtilsTests: **Arff** - Class in uk.ac.ed.inf.enzml.weka: Creates an ARFF file: checks file properties, loads data from database, fills and writes to file the ARFF attributes, classes and instances, generates auxiliary files if needed. **Arff(String, String)** - Constructor for class uk.ac.ed.inf.enzml.weka.Arff: Apache logger **arff\_EC\_Instance1** - Static variable in class test.mulan.MulanArffTest: **ARFF\_FILE\_PATH\_PROP** - Static variable in class uk.ac.ed.inf.enzml.weka.ArffProperties: the file path where the ARFF file will be written, inclusive of file name. **ARFF\_ID\_COLUMN** - Static variable in class uk.ac.ed.inf.enzml.weka.ArffPropsTable: **ARFF\_PROPS\_ARCHAEA** - Static variable in class uk.ac.ed.inf.enzml.ProjectParameters: **ARFF\_PROPS\_BACTERIA** - Static variable in class uk.ac.ed.inf.enzml.ProjectParameters: **ARFF\_PROPS\_EUKARIA** - Static variable in class uk.ac.ed.inf.enzml.ProjectParameters: **ARFF\_PROPS\_FUNGI** - Static variable in class uk.ac.ed.inf.enzml.ProjectParameters: **ARFF\_PROPS\_HUMAN** - Static variable in class uk.ac.ed.inf.enzml.ProjectParameters: **ARFF\_PROPS\_INVERTEBRATES** - Static variable in class uk.ac.ed.inf.enzml.ProjectParameters: **ARFF\_PROPS\_NOT\_HUMAN** - Static variable in class uk.ac.ed.inf.enzml.ProjectParameters: all swissprot-kegg agree records, except human **ARFF\_PROPS\_PATH** - Static variable in class uk.ac.ed.inf.enzml.ProjectParameters: **ARFF\_PROPS\_PLANTS** - Static variable in class uk.ac.ed.inf.enzml.ProjectParameters: **ARFF\_PROPS\_RANDOM\_100** - Static variable in class uk.ac.ed.inf.enzml.ProjectParameters: **ARFF\_PROPS\_RANDOM\_ARCHAEA** - Static variable in class uk.ac.ed.inf.enzml.ProjectParameters: **ARFF\_PROPS\_RANDOM\_BACTERIA** - Static variable in class uk.ac.ed.inf.enzml.ProjectParameters: **ARFF\_PROPS\_RANDOM\_EUKARIA** - Static variable in class uk.ac.ed.inf.enzml.ProjectParameters: **ARFF\_PROPS\_RANDOM\_FUNGI** - Static variable in class uk.ac.ed.inf.enzml.ProjectParameters: **ARFF\_PROPS\_RANDOM\_INVERTEBRATES** - Static variable in class uk.ac.ed.inf.enzml.ProjectParameters: **ARFF\_PROPS\_RANDOM\_PLANTS** - Static variable in class uk.ac.ed.inf.enzml.ProjectParameters: **ARFF\_PROPS\_RANDOM\_VERTEBRATES** - Static variable in class uk.ac.ed.inf.enzml.ProjectParameters: **ARFF\_PROPS\_SWISSKEGG** - Static variable in class uk.ac.ed.inf.enzml.ProjectParameters: **ARFF\_PROPS\_TREMBL\_KEGG** - Static variable in class uk.ac.ed.inf.enzml.ProjectParameters: **ARFF\_PROPS\_VERTEBRATES** - Static variable in class uk.ac.ed.inf.enzml.ProjectParameters: **ARFF\_TABLE** - Static variable in class uk.ac.ed.inf.enzml.ProjectParameters: The name of the table to save the arff file details to **arffEmptyInstance** - Static variable in class test.dataharness.DataOne: **arffEmptyInstance** - Static variable in class test.dataharness.DataTwo: **ArffGeneratorForFilterTests** - Class in test.mulan.attributesfilter: **ArffGeneratorForFilterTests()** - Constructor for class test.mulan.attributesfilter.ArffGeneratorForFilterTests: **arffInstance1** - Static variable in class test.dataharness.DataOne: **arffInstance1** - Static variable in class test.dataharness.DataTwo: **arffInstance1** - Static variable in class test.mulan.MulanArffTest: **arffInstance1b** - Static variable in class test.dataharness.DataOne: **arffInstance1b** - Static variable in class test.dataharness.DataTwo: **arffInstance2** - Static variable in class test.dataharness.DataOne: **arffInstance2** - Static variable in class test.dataharness.DataTwo: **arffInstance2** - Static variable in class test.mulan.MulanArffTest: **arffInstance4** - Static variable in class test.dataharness.DataOne: **arffInstance4** - Static variable in class test.dataharness.DataTwo: **arffInstance4** - Static variable in class test.mulan.MulanArffTest: **arffInstanceAttributeVoid** - Static variable in class test.mulan.MulanArffTest: **arffInstanceAttVoid** - Static variable in class test.dataharness.DataOne: **arffInstanceAttVoid** - Static variable in class test.dataharness.DataTwo: **arffInstanceClassAttributeVoid** - Static variable in class test.mulan.MulanArffTest: **arffInstanceClassVoid** - Static variable in class test.dataharness.DataOne: **arffInstanceClassVoid** - Static variable in class test.dataharness.DataTwo: **arffInstanceClassVoid** - Static variable in class test.mulan.MulanArffTest: **ArffProperties** - Class in uk.ac.ed.inf.enzml.weka: Contains the properties for a Weka ARFF file It contains: 1. **ArffProperties(String)** - Constructor for class uk.ac.ed.inf.enzml.weka.ArffProperties: Constructor loads the properties from file **ArffPropsFilesTest** - Class in test.dataharness: Test the ARFF properties files used in the test harness and their content. **ArffPropsFilesTest()** - Constructor for class test.dataharness.ArffPropsFilesTest: **ArffPropsOneTest** - Class in test.dataharness: Get the arff data properties from file **ArffPropsOneTest()** - Constructor for class test.dataharness.ArffPropsOneTest: **ArffPropsQueriesOneTest** - Class in test.dataharness: Class **ArffPropsQueriesOneTest()** - Constructor for class test.dataharness.ArffPropsQueriesOneTest: **ArffPropsQueriesTest** - Class in test.dataharness: Checks the results of the queries contained in the arff properties files **ArffPropsQueriesTest()** - Constructor for class test.dataharness.ArffPropsQueriesTest: **ArffPropsQueriesTwoTest** - Class in test.dataharness: Class **ArffPropsQueriesTwoTest()** - Constructor for class test.dataharness.ArffPropsQueriesTwoTest: **ArffPropsTable** - Class in uk.ac.ed.inf.enzml.weka: Class to keep the name and column types for the database table to store the ARFF file parameters (the data files for Weka or Mulan machine learning) **ArffPropsTable()** - Constructor for class uk.ac.ed.inf.enzml.weka.ArffPropsTable: **ArffPropsTableManager** - Class in uk.ac.ed.inf.enzml.weka: Writes into a database table the pointers needed to build an arff file (the name of the arff file, the query to select instances, attributes, class values etc) TODO integrate arff recording to database with mulan experiment recording **ArffPropsTableManager(Arff)** - Constructor for class uk.ac.ed.inf.enzml.weka.ArffPropsTableManager: Constructor 1: initialises the table manager to point to the arff table. **ArffPropsTableManager(DbManager)** - Constructor for class uk.ac.ed.inf.enzml.weka.ArffPropsTableManager: Constructor 2: initialises the table manager to point to the arff table. **ArffPropsTableManagerTest** - Class in test.weka: Class **ArffPropsTableManagerTest()** - Constructor for class test.weka.ArffPropsTableManagerTest: **ArffPropsTableReader** - Class in uk.ac.ed.inf.enzml.weka: Reads properties from the table where the arff records are collected. **ArffPropsTableReader(DbManager)** - Constructor for class uk.ac.ed.inf.enzml.weka.ArffPropsTableReader: **ArffPropsTableReaderTest** - Class in test.weka: Class **ArffPropsTableReaderTest()** - Constructor for class test.weka.ArffPropsTableReaderTest: **ArffPropsTableTest** - Class in test.weka: Class **ArffPropsTableTest()** - Constructor for class test.weka.ArffPropsTableTest: **ArffPropsTwoTest** - Class in test.dataharness: Class **ArffPropsTwoTest()** - Constructor for class test.dataharness.ArffPropsTwoTest: **ArffTest** - Class in test.weka: Class **ArffTest()** - Constructor for class test.weka.ArffTest: **ATT1** - Static variable in class test.dataharness.DataOne: **ATT1\_TO\_STRING** - Static variable in class test.dataharness.DataOne: **ATT1\_TO\_STRING** - Static variable in class test.mulan.MulanArffTest: **ATT2** - Static variable in class test.dataharness.DataOne: **ATT2\_TO\_STRING** - Static variable in class test.dataharness.DataOne: **ATT2\_TO\_STRING** - Static variable in class test.mulan.MulanArffTest: **ATT3** - Static variable in class test.dataharness.DataOne: **ATT3\_TO\_STRING** - Static variable in class test.dataharness.DataOne: **ATT3\_TO\_STRING** - Static variable in class test.mulan.MulanArffTest: **ATT4** - Static variable in class test.dataharness.DataOne: **ATT4\_TO\_STRING** - Static variable in class test.dataharness.DataOne: **ATT4\_TO\_STRING** - Static variable in class test.mulan.MulanArffTest: **ATT5** - Static variable in class test.dataharness.DataOne: **ATT5\_TO\_STRING** - Static variable in class test.dataharness.DataOne: **ATT5\_TO\_STRING** - Static variable in class test.mulan.MulanArffTest: **ATT\_EC\_1111\_TO\_STRING** - Static variable in class test.mulan.MulanArffTest: **ATT\_EC\_111\_TO\_STRING** - Static variable in class test.mulan.MulanArffTest: **ATT\_EC\_11\_TO\_STRING** - Static variable in class test.mulan.MulanArffTest: **ATT\_EC\_1\_TO\_STRING** - Static variable in class test.mulan.MulanArffTest: **ATT\_EC\_2222\_TO\_STRING** - Static variable in class test.mulan.MulanArffTest: **ATT\_EC\_222\_TO\_STRING** - Static variable in class test.mulan.MulanArffTest: **ATT\_EC\_22\_TO\_STRING** - Static variable in class test.mulan.MulanArffTest: **ATT\_EC\_2\_TO\_STRING** - Static variable in class test.mulan.MulanArffTest: **ATTRIBUTE\_FIELD** - Static variable in class test.dataharness.CreateDataTable: **ATTRIBUTE\_ID** - Static variable in class uk.ac.ed.inf.enzml.mulan.predict.MulanPredict: **ATTRIBUTE\_NAME** - Static variable in class uk.ac.ed.inf.enzml.mulan.predict.MulanPredict: **AttributeFactory** - Class in uk.ac.ed.inf.enzml.weka: The attribute factory produces binary and nominal Weka attributes. **AttributeFactory(DataSetGenerator)** - Constructor for class uk.ac.ed.inf.enzml.weka.AttributeFactory: Takes the attribute names from the dataset generator object. **AttributeFactoryTest** - Class in test.weka: Class **AttributeFactoryTest()** - Constructor for class test.weka.AttributeFactoryTest: **attributeIsValid(Attribute)** - Static method in class uk.ac.ed.inf.enzml.weka.AttributeFactory: Check if an attribute is valid (not null, non-empty name) **attributesColumn()** - Static method in class test.dataharness.DataOne: **AttributesFilter** - Class in uk.ac.ed.inf.enzml.mulan.attributesfilter: This class filters the attributes of a dataset (the test set) to make it compatible with another dataset (the train set). **AttributesFilter(int, int, String, String)** - Constructor for class uk.ac.ed.inf.enzml.mulan.attributesfilter.AttributesFilter: **AttributesFilteredArff** - Class in uk.ac.ed.inf.enzml.mulan.attributesfilter: **AttributesFilteredArff(String, String, TreeSet<String>, AttributesFilter, int)** - Constructor for class uk.ac.ed.inf.enzml.mulan.attributesfilter.AttributesFilteredArff: **AttributesFilteredArffTest** - Class in test.mulan.attributesfilter: **AttributesFilteredArffTest()** - Constructor for class test.mulan.attributesfilter.AttributesFilteredArffTest: **AttributesFilteredAttributeFactory** - Class in uk.ac.ed.inf.enzml.mulan.attributesfilter: Takes the list of attributes from AttributesFilteredDataSetGenerator, instead of taking it from the instances-attributes map. **AttributesFilteredAttributeFactory(AttributesFilteredDataSetGenerator)** - Constructor for class uk.ac.ed.inf.enzml.mulan.attributesfilter.AttributesFilteredAttributeFactory: **AttributesFilteredDataSetGenerator** - Class in uk.ac.ed.inf.enzml.mulan.attributesfilter: Get passed a list of attributes, instead of taking it from instances-attributes map. **AttributesFilteredDataSetGenerator(AttributesFilteredDataSetManager, OneToManyMap<String, String>, OneToManyMap<String, String>, TreeSet<String>)** - Constructor for class uk.ac.ed.inf.enzml.mulan.attributesfilter.AttributesFilteredDataSetGenerator: **AttributesFilteredDataSetGeneratorTest** - Class in test.mulan.attributesfilter: **AttributesFilteredDataSetGeneratorTest()** - Constructor for class test.mulan.attributesfilter.AttributesFilteredDataSetGeneratorTest: **AttributesFilteredDataSetManager** - Class in uk.ac.ed.inf.enzml.mulan.attributesfilter: Get passed a list of attributes, instead of taking it from instances-attributes map. **AttributesFilteredDataSetManager(AttributesFilteredArff)** - Constructor for class uk.ac.ed.inf.enzml.mulan.attributesfilter.AttributesFilteredDataSetManager: **AttributesFilterTest** - Class in test.mulan.attributesfilter: **AttributesFilterTest()** - Constructor for class test.mulan.attributesfilter.AttributesFilterTest: **attributesSection(Instances)** - Method in class uk.ac.ed.inf.enzml.weka.DataSetWriter: **attributesValues()** - Static method in class test.dataharness.DataOne: Results of a select distinct instance, attribute query **AttributeUtils** - Class in uk.ac.ed.inf.enzml.mulan: **AttributeUtils()** - Constructor for class uk.ac.ed.inf.enzml.mulan.AttributeUtils: **AUTHOR** - Static variable in class uk.ac.ed.inf.enzml.ProjectParameters

---


|  |  |  |  |  |  |  |  |  |  |  |
| --- | --- | --- | --- | --- | --- | --- | --- | --- | --- | --- |
| |  |  |  |  |  |  |  |  | | --- | --- | --- | --- | --- | --- | --- | --- | | **Overview** | Package | Class | Use | **Tree** | **Deprecated** | **Index** | **Help** | | |  |
| PREV LETTER   **NEXT LETTER** | **FRAMES**    **NO FRAMES**     **All Classes** |


A B C D E F G I K L M N P R S T U V W X 

---
