# Supplementary material for: EnzML: multi-label prediction of enzyme classes using InterPro signatures
Source: BMC Bioinformatics. 2012 Apr 25;13:61. doi: 10.1186/1471-2105-13-61 (PMC3483700; doi:10.1186/1471-2105-13-61)
Supplement: Addtional file 5 — The Java code to format the data files, evaluate and predict. The file enzml_java_code.tar.gz contains the Java code used to format database data to ARFF and XML formats, to execute cross and train-test (jackknife) evaluations and to record evaluation results to database. More information is included in the readme.txt file and the Javadoc files. The code can be used with a MySQL database. To use a different database software, other JDBC drivers might be required. [file 1471-2105-13-61-S5.gz › java_code/enzml2011/doc/index-files/index-14.html]

R-Index


---


|  |  |  |  |  |  |  |  |  |  |  |
| --- | --- | --- | --- | --- | --- | --- | --- | --- | --- | --- |
| |  |  |  |  |  |  |  |  | | --- | --- | --- | --- | --- | --- | --- | --- | | **Overview** | Package | Class | Use | **Tree** | **Deprecated** | **Index** | **Help** | | |  |
| **PREV LETTER**   **NEXT LETTER** | **FRAMES**    **NO FRAMES**     **All Classes** |


A B C D E F G I K L M N P R S T U V W X 

---


## **R**

**refreshConnection(DbManager)** - Method in class uk.ac.ed.inf.enzml.mulan.learn.ResultsSaver: **ResultCheckerTest** - Class in test.mulan.learn: Class **ResultCheckerTest(TableRow)** - Constructor for class test.mulan.learn.ResultCheckerTest: **RESULTS\_DIRECTORY** - Static variable in class uk.ac.ed.inf.enzml.ProjectParameters: **ResultsFormatter** - Class in uk.ac.ed.inf.enzml.mulan.learn: Class **ResultsFormatter()** - Constructor for class uk.ac.ed.inf.enzml.mulan.learn.ResultsFormatter: **ResultsFormatterTest** - Class in test.mulan.learn: Class **ResultsFormatterTest()** - Constructor for class test.mulan.learn.ResultsFormatterTest: **ResultsSaver** - Class in uk.ac.ed.inf.enzml.mulan.learn: Outputs cross-evaluation results to screen, file and database. **ResultsSaver(DbManager, String)** - Constructor for class uk.ac.ed.inf.enzml.mulan.learn.ResultsSaver: **ResultsSaverTest** - Class in test.mulan.learn: Class **ResultsSaverTest()** - Constructor for class test.mulan.learn.ResultsSaverTest: **resultsString(TableRow)** - Method in class uk.ac.ed.inf.enzml.mulan.learn.ResultsSaver: **run(int, int, String, String)** - Static method in class uk.ac.ed.inf.enzml.mulan.attributesfilter.AttributesFilter: Filter the old test arff by the train arff given and regenerate the old train set using all train+test classes. **RUN** - Static variable in class uk.ac.ed.inf.enzml.mulan.learn.ExperimentTable: the evaluation run number (eg: between 0 and 9 for a 10 fold cross evaluation) **run(int[], String)** - Static method in class uk.ac.ed.inf.enzml.mulan.learn.MulanCrossExperimenter: Run a cross-evaluation of several ARFF files (using one or several algorithms, as needed) **run(int, MulanLearner, String, String)** - Static method in class uk.ac.ed.inf.enzml.mulan.learn.MulanSerializer: **run()** - Static method in class uk.ac.ed.inf.enzml.mulan.learn.traintest.TrainTestExperimenter: **run(String, String, int, int, MulanLearner, String, String)** - Static method in class uk.ac.ed.inf.enzml.mulan.learn.traintest.TrainTestExperimenterSerialized: **run(String, String)** - Static method in class uk.ac.ed.inf.enzml.mulan.MulanArff: Generates the ARFF (and XML) files and returns the identifier of the arff record in the database **run(String[], String)** - Static method in class uk.ac.ed.inf.enzml.mulan.MulanArff: Generates a number of ARFF files (the database the records are written is the same for all ARFF files) **run(int, int, String, String, MulanLearner)** - Static method in class uk.ac.ed.inf.enzml.mulan.predict.MulanPredict: Generates and saves predictions. **run(String, String)** - Static method in class uk.ac.ed.inf.enzml.weka.Arff: **run2\_3()** - Static method in class uk.ac.ed.inf.enzml.mulan.learn.traintest.TrainTestFullRun: Executes 2. **run3()** - Static method in class uk.ac.ed.inf.enzml.mulan.learn.traintest.TrainTestFullRun: When files and trained model are already available, it executes exclusively the train-test evaluation

---


|  |  |  |  |  |  |  |  |  |  |  |
| --- | --- | --- | --- | --- | --- | --- | --- | --- | --- | --- |
| |  |  |  |  |  |  |  |  | | --- | --- | --- | --- | --- | --- | --- | --- | | **Overview** | Package | Class | Use | **Tree** | **Deprecated** | **Index** | **Help** | | |  |
| **PREV LETTER**   **NEXT LETTER** | **FRAMES**    **NO FRAMES**     **All Classes** |


A B C D E F G I K L M N P R S T U V W X 

---
