# Supplementary material for: EnzML: multi-label prediction of enzyme classes using InterPro signatures
Source: BMC Bioinformatics. 2012 Apr 25;13:61. doi: 10.1186/1471-2105-13-61 (PMC3483700; doi:10.1186/1471-2105-13-61)
Supplement: Addtional file 5 — The Java code to format the data files, evaluate and predict. The file enzml_java_code.tar.gz contains the Java code used to format database data to ARFF and XML formats, to execute cross and train-test (jackknife) evaluations and to record evaluation results to database. More information is included in the readme.txt file and the Javadoc files. The code can be used with a MySQL database. To use a different database software, other JDBC drivers might be required. [file 1471-2105-13-61-S5.gz › java_code/enzml2011/doc/index-files/index-11.html]

M-Index


---


|  |  |  |  |  |  |  |  |  |  |  |
| --- | --- | --- | --- | --- | --- | --- | --- | --- | --- | --- |
| |  |  |  |  |  |  |  |  | | --- | --- | --- | --- | --- | --- | --- | --- | | **Overview** | Package | Class | Use | **Tree** | **Deprecated** | **Index** | **Help** | | |  |
| **PREV LETTER**   **NEXT LETTER** | **FRAMES**    **NO FRAMES**     **All Classes** |


A B C D E F G I K L M N P R S T U V W X 

---


## **M**

**m\_arff** - Static variable in class test.weka.DataSetManagerTest: **m\_memoryAbundant** - Variable in class uk.ac.ed.inf.enzml.mulan.MulanArff: if plenty of memory available, also load each instance in weka instances, otherwise only write them to file one by one **MACHINE\_LEARNING\_LOCAL\_DB\_PROPS** - Static variable in class uk.ac.ed.inf.enzml.ProjectParameters: **MACHINE\_LEARNING\_PROPS** - Static variable in class uk.ac.ed.inf.enzml.ProjectParameters: **MACHINE\_LEARNING\_REMOTE\_DB\_PROPS** - Static variable in class uk.ac.ed.inf.enzml.ProjectParameters: **main(String[])** - Static method in class test.AllArffTests: **main(String[])** - Static method in class test.AllMulanArffTests: **main(String[])** - Static method in class test.AllMulanLearningTests: **main(String[])** - Static method in class test.AllMulanPredictionTests: **main(String[])** - Static method in class test.AllMulanTests: **main(String[])** - Static method in class test.AllPreliminaryTests: **main(String[])** - Static method in class test.AllTests: **main(String[])** - Static method in class test.AllTrainTestsTests: **main(String[])** - Static method in class test.AllUtilsTests: **main(String[])** - Static method in class test.dataharness.AllDataTests: **main(String[])** - Static method in class test.mulan.predict.MulanEmitPredictions: **main(String[])** - Static method in class uk.ac.ed.inf.enzml.mulan.attributesfilter.AttributesFilter: **main(String[])** - Static method in class uk.ac.ed.inf.enzml.mulan.learn.ExperimentTable: **main(String[])** - Static method in class uk.ac.ed.inf.enzml.mulan.learn.MulanCrossExperimenter: **main(String[])** - Static method in class uk.ac.ed.inf.enzml.mulan.learn.MulanSerializer: **main(String[])** - Static method in class uk.ac.ed.inf.enzml.mulan.learn.traintest.MulanTrain: **main(String[])** - Static method in class uk.ac.ed.inf.enzml.mulan.learn.traintest.TrainTestExperimenter: **main(String[])** - Static method in class uk.ac.ed.inf.enzml.mulan.learn.traintest.TrainTestExperimenterSerialized: **main(String[])** - Static method in class uk.ac.ed.inf.enzml.mulan.learn.traintest.TrainTestFullRun: **main(String[])** - Static method in class uk.ac.ed.inf.enzml.mulan.MulanArff: **main(String[])** - Static method in class uk.ac.ed.inf.enzml.mulan.predict.MulanPredict: **main(String[])** - Static method in class uk.ac.ed.inf.enzml.mulan.predict.MulanPredictWithTrainedModel: **main(String[])** - Static method in class uk.ac.ed.inf.enzml.weka.Arff: **main(String[])** - Static method in class uk.ac.ed.inf.enzml.weka.ArffPropsTableManager: Main for recreating table **memoryIsAbundant()** - Method in class uk.ac.ed.inf.enzml.weka.Arff: **MODELS\_PATH** - Static variable in class uk.ac.ed.inf.enzml.ProjectParameters: **MULAN\_DATABASE\_NAME** - Static variable in class uk.ac.ed.inf.enzml.mulan.database.MulanDbCreator: **MulanArff** - Class in uk.ac.ed.inf.enzml.mulan: Generates an Attribute Relation Format File (ARFF) in Mulan format (multiple class labels per instance). **MulanArff(String, String)** - Constructor for class uk.ac.ed.inf.enzml.mulan.MulanArff: **MulanArff(String, String, boolean)** - Constructor for class uk.ac.ed.inf.enzml.mulan.MulanArff: **MulanArffProperties** - Class in uk.ac.ed.inf.enzml.mulan: Class **MulanArffProperties(String)** - Constructor for class uk.ac.ed.inf.enzml.mulan.MulanArffProperties: **MulanArffRecord** - Class in uk.ac.ed.inf.enzml.mulan: Checks that an ARFF record read from the database contains valid parameters to read an ARFF file. **MulanArffRecord(int, String)** - Constructor for class uk.ac.ed.inf.enzml.mulan.MulanArffRecord: **MulanArffRecordTest** - Class in test.mulan: Class **MulanArffRecordTest()** - Constructor for class test.mulan.MulanArffRecordTest: **MulanArffTest** - Class in test.mulan: Class **MulanArffTest()** - Constructor for class test.mulan.MulanArffTest: **MulanAttributeFactory** - Class in uk.ac.ed.inf.enzml.mulan: In the Mulan ARFF, the class attribute is not generated. **MulanAttributeFactory(MulanDataSetGenerator)** - Constructor for class uk.ac.ed.inf.enzml.mulan.MulanAttributeFactory: **MulanCrossEvaluator** - Class in uk.ac.ed.inf.enzml.mulan.learn: Class created to execute cross-evaluations and extract individual evaluation results (not possible from the Mulan class Evaluator, because only averaged results are returned). **MulanCrossEvaluator(MulanCrossExperimenter, boolean)** - Constructor for class uk.ac.ed.inf.enzml.mulan.learn.MulanCrossEvaluator: **MulanCrossExperimenter** - Class in uk.ac.ed.inf.enzml.mulan.learn: Using Mulan (multi-label machine learning), this class initialises and executes either Train-Test evaluation (if two different arff records are given) (calling `MulanTrainTestEvaluator`) or cross-evaluations (calling `MulanCrossEvaluator`) if only one arff record is given. **MulanCrossExperimenter(String, String, int, MulanLearner, String)** - Constructor for class uk.ac.ed.inf.enzml.mulan.learn.MulanCrossExperimenter: public MulanCrossExperimenter(String resultsDbPropsFile, String arffRecordDbProps, MulanArffRecord trainArffRecord, MulanLearner learner, String resultsFolder) { m\_trainArffRecord = trainArffRecord; this.initialise(resultsDbPropsFile, arffRecordDbProps, learner, resultsFolder); } **MulanDataSetDbLoader** - Class in uk.ac.ed.inf.enzml.mulan: Loads data from database to a Mulan data factory **MulanDataSetDbLoader(MulanDataSetManager)** - Constructor for class uk.ac.ed.inf.enzml.mulan.MulanDataSetDbLoader: **MulanDataSetDbLoaderTest** - Class in test.mulan: Class **MulanDataSetDbLoaderTest()** - Constructor for class test.mulan.MulanDataSetDbLoaderTest: **MulanDataSetGenerator** - Class in uk.ac.ed.inf.enzml.mulan: Class **MulanDataSetGenerator(MulanDataSetManager, OneToManyMap<String, String>, OneToManyMap<String, String>)** - Constructor for class uk.ac.ed.inf.enzml.mulan.MulanDataSetGenerator: **MulanDataSetGeneratorTest** - Class in test.mulan: Class **MulanDataSetGeneratorTest()** - Constructor for class test.mulan.MulanDataSetGeneratorTest: **MulanDataSetManager** - Class in uk.ac.ed.inf.enzml.mulan: This class generates a data set (instances, attributes, classes) in Mulan style (for multi-label machine learning). **MulanDataSetManager(MulanArff)** - Constructor for class uk.ac.ed.inf.enzml.mulan.MulanDataSetManager: **MulanDataSetManagerTest** - Class in test.mulan: Class **MulanDataSetManagerTest()** - Constructor for class test.mulan.MulanDataSetManagerTest: **MulanDataSetWriterTest** - Class in test.mulan: Class **MulanDataSetWriterTest()** - Constructor for class test.mulan.MulanDataSetWriterTest: **MulanDbCreator** - Class in uk.ac.ed.inf.enzml.mulan.database: Creates the table to store Mulan machine learning results. **MulanDbCreator(DbManager)** - Constructor for class uk.ac.ed.inf.enzml.mulan.database.MulanDbCreator: **MulanDbCreatorTest** - Class in test.mulan.learn.database: Class **MulanDbCreatorTest()** - Constructor for class test.mulan.learn.database.MulanDbCreatorTest: **MulanDbManager** - Class in uk.ac.ed.inf.enzml.mulan.database: The database manager controls the writing to database. **MulanDbManager(String)** - Constructor for class uk.ac.ed.inf.enzml.mulan.database.MulanDbManager: The `DbManager` constructor creates `DbCreator`, `DbWriter` and `DbReader` objects and writes the necessary tables to database. **MulanDbManagerTest** - Class in test.mulan.learn.database: Class **MulanDbManagerTest()** - Constructor for class test.mulan.learn.database.MulanDbManagerTest: **MulanDbReader** - Class in uk.ac.ed.inf.enzml.mulan.database: Reads ARFF file path from database **MulanDbReader(DbManager)** - Constructor for class uk.ac.ed.inf.enzml.mulan.database.MulanDbReader: **MulanDbReaderTest** - Class in test.mulan.learn.database: Class **MulanDbReaderTest()** - Constructor for class test.mulan.learn.database.MulanDbReaderTest: **MulanDbWriter** - Class in uk.ac.ed.inf.enzml.mulan.database: Class **MulanDbWriter(DbManager)** - Constructor for class uk.ac.ed.inf.enzml.mulan.database.MulanDbWriter: **MulanDbWriterTest** - Class in test.mulan.learn.database: Class **MulanDbWriterTest()** - Constructor for class test.mulan.learn.database.MulanDbWriterTest: **MulanEmitPredictions** - Class in test.mulan.predict: Class **MulanEmitPredictions()** - Constructor for class test.mulan.predict.MulanEmitPredictions: **MulanInstancesFiller** - Class in uk.ac.ed.inf.enzml.mulan: Class **MulanInstancesFiller(MulanDataSetGenerator)** - Constructor for class uk.ac.ed.inf.enzml.mulan.MulanInstancesFiller: **MulanInstancesFillerTest** - Class in test.mulan: Class **MulanInstancesFillerTest()** - Constructor for class test.mulan.MulanInstancesFillerTest: **MulanLearner** - Class in uk.ac.ed.inf.enzml.mulan.learn: Containing a Mulan learner and its name, that is: the name of the MultiLabelLearnerBase plus the name of the algorithm within **MulanLearner(String, String, MultiLabelLearnerBase)** - Constructor for class uk.ac.ed.inf.enzml.mulan.learn.MulanLearner: **MulanLearners** - Class in uk.ac.ed.inf.enzml.mulan.learn: Class **MulanLearners()** - Constructor for class uk.ac.ed.inf.enzml.mulan.learn.MulanLearners: **MulanPredict** - Class in uk.ac.ed.inf.enzml.mulan.predict: First trains on a dataset (training set) and then emits predictions for a (compatible unlabelled) dataset. **MulanPredict(int, int, String, String, MulanLearner)** - Constructor for class uk.ac.ed.inf.enzml.mulan.predict.MulanPredict: **MulanPredictTest** - Class in test.mulan.predict: Train arff: inst1, inst2 etc. **MulanPredictTest()** - Constructor for class test.mulan.predict.MulanPredictTest: **MulanPredictWithTrainedModel** - Class in uk.ac.ed.inf.enzml.mulan.predict: Predicts an unlabeled data set using a previously trained and serialized classifier. **MulanPredictWithTrainedModel(int, int, String, String, String, MulanLearner)** - Constructor for class uk.ac.ed.inf.enzml.mulan.predict.MulanPredictWithTrainedModel: **MulanSerializer** - Class in uk.ac.ed.inf.enzml.mulan.learn: Takes an arff file and an algorithm. **MulanSerializer(MulanArffRecord, MulanLearner, String)** - Constructor for class uk.ac.ed.inf.enzml.mulan.learn.MulanSerializer: **MulanTrain** - Class in uk.ac.ed.inf.enzml.mulan.learn.traintest: Give arff and xml file path, weka algorithm, cross evaluation fold and it emits learning results **MulanTrain()** - Constructor for class uk.ac.ed.inf.enzml.mulan.learn.traintest.MulanTrain

---


|  |  |  |  |  |  |  |  |  |  |  |
| --- | --- | --- | --- | --- | --- | --- | --- | --- | --- | --- |
| |  |  |  |  |  |  |  |  | | --- | --- | --- | --- | --- | --- | --- | --- | | **Overview** | Package | Class | Use | **Tree** | **Deprecated** | **Index** | **Help** | | |  |
| **PREV LETTER**   **NEXT LETTER** | **FRAMES**    **NO FRAMES**     **All Classes** |


A B C D E F G I K L M N P R S T U V W X 

---
