# Supplementary material for: EnzML: multi-label prediction of enzyme classes using InterPro signatures
Source: BMC Bioinformatics. 2012 Apr 25;13:61. doi: 10.1186/1471-2105-13-61 (PMC3483700; doi:10.1186/1471-2105-13-61)
Supplement: Addtional file 5 — The Java code to format the data files, evaluate and predict. The file enzml_java_code.tar.gz contains the Java code used to format database data to ARFF and XML formats, to execute cross and train-test (jackknife) evaluations and to record evaluation results to database. More information is included in the readme.txt file and the Javadoc files. The code can be used with a MySQL database. To use a different database software, other JDBC drivers might be required. [file 1471-2105-13-61-S5.gz › java_code/enzml2011/doc/index-files/index-7.html]

 
 
 
 
 
 
G-Index
 

 

 

 
function windowTitle()
{
    if (location.href.indexOf('is-external=true') == -1) {
        parent.document.title="G-Index";
    }
}
 
 
 

 

 
 


 
   
  
 
 
 
   
 
   
          Overview     
        Package   
        Class   
        Use   
          Tree     
          Deprecated     
       Index    
          Help     
   
 
 
  
 
 
 

 
  
   PREV LETTER   
   NEXT LETTER    
  
    FRAMES     
   NO FRAMES     
  
    All Classes  ');
  }
  //-->
 
 
    All Classes  
 


  
 
 
  
 

 A   B   C   D   E   F   G   I   K   L   M   N   P   R   S   T   U   V   W   X   
    
 G  
 
   generateAndWriteArffFile()   - 
Method in class uk.ac.ed.inf.enzml.weka. Arff 
 The core of the method to write Weka style ARFF files: save properties to
 experiment table, load data from data database and save arff to file
   generateArff(String)   - 
Method in class test.mulan.attributesfilter. ArffGeneratorForFilterTests 
 Generate an arff file and return the arff record id
   generateArff()   - 
Method in class uk.ac.ed.inf.enzml.weka. Arff 
  
   generateBinaryAttribute(String)   - 
Method in class uk.ac.ed.inf.enzml.mulan. MulanAttributeFactory 
  
   generateBinaryAttribute(String)   - 
Method in class uk.ac.ed.inf.enzml.weka. AttributeFactory 
 Generates a binary attribute
   generateInstancesNames()   - 
Method in class uk.ac.ed.inf.enzml.mulan.predict. MulanPredict 
  
   generateNominalAttribute(String, Vector<String>)   - 
Method in class uk.ac.ed.inf.enzml.weka. AttributeFactory 
 Generate a nominal attribute (with a list of possible values).
   generateNominalAttributeWithDummy(String, Set<String>)   - 
Method in class uk.ac.ed.inf.enzml.weka. AttributeFactory 
 Generate a nominal attribute (with a list of possible values).
   generateTestArff()   - 
Method in class test.mulan.attributesfilter. ArffGeneratorForFilterTests 
 Generate the old test arff and return the arff record id
   generateTestArffAndReturnId(String)   - 
Static method in class test.mulan. MulanArffTest 
 Create test arff file
   generateTrainArff()   - 
Method in class test.mulan.attributesfilter. ArffGeneratorForFilterTests 
 Generate the old train arff and return the arff record id
   getArff()   - 
Static method in class test.weka. ArffTest 
  
   getArffAndResultsProperties()   - 
Method in class uk.ac.ed.inf.enzml.mulan.attributesfilter. AttributesFilter 
  
   getArffDbConnPath()   - 
Method in class uk.ac.ed.inf.enzml.mulan. MulanArffRecord 
  
   getArffFileName()   - 
Method in class uk.ac.ed.inf.enzml.mulan. MulanArffRecord 
  
   getArffFilePath()   - 
Method in class uk.ac.ed.inf.enzml.mulan.learn. MulanCrossEvaluator 
  
   getArffFilePath()   - 
Method in class uk.ac.ed.inf.enzml.weka. ArffProperties 
 Get the path to the ARFF file
   getArffFilePathTrainFile()   - 
Static method in class test.mulan. MulanDataSetManagerTest 
  
   getArffGenerator()   - 
Method in class uk.ac.ed.inf.enzml.mulan.attributesfilter. AttributesFilteredDataSetManager 
  
   getArffGenerator()   - 
Method in class uk.ac.ed.inf.enzml.mulan. MulanDataSetManager 
  
   getArffGenerator()   - 
Method in class uk.ac.ed.inf.enzml.weka. ArffPropsTableManager 
  
   getArffGenerator()   - 
Method in class uk.ac.ed.inf.enzml.weka. DataSetManager 
  
   getArffId()   - 
Method in class uk.ac.ed.inf.enzml.mulan. MulanArffRecord 
  
   getArffPath(int)   - 
Method in class uk.ac.ed.inf.enzml.mulan.database. MulanDbReader 
 Get an ARFF path from database row
   getArffPath()   - 
Method in class uk.ac.ed.inf.enzml.weka. Arff 
  
   getArffPathFrobDb(int)   - 
Method in class uk.ac.ed.inf.enzml.weka. ArffPropsTableReader 
  
   getArffProperties()   - 
Method in class uk.ac.ed.inf.enzml.weka. DataSetChecker 
  
   getArffProperties()   - 
Method in class uk.ac.ed.inf.enzml.weka. DataSetManager 
  
   getArffProperties1()   - 
Static method in class test.dataharness. ArffPropsOneTest 
  
   getArffProperties2()   - 
Static method in class test.dataharness. ArffPropsTwoTest 
  
   getArffPropsPathFrobDb(int)   - 
Method in class uk.ac.ed.inf.enzml.weka. ArffPropsTableReader 
  
   getArffRecord()   - 
Method in class uk.ac.ed.inf.enzml.mulan.learn. MulanSerializer 
  
   getArffRecordId()   - 
Method in class uk.ac.ed.inf.enzml.weka. Arff 
  
   getArffRecordId()   - 
Method in class uk.ac.ed.inf.enzml.weka. ArffPropsTableManager 
  
   getArffRecordsDbManager()   - 
Method in class uk.ac.ed.inf.enzml.mulan.attributesfilter. AttributesFilter 
  
   getArffTableReader()   - 
Method in class uk.ac.ed.inf.enzml.mulan.attributesfilter. AttributesFilter 
  
   getArffWithNoXmlProperty()   - 
Static method in class test.weka. ArffTest 
  
   getAttributeAbsentOption()   - 
Method in class uk.ac.ed.inf.enzml.mulan. MulanAttributeFactory 
  
   getAttributeAbsentOption()   - 
Method in class uk.ac.ed.inf.enzml.weka. AttributeFactory 
  
   getAttributeByName(String)   - 
Method in class uk.ac.ed.inf.enzml.weka. AttributeFactory 
  
   getAttributePresentOption()   - 
Method in class uk.ac.ed.inf.enzml.mulan. MulanAttributeFactory 
  
   getAttributePresentOption()   - 
Method in class uk.ac.ed.inf.enzml.weka. AttributeFactory 
  
   getAttributes()   - 
Method in class uk.ac.ed.inf.enzml.weka. AttributeFactory 
  
   getAttributes()   - 
Method in class uk.ac.ed.inf.enzml.weka. DataSetGenerator 
  
   getAttributesFactory()   - 
Method in class uk.ac.ed.inf.enzml.weka. DataSetGenerator 
  
   getAttributesFilter()   - 
Static method in class test.mulan.attributesfilter. AttributesFilterTest 
  
   getAttributesNames()   - 
Method in class uk.ac.ed.inf.enzml.mulan.attributesfilter. AttributesFilteredArff 
  
   getAttributesNames()   - 
Method in class uk.ac.ed.inf.enzml.weka. AttributeFactory 
  
   getBinaryAttributeValuesSet()   - 
Method in class uk.ac.ed.inf.enzml.mulan. MulanAttributeFactory 
  
   getBinaryAttributeValuesSet()   - 
Method in class uk.ac.ed.inf.enzml.weka. AttributeFactory 
 Get the possible attribute values for a yes/no attribute.
   getBRk1NN()   - 
Static method in class test.mulan.learn. LearnerTest 
  
   getBRk1NN()   - 
Static method in class uk.ac.ed.inf.enzml.mulan.learn. MulanLearner 
  
   getBRkNN(int)   - 
Static method in class test.mulan.learn. LearnerTest 
  
   getBRkNN(int)   - 
Static method in class uk.ac.ed.inf.enzml.mulan.learn. MulanLearner 
  
   getClassAttribute()   - 
Method in class uk.ac.ed.inf.enzml.weka. AttributeFactory 
 Get last attribute as class attribute
   getClassName()   - 
Method in class uk.ac.ed.inf.enzml.weka. DataSetGenerator 
  
   getClassName()   - 
Method in class uk.ac.ed.inf.enzml.weka. DataSetManager 
  
   getColumnDefinitions()   - 
Static method in class uk.ac.ed.inf.enzml.mulan.learn. ExperimentTable 
  
   getColumnDefinitions()   - 
Static method in class uk.ac.ed.inf.enzml.weka. ArffPropsTable 
 Creates the table for the ARFF file records
   getCorrectAttributes()   - 
Static method in class test.mulan.attributesfilter. AttributesFilteredArffTest 
  
   getCorrectClasses()   - 
Static method in class test.mulan.attributesfilter. AttributesFilteredArffTest 
  
   getCurrentResultsRow()   - 
Method in class uk.ac.ed.inf.enzml.mulan.learn. ResultsSaver 
  
   getDataArffProperties()   - 
Method in class uk.ac.ed.inf.enzml.weka. Arff 
  
   getDataDbManager()   - 
Method in class uk.ac.ed.inf.enzml.mulan.attributesfilter. AttributesFilter 
  
   getDataDbManager()   - 
Method in class uk.ac.ed.inf.enzml.weka. Arff 
  
   getDataDbPath()   - 
Method in class uk.ac.ed.inf.enzml.mulan.attributesfilter. AttributesFilter 
  
   getDataLoader()   - 
Method in class uk.ac.ed.inf.enzml.mulan. MulanDataSetManager 
  
   getDataLoader()   - 
Method in class uk.ac.ed.inf.enzml.weka. DataSetManager 
  
   getDataProperties()   - 
Method in class uk.ac.ed.inf.enzml.weka. DataSetDbLoader 
  
   getDataPropsFilePath()   - 
Method in class uk.ac.ed.inf.enzml.weka. Arff 
  
   getDataSetChecker()   - 
Method in class uk.ac.ed.inf.enzml.weka. DataSetManager 
  
   getDataSetCheckerKO()   - 
Static method in class test.weka. DataSetCheckerTest 
  
   getDataSetCheckerOK()   - 
Static method in class test.weka. DataSetCheckerTest 
  
   getDataSetGenerator()   - 
Static method in class test.weka. DataSetGeneratorTest 
  
   getDataSetGenerator()   - 
Method in class uk.ac.ed.inf.enzml.mulan.attributesfilter. AttributesFilteredAttributeFactory 
  
   getDataSetGenerator()   - 
Method in class uk.ac.ed.inf.enzml.mulan.attributesfilter. AttributesFilteredDataSetManager 
  
   getDataSetGenerator()   - 
Method in class uk.ac.ed.inf.enzml.mulan. MulanAttributeFactory 
  
   getDataSetGenerator()   - 
Method in class uk.ac.ed.inf.enzml.mulan. MulanDataSetManager 
  
   getDataSetGenerator()   - 
Method in class uk.ac.ed.inf.enzml.mulan. MulanInstancesFiller 
  
   getDataSetGenerator()   - 
Method in class uk.ac.ed.inf.enzml.weka. AttributeFactory 
  
   getDataSetGenerator()   - 
Method in class uk.ac.ed.inf.enzml.weka. DataSetManager 
  
   getDataSetGenerator()   - 
Method in class uk.ac.ed.inf.enzml.weka. InstancesFiller 
 get instances map public TreeMap 
 getInstanceNameObjectMap() { return m_instancesMap; }
   getDataSetGeneratorTrainFile()   - 
Static method in class test.mulan. MulanDataSetGeneratorTest 
 public static MulanDataSetGenerator getDataSetGeneratorArff1() {
                return MulanDataSetManagerTest.getDataSetManagerArff1().getDataSetGenerator();
        }
   getDataSetManager()   - 
Static method in class test.weka. DataSetManagerTest 
  
   getDataSetManager()   - 
Method in class uk.ac.ed.inf.enzml.mulan.attributesfilter. AttributesFilteredArff 
  
   getDataSetManager()   - 
Method in class uk.ac.ed.inf.enzml.mulan. MulanArff 
  
   getDataSetManager()   - 
Method in class uk.ac.ed.inf.enzml.weka. Arff 
  
   getDataSetManager()   - 
Method in class uk.ac.ed.inf.enzml.weka. DataSetDbLoader 
  
   getDataSetManager()   - 
Method in class uk.ac.ed.inf.enzml.weka. DataSetWriter 
  
   getDataSetManagerArff1()   - 
Static method in class test.mulan. MulanDataSetManagerTest 
  
   getDataSetManagerTrainFile()   - 
Static method in class test.mulan. MulanDataSetManagerTest 
  
   getDataSetName()   - 
Method in class uk.ac.ed.inf.enzml.weka. DataSetManager 
  
   getDataSetWithNoXmlProperty()   - 
Static method in class test.weka. DataSetManagerTest 
  
   getDataSetWriter()   - 
Static method in class test.weka. DataSetWriterTest 
  
   getDataSetWriter()   - 
Method in class uk.ac.ed.inf.enzml.weka. DataSetManager 
  
   getDbConn()   - 
Method in class uk.ac.ed.inf.enzml.weka. Arff 
  
   getDbConn()   - 
Method in class uk.ac.ed.inf.enzml.weka. ArffProperties 
  
   getDbCreator()   - 
Static method in class test.mulan.learn.database. MulanDbCreatorTest 
  
   getDbLoader()   - 
Static method in class test.mulan. MulanDataSetDbLoaderTest 
  
   getDbLoader()   - 
Static method in class test.weka. DataSetDbLoaderTest 
  
   getDbManager()   - 
Static method in class test.dataharness. DatabaseTest 
  
   getDbManager()   - 
Static method in class test.mulan.learn.database. MulanDbManagerTest 
  
   getDbReader()   - 
Static method in class test.mulan.learn.database. MulanDbReaderTest 
  
   getDbReader()   - 
Method in class uk.ac.ed.inf.enzml.weka. Arff 
  
   getDbWriter()   - 
Static method in class test.mulan.learn.database. MulanDbManagerTest 
  
   getDirectoryForSerializedModel()   - 
Method in class uk.ac.ed.inf.enzml.mulan.learn. MulanSerializer 
  
   getEndTime()   - 
Method in class uk.ac.ed.inf.enzml.mulan.learn. MulanCrossEvaluator 
  
   getEvaluation()   - 
Static method in class test.mulan.learn. ResultsFormatterTest 
  
   getEvaluationMeasuresRows()   - 
Method in class uk.ac.ed.inf.enzml.mulan.learn. MulanCrossEvaluator 
  
   getEvaluationRows(TrainTestEvaluator)   - 
Static method in class test.mulan.learn.traintest. TrainTestEvaluatorTest 
  
   getExpectedArff()   - 
Static method in class test.weka. DataSetManagerTest 
  
   getExpectedMulanTestFile()   - 
Static method in class test.mulan. MulanArffTest 
  
   getExpectedMulanTrainFile()   - 
Static method in class test.mulan. MulanArffTest 
  
   getExpectedMulanXml2()   - 
Static method in class test.mulan. MulanArffTest 
  
   getExpectedMulanXmlTestFile()   - 
Static method in class test.mulan. MulanArffTest 
  
   getExpectedMulanXmlTrainFile()   - 
Static method in class test.mulan. MulanArffTest 
  
   getExperimentDbManager()   - 
Method in class uk.ac.ed.inf.enzml.mulan.learn. MulanCrossExperimenter 
 public MulanCrossEvaluator getEvaluator() { return m_evaluator; }
   getExperimenter()   - 
Method in class uk.ac.ed.inf.enzml.mulan.learn. MulanCrossEvaluator 
 public Evaluation[] getEvaluations() { return m_evaluations; }
   getExperimenter()   - 
Method in class uk.ac.ed.inf.enzml.mulan.learn.traintest. TrainTestEvaluator 
  
   getExperimentPropsFile()   - 
Method in class uk.ac.ed.inf.enzml.mulan.learn. MulanCrossExperimenter 
  
   getFileForSerializedModel()   - 
Method in class uk.ac.ed.inf.enzml.mulan.learn. MulanSerializer 
  
   getFilePath()   - 
Static method in class test.mulan.learn. ResultsSaverTest 
  
   getFilePath()   - 
Method in class uk.ac.ed.inf.enzml.mulan.learn. ResultsSaver 
 return the file path the results will be saved to
   getFilePath()   - 
Method in class uk.ac.ed.inf.enzml.weka. DataSetWriter 
  
   getFileRole(String)   - 
Static method in class test.mulan.attributesfilter. AttributesFilteredArffTest 
 Returns the role ("train" or "test") of the file for a potential
 train/test evaluation.
   getFilteredArffName(int, int, int)   - 
Static method in class uk.ac.ed.inf.enzml.mulan.attributesfilter. AttributesFilteredArff 
  
   getFilteredArffs(int, int)   - 
Static method in class test.mulan.learn.traintest. EvaluationMetricsTest 
  
   getFilterManager()   - 
Method in class uk.ac.ed.inf.enzml.mulan.attributesfilter. AttributesFilteredArff 
  
   getFirstTestArffFromDb()   - 
Static method in class test.mulan. MulanArffTest 
 public static int getFirstFilteredTestArff() { ArffPropsTableReader
 reader = ArffPropsTableReaderTest.getReader1(); // return the first arff
 having different identifiers as "old test id" // and
 "id the attributes were taken from (train set)"
 
 // return the first arff id containing "test" in its pathname return
 reader.getIdByArffPath("_with_attr_of_"); }
 
 public static int getFirstFilteredTrainArff() { ArffPropsTableReader
 reader = ArffPropsTableReaderTest.getReader1(); // return the first arff
 having different identifiers as "old test id" // and
 "id the attributes were taken from (train set)"
 
 // return the first arff id containing "test" in its pathname return
 reader.getIdByArffPath("_with_attr_of_"); }
   getFirstTrainArffFromDb()   - 
Static method in class test.mulan. MulanArffTest 
  
   getFirstValidArff2FromDb()   - 
Static method in class test.mulan. MulanArffTest 
  
   getIdByArffPath(String)   - 
Method in class uk.ac.ed.inf.enzml.weka. ArffPropsTableReader 
 Returns the first arff id in the table whose arff path matches the given
 string (or part of it)
   getInsertDataSqlStatement()   - 
Method in class test.dataharness. CreateDataTable 
  
   getInsertDataSqlStatement()   - 
Method in class test.dataharness. DataTableOneTest 
  
   getInsertDataSqlStatement()   - 
Method in class test.dataharness. DataTableThreeTest 
  
   getInsertDataSqlStatement()   - 
Method in class test.dataharness. DataTableTwoTest 
  
   getInstance1_2_empty_ArffId()   - 
Static method in class test.mulan.learn.traintest. EvaluationMetricsTest 
  
   getInstance1_2ArffId()   - 
Static method in class test.mulan.learn.traintest. EvaluationMetricsTest 
  
   getInstance3ArffId()   - 
Static method in class test.mulan.learn.traintest. EvaluationMetricsTest 
  
   getInstanceAttributeData()   - 
Method in class uk.ac.ed.inf.enzml.weka. DataSetDbLoader 
  
   getInstanceAttributeMap()   - 
Static method in class test.dataharness. DataTwo 
  
   getInstanceAttributeMap()   - 
Method in class uk.ac.ed.inf.enzml.weka. DataSetGenerator 
  
   getInstanceClassData()   - 
Method in class uk.ac.ed.inf.enzml.weka. DataSetDbLoader 
  
   getInstanceClassMap()   - 
Static method in class test.dataharness. DataTwo 
  
   getInstanceClassMap()   - 
Method in class uk.ac.ed.inf.enzml.weka. DataSetGenerator 
  
   getInstances()   - 
Method in class uk.ac.ed.inf.enzml.mulan. MulanArff 
  
   getInstances(DbReader, String)   - 
Method in class uk.ac.ed.inf.enzml.mulan.predict. MulanPredict 
  
   getInstances()   - 
Method in class uk.ac.ed.inf.enzml.weka. Arff 
  
   getInstances()   - 
Method in class uk.ac.ed.inf.enzml.weka. DataSetGenerator 
  
   getInstancesFactory()   - 
Method in class uk.ac.ed.inf.enzml.weka. DataSetGenerator 
  
   getInstancesNames()   - 
Method in class uk.ac.ed.inf.enzml.mulan. MulanArff 
  
   getInstancesNames()   - 
Method in class uk.ac.ed.inf.enzml.weka. DataSetGenerator 
  
   getInstancesNames()   - 
Method in class uk.ac.ed.inf.enzml.weka. InstancesFiller 
  
   getInstancesNamesFromFile(String)   - 
Static method in class uk.ac.ed.inf.enzml.mulan. MulanArffRecord 
 Gets the instances names directly from the comment line above each
 instance
   getLearner()   - 
Method in class uk.ac.ed.inf.enzml.mulan.learn. MulanCrossEvaluator 
  
   getLearner()   - 
Method in class uk.ac.ed.inf.enzml.mulan.learn. MulanCrossExperimenter 
 public String getExperimentPropsPath() { return m_experimentPropsFile; }
   getLearner()   - 
Method in class uk.ac.ed.inf.enzml.mulan.learn. MulanLearner 
  
   getLearner()   - 
Method in class uk.ac.ed.inf.enzml.mulan.learn. MulanSerializer 
  
   getLearnerBaseType()   - 
Method in class uk.ac.ed.inf.enzml.mulan.learn. MulanLearner 
  
   getLearnerFullName()   - 
Method in class uk.ac.ed.inf.enzml.mulan.learn. MulanLearner 
 Get the full type of a learner: multi-label learner base + algorithm
 within
   getLearnerName(Object)   - 
Static method in class uk.ac.ed.inf.enzml.mulan.learn. MulanLearner 
  
   getLearners()   - 
Static method in class uk.ac.ed.inf.enzml.mulan.learn. MulanLearners 
  
   getLearnerType()   - 
Method in class uk.ac.ed.inf.enzml.mulan.learn. MulanLearner 
  
   getLoadedMulan(String)   - 
Static method in class test.mulan. MulanArffTest 
  
   getLog()   - 
Method in class uk.ac.ed.inf.enzml.weka. DataSetWriter 
  
   getLogFile()   - 
Method in class uk.ac.ed.inf.enzml.mulan.learn. MulanSerializer 
  
   getManager()   - 
Method in class uk.ac.ed.inf.enzml.mulan.attributesfilter. AttributesFilteredDataSetGenerator 
  
   getManager()   - 
Method in class uk.ac.ed.inf.enzml.weka. DataSetChecker 
  
   getManager()   - 
Method in class uk.ac.ed.inf.enzml.weka. DataSetGenerator 
  
   getManager1()   - 
Static method in class test.weka. ArffPropsTableManagerTest 
  
   getManager2()   - 
Static method in class test.weka. ArffPropsTableManagerTest 
  
   getMap(String)   - 
Method in class uk.ac.ed.inf.enzml.weka. DataSetDbLoader 
  
   getMeasureFieldNames()   - 
Static method in class uk.ac.ed.inf.enzml.mulan.learn. ExperimentTable 
 A hashtable containing for each evaluation measure the corresponding
 table field name.
   getMeasureFields()   - 
Static method in class uk.ac.ed.inf.enzml.mulan.learn. ExperimentTable 
 A hashtable containing for each measure field name the corresponding
 table field object (name + sql data type).
   getMeasureNames()   - 
Static method in class uk.ac.ed.inf.enzml.mulan.learn. EvaluationParameters 
 Get a list containing the evaluation measure names.
   getMeasureTableField(String)   - 
Static method in class uk.ac.ed.inf.enzml.mulan.learn. ExperimentTable 
 For each evaluation measure, gets the corresponding table field The field
 name is the evaluation measure name, all lower-case, with spaces and
 dashes substituted with underscores.
   getMulan(String)   - 
Static method in class test.mulan. MulanArffTest 
  
   getMulanArff1()   - 
Static method in class test.mulan. MulanArffTest 
  
   getMulanArff1LoadedAndWritten()   - 
Static method in class test.mulan. MulanArffTest 
  
   getMulanArffTest()   - 
Static method in class test.mulan. MulanArffTest 
  
   getMulanArffTestLoadedAndWritten()   - 
Static method in class test.mulan. MulanArffTest 
  
   getMulanArffTrain()   - 
Static method in class test.mulan. MulanArffTest 
  
   getMulanArffTrainLoadedAndWritten()   - 
Static method in class test.mulan. MulanArffTest 
  
   getMulanEvaluator2()   - 
Static method in class test.mulan.learn. CrossEvaluatorTest 
  
   getMulanExperimenter(int)   - 
Static method in class test.mulan.learn. ExperimenterTest 
  
   getMulanExperimenter(int, String, String)   - 
Static method in class test.mulan.learn. ExperimenterTest 
  
   getMulanExperimenter2()   - 
Static method in class test.mulan.learn. ExperimenterTest 
  
   getMulanExperimenterFilterTrainset()   - 
Static method in class test.mulan.learn. ExperimenterTest 
  
   getMulanLearnerBRk1NN()   - 
Static method in class test.mulan.learn. LearnerTest 
  
   getMulanLearnerOneR()   - 
Static method in class test.mulan.learn. LearnerTest 
  
   getMulanLearnerZeroR()   - 
Static method in class test.mulan.learn. LearnerTest 
  
   getMulanPredict(int, int)   - 
Static method in class test.mulan.predict. MulanPredictTest 
  
   getMulanPredictOnAttributesFilteredFiles()   - 
Static method in class test.mulan.predict. MulanPredictTest 
  
   getMulanPredictPlain()   - 
Static method in class test.mulan.predict. MulanPredictTest 
  
   getMulanSerializer2()   - 
Static method in class test.mulan.learn. SerializerTest 
  
   getMulanSerializerFilterTrainset()   - 
Static method in class test.mulan.learn. SerializerTest 
  
   getMulanTrainTestEvaluatorBRk1NN()   - 
Static method in class test.mulan.learn.traintest. TrainTestEvaluatorTest 
  
   getMulanTrainTestExperBRk1NN()   - 
Static method in class test.mulan.learn.traintest. TrainTestExperimenterTest 
  
   getMulanTrainTestExperBRk1NNById(int, int)   - 
Static method in class test.mulan.learn.traintest. TrainTestExperimenterTest 
 public static TrainTestExperimenter
 getFirstMulanTrainTestExpMLk1NNFromDb() { // train arff should have
 pattern "trainset_" // the testset has pattern "testset_" // the test set
 is filtered by the training set to generate a file // called: //
 testset_x_filteredby_trainset_y
 
 int trainId = MulanArffGeneratorTest.getFirstTrainArff(); int testId =
 MulanArffGeneratorTest.getFirstTestArff(); return
 TrainTestExperimenterTest.getMulanTrainTestExperMLk1NNById( trainId,
 testId);
 
 }
   getMulanTrainTestExperById(int, int, MulanLearner)   - 
Static method in class test.mulan.learn.traintest. TrainTestExperimenterTest 
  
   getMultiLabelInstances(int)   - 
Method in class uk.ac.ed.inf.enzml.mulan.database. MulanDbReader 
  
   getNewEvaluator()   - 
Method in class uk.ac.ed.inf.enzml.mulan.learn.traintest. TrainTestExperimenterSerialized 
  
   getNewFilteredArffs()   - 
Static method in class test.mulan.attributesfilter. AttributesFilteredArffTest 
 Get the new, filtered, train and test arff files
   getNewTestArffId()   - 
Method in class uk.ac.ed.inf.enzml.mulan.attributesfilter. AttributesFilter 
  
   getNewTestDataSetGenerator()   - 
Method in class test.mulan.attributesfilter. AttributesFilteredDataSetGeneratorTest 
  
   getNewTrainArffId()   - 
Method in class uk.ac.ed.inf.enzml.mulan.attributesfilter. AttributesFilter 
  
   getNumberOfAttributes()   - 
Method in class uk.ac.ed.inf.enzml.mulan. MulanDataSetGenerator 
 Get the number of distinct attribute values (all attribute values - class
 values)
   getNumberOfAttributes()   - 
Method in class uk.ac.ed.inf.enzml.weka. DataSetGenerator 
  
   getNumberOfClassValues()   - 
Method in class uk.ac.ed.inf.enzml.weka. DataSetGenerator 
  
   getNumberOfInstanceAttributeCouples()   - 
Method in class uk.ac.ed.inf.enzml.mulan. MulanDataSetGenerator 
 Get the number of distinct instance-attribute couples of values by
 subtracting the number of instance-class_attribute values from the total
 number.
   getNumberOfInstanceAttributeCouples()   - 
Method in class uk.ac.ed.inf.enzml.weka. DataSetGenerator 
  
   getNumberOfInstanceClassCouples()   - 
Method in class uk.ac.ed.inf.enzml.weka. DataSetGenerator 
  
   getNumberOfInstances()   - 
Method in class uk.ac.ed.inf.enzml.weka. DataSetGenerator 
  
   getOldTestArffId()   - 
Method in class uk.ac.ed.inf.enzml.mulan.attributesfilter. AttributesFilter 
  
   getOldTrainArffId()   - 
Method in class uk.ac.ed.inf.enzml.mulan.attributesfilter. AttributesFilter 
 public MulanArff getTrainArff() { return m_trainArff; }
   getPredictionsFile()   - 
Method in class uk.ac.ed.inf.enzml.mulan.predict. MulanPredict 
 The full path to the predictions file
   getProperties()   - 
Method in class uk.ac.ed.inf.enzml.weka. ArffProperties 
 Get the properties
   getProperties()   - 
Method in class uk.ac.ed.inf.enzml.weka. DataSetChecker 
  
   getPropertiesFilePath()   - 
Method in class uk.ac.ed.inf.enzml.weka. ArffProperties 
 Get the properties file path
   getPropertiesFromDb(int)   - 
Method in class uk.ac.ed.inf.enzml.weka. ArffPropsTableReader 
  
   getProperty(String)   - 
Method in class uk.ac.ed.inf.enzml.weka. ArffProperties 
 Get a property value
   getPropertyValueFromTable(String, int)   - 
Method in class uk.ac.ed.inf.enzml.weka. ArffPropsTableReader 
  
   getReader()   - 
Method in class uk.ac.ed.inf.enzml.weka. ArffPropsTableManager 
  
   getReader1()   - 
Static method in class test.weka. ArffPropsTableReaderTest 
  
   getReader2()   - 
Static method in class test.weka. ArffPropsTableReaderTest 
  
   getReaderTest()   - 
Static method in class test.weka. ArffPropsTableReaderTest 
  
   getReaderTrain()   - 
Static method in class test.weka. ArffPropsTableReaderTest 
  
   getRecord(int)   - 
Static method in class test.mulan. MulanArffRecordTest 
  
   getResultsCanBeSaved()   - 
Method in class uk.ac.ed.inf.enzml.mulan.learn. ResultsSaver 
  
   getResultsDbManager()   - 
Method in class uk.ac.ed.inf.enzml.mulan.learn. ResultsSaver 
  
   getResultsDbManager()   - 
Method in class uk.ac.ed.inf.enzml.weka. Arff 
  
   getResultsSaver()   - 
Static method in class test.mulan.learn. ResultsSaverTest 
  
   getResultsSaver()   - 
Method in class uk.ac.ed.inf.enzml.mulan.learn. MulanCrossExperimenter 
  
   getResultsTableManager()   - 
Method in class uk.ac.ed.inf.enzml.mulan.learn. ResultsSaver 
  
   getStartTime()   - 
Method in class uk.ac.ed.inf.enzml.mulan.learn. MulanCrossEvaluator 
  
   getTableManager()   - 
Method in class uk.ac.ed.inf.enzml.mulan.learn. ResultsSaver 
  
   getTableName()   - 
Method in class test.dataharness. CreateDataTable 
  
   getTableReader()   - 
Method in class test.dataharness. CreateDataTable 
  
   getTestArffRecord()   - 
Method in class uk.ac.ed.inf.enzml.mulan.learn.traintest. TrainTestExperimenter 
 public TrainTestEvaluator getEvaluator() { return (TrainTestEvaluator)
 m_evaluator; }
   getTrainArff()   - 
Method in class uk.ac.ed.inf.enzml.mulan.predict. MulanPredict 
  
   getTrainArffRecord()   - 
Method in class uk.ac.ed.inf.enzml.mulan.learn. MulanCrossExperimenter 
  
   getTrainAttributesAndTrainTestClasses()   - 
Method in class uk.ac.ed.inf.enzml.mulan.attributesfilter. AttributesFilter 
  
   getTrainedModel()   - 
Method in class uk.ac.ed.inf.enzml.mulan.predict. MulanPredictWithTrainedModel 
  
   getTrainedModelFile()   - 
Method in class uk.ac.ed.inf.enzml.mulan.learn.traintest. TrainTestEvaluatorSerialised 
  
   getTrainedModelFile()   - 
Method in class uk.ac.ed.inf.enzml.mulan.learn.traintest. TrainTestExperimenterSerialized 
  
   getTrainXmlPath()   - 
Method in class uk.ac.ed.inf.enzml.mulan.attributesfilter. AttributesFilter 
  
   getUnlabeledArff()   - 
Method in class uk.ac.ed.inf.enzml.mulan.predict. MulanPredict 
  
   getUnlabeledData()   - 
Method in class uk.ac.ed.inf.enzml.mulan.predict. MulanPredict 
  
   getValueOptions()   - 
Method in class test.weka. AttributeFactoryTest 
  
   getWrittenMulan(String)   - 
Static method in class test.mulan. MulanArffTest 
  
   getXmlFileName()   - 
Method in class uk.ac.ed.inf.enzml.mulan. MulanArffRecord 
  
   getXmlFilePath()   - 
Method in class uk.ac.ed.inf.enzml.mulan.learn. MulanCrossEvaluator 
  
   getXmlPath()   - 
Method in class uk.ac.ed.inf.enzml.mulan. MulanArff 
  
   getXmlPathById(int)   - 
Method in class uk.ac.ed.inf.enzml.mulan.attributesfilter. AttributesFilter 
  
   getXMLpathFromArffPath(String)   - 
Static method in class uk.ac.ed.inf.enzml.mulan. MulanArff 
 Generates the xml file path: same name+path as .arff file, but .xml
 termination
   getXmlQueryById(int)   - 
Method in class uk.ac.ed.inf.enzml.mulan.attributesfilter. AttributesFilter 
  
 
 


 
   
  
 
 
 
   
 
   
          Overview     
        Package   
        Class   
        Use   
          Tree     
          Deprecated     
       Index    
          Help     
   
 
 
  
 
 
 

 
  
   PREV LETTER   
   NEXT LETTER    
  
    FRAMES     
   NO FRAMES     
  
    All Classes  ');
  }
  //-->
 
 
    All Classes  
 


  
 
 
  
 

 A   B   C   D   E   F   G   I   K   L   M   N   P   R   S   T   U   V   W   X   

 
 
