# Supplementary material for: EnzML: multi-label prediction of enzyme classes using InterPro signatures
Source: BMC Bioinformatics. 2012 Apr 25;13:61. doi: 10.1186/1471-2105-13-61 (PMC3483700; doi:10.1186/1471-2105-13-61)
Supplement: Addtional file 5 — The Java code to format the data files, evaluate and predict. The file enzml_java_code.tar.gz contains the Java code used to format database data to ARFF and XML formats, to execute cross and train-test (jackknife) evaluations and to record evaluation results to database. More information is included in the readme.txt file and the Javadoc files. The code can be used with a MySQL database. To use a different database software, other JDBC drivers might be required. [file 1471-2105-13-61-S5.gz › java_code/enzml2011/doc/index-files/index-3.html]

C-Index


---


|  |  |  |  |  |  |  |  |  |  |  |
| --- | --- | --- | --- | --- | --- | --- | --- | --- | --- | --- |
| |  |  |  |  |  |  |  |  | | --- | --- | --- | --- | --- | --- | --- | --- | | **Overview** | Package | Class | Use | **Tree** | **Deprecated** | **Index** | **Help** | | |  |
| **PREV LETTER**   **NEXT LETTER** | **FRAMES**    **NO FRAMES**     **All Classes** |


A B C D E F G I K L M N P R S T U V W X 

---


## **C**

**checkAndInitialise(String, String)** - Method in class uk.ac.ed.inf.enzml.weka.Arff: **checkDouble(String, Double)** - Method in class test.mulan.learn.ResultCheckerTest: **checkPropertiesKeys(Properties)** - Method in class uk.ac.ed.inf.enzml.weka.ArffProperties: **CLASS1** - Static variable in class test.dataharness.DataOne: **CLASS2** - Static variable in class test.dataharness.DataOne: **CLASS3** - Static variable in class test.dataharness.DataOne: **CLASS4** - Static variable in class test.dataharness.DataOne: **CLASS\_FIELD** - Static variable in class test.dataharness.CreateDataTable: **CLASS\_NAME\_PROP** - Static variable in class uk.ac.ed.inf.enzml.weka.ArffProperties: The class name. **CLASS\_TO\_STRING** - Static variable in class test.dataharness.DataOne: **classColumn()** - Static method in class test.dataharness.DataOne: **classValues()** - Static method in class test.dataharness.DataOne: Results of a select distinct instance, class query **CONFIDENCE** - Static variable in class uk.ac.ed.inf.enzml.mulan.predict.MulanPredict: **coreKeys()** - Method in class uk.ac.ed.inf.enzml.weka.ArffProperties: The core ARFF properties, essential to generate a Weka ARFF file **corePropertiesAreCorrect(Properties)** - Method in class uk.ac.ed.inf.enzml.weka.ArffProperties: **createAndloadDataSet()** - Method in class uk.ac.ed.inf.enzml.weka.DataSetDbLoader: Create a data set and load it with data from database **CreateDataTable** - Class in test.dataharness: Create a table for test instances (and their attributes and classes) **CreateDataTable()** - Constructor for class test.dataharness.CreateDataTable: **createEvaluationRecord(int, int, int, String, String, MulanLearner, Evaluation, TableManager)** - Static method in class uk.ac.ed.inf.enzml.mulan.learn.ResultsFormatter: Add timestamps and identifiers to the cross evaluation results **createExperimentsTable()** - Method in class uk.ac.ed.inf.enzml.mulan.database.MulanDbCreator: **createNewArff(int)** - Method in class uk.ac.ed.inf.enzml.mulan.attributesfilter.AttributesFilter: **createNewTestArff()** - Method in class uk.ac.ed.inf.enzml.mulan.attributesfilter.AttributesFilter: **createNewTrainArff()** - Method in class uk.ac.ed.inf.enzml.mulan.attributesfilter.AttributesFilter: **createRow()** - Method in class uk.ac.ed.inf.enzml.weka.ArffPropsTableManager: **createTables()** - Method in class uk.ac.ed.inf.enzml.mulan.database.MulanDbCreator: **createTestTable(String)** - Method in class test.dataharness.CreateDataTable: **crossEvaluateAndSaveResults(int)** - Method in class uk.ac.ed.inf.enzml.mulan.learn.MulanCrossExperimenter: Cross validates and saves results to database **CrossEvaluatorTest** - Class in test.mulan.learn: Class **CrossEvaluatorTest()** - Constructor for class test.mulan.learn.CrossEvaluatorTest

---


|  |  |  |  |  |  |  |  |  |  |  |
| --- | --- | --- | --- | --- | --- | --- | --- | --- | --- | --- |
| |  |  |  |  |  |  |  |  | | --- | --- | --- | --- | --- | --- | --- | --- | | **Overview** | Package | Class | Use | **Tree** | **Deprecated** | **Index** | **Help** | | |  |
| **PREV LETTER**   **NEXT LETTER** | **FRAMES**    **NO FRAMES**     **All Classes** |


A B C D E F G I K L M N P R S T U V W X 

---
