# Supplementary material for: EnzML: multi-label prediction of enzyme classes using InterPro signatures
Source: BMC Bioinformatics. 2012 Apr 25;13:61. doi: 10.1186/1471-2105-13-61 (PMC3483700; doi:10.1186/1471-2105-13-61)
Supplement: Addtional file 5 — The Java code to format the data files, evaluate and predict. The file enzml_java_code.tar.gz contains the Java code used to format database data to ARFF and XML formats, to execute cross and train-test (jackknife) evaluations and to record evaluation results to database. More information is included in the readme.txt file and the Javadoc files. The code can be used with a MySQL database. To use a different database software, other JDBC drivers might be required. [file 1471-2105-13-61-S5.gz › java_code/enzml2011/doc/index-files/index-6.html]

F-Index


---


|  |  |  |  |  |  |  |  |  |  |  |
| --- | --- | --- | --- | --- | --- | --- | --- | --- | --- | --- |
| |  |  |  |  |  |  |  |  | | --- | --- | --- | --- | --- | --- | --- | --- | | **Overview** | Package | Class | Use | **Tree** | **Deprecated** | **Index** | **Help** | | |  |
| **PREV LETTER**   **NEXT LETTER** | **FRAMES**    **NO FRAMES**     **All Classes** |


A B C D E F G I K L M N P R S T U V W X 

---


## **F**

**fillAndWriteInstancesConditional()** - Method in class uk.ac.ed.inf.enzml.weka.DataSetGenerator: If memory is abundant and the instances content is required, store the instances. **fillArffButDontWrite()** - Method in class uk.ac.ed.inf.enzml.weka.Arff: Loads instances, but does not write the arff file or arff database record. **fillArffButDontWrite()** - Method in class uk.ac.ed.inf.enzml.weka.DataSetManager: **fillInstance(String, SparseInstance)** - Method in class uk.ac.ed.inf.enzml.mulan.MulanInstancesFiller: Consider all attributes! there is no last class attribute. **fillInstance(String, SparseInstance)** - Method in class uk.ac.ed.inf.enzml.weka.InstancesFiller: Returns an instance filled with all its attribute values. **fillInstances()** - Method in class uk.ac.ed.inf.enzml.weka.DataSetGenerator: **FILTER\_OLD\_TEST\_SET** - Static variable in class test.dataharness.TestProjectParameters: **FILTER\_TRAIN\_SET** - Static variable in class test.dataharness.TestProjectParameters: **formatFileName()** - Method in class uk.ac.ed.inf.enzml.mulan.learn.MulanSerializer: **fromEnumerationToVector(Enumeration<Attribute>)** - Static method in class uk.ac.ed.inf.enzml.mulan.AttributeUtils: **fromEnumerationToVector(Enumeration<Instance>)** - Static method in class uk.ac.ed.inf.enzml.mulan.InstanceUtils: **fullRun()** - Static method in class uk.ac.ed.inf.enzml.mulan.learn.traintest.TrainTestFullRun: Executes: 1.

---


|  |  |  |  |  |  |  |  |  |  |  |
| --- | --- | --- | --- | --- | --- | --- | --- | --- | --- | --- |
| |  |  |  |  |  |  |  |  | | --- | --- | --- | --- | --- | --- | --- | --- | | **Overview** | Package | Class | Use | **Tree** | **Deprecated** | **Index** | **Help** | | |  |
| **PREV LETTER**   **NEXT LETTER** | **FRAMES**    **NO FRAMES**     **All Classes** |


A B C D E F G I K L M N P R S T U V W X 

---
