# Supplementary material for: EnzML: multi-label prediction of enzyme classes using InterPro signatures
Source: BMC Bioinformatics. 2012 Apr 25;13:61. doi: 10.1186/1471-2105-13-61 (PMC3483700; doi:10.1186/1471-2105-13-61)
Supplement: Addtional file 5 — The Java code to format the data files, evaluate and predict. The file enzml_java_code.tar.gz contains the Java code used to format database data to ARFF and XML formats, to execute cross and train-test (jackknife) evaluations and to record evaluation results to database. More information is included in the readme.txt file and the Javadoc files. The code can be used with a MySQL database. To use a different database software, other JDBC drivers might be required. [file 1471-2105-13-61-S5.gz › java_code/enzml2011/doc/index-files/index-16.html]

T-Index


---


|  |  |  |  |  |  |  |  |  |  |  |
| --- | --- | --- | --- | --- | --- | --- | --- | --- | --- | --- |
| |  |  |  |  |  |  |  |  | | --- | --- | --- | --- | --- | --- | --- | --- | | **Overview** | Package | Class | Use | **Tree** | **Deprecated** | **Index** | **Help** | | |  |
| **PREV LETTER**   **NEXT LETTER** | **FRAMES**    **NO FRAMES**     **All Classes** |


A B C D E F G I K L M N P R S T U V W X 

---


## **T**

**TABLE\_NAME** - Static variable in class uk.ac.ed.inf.enzml.weka.ArffPropsTable: **TABLE\_ONE\_NAME** - Static variable in class test.dataharness.DataTableOneTest: **TABLE\_TWO\_NAME** - Static variable in class test.dataharness.DataTableThreeTest: **TABLE\_TWO\_NAME** - Static variable in class test.dataharness.DataTableTwoTest: **tearDown()** - Method in class test.mulan.learn.ResultsSaverTest: **test** - package test: **test()** - Method in class test.dataharness.TestProjectParametersTest: **test.dataharness** - package test.dataharness: **test.mulan** - package test.mulan: **test.mulan.attributesfilter** - package test.mulan.attributesfilter: **test.mulan.learn** - package test.mulan.learn: **test.mulan.learn.database** - package test.mulan.learn.database: **test.mulan.learn.traintest** - package test.mulan.learn.traintest: **test.mulan.predict** - package test.mulan.predict: **test.weka** - package test.weka: **TEST\_ARFF\_DB\_PROPS** - Static variable in class test.dataharness.TestProjectParameters: **TEST\_ARFF\_FILES\_PATH** - Static variable in class test.dataharness.TestProjectParameters: **TEST\_ARFF\_ID** - Static variable in class uk.ac.ed.inf.enzml.mulan.learn.ExperimentTable: the table column to store the arff file id **TEST\_ARFF\_PROPS\_1** - Static variable in class test.dataharness.TestProjectParameters: **TEST\_ARFF\_PROPS\_2** - Static variable in class test.dataharness.TestProjectParameters: **TEST\_ARFF\_PROPS\_NO\_XML** - Static variable in class test.dataharness.TestProjectParameters: **TEST\_ARFF\_PROPS\_PATH** - Static variable in class test.dataharness.TestProjectParameters: **TEST\_BASE\_DATA\_PATH** - Static variable in class test.dataharness.TestProjectParameters: **TEST\_BASE\_PATH** - Static variable in class test.dataharness.TestProjectParameters: **TEST\_DATA\_DIR** - Static variable in class test.dataharness.TestProjectParameters: **TEST\_DB\_CONN\_PROPERTIES** - Static variable in class test.dataharness.TestProjectParameters: **TEST\_MACHINE\_LEARNING\_PROPS** - Static variable in class test.dataharness.TestProjectParameters: **TEST\_ML\_PROPS\_PATH** - Static variable in class test.dataharness.TestProjectParameters: **TEST\_MODEL\_FOLDER** - Static variable in class test.dataharness.TestProjectParameters: **TEST\_PREDICTIONS\_FOLDER** - Static variable in class test.dataharness.TestProjectParameters: **TEST\_PROPS\_PATH** - Static variable in class test.dataharness.TestProjectParameters: **TEST\_RESULTS\_FOLDER** - Static variable in class test.dataharness.TestProjectParameters: **TEST\_ROLE** - Static variable in class test.mulan.attributesfilter.AttributesFilteredArffTest: **testANewFilteredFileContent(int, int, String[])** - Static method in class test.mulan.attributesfilter.AttributesFilteredArffTest: **testArffName()** - Method in class test.mulan.attributesfilter.AttributesFilteredArffTest: **testCheckTrainTestEvalRow(Vector<TableRow>)** - Static method in class test.mulan.learn.traintest.TrainTestEvaluatorTest: Executes generic tests (number of metrics etc.) on a table row containing evaluation results. **testCreateArffFileTable()** - Method in class test.weka.ArffPropsTableManagerTest: **testCreateAttribute()** - Method in class test.weka.WekaTest: **testCreateAttributeFactory()** - Method in class test.mulan.attributesfilter.AttributesFilteredDataSetGeneratorTest: **testCreateAttributesList()** - Method in class test.mulan.MulanDataSetGeneratorTest: **testCreateAttributeVector()** - Method in class test.weka.WekaTest: **testCreateEvaluationRecord()** - Method in class test.mulan.learn.ResultsFormatterTest: **testCreateExperimentsTable()** - Method in class test.mulan.learn.database.MulanDbCreatorTest: **testCreateInstance()** - Method in class test.weka.WekaTest: **testCreateInstances()** - Method in class test.weka.WekaTest: **testCreateNewTestArff()** - Method in class test.mulan.attributesfilter.AttributesFilterTest: **testCrossValidate1()** - Method in class test.mulan.learn.CrossEvaluatorTest: **testDatabase()** - Method in class test.dataharness.DatabaseTest: **testDataStatistics()** - Method in class test.mulan.MulanDataSetGeneratorTest: **testDataStatistics()** - Method in class test.weka.DataSetWriterTest: **testDbConn()** - Method in class test.mulan.learn.database.MulanDbManagerTest: **testDeserialize()** - Method in class test.mulan.learn.SerializerTest: **testEvalResultsInstance12()** - Method in class test.mulan.learn.traintest.EvaluationMetricsTest: Train on instances 1 and 2 and test on instances 1 and 2: should have perfect precision/accuracy/recall **testEvalResultsTrainInst12TestInst3()** - Method in class test.mulan.learn.traintest.EvaluationMetricsTest: Train on instances 1 and 2 and test on instances 3: should have zero precision/accuracy/recall **testEvaluation1()** - Method in class test.mulan.learn.traintest.TrainTestEvaluatorTest: **testEvaluationResults1(TableRow)** - Static method in class test.mulan.learn.traintest.TrainTestEvaluatorTest: **testFillInstances()** - Method in class test.mulan.MulanInstancesFillerTest: **testFillNonExistingInstances()** - Method in class test.mulan.MulanInstancesFillerTest: **testGenerateArffFileFromDb()** - Method in class test.weka.ArffTest: **testGenerateArffFileFromDb2()** - Method in class test.mulan.MulanArffTest: public static String getExpectedMulanArff1() { return "@relation test\_data\n\n" + MulanArffTest.ATT1\_TO\_STRING + "\n" + MulanArffTest.ATT2\_TO\_STRING + "\n" + MulanArffTest.ATT3\_TO\_STRING + "\n" + MulanArffTest.ATT4\_TO\_STRING + "\n" + MulanArffTest.ATT5\_TO\_STRING + "\n" + "@attribute class1 {0,1}\n" + "@attribute class2 {0,1}\n" + "@attribute class3 {0,1}\n" + "@attribute class4 {0,1}\n" + "\n@data\n" + "% " + DataOne.INST1 + "\n" + MulanArffTest.arffInstance1 + "\n" + "% " + DataOne.INST2 + "\n" + MulanArffTest.arffInstance2 + "\n" + "% " + DataOne.INST4 + "\n" + MulanArffTest.arffInstance4 + "\n" + "% " + DataOne.INSTATTVOID + "\n" + MulanArffTest.arffInstanceAttributeVoid + "\n" + "% " + DataOne.INSTCLASSATTVOID + "\n" + MulanArffTest.arffInstanceClassAttributeVoid + "\n" + "% " + DataOne.INSTCLASSVOID + "\n" + MulanArffTest.arffInstanceClassVoid; } public static String getExpectedMulanArff2() { String attributes = "@relation test\_ec\_data\n\n@attribute 1.-.-.- {0,1}\n@attribute 1.1.-.- {0,1}\n@attribute 1.1.1.- {0,1}\n@attribute 1.1.1.1 {0,1}\n@attribute 2.-.-.- {0,1}\n@attribute 2.2.-.- {0,1}\n@attribute 2.2.2.- {0,1}\n@attribute 2.2.2.2 {0,1}\n@attribute 3.-.-.- {0,1}\n@attribute 3.3.-.- {0,1}\n@attribute 3.3.3.- {0,1}\n@attribute 3.3.3.3 {0,1}\n@attribute att1 {0,1}\n@attribute att2 {0,1}\n@attribute att3 {0,1}\n@attribute att4 {0,1}\n@attribute att5 {0,1}\n\n"; String instances = "@data\n% inst1\n{0 1,1 1,2 1,3 1,12 1}\n% inst10\n{4 1,5 1,6 1,7 1,16 1}\n% inst11\n{4 1,5 1,6 1,7 1}\n% inst12\n{12 1}\n% inst13\n{}\n% inst2\n{4 1,5 1,6 1,7 1,14 1}\n% inst3\n{0 1,1 1,2 1,3 1,12 1}\n% inst4\n{4 1,5 1,6 1,7 1,14 1}\n% inst5\n{0 1,1 1,2 1,3 1,12 1}\n% inst6\n{4 1,5 1,6 1,7 1,14 1}\n% inst7\n{0 1,1 1,2 1,3 1,12 1}\n% inst8\n{4 1,5 1,6 1,7 1,14 1}\n% inst9\n{0 1,1 1,2 1,3 1,15 1}\n% instA\n{8 1,9 1,10 1,11 1,13 1}\n% instB\n{4 1,5 1,6 1,7 1,14 1}\n% instC\n{4 1,5 1,6 1,7 1,13 1,14 1}\n% inst\_att\_void\n{4 1,5 1,6 1,7 1}\n% inst\_class\_att\_void\n{}\n% inst\_class\_void\n{13 1}"; return attributes + instances; } **testGenerateBinaryAttribute()** - Method in class test.weka.AttributeFactoryTest: **testGenerateNominalAttribute()** - Method in class test.weka.AttributeFactoryTest: **testGetArff()** - Method in class test.weka.DataSetManagerTest: **testGetArffPath()** - Method in class test.mulan.learn.database.MulanDbReaderTest: **testGetAttributes()** - Method in class test.weka.DataSetGeneratorTest: **testGetColumnDefinitions()** - Method in class test.weka.ArffPropsTableTest: **testGetColumnsList()** - Method in class test.mulan.learn.ExperimentTableTest: **testGetDataSetName()** - Static method in class test.weka.DataSetManagerTest: **testGetExampleBasedAccuracyMeasure()** - Method in class test.mulan.learn.EvaluationParametersTest: **testGetFilteredTestArffRecord()** - Method in class test.mulan.learn.traintest.TrainTestExperimenterTest: **testGetInstancesNamesFromFile()** - Method in class test.mulan.MulanArffRecordTest: **testGetLearnerBaseType()** - Method in class test.mulan.learn.LearnerTest: **testGetLearnerType()** - Method in class test.mulan.learn.LearnerTest: **testGetLernerFullName()** - Method in class test.mulan.learn.LearnerTest: **testGetMeasures()** - Method in class test.mulan.learn.EvaluationParametersTest: **testGetOptions()** - Method in class test.weka.AttributeFactoryTest: **testGetProperties1()** - Method in class test.dataharness.ArffPropsOneTest: **testGetProperties1()** - Method in class test.dataharness.ArffPropsTwoTest: **testGetPropertiesFilePath1()** - Method in class test.dataharness.ArffPropsOneTest: **testGetPropertiesFilePath2()** - Method in class test.dataharness.ArffPropsTwoTest: **testGetProperty1ValueFromTable()** - Method in class test.weka.ArffPropsTableReaderTest: **testGetResultsCanBeSaved()** - Method in class test.mulan.learn.ResultsSaverTest: **testGetStatistics()** - Method in class test.weka.DataSetGeneratorTest: **testGetTableField()** - Method in class test.mulan.learn.ExperimentTableTest: **testHeader(String)** - Method in class test.mulan.predict.MulanPredictTest: **testInitialisationKO()** - Method in class test.weka.DataSetCheckerTest: No xml query property, the initialisation should fail \*before\* generating the arff and xml files (and not waste time in generating the arff file and then fail at the generation of the xml file) **testInitialisationOK()** - Method in class test.weka.DataSetCheckerTest: **testInstancesSection()** - Method in class test.weka.DataSetWriterTest: **testLoadDataMulan()** - Method in class test.mulan.MulanDataSetDbLoaderTest: **testLoadDataTableOne()** - Method in class test.weka.DataSetDbLoaderTest: **testLogHeader()** - Method in class test.weka.DataSetWriterTest: **testLogTail()** - Method in class test.weka.DataSetWriterTest: **testMetrics\_Train\_1\_2\_empty\_Test\_3(TableRow)** - Static method in class test.mulan.learn.traintest.EvaluationMetricsTest: **testMetrics\_Train\_1\_2\_Test\_3(TableRow)** - Static method in class test.mulan.learn.traintest.EvaluationMetricsTest: **testMetricsTrain\_1\_2\_Test\_1\_2(TableRow)** - Static method in class test.mulan.learn.traintest.EvaluationMetricsTest: **testNewTestsetFile()** - Method in class test.mulan.attributesfilter.AttributesFilteredArffTest: **testNewTrainsetFile()** - Method in class test.mulan.attributesfilter.AttributesFilteredArffTest: **testPrintPredictions()** - Method in class test.mulan.predict.MulanPredictTest: **testPrintPredictionsAttributesFiltered()** - Method in class test.mulan.predict.MulanPredictTest: **TestProjectParameters** - Class in test.dataharness: **TestProjectParameters()** - Constructor for class test.dataharness.TestProjectParameters: **TestProjectParametersTest** - Class in test.dataharness: Class **TestProjectParametersTest()** - Constructor for class test.dataharness.TestProjectParametersTest: **testPropsFileIsReadable(String)** - Method in class test.dataharness.ArffPropsFilesTest: **testPropsFiles()** - Method in class test.dataharness.ArffPropsFilesTest: **testQueries()** - Method in class test.dataharness.ArffPropsQueriesOneTest: **testQueries(ArffProperties, String, String, String)** - Static method in class test.dataharness.ArffPropsQueriesTest: Test queries **testQueries()** - Method in class test.dataharness.ArffPropsQueriesTwoTest: **testQueryResults(String, int, Vector<String>)** - Static method in class test.dataharness.ArffPropsQueriesTest: **testRun()** - Method in class test.mulan.learn.ExperimenterTest: **testRunningTime()** - Method in class test.mulan.learn.CrossEvaluatorTest: public void testEvaluationResults() { MulanEvaluator evaluator = MulanEvaluatorTest.getMulanEvaluator(); assertFalse(evaluator == null); if (evaluator != null) { Evaluation[] results = evaluator.getEvaluationResults(); assertEquals(10, results.length); Evaluation e0 = results[0]; System.out.println(e0.toString()); java.util.List measures = e0.getMeasures(); assertEquals(22, measures.size()); // hamming loss Measure m0 = measures.get(0); assertEquals("Hamming Loss", m0.getName()); double value = m0.getValue(); System.out.println("MulanEvaluatorTest. **testSaveProperties1ToTable()** - Method in class test.weka.ArffPropsTableManagerTest: **testSaveResults()** - Method in class test.mulan.learn.ResultsSaverTest: **testSerializeMlkNN2()** - Static method in class test.mulan.learn.SerializerTest: **testSetAttributeValue()** - Method in class test.weka.WekaTest: **testSetClass()** - Method in class test.weka.WekaTest: **testTableContent()** - Method in class test.dataharness.DataTableOneTest: **testTableContent()** - Method in class test.dataharness.DataTableThreeTest: **testTableContent()** - Method in class test.dataharness.DataTableTwoTest: **testTableInstanceAttribute(String, String)** - Method in class test.dataharness.CreateDataTable: **testTableInstanceClass(String, String)** - Method in class test.dataharness.CreateDataTable: **testTrain\_1\_2\_empty\_Test\_3()** - Method in class test.mulan.learn.traintest.EvaluationMetricsTest: Train on instances 1 and 2 and empty and test on instances 3, which is empty once filtered **testWriteArff()** - Method in class test.mulan.MulanDataSetManagerTest: **testWriteInstance()** - Method in class test.weka.InstancesFillerTest: public void testFillNonExistingInstances() { InstancesFactory factory = InstancesFactoryTest.instancesFactory(); SparseInstance instance = new SparseInstance(factory.getDataSet() .getAttributes().size()); // set the dataset (Instances) for the instance instance.setDataset(factory.getDataSet().getInstances()); // fill instance attribute values instance = factory.fillInstance("empty", instance); assertEquals("@attribute att1 {n,y}", instance.attribute(0).toString()); assertEquals("@attribute att3 {n,y}", instance.attribute(2).toString()); assertEquals(4, instance.classIndex()); assertEquals("NaN", Double.toString(instance.classValue())); assertEquals(4, instance.index(0)); assertEquals(5, instance.numAttributes()); assertEquals(1, instance.numValues()); assertEquals("{4 ?}", instance.toString()); } **testWriteOnDatabase()** - Method in class test.weka.ArffPropsTableManagerTest: **TIMESTAMP\_FIELD** - Static variable in class uk.ac.ed.inf.enzml.weka.ArffProperties: the timestamp field **toString()** - Method in class uk.ac.ed.inf.enzml.mulan.learn.traintest.TrainTestExperimenterSerialized: **toString()** - Method in class uk.ac.ed.inf.enzml.weka.ArffProperties: **TRAIN\_ARFF\_ID** - Static variable in class uk.ac.ed.inf.enzml.mulan.learn.ExperimentTable: the table column to store the arff file id **TRAIN\_ROLE** - Static variable in class test.mulan.attributesfilter.AttributesFilteredArffTest: **TrainTestEvaluator** - Class in uk.ac.ed.inf.enzml.mulan.learn.traintest: Train/test evaluator. **TrainTestEvaluator(TrainTestExperimenter)** - Constructor for class uk.ac.ed.inf.enzml.mulan.learn.traintest.TrainTestEvaluator: **TrainTestEvaluatorSerialised** - Class in uk.ac.ed.inf.enzml.mulan.learn.traintest: Class **TrainTestEvaluatorSerialised(TrainTestExperimenter, String)** - Constructor for class uk.ac.ed.inf.enzml.mulan.learn.traintest.TrainTestEvaluatorSerialised: **TrainTestEvaluatorTest** - Class in test.mulan.learn.traintest: **TrainTestEvaluatorTest()** - Constructor for class test.mulan.learn.traintest.TrainTestEvaluatorTest: **TrainTestExperimenter** - Class in uk.ac.ed.inf.enzml.mulan.learn.traintest: Using Mulan (multi-label machine learning), this class initialises and executes Train-Test evaluation (two arff records are needed, for the training and testing files) (calling `MulanTrainTestEvaluator`). **TrainTestExperimenter(String, String, int, int, MulanLearner, String)** - Constructor for class uk.ac.ed.inf.enzml.mulan.learn.traintest.TrainTestExperimenter: **TrainTestExperimenterSerialized** - Class in uk.ac.ed.inf.enzml.mulan.learn.traintest: Executes a train/test evaluation using a previously trained model saved on file. **TrainTestExperimenterSerialized(String, String, int, int, MulanLearner, String, String)** - Constructor for class uk.ac.ed.inf.enzml.mulan.learn.traintest.TrainTestExperimenterSerialized: **TrainTestExperimenterTest** - Class in test.mulan.learn.traintest: **TrainTestExperimenterTest()** - Constructor for class test.mulan.learn.traintest.TrainTestExperimenterTest: **TrainTestFullRun** - Class in uk.ac.ed.inf.enzml.mulan.learn.traintest: Runs a full train/test evaluation: 1. **TrainTestFullRun()** - Constructor for class uk.ac.ed.inf.enzml.mulan.learn.traintest.TrainTestFullRun: **trainTestValidate()** - Method in class uk.ac.ed.inf.enzml.mulan.learn.traintest.TrainTestEvaluator: Evaluates a `MultiLabelLearner` via train/test-validation

---


|  |  |  |  |  |  |  |  |  |  |  |
| --- | --- | --- | --- | --- | --- | --- | --- | --- | --- | --- |
| |  |  |  |  |  |  |  |  | | --- | --- | --- | --- | --- | --- | --- | --- | | **Overview** | Package | Class | Use | **Tree** | **Deprecated** | **Index** | **Help** | | |  |
| **PREV LETTER**   **NEXT LETTER** | **FRAMES**    **NO FRAMES**     **All Classes** |


A B C D E F G I K L M N P R S T U V W X 

---
