# Supplementary material for: EnzML: multi-label prediction of enzyme classes using InterPro signatures
Source: BMC Bioinformatics. 2012 Apr 25;13:61. doi: 10.1186/1471-2105-13-61 (PMC3483700; doi:10.1186/1471-2105-13-61)
Supplement: Addtional file 5 — The Java code to format the data files, evaluate and predict. The file enzml_java_code.tar.gz contains the Java code used to format database data to ARFF and XML formats, to execute cross and train-test (jackknife) evaluations and to record evaluation results to database. More information is included in the readme.txt file and the Javadoc files. The code can be used with a MySQL database. To use a different database software, other JDBC drivers might be required. [file 1471-2105-13-61-S5.gz › java_code/enzml2011/doc/index-files/index-8.html]

I-Index


---


|  |  |  |  |  |  |  |  |  |  |  |
| --- | --- | --- | --- | --- | --- | --- | --- | --- | --- | --- |
| |  |  |  |  |  |  |  |  | | --- | --- | --- | --- | --- | --- | --- | --- | | **Overview** | Package | Class | Use | **Tree** | **Deprecated** | **Index** | **Help** | | |  |
| **PREV LETTER**   **NEXT LETTER** | **FRAMES**    **NO FRAMES**     **All Classes** |


A B C D E F G I K L M N P R S T U V W X 

---


## **I**

**IdentifiedSparseInstance** - Class in uk.ac.ed.inf.enzml.weka: Class **IdentifiedSparseInstance()** - Constructor for class uk.ac.ed.inf.enzml.weka.IdentifiedSparseInstance: **initialisationIsCorrect()** - Method in class uk.ac.ed.inf.enzml.weka.DataSetManager: **initialise(String)** - Method in class test.dataharness.CreateDataTable: **INST1** - Static variable in class test.dataharness.DataOne: **INST1** - Static variable in class test.dataharness.DataTwo: **INST2** - Static variable in class test.dataharness.DataOne: **INST2** - Static variable in class test.dataharness.DataTwo: **INST3** - Static variable in class test.dataharness.DataOne: **INST3** - Static variable in class test.dataharness.DataTwo: **INST4** - Static variable in class test.dataharness.DataOne: **INST4** - Static variable in class test.dataharness.DataTwo: **INSTANCE\_1\_2** - Static variable in class test.dataharness.TestProjectParameters: **INSTANCE\_1\_2\_EMPTY** - Static variable in class test.dataharness.TestProjectParameters: **INSTANCE\_3** - Static variable in class test.dataharness.TestProjectParameters: **INSTANCE\_ATTRIBUTE\_QUERY** - Static variable in class test.dataharness.ArffPropsQueriesOneTest: **INSTANCE\_ATTRIBUTE\_QUERY** - Static variable in class test.dataharness.ArffPropsQueriesTwoTest: **INSTANCE\_ATTRIBUTE\_QUERY\_PROP** - Static variable in class uk.ac.ed.inf.enzml.weka.ArffProperties: The query to get the attributes for each instance **INSTANCE\_CLASS\_QUERY** - Static variable in class test.dataharness.ArffPropsQueriesOneTest: **INSTANCE\_CLASS\_QUERY** - Static variable in class test.dataharness.ArffPropsQueriesTwoTest: **INSTANCE\_CLASS\_QUERY\_PROP** - Static variable in class uk.ac.ed.inf.enzml.weka.ArffProperties: The query to get the class value for each instance **INSTANCE\_FIELD** - Static variable in class test.dataharness.CreateDataTable: **INSTANCE\_ID** - Static variable in class uk.ac.ed.inf.enzml.mulan.predict.MulanPredict: **INSTANCE\_NAME** - Static variable in class uk.ac.ed.inf.enzml.mulan.predict.MulanPredict: **instancesColumn()** - Static method in class test.dataharness.DataOne: **instancesFactory()** - Static method in class test.mulan.MulanInstancesFillerTest: **instancesFactory()** - Static method in class test.weka.InstancesFillerTest: **InstancesFiller** - Class in uk.ac.ed.inf.enzml.weka: Generates and fills individual instances for a weka data set. **InstancesFiller(DataSetGenerator)** - Constructor for class uk.ac.ed.inf.enzml.weka.InstancesFiller: **InstancesFillerTest** - Class in test.weka: Class **InstancesFillerTest()** - Constructor for class test.weka.InstancesFillerTest: **instancesValues()** - Static method in class test.dataharness.DataOne: Results of a select distinct instance, class query **InstanceUtils** - Class in uk.ac.ed.inf.enzml.mulan: Class **InstanceUtils()** - Constructor for class uk.ac.ed.inf.enzml.mulan.InstanceUtils: **INSTATTVOID** - Static variable in class test.dataharness.DataOne: **INSTCLASSATTVOID** - Static variable in class test.dataharness.DataOne: **INSTCLASSVOID** - Static variable in class test.dataharness.DataOne: **internallyCrossValidate(int, int)** - Method in class uk.ac.ed.inf.enzml.mulan.learn.MulanCrossEvaluator: Evaluates a `MultiLabelLearner` via cross-validation on given data set with defined number of folds.

---


|  |  |  |  |  |  |  |  |  |  |  |
| --- | --- | --- | --- | --- | --- | --- | --- | --- | --- | --- |
| |  |  |  |  |  |  |  |  | | --- | --- | --- | --- | --- | --- | --- | --- | | **Overview** | Package | Class | Use | **Tree** | **Deprecated** | **Index** | **Help** | | |  |
| **PREV LETTER**   **NEXT LETTER** | **FRAMES**    **NO FRAMES**     **All Classes** |


A B C D E F G I K L M N P R S T U V W X 

---
