# Supplementary material for: EnzML: multi-label prediction of enzyme classes using InterPro signatures
Source: BMC Bioinformatics. 2012 Apr 25;13:61. doi: 10.1186/1471-2105-13-61 (PMC3483700; doi:10.1186/1471-2105-13-61)
Supplement: Addtional file 5 — The Java code to format the data files, evaluate and predict. The file enzml_java_code.tar.gz contains the Java code used to format database data to ARFF and XML formats, to execute cross and train-test (jackknife) evaluations and to record evaluation results to database. More information is included in the readme.txt file and the Javadoc files. The code can be used with a MySQL database. To use a different database software, other JDBC drivers might be required. [file 1471-2105-13-61-S5.gz › java_code/enzml2011/doc/index-files/index-15.html]

S-Index


---


|  |  |  |  |  |  |  |  |  |  |  |
| --- | --- | --- | --- | --- | --- | --- | --- | --- | --- | --- |
| |  |  |  |  |  |  |  |  | | --- | --- | --- | --- | --- | --- | --- | --- | | **Overview** | Package | Class | Use | **Tree** | **Deprecated** | **Index** | **Help** | | |  |
| **PREV LETTER**   **NEXT LETTER** | **FRAMES**    **NO FRAMES**     **All Classes** |


A B C D E F G I K L M N P R S T U V W X 

---


## **S**

**savePredictions()** - Method in class uk.ac.ed.inf.enzml.mulan.predict.MulanPredict: **saveResults(int, int, int, String, String, MulanLearner, Evaluation)** - Method in class uk.ac.ed.inf.enzml.mulan.learn.ResultsSaver: **saveStatisticsAsProperties(String, String, String)** - Method in class uk.ac.ed.inf.enzml.weka.ArffProperties: **serialize(MultiLabelLearnerBase)** - Method in class uk.ac.ed.inf.enzml.mulan.learn.MulanSerializer: **SerializerTest** - Class in test.mulan.learn: Class **SerializerTest()** - Constructor for class test.mulan.learn.SerializerTest: **setArffRecordId(int)** - Method in class uk.ac.ed.inf.enzml.weka.Arff: Sets the arff record id and returns it for further use. **setDataSetManager()** - Method in class uk.ac.ed.inf.enzml.mulan.attributesfilter.AttributesFilteredArff: **setDataSetManager()** - Method in class uk.ac.ed.inf.enzml.mulan.MulanArff: **setDataSetManager()** - Method in class uk.ac.ed.inf.enzml.weka.Arff: **setEndTime(String)** - Method in class uk.ac.ed.inf.enzml.mulan.learn.MulanCrossEvaluator: **setEvaluationMeasures(Vector<TableRow>)** - Method in class uk.ac.ed.inf.enzml.mulan.learn.MulanCrossEvaluator: **setFileForSerializedModel()** - Method in class uk.ac.ed.inf.enzml.mulan.learn.MulanSerializer: **setInstancesNames()** - Method in class uk.ac.ed.inf.enzml.weka.DataSetGenerator: **setLogFile()** - Method in class uk.ac.ed.inf.enzml.mulan.learn.MulanSerializer: **setPredictionsAndLogFile(String)** - Method in class uk.ac.ed.inf.enzml.mulan.predict.MulanPredict: **setResultsDbManager(DbManager)** - Method in class uk.ac.ed.inf.enzml.mulan.learn.ResultsSaver: **setResultsSaver()** - Method in class uk.ac.ed.inf.enzml.mulan.learn.MulanCrossExperimenter: **setTableName(String)** - Method in class test.dataharness.CreateDataTable: **setTableName(String)** - Method in class test.dataharness.DataTableOneTest: **setTableName()** - Method in class test.dataharness.DataTableThreeTest: **setTableName()** - Method in class test.dataharness.DataTableTwoTest: **setTrainedModelFile(String)** - Method in class uk.ac.ed.inf.enzml.mulan.learn.traintest.TrainTestExperimenterSerialized: **setUp()** - Method in class test.mulan.learn.CrossEvaluatorTest: **setUp()** - Method in class test.mulan.learn.database.MulanDbCreatorTest: **setUp()** - Method in class test.mulan.learn.database.MulanDbManagerTest: **setUp()** - Method in class test.mulan.learn.database.MulanDbReaderTest: **setUp()** - Method in class test.mulan.learn.database.MulanDbWriterTest: **setUp()** - Method in class test.mulan.learn.EvaluationParametersTest: **setUp()** - Method in class test.mulan.learn.ExperimenterTest: **setUp()** - Method in class test.mulan.learn.ExperimentTableTest: **setUp()** - Method in class test.mulan.learn.LearnerTest: **setUp()** - Method in class test.mulan.learn.ResultsFormatterTest: **setUp()** - Method in class test.mulan.learn.ResultsSaverTest: **setUp()** - Method in class test.mulan.learn.SerializerTest: **setUp()** - Method in class test.mulan.MulanDataSetDbLoaderTest: **setUp()** - Method in class test.weka.ArffPropsTableManagerTest: **setUp()** - Method in class test.weka.ArffPropsTableReaderTest: **setUp()** - Method in class test.weka.ArffPropsTableTest: **setUp()** - Method in class test.weka.ArffTest: **setUp()** - Method in class test.weka.DataSetCheckerTest: **setUp()** - Method in class test.weka.DataSetGeneratorTest: **setUp()** - Method in class test.weka.DataSetManagerTest: **setUp()** - Method in class test.weka.DataSetWriterTest: **SOFTWARE\_PROJECT\_NAME** - Static variable in class uk.ac.ed.inf.enzml.ProjectParameters: **START\_TIMESTAMP** - Static variable in class uk.ac.ed.inf.enzml.mulan.learn.ExperimentTable: time at beginning of computation **statisticsKeys()** - Method in class uk.ac.ed.inf.enzml.weka.ArffProperties: The data statistics properties (number of attributes, classes, instances) added after the arff file generation **statsPropertiesAreCorrect(Properties)** - Method in class uk.ac.ed.inf.enzml.weka.ArffProperties: **suite()** - Static method in class test.AllArffTests: **suite()** - Static method in class test.AllMulanArffTests: **suite()** - Static method in class test.AllMulanLearningTests: **suite()** - Static method in class test.AllMulanPredictionTests: **suite()** - Static method in class test.AllMulanTests: **suite()** - Static method in class test.AllPreliminaryTests: **suite()** - Static method in class test.AllTests: **suite()** - Static method in class test.AllTrainTestsTests: **suite()** - Static method in class test.AllUtilsTests: **suite()** - Static method in class test.dataharness.AllDataTests

---


|  |  |  |  |  |  |  |  |  |  |  |
| --- | --- | --- | --- | --- | --- | --- | --- | --- | --- | --- |
| |  |  |  |  |  |  |  |  | | --- | --- | --- | --- | --- | --- | --- | --- | | **Overview** | Package | Class | Use | **Tree** | **Deprecated** | **Index** | **Help** | | |  |
| **PREV LETTER**   **NEXT LETTER** | **FRAMES**    **NO FRAMES**     **All Classes** |


A B C D E F G I K L M N P R S T U V W X 

---
