# Supplementary material for: EnzML: multi-label prediction of enzyme classes using InterPro signatures
Source: BMC Bioinformatics. 2012 Apr 25;13:61. doi: 10.1186/1471-2105-13-61 (PMC3483700; doi:10.1186/1471-2105-13-61)
Supplement: Addtional file 5 — The Java code to format the data files, evaluate and predict. The file enzml_java_code.tar.gz contains the Java code used to format database data to ARFF and XML formats, to execute cross and train-test (jackknife) evaluations and to record evaluation results to database. More information is included in the readme.txt file and the Javadoc files. The code can be used with a MySQL database. To use a different database software, other JDBC drivers might be required. [file 1471-2105-13-61-S5.gz › java_code/enzml2011/doc/index-files/index-4.html]

D-Index


---


|  |  |  |  |  |  |  |  |  |  |  |
| --- | --- | --- | --- | --- | --- | --- | --- | --- | --- | --- |
| |  |  |  |  |  |  |  |  | | --- | --- | --- | --- | --- | --- | --- | --- | | **Overview** | Package | Class | Use | **Tree** | **Deprecated** | **Index** | **Help** | | |  |
| **PREV LETTER**   **NEXT LETTER** | **FRAMES**    **NO FRAMES**     **All Classes** |


A B C D E F G I K L M N P R S T U V W X 

---


## **D**

**DATA\_PROPERTIES\_FILE\_PATH\_PROP** - Static variable in class uk.ac.ed.inf.enzml.weka.ArffProperties: the path to the properties file itself, from which the other properties were read **DatabaseTest** - Class in test.dataharness: Check the test database **DatabaseTest()** - Constructor for class test.dataharness.DatabaseTest: **dataMapsAreCorrect(OneToManyMap, OneToManyMap)** - Method in class uk.ac.ed.inf.enzml.weka.DataSetChecker: public boolean initialisationIsCorrect() { return m\_initialisationIsCorrect; } public void setInitialisation(boolean isCorrect) { m\_initialisationIsCorrect = isCorrect; } **DataOne** - Class in test.dataharness: Class **DataOne()** - Constructor for class test.dataharness.DataOne: **DATASET\_NAME** - Static variable in class test.dataharness.DataOne: **DATASET\_NAME** - Static variable in class test.dataharness.DataTwo: **DATASET\_NAME\_PROP** - Static variable in class uk.ac.ed.inf.enzml.weka.ArffProperties: the experiment short name **DataSetChecker** - Class in uk.ac.ed.inf.enzml.weka: Checks that all data (or file paths) needed to build the ARFF file is available **DataSetChecker(DataSetManager)** - Constructor for class uk.ac.ed.inf.enzml.weka.DataSetChecker: **DataSetCheckerTest** - Class in test.weka: Class **DataSetCheckerTest()** - Constructor for class test.weka.DataSetCheckerTest: **DataSetDbLoader** - Class in uk.ac.ed.inf.enzml.weka: Loads data (instances, attribute values, class values) from database using the queries in the data Properties. **DataSetDbLoader(DataSetManager)** - Constructor for class uk.ac.ed.inf.enzml.weka.DataSetDbLoader: **DataSetDbLoaderTest** - Class in test.weka: Class **DataSetDbLoaderTest()** - Constructor for class test.weka.DataSetDbLoaderTest: **DataSetGenerator** - Class in uk.ac.ed.inf.enzml.weka: Generates and fills the Weka instances. **DataSetGenerator(DataSetManager, OneToManyMap<String, String>, OneToManyMap<String, String>)** - Constructor for class uk.ac.ed.inf.enzml.weka.DataSetGenerator: **DataSetGeneratorTest** - Class in test.weka: Class **DataSetGeneratorTest()** - Constructor for class test.weka.DataSetGeneratorTest: **DataSetManager** - Class in uk.ac.ed.inf.enzml.weka: DataSet generates a collection of Weka instances (rows of the ARFF file), their attributes (column names) and their attribute values (cell values) The last attribute is the class attribute. **DataSetManager(Arff)** - Constructor for class uk.ac.ed.inf.enzml.weka.DataSetManager: **DataSetManagerTest** - Class in test.weka: Class **DataSetManagerTest()** - Constructor for class test.weka.DataSetManagerTest: **DataSetWriter** - Class in uk.ac.ed.inf.enzml.weka: Class **DataSetWriter(DataSetManager)** - Constructor for class uk.ac.ed.inf.enzml.weka.DataSetWriter: **DataSetWriterTest** - Class in test.weka: Class **DataSetWriterTest()** - Constructor for class test.weka.DataSetWriterTest: **dataStatistics()** - Method in class uk.ac.ed.inf.enzml.weka.DataSetWriter: **DataTableOneTest** - Class in test.dataharness: Basic data table containing 3 columns with: instances, attributes, classes. **DataTableOneTest()** - Constructor for class test.dataharness.DataTableOneTest: **DataTableThreeTest** - Class in test.dataharness: **DataTableThreeTest()** - Constructor for class test.dataharness.DataTableThreeTest: **DataTableTwoTest** - Class in test.dataharness: **DataTableTwoTest()** - Constructor for class test.dataharness.DataTableTwoTest: **DataTwo** - Class in test.dataharness: Class **DataTwo()** - Constructor for class test.dataharness.DataTwo: **deriveXmlPathFromArff(String)** - Method in class uk.ac.ed.inf.enzml.mulan.MulanArffRecord: **DESCRIPTION\_PROP** - Static variable in class uk.ac.ed.inf.enzml.weka.ArffProperties: **deserialize(String)** - Static method in class uk.ac.ed.inf.enzml.mulan.learn.MulanSerializer: Get trained learner from file **DUMMY\_VALUE** - Static variable in class uk.ac.ed.inf.enzml.weka.DataSetManager

---


|  |  |  |  |  |  |  |  |  |  |  |
| --- | --- | --- | --- | --- | --- | --- | --- | --- | --- | --- |
| |  |  |  |  |  |  |  |  | | --- | --- | --- | --- | --- | --- | --- | --- | | **Overview** | Package | Class | Use | **Tree** | **Deprecated** | **Index** | **Help** | | |  |
| **PREV LETTER**   **NEXT LETTER** | **FRAMES**    **NO FRAMES**     **All Classes** |


A B C D E F G I K L M N P R S T U V W X 

---
