# Supplementary material for: EnzML: multi-label prediction of enzyme classes using InterPro signatures
Source: BMC Bioinformatics. 2012 Apr 25;13:61. doi: 10.1186/1471-2105-13-61 (PMC3483700; doi:10.1186/1471-2105-13-61)
Supplement: Addtional file 5 — The Java code to format the data files, evaluate and predict. The file enzml_java_code.tar.gz contains the Java code used to format database data to ARFF and XML formats, to execute cross and train-test (jackknife) evaluations and to record evaluation results to database. More information is included in the readme.txt file and the Javadoc files. The code can be used with a MySQL database. To use a different database software, other JDBC drivers might be required. [file 1471-2105-13-61-S5.gz › java_code/enzml2011/doc/index-files/index-5.html]

E-Index


---


|  |  |  |  |  |  |  |  |  |  |  |
| --- | --- | --- | --- | --- | --- | --- | --- | --- | --- | --- |
| |  |  |  |  |  |  |  |  | | --- | --- | --- | --- | --- | --- | --- | --- | | **Overview** | Package | Class | Use | **Tree** | **Deprecated** | **Index** | **Help** | | |  |
| **PREV LETTER**   **NEXT LETTER** | **FRAMES**    **NO FRAMES**     **All Classes** |


A B C D E F G I K L M N P R S T U V W X 

---


## **E**

**END\_TIMESTAMP** - Static variable in class uk.ac.ed.inf.enzml.mulan.learn.ExperimentTable: time at end of computation **evaluate(MultiLabelLearner, MultiLabelInstances)** - Method in class uk.ac.ed.inf.enzml.mulan.learn.EvaluatorNoAUC: Evaluates a `MultiLabelLearner` on given test data set. **evaluate(int)** - Method in class uk.ac.ed.inf.enzml.mulan.learn.MulanCrossEvaluator: Evaluates (runs a cross validation) **evaluateAndSave()** - Method in class uk.ac.ed.inf.enzml.mulan.learn.traintest.TrainTestExperimenter: **EvaluationMetricsTest** - Class in test.mulan.learn.traintest: Tests the evaluation metrics results: accuracy, precision, recall etc. **EvaluationMetricsTest()** - Constructor for class test.mulan.learn.traintest.EvaluationMetricsTest: **EvaluationParameters** - Class in uk.ac.ed.inf.enzml.mulan.learn: Contains the evaluation parameters (micro-macro precision, micro-macro accuracy etc.) of a Multi label Mulan experiment. **EvaluationParameters()** - Constructor for class uk.ac.ed.inf.enzml.mulan.learn.EvaluationParameters: **EvaluationParametersTest** - Class in test.mulan.learn: Class **EvaluationParametersTest()** - Constructor for class test.mulan.learn.EvaluationParametersTest: **EvaluatorNoAUC** - Class in uk.ac.ed.inf.enzml.mulan.learn: Prepares the evaluation measures taking into consideration the dataset size. **EvaluatorNoAUC()** - Constructor for class uk.ac.ed.inf.enzml.mulan.learn.EvaluatorNoAUC: **ExperimenterTest** - Class in test.mulan.learn: Class **ExperimenterTest()** - Constructor for class test.mulan.learn.ExperimenterTest: **EXPERIMENTS\_TABLE\_NAME** - Static variable in class uk.ac.ed.inf.enzml.mulan.learn.ExperimentTable: the name for the table to store the evaluation results **ExperimentTable** - Class in uk.ac.ed.inf.enzml.mulan.learn: Gives the names and table field names for multi-label specific evaluation measures (such as micro-accuracy, macro-accuracy, hamming loss etc.) **ExperimentTable()** - Constructor for class uk.ac.ed.inf.enzml.mulan.learn.ExperimentTable: **ExperimentTableTest** - Class in test.mulan.learn: Class **ExperimentTableTest()** - Constructor for class test.mulan.learn.ExperimentTableTest

---


|  |  |  |  |  |  |  |  |  |  |  |
| --- | --- | --- | --- | --- | --- | --- | --- | --- | --- | --- |
| |  |  |  |  |  |  |  |  | | --- | --- | --- | --- | --- | --- | --- | --- | | **Overview** | Package | Class | Use | **Tree** | **Deprecated** | **Index** | **Help** | | |  |
| **PREV LETTER**   **NEXT LETTER** | **FRAMES**    **NO FRAMES**     **All Classes** |


A B C D E F G I K L M N P R S T U V W X 

---
