# Supplementary material for: EnzML: multi-label prediction of enzyme classes using InterPro signatures
Source: BMC Bioinformatics. 2012 Apr 25;13:61. doi: 10.1186/1471-2105-13-61 (PMC3483700; doi:10.1186/1471-2105-13-61)
Supplement: Addtional file 5 — The Java code to format the data files, evaluate and predict. The file enzml_java_code.tar.gz contains the Java code used to format database data to ARFF and XML formats, to execute cross and train-test (jackknife) evaluations and to record evaluation results to database. More information is included in the readme.txt file and the Javadoc files. The code can be used with a MySQL database. To use a different database software, other JDBC drivers might be required. [file 1471-2105-13-61-S5.gz › java_code/enzml2011/doc/index-files/index-19.html]

W-Index


---


|  |  |  |  |  |  |  |  |  |  |  |
| --- | --- | --- | --- | --- | --- | --- | --- | --- | --- | --- |
| |  |  |  |  |  |  |  |  | | --- | --- | --- | --- | --- | --- | --- | --- | | **Overview** | Package | Class | Use | **Tree** | **Deprecated** | **Index** | **Help** | | |  |
| **PREV LETTER**   **NEXT LETTER** | **FRAMES**    **NO FRAMES**     **All Classes** |


A B C D E F G I K L M N P R S T U V W X 

---


## **W**

**WEB\_PAGE** - Static variable in class uk.ac.ed.inf.enzml.ProjectParameters: **WekaTest** - Class in test.weka: Class to test basic weka instance creation **WekaTest()** - Constructor for class test.weka.WekaTest: **writeArff()** - Method in class uk.ac.ed.inf.enzml.weka.DataSetManager: **writeArffRecordToDb()** - Method in class uk.ac.ed.inf.enzml.weka.ArffPropsTableManager: **writeAttributesAndClasses(Instances)** - Method in class uk.ac.ed.inf.enzml.weka.DataSetWriter: Write the data instances to file **writeDataStatistics()** - Method in class uk.ac.ed.inf.enzml.weka.DataSetWriter: Logs to file the number of instances, classes and attributes in the dataset. **writeHeader()** - Method in class uk.ac.ed.inf.enzml.weka.DataSetWriter: **writeInstanceToFile(String, Instance)** - Method in class uk.ac.ed.inf.enzml.weka.DataSetWriter: Write an individual Weka instance to file **writeTail()** - Method in class uk.ac.ed.inf.enzml.weka.DataSetWriter

---


|  |  |  |  |  |  |  |  |  |  |  |
| --- | --- | --- | --- | --- | --- | --- | --- | --- | --- | --- |
| |  |  |  |  |  |  |  |  | | --- | --- | --- | --- | --- | --- | --- | --- | | **Overview** | Package | Class | Use | **Tree** | **Deprecated** | **Index** | **Help** | | |  |
| **PREV LETTER**   **NEXT LETTER** | **FRAMES**    **NO FRAMES**     **All Classes** |


A B C D E F G I K L M N P R S T U V W X 

---
