# Supplementary material for: EnzML: multi-label prediction of enzyme classes using InterPro signatures
Source: BMC Bioinformatics. 2012 Apr 25;13:61. doi: 10.1186/1471-2105-13-61 (PMC3483700; doi:10.1186/1471-2105-13-61)
Supplement: Addtional file 5 — The Java code to format the data files, evaluate and predict. The file enzml_java_code.tar.gz contains the Java code used to format database data to ARFF and XML formats, to execute cross and train-test (jackknife) evaluations and to record evaluation results to database. More information is included in the readme.txt file and the Javadoc files. The code can be used with a MySQL database. To use a different database software, other JDBC drivers might be required. [file 1471-2105-13-61-S5.gz › java_code/utils/doc/test/XmlNodeTest.html]

XmlNodeTest


---


|  |  |  |  |  |  |  |  |  |  |  |
| --- | --- | --- | --- | --- | --- | --- | --- | --- | --- | --- |
| |  |  |  |  |  |  |  |  | | --- | --- | --- | --- | --- | --- | --- | --- | | **Overview** | **Package** | **Class** | **Use** | **Tree** | **Deprecated** | **Index** | **Help** | | |  |
| **PREV CLASS**   **NEXT CLASS** | **FRAMES**    **NO FRAMES**     **All Classes** |
| SUMMARY: NESTED | FIELD | CONSTR | METHOD | DETAIL: FIELD | CONSTR | METHOD |


---


## test Class XmlNodeTest

```
java.lang.Object
  junit.framework.Assert
      junit.framework.TestCase
          test.XmlNodeTest
```

**All Implemented Interfaces:**: junit.framework.Test

---

``` public class XmlNodeTest extends junit.framework.TestCase ```

Class

**Version:**
:   18 Nov 2008

**Author:**
:   Luna De Ferrari luna.deferrari-at-ed.ac.uk

---

| **Constructor Summary** | |
| --- | --- |
| `XmlNodeTest()` |


| **Method Summary** | |
| --- | --- |
| `static XmlNode` | `getNumberedXmlTree()` |
| `static XmlNode` | `getPdbSimpleElement()` |
| `static java.lang.String` | `getXml1String()` |
| `static java.lang.String` | `getXml2String()` |
| `static java.lang.String` | `getXml3String()` |
| `static java.lang.String` | `getXml4String()` |
| `static java.lang.String` | `getXml5String()` |
| `static java.lang.String` | `getXml6String()` |
| `static java.lang.String` | `getXml7String()` |
| `static java.lang.String` | `getXmlString()` |
| `void` | `setUp()` |
| `void` | `testAddChildElement()` |
| `void` | `testNodeHeadToString()` |
| `void` | `testSimpleElement()` |
| `void` | `testToString()` |

| **Methods inherited from class junit.framework.TestCase** |
| --- |
| `countTestCases, getName, run, run, runBare, setName, toString` |

| **Methods inherited from class junit.framework.Assert** |
| --- |
| `assertEquals, assertEquals, assertEquals, assertEquals, assertEquals, assertEquals, assertEquals, assertEquals, assertEquals, assertEquals, assertEquals, assertEquals, assertEquals, assertEquals, assertEquals, assertEquals, assertEquals, assertEquals, assertEquals, assertEquals, assertFalse, assertFalse, assertNotNull, assertNotNull, assertNotSame, assertNotSame, assertNull, assertNull, assertSame, assertSame, assertTrue, assertTrue, fail, fail, failNotEquals, failNotSame, failSame, format` |

| **Methods inherited from class java.lang.Object** |
| --- |
| `equals, getClass, hashCode, notify, notifyAll, wait, wait, wait` |

| **Constructor Detail** |
| --- |

### XmlNodeTest

```
public XmlNodeTest()
```


| **Method Detail** |
| --- |

### setUp

```
public void setUp()
           throws java.lang.Exception
```

:   **Overrides:**: `setUp` in class `junit.framework.TestCase`
:   **Throws:**: `java.lang.Exception`

---


### testAddChildElement

```
public void testAddChildElement()
```

---


### testNodeHeadToString

```
public void testNodeHeadToString()
```

---


### testSimpleElement

```
public void testSimpleElement()
```

---


### testToString

```
public void testToString()
```

---


### getNumberedXmlTree

```
public static XmlNode getNumberedXmlTree()
```

---


### getPdbSimpleElement

```
public static XmlNode getPdbSimpleElement()
```

:   **Returns:**: an xml node containing PDB data

---


### getXml1String

```
public static java.lang.String getXml1String()
```

---


### getXml2String

```
public static java.lang.String getXml2String()
```

---


### getXml3String

```
public static java.lang.String getXml3String()
```

---


### getXml4String

```
public static java.lang.String getXml4String()
```

---


### getXml5String

```
public static java.lang.String getXml5String()
```

---


### getXml6String

```
public static java.lang.String getXml6String()
```

---


### getXml7String

```
public static java.lang.String getXml7String()
```

---


### getXmlString

```
public static java.lang.String getXmlString()
```


---


|  |  |  |  |  |  |  |  |  |  |  |
| --- | --- | --- | --- | --- | --- | --- | --- | --- | --- | --- |
| |  |  |  |  |  |  |  |  | | --- | --- | --- | --- | --- | --- | --- | --- | | **Overview** | **Package** | **Class** | **Use** | **Tree** | **Deprecated** | **Index** | **Help** | | |  |
| **PREV CLASS**   **NEXT CLASS** | **FRAMES**    **NO FRAMES**     **All Classes** |
| SUMMARY: NESTED | FIELD | CONSTR | METHOD | DETAIL: FIELD | CONSTR | METHOD |


---
