# Supplementary material for: EnzML: multi-label prediction of enzyme classes using InterPro signatures
Source: BMC Bioinformatics. 2012 Apr 25;13:61. doi: 10.1186/1471-2105-13-61 (PMC3483700; doi:10.1186/1471-2105-13-61)
Supplement: Addtional file 5 — The Java code to format the data files, evaluate and predict. The file enzml_java_code.tar.gz contains the Java code used to format database data to ARFF and XML formats, to execute cross and train-test (jackknife) evaluations and to record evaluation results to database. More information is included in the readme.txt file and the Javadoc files. The code can be used with a MySQL database. To use a different database software, other JDBC drivers might be required. [file 1471-2105-13-61-S5.gz › java_code/utils/doc/index-files/index-1.html]

A-Index


---


|  |  |  |  |  |  |  |  |  |  |  |
| --- | --- | --- | --- | --- | --- | --- | --- | --- | --- | --- |
| |  |  |  |  |  |  |  |  | | --- | --- | --- | --- | --- | --- | --- | --- | | **Overview** | Package | Class | Use | **Tree** | **Deprecated** | **Index** | **Help** | | |  |
| PREV LETTER   **NEXT LETTER** | **FRAMES**    **NO FRAMES**     **All Classes** |


A B C D E F G H I J K L M N O P Q R S T U V W X Y 

---


## **A**

**AbstractContinousDistribution** - Class in cern.jet.random: Abstract base class for all continous distributions. **AbstractDistribution** - Class in cern.jet.random: Abstract base class for all random distributions. **addAll(OneToManyMap<T, U>)** - Method in class uk.ac.ed.inf.utils.maputils.OneToManyMap: Add all elements of a one2many map to another map **addChildNode(XmlNode)** - Method in class uk.ac.ed.inf.utils.webutils.simpledomparser.XmlNode: Adds a child element to the node, and returns the node itself **addKeyAndValue(T, U)** - Method in class uk.ac.ed.inf.utils.maputils.OneToManyMap: Add a key-value pair to the map **addKeyValue(String, String)** - Method in class uk.ac.ed.inf.utils.maputils.TableMap: Add a key/value set to the one to many map **addStringToArray(String[], String)** - Static method in class uk.ac.ed.inf.utils.ListUtils: Takes an array and adds a string to all elements **addStringToArrayList(ArrayList, String)** - Static method in class uk.ac.ed.inf.utils.ListUtils: Takes an arrayList and adds a string to all elements **addSubset(Set)** - Method in class uk.ac.ed.inf.utils.setutils.Set: **AllDatabaseUtilsTests** - Class in test: Class **AllDatabaseUtilsTests()** - Constructor for class test.AllDatabaseUtilsTests: **AllStatsUtilsTests** - Class in uk.ac.ed.inf.utils.stats.tests: Class **AllStatsUtilsTests()** - Constructor for class uk.ac.ed.inf.utils.stats.tests.AllStatsUtilsTests: **AllUtilsTests** - Class in test: Class **AllUtilsTests()** - Constructor for class test.AllUtilsTests: **appendPropertiesToFile(File, Properties)** - Static method in class uk.ac.ed.inf.utils.FileUtils: **appendToFile(File, String)** - Static method in class uk.ac.ed.inf.utils.FileUtils: Append text to file **appendToFile(String, String)** - Static method in class uk.ac.ed.inf.utils.FileUtils: Append text to file **appendToFileAndPrint(String, String)** - Static method in class uk.ac.ed.inf.utils.FileUtils: Append print text to screen (system.out) and append it to file. **apply(double)** - Method in interface cern.colt.function.DoubleFunction: Applies a function to an argument. **apply(int)** - Method in interface cern.colt.function.IntFunction: Applies a function to an argument. **apply(double)** - Method in class cern.jet.random.AbstractDistribution: Equivalent to nextDouble(). **apply(int)** - Method in class cern.jet.random.AbstractDistribution: Equivalent to nextInt(). **apply(double)** - Method in class cern.jet.random.engine.RandomEngine: Equivalent to raw(). **apply(int)** - Method in class cern.jet.random.engine.RandomEngine: Equivalent to nextInt(). **arrayFrequencies(Integer[])** - Static method in class uk.ac.ed.inf.utils.stats.StatUtils: Records the freqencies with which the integers appear in the array **arrayIsEmpty(String[])** - Static method in class uk.ac.ed.inf.utils.ArrayUtils: Checks whether an array is empty **arrayList()** - Static method in class test.ListUtilsTest: **Deprecated.** *Use `ListUtilsTest.list()` instead* **ArrayUtils** - Class in uk.ac.ed.inf.utils: Utilities to manipulate arrays. **ArrayUtils()** - Constructor for class uk.ac.ed.inf.utils.ArrayUtils: **AUTO\_INCREMENT\_SQL\_DATATYPE** - Static variable in class uk.ac.ed.inf.utils.database.DbUtils

---


|  |  |  |  |  |  |  |  |  |  |  |
| --- | --- | --- | --- | --- | --- | --- | --- | --- | --- | --- |
| |  |  |  |  |  |  |  |  | | --- | --- | --- | --- | --- | --- | --- | --- | | **Overview** | Package | Class | Use | **Tree** | **Deprecated** | **Index** | **Help** | | |  |
| PREV LETTER   **NEXT LETTER** | **FRAMES**    **NO FRAMES**     **All Classes** |


A B C D E F G H I J K L M N O P Q R S T U V W X Y 

---
