# Supplementary material for: EnzML: multi-label prediction of enzyme classes using InterPro signatures
Source: BMC Bioinformatics. 2012 Apr 25;13:61. doi: 10.1186/1471-2105-13-61 (PMC3483700; doi:10.1186/1471-2105-13-61)
Supplement: Addtional file 5 — The Java code to format the data files, evaluate and predict. The file enzml_java_code.tar.gz contains the Java code used to format database data to ARFF and XML formats, to execute cross and train-test (jackknife) evaluations and to record evaluation results to database. More information is included in the readme.txt file and the Javadoc files. The code can be used with a MySQL database. To use a different database software, other JDBC drivers might be required. [file 1471-2105-13-61-S5.gz › java_code/utils/doc/index-files/index-13.html]

M-Index


---


|  |  |  |  |  |  |  |  |  |  |  |
| --- | --- | --- | --- | --- | --- | --- | --- | --- | --- | --- |
| |  |  |  |  |  |  |  |  | | --- | --- | --- | --- | --- | --- | --- | --- | | **Overview** | Package | Class | Use | **Tree** | **Deprecated** | **Index** | **Help** | | |  |
| **PREV LETTER**   **NEXT LETTER** | **FRAMES**    **NO FRAMES**     **All Classes** |


A B C D E F G H I J K L M N O P Q R S T U V W X Y 

---


## **M**

**m\_dbWriter** - Variable in class uk.ac.ed.inf.utils.database.DbManager: the database recorder **m\_debug** - Static variable in class uk.ac.ed.inf.utils.RegExpUtils: **m\_debug** - Static variable in class uk.ac.ed.inf.utils.setutils.SupersetsManager: **m\_debug** - Static variable in class uk.ac.ed.inf.utils.webutils.simpledomparser.XmlSearcher: **m\_debug** - Static variable in class uk.ac.ed.inf.utils.webutils.WebUtils: **m\_initialisationIsCorrect** - Variable in class uk.ac.ed.inf.utils.Initialised: **m\_map** - Variable in class uk.ac.ed.inf.utils.maputils.OneToManyMap: the map containing for each key the collection of values that key can have **m\_writer** - Variable in class uk.ac.ed.inf.utils.database.TableManager: the database recorder **main(String[])** - Static method in class edu.cornell.lassp.houle.RngPack.RandomApp: **main(String[])** - Static method in class test.AllDatabaseUtilsTests: **main(String[])** - Static method in class test.AllUtilsTests: **main(String[])** - Static method in class uk.ac.ed.inf.utils.database.DbUtils: Runs the 'analyze table ' command for all the tables in a mysql database **main(String[])** - Static method in class uk.ac.ed.inf.utils.EntrezUtils: Mains for testing **main(String[])** - Static method in class uk.ac.ed.inf.utils.FileUtils: Main method for testing **main(String[])** - Static method in class uk.ac.ed.inf.utils.guiutils.GuiUtils: For testing **main(String[])** - Static method in class uk.ac.ed.inf.utils.guiutils.SimpleRadioButtonPanel: For testing **main(String[])** - Static method in class uk.ac.ed.inf.utils.setutils.SupersetsManager: **main(String[])** - Static method in class uk.ac.ed.inf.utils.stats.tests.AllStatsUtilsTests: **main(String[])** - Static method in class uk.ac.ed.inf.utils.Utils: Main method for testing **makeDefault()** - Static method in class cern.jet.random.engine.RandomEngine: Constructs and returns a new uniform random number engine seeded with the current time. **makeDefaultGenerator()** - Static method in class cern.jet.random.AbstractDistribution: Constructs and returns a new uniform random number generation engine seeded with the current time. **Managed** - Class in uk.ac.ed.inf.utils.database: A class with a manager object **Managed(Manager)** - Constructor for class uk.ac.ed.inf.utils.database.Managed: **Manager** - Class in uk.ac.ed.inf.utils.database: A manager manages: for example, for a database object, the `DbManager` manages a `DbCreator` to create the tables, `DbReader` to read data from and `DbWriter` to write data to the database. **Manager()** - Constructor for class uk.ac.ed.inf.utils.database.Manager: **map()** - Static method in class test.maputils.TableMapTest: **mapIsNullOrEmpty()** - Method in class uk.ac.ed.inf.utils.maputils.OneToManyMap: public void initialise(Vector keys, Vector values) { this.setMap(); this.generateMap(keys, values); } **mapToString(Map)** - Static method in class uk.ac.ed.inf.utils.maputils.MapUtils: Creates a string representation of a map, as index,tab,key,tab,value **MapUtils** - Class in uk.ac.ed.inf.utils.maputils: Utilities to manipulate maps and hashmaps **MapUtils()** - Constructor for class uk.ac.ed.inf.utils.maputils.MapUtils: **MapUtilsTest** - Class in test.maputils: Class **MapUtilsTest()** - Constructor for class test.maputils.MapUtilsTest: **MAX\_BIGINT\_LENGTH** - Static variable in class uk.ac.ed.inf.utils.database.SqlUtils: Maximum length in characters for a signed SQL INT BIGINT[(M)] A large integer. **MAX\_CHAR\_LENGTH** - Static variable in class uk.ac.ed.inf.utils.database.SqlUtils: Maximum length in characters for an SQL CHAR column CHAR(M) A fixed-length string. **MAX\_INT\_LENGTH** - Static variable in class uk.ac.ed.inf.utils.database.SqlUtils: Maximum length in characters for a signed SQL INT INT[(M)] A normal-size integer. **maxlev** - Static variable in class edu.cornell.lassp.houle.RngPack.Ranlux: Maximum luxury level: `maxlev=4` **md5Hash(String)** - Static method in class uk.ac.ed.inf.utils.webutils.WebUtils: Create the md5 hash of a text From http://snippets.dzone.com/posts/show/3686 **MersenneTwister** - Class in cern.jet.random.engine: MersenneTwister (MT19937) is one of the strongest uniform pseudo-random number generators known so far; at the same time it is quick. **MersenneTwister()** - Constructor for class cern.jet.random.engine.MersenneTwister: Constructs and returns a random number generator with a default seed, which is a **constant**. **MersenneTwister(Date)** - Constructor for class cern.jet.random.engine.MersenneTwister: Constructs and returns a random number generator seeded with the given date. **MersenneTwister(int)** - Constructor for class cern.jet.random.engine.MersenneTwister: Constructs and returns a random number generator with the given seed. **messagePopUp(String, String)** - Static method in class uk.ac.ed.inf.utils.guiutils.GuiUtils: Pop-up window with message (icon: information > "i") **MILLISECONDS** - Static variable in class uk.ac.ed.inf.utils.TimeUtils: **MINUTE** - Static variable in class uk.ac.ed.inf.utils.TimeUtils: **MONTH** - Static variable in class uk.ac.ed.inf.utils.TimeUtils: **multiOptionsPopUp(String, String, String[])** - Static method in class uk.ac.ed.inf.utils.guiutils.GuiUtils: A multiple options popup.

---


|  |  |  |  |  |  |  |  |  |  |  |
| --- | --- | --- | --- | --- | --- | --- | --- | --- | --- | --- |
| |  |  |  |  |  |  |  |  | | --- | --- | --- | --- | --- | --- | --- | --- | | **Overview** | Package | Class | Use | **Tree** | **Deprecated** | **Index** | **Help** | | |  |
| **PREV LETTER**   **NEXT LETTER** | **FRAMES**    **NO FRAMES**     **All Classes** |


A B C D E F G H I J K L M N O P Q R S T U V W X Y 

---
