# Supplementary material for: EnzML: multi-label prediction of enzyme classes using InterPro signatures
Source: BMC Bioinformatics. 2012 Apr 25;13:61. doi: 10.1186/1471-2105-13-61 (PMC3483700; doi:10.1186/1471-2105-13-61)
Supplement: Addtional file 5 — The Java code to format the data files, evaluate and predict. The file enzml_java_code.tar.gz contains the Java code used to format database data to ARFF and XML formats, to execute cross and train-test (jackknife) evaluations and to record evaluation results to database. More information is included in the readme.txt file and the Javadoc files. The code can be used with a MySQL database. To use a different database software, other JDBC drivers might be required. [file 1471-2105-13-61-S5.gz › java_code/utils/doc/index-files/index-20.html]

T-Index


---


|  |  |  |  |  |  |  |  |  |  |  |
| --- | --- | --- | --- | --- | --- | --- | --- | --- | --- | --- |
| |  |  |  |  |  |  |  |  | | --- | --- | --- | --- | --- | --- | --- | --- | | **Overview** | Package | Class | Use | **Tree** | **Deprecated** | **Index** | **Help** | | |  |
| **PREV LETTER**   **NEXT LETTER** | **FRAMES**    **NO FRAMES**     **All Classes** |


A B C D E F G H I J K L M N O P Q R S T U V W X Y 

---


## **T**

**Table** - Class in uk.ac.ed.inf.utils.database: An SQL database table definition (table name + columns) **Table(String, Vector<TableColumn>)** - Constructor for class uk.ac.ed.inf.utils.database.Table: List of primary key fields (starting from 0) **TABLE1** - Static variable in class test.database.TableCreatorTest: **TableColumn** - Class in uk.ac.ed.inf.utils.database: A column object contains all the information to create, fill and delete a database table column **TableColumn(String, String, boolean)** - Constructor for class uk.ac.ed.inf.utils.database.TableColumn: Constructor 2: takes the column name, data type and primary key status **TableCreator** - Class in uk.ac.ed.inf.utils.database: Class to create a table object in the database. **TableCreator(TableManager, Table)** - Constructor for class uk.ac.ed.inf.utils.database.TableCreator: **TableCreatorTest** - Class in test.database: Class **TableCreatorTest()** - Constructor for class test.database.TableCreatorTest: **tableExistsInDb(String)** - Method in class uk.ac.ed.inf.utils.database.DbReader: Checks if a table exists in the database **TableManaged** - Class in uk.ac.ed.inf.utils.database: TableManaged is a class which has a `TableManager` (an object that coordinates its table activities) **TableManaged(DbManager, TableManager)** - Constructor for class uk.ac.ed.inf.utils.database.TableManaged: **TableManager** - Class in uk.ac.ed.inf.utils.database: Class to create/read/write to a database table. **TableManager(DbManager, String)** - Constructor for class uk.ac.ed.inf.utils.database.TableManager: Constructor to create a generic manager to read/write to a table. **TableManager(DbManager, Table)** - Constructor for class uk.ac.ed.inf.utils.database.TableManager: **TableManagerTest** - Class in test.database: Class **TableManagerTest()** - Constructor for class test.database.TableManagerTest: **TableMap** - Class in uk.ac.ed.inf.utils.maputils: A table map is a map containing relations [key > list of values] [value > list of keys] **TableMap()** - Constructor for class uk.ac.ed.inf.utils.maputils.TableMap: Initialises the object with an empty 'one to many' map. **TableMap(Vector<String>, Vector<String>)** - Constructor for class uk.ac.ed.inf.utils.maputils.TableMap: Initialise with a list of key-value pairs **TableMapTest** - Class in test.maputils: Class **TableMapTest()** - Constructor for class test.maputils.TableMapTest: **TableReader** - Class in uk.ac.ed.inf.utils.database: Class **TableReader(TableManager)** - Constructor for class uk.ac.ed.inf.utils.database.TableReader: **TableReaderTest** - Class in test.database: Class **TableReaderTest()** - Constructor for class test.database.TableReaderTest: **TableRow** - Class in uk.ac.ed.inf.utils.database: Contains the data for a generic row (a list of [table column, value] entries) to be written to a database table. **TableRow(TableManager)** - Constructor for class uk.ac.ed.inf.utils.database.TableRow: **TableRow(TableManager, TreeMap<String, String>)** - Constructor for class uk.ac.ed.inf.utils.database.TableRow: the auto-generated key of this row in its table (if available) **TableRow(TableManager, Vector<String>, Vector<String>)** - Constructor for class uk.ac.ed.inf.utils.database.TableRow: **TableRowTest** - Class in test.database: Class **TableRowTest()** - Constructor for class test.database.TableRowTest: **TableTest** - Class in test.database: Class **TableTest()** - Constructor for class test.database.TableTest: **TableWriter** - Class in uk.ac.ed.inf.utils.database: A TableWriter object contains all the information to create, fill and delete a database table. **TableWriter(TableManager)** - Constructor for class uk.ac.ed.inf.utils.database.TableWriter: Constructor 2: takes the table name and a list of fields **TableWriterTest** - Class in test.database: Class **TableWriterTest()** - Constructor for class test.database.TableWriterTest: **tearDown()** - Method in class test.database.DbConnPropsTest: **tearDown()** - Method in class test.database.DbReaderTest: **test** - package test: **test.database** - package test.database: **test.maputils** - package test.maputils: **test.setutils** - package test.setutils: **TEST\_DATABASE\_CONNECTION** - Static variable in class test.database.DbManagerTest: **TEST\_DB\_CONN\_PATH** - Static variable in class uk.ac.ed.inf.utils.PathUtils: **TEST\_HOME** - Static variable in class uk.ac.ed.inf.utils.PathUtils: **testAddAll()** - Method in class test.maputils.IndexedOneToManyMapTest: **testAddAll()** - Method in class test.maputils.OneToManyMapTest: **testAddChildElement()** - Method in class test.XmlNodeTest: **testAddKeyValue()** - Method in class test.maputils.IndexedOneToManyMapTest: **testAddKeyValue()** - Method in class test.maputils.OneToManyMapTest: **testAddSubset()** - Method in class test.setutils.SetTest: **testAddValue()** - Method in class test.maputils.TableMapTest: **testAddValueToValues()** - Method in class test.maputils.IndexedOneToManyMapTest: **testAddValueToValues()** - Method in class test.maputils.OneToManyMapTest: **testCanWriteParent()** - Method in class test.FileUtilsTest: **testCheckRow()** - Method in class test.database.TableRowTest: **testCheckRowFields()** - Method in class test.database.TableRowTest: **testClass()** - Method in class test.UtilsTest: public void testBigDecimal(){ this.testBigDecimalGeneric(0.0, 1.0, 0.1); this.testBigDecimalGeneric(0.0, 1.0, 0.2); this.testBigDecimalGeneric(0.0, 1.0, 0.3); } public void testDoubleIncrements1(){ this.testDoubleIncrementsBigDecimal(0.0, 10.0, 0.1); this.testDoubleIncrementsBigDecimal(0.0, 1.0, 0.1); this.testDoubleIncrementsBigDecimal(0.0, 1.0, 0.2); this.testDoubleIncrementsBigDecimal(0.0, 1.0, 0.3); // is not well rounded this.testDoubleIncrementsBigDecimal(0.0, 1.7, 0.3); // increment is null Vector increments = this.testDoubleIncrementsBigDecimal(0.0, 10.0, 0.0); //assertNull(increments); } public Vector testDoubleIncrementsBigDecimal(Double min, Double max, Double percOfIncrement){ TreeSet basicIncrements = Utils.getDoubleIncrementsBigDecimal(min, max, percOfIncrement); if(basicIncrements != null){ Vector increments = new Vector(basicIncrements); Double range = max - min; Double increment = range \* percOfIncrement; if(increment > 0 || increment < 0){ System.out.println("min: " + min + ", max: " + max + ", increment: " + increment + " " + increments.toString()); Double noOfincrements = (range / increment) + 1; int numberOfIncrements = noOfincrements.intValue(); //assertEquals(numberOfIncrements, increments.size()); for(int i=0; i < numberOfIncrements; i++ ){ Double value = min + (i \* increment); //assertEquals(value, (Double) increments.get(i)); } }else{ //assertEquals(null, increments); } return increments; }else{ return null; } } public void testBigDecimalGeneric(Double minDouble, Double maxDouble, Double percentOfIncrementDouble){ BigDecimal min = BigDecimal.valueOf(minDouble); BigDecimal max = BigDecimal.valueOf(maxDouble); BigDecimal percentOfIncrement = BigDecimal.valueOf(percentOfIncrementDouble); Vector intervals = new Vector(); BigDecimal range = max.subtract(min); System.out.println("UtilsTest BigDecimal: " + min + " " + max + " " + percentOfIncrement + " " + range); System.out.println("zero " + BigDecimal.ZERO); assertEquals(1, range.compareTo(BigDecimal.ZERO)); BigDecimal increment = range.multiply(percentOfIncrement); System.out.println("range.divide(increment, RoundingMode.DOWN) " + range.divide(increment, RoundingMode.DOWN)); BigDecimal value = min; value = value.add(increment); System.out.println("value.add(increment) " + value); System.out.println("value.add(increment).ROUND\_DOWN " + value.add(increment).ROUND\_DOWN); System.out.println("max.ROUND\_DOWN " + max.ROUND\_DOWN); System.out.println("---------------------------------"); BigDecimal one0 = BigDecimal.valueOf(1.0); BigDecimal one1 = BigDecimal.valueOf(1.0); BigDecimal range0 = one0.subtract(one1); assertTrue(range0.compareTo(BigDecimal.ZERO) == 0); } **testColumnExistsInTable()** - Method in class test.database.TableReaderTest: **testConnection()** - Method in class test.database.DbManagerTest: **testConnection(Connection, String)** - Static method in class uk.ac.ed.inf.utils.database.DbUtils: Test Validity of a Connection. **testCountValues()** - Method in class test.CollectionUtilsTest: **testCountValues()** - Method in class test.maputils.MapUtilsTest: **testCreateTable()** - Method in class test.database.TableCreatorTest: **testDateConverter()** - Method in class test.TimeUtilsTest: **testDepthFirstSearchOfParent()** - Method in class test.XmlSearcherTest: **testDoubleFormat()** - Method in class test.NumberUtilsTest: **testDoubleIncrements2()** - Method in class test.UtilsTest: **testDropTable()** - Method in class test.database.TableCreatorTest: **testElapsedTime()** - Method in class test.TimeUtilsTest: **testExtractInteger()** - Method in class uk.ac.ed.inf.utils.stats.tests.UniformRandomUtilsTest: **testExtractUniformRandomMapEntry()** - Method in class uk.ac.ed.inf.utils.stats.tests.UniformRandomUtilsTest: **testFillMap()** - Method in class test.maputils.TableMapTest: **testFilterListsByRegExp()** - Method in class test.RegExpUtilsTest: **testFormatPercentage()** - Method in class test.NumberUtilsTest: **testFrequenciesToPercentOfFrequency()** - Method in class uk.ac.ed.inf.utils.stats.tests.StatUtilsTest: **testGetChildByTag()** - Method in class test.XmlSearcherTest: **testGetColumns()** - Method in class test.database.TableTest: **testGetConnection()** - Method in class test.database.DbConnPropsTest: **testGetConnectionLog()** - Method in class test.database.DbConnPropsTest: **testGetDatabaseName()** - Method in class test.database.DbConnPropsTest: **testGetDatabaseType()** - Method in class test.database.DbConnPropsTest: **testGetDriverName()** - Method in class test.database.DbConnPropsTest: **testGetFileNameFromDirectory()** - Method in class test.FileUtilsTest: **testGetFileNameFromDirectory2()** - Method in class test.FileUtilsTest: **testGetHost()** - Method in class test.database.DbConnPropsTest: **testGetInsertSql()** - Method in class test.database.TableWriterTest: **testGetInstance()** - Method in class uk.ac.ed.inf.utils.stats.tests.UniformRandomSingletonTest: **testGetKeys()** - Method in class test.maputils.TableMapTest: **testGetKeysOrValuesSet()** - Method in class test.maputils.IndexedOneToManyMapTest: **testGetKeysOrValuesSet()** - Method in class test.maputils.OneToManyMapTest: **testGetKeysVectorValuesVector()** - Method in class test.maputils.IndexedOneToManyMapTest: **testGetKeysVectorValuesVector()** - Method in class test.maputils.OneToManyMapTest: **testGetKeyValueNumber()** - Method in class test.maputils.TableMapTest: **testGetListOfDbTables()** - Method in class test.database.DbReaderTest: **testGetLoadInfileSql()** - Method in class test.database.TableWriterTest: **testGetMatchesBoundaries()** - Static method in class test.RegExpUtilsTest: **testGetNonEmptyKeysOrValues()** - Method in class test.maputils.IndexedOneToManyMapTest: **testGetNonEmptyKeysOrValues()** - Method in class test.maputils.OneToManyMapTest: **testGetParetoFrequencies()** - Method in class uk.ac.ed.inf.utils.stats.tests.TruncatedParetoTest: **testGetReaderFromURL()** - Method in class test.WebUtilsTest: **testGetSelectWhereEqualSql()** - Method in class test.database.SqlUtilsTest: **testGetSqlTimestamp()** - Method in class test.TimeUtilsTest: **testGetSqlToCreateTable()** - Method in class test.database.TableCreatorTest: **testGetSqlToDropTable()** - Method in class test.database.DbCreatorTest: **testGetStatement()** - Method in class test.database.DbConnPropsTest: **testGetTableFields()** - Method in class test.database.TableReaderTest: **testGetTableName()** - Method in class test.database.TableCreatorTest: **testGetTableName()** - Method in class test.database.TableTest: **testGetTidyDOM()** - Method in class test.XmlUtilsTest: **testGetUniformRandomSubmap()** - Method in class uk.ac.ed.inf.utils.stats.tests.UniformRandomUtilsTest: **testGetValues()** - Method in class test.maputils.TableMapTest: **testGetValuesForAKey()** - Method in class test.maputils.IndexedOneToManyMapTest: **testGetValuesForAKey()** - Method in class test.maputils.OneToManyMapTest: **testGetValuesForAKey()** - Method in class test.maputils.TableMapTest: **testGetXmlFromHTML()** - Method in class test.XmlUtilsTest: **testHasKey()** - Method in class test.database.TableTest: **testHistogram()** - Method in class uk.ac.ed.inf.utils.stats.tests.StatUtilsTest: **testHumanReadableTimestamp()** - Method in class test.TimeUtilsTest: **testIncrementsUtil()** - Method in class test.UtilsTest: **testIndexedMap()** - Method in class test.maputils.MapUtilsTest: **testInsert()** - Method in class test.database.DbWriterTest: **testIntegerIncrements(Integer, Integer, Double)** - Method in class test.UtilsTest: **testIsDash()** - Method in class test.StringUtilsTest: **testIsSubset()** - Method in class test.setutils.SetTest: **testJavaReflectionGetFields()** - Method in class test.ReflectionUtilsTest: only works for public fields **testJavaReflectionMethods()** - Method in class test.ReflectionUtilsTest: **testListIsNullOrEmpty()** - Method in class test.ListUtilsTest: **testManager()** - Method in class test.setutils.SupersetsManagerTest: **testManagerWithDatabaseData()** - Method in class test.setutils.SupersetsManagerTest: **testMapIsFilled()** - Method in class test.maputils.IndexedOneToManyMapTest: **testMapIsFilled()** - Method in class test.maputils.OneToManyMapTest: **testMapToString()** - Method in class test.maputils.MapUtilsTest: **testMd5Hash()** - Method in class test.WebUtilsTest: **testMillisecTimeStamp()** - Method in class test.TimeUtilsTest: **testMultilineMatch()** - Method in class test.RegExpUtilsTest: **testNanosecStamp()** - Method in class test.TimeUtilsTest: **testNextInt()** - Method in class uk.ac.ed.inf.utils.stats.tests.TruncatedParetoTest: **testNodeHeadToString()** - Method in class test.XmlNodeTest: **testObjectIncrements()** - Method in class test.UtilsTest: **testPareto()** - Method in class uk.ac.ed.inf.utils.stats.tests.ParetoTest: **testPareto()** - Method in class uk.ac.ed.inf.utils.stats.tests.TruncatedParetoTest: **testParetoOriginal()** - Method in class uk.ac.ed.inf.utils.stats.tests.StatUtilsTest: **testPerformance()** - Method in class uk.ac.ed.inf.utils.stats.tests.PseudoTruncatedParetoTest: **testPickInteger()** - Method in class uk.ac.ed.inf.utils.stats.tests.RandomUtilsTest: **testProbabilityDensityFunction()** - Method in class uk.ac.ed.inf.utils.stats.tests.StatUtilsTest: public void testCumulativeDistributionFunction(){ ParetoDistribution pareto = new ParetoDistribution(); System.out.println(pareto.getPareto().cdf(1.0)); System.out.println(pareto.getPareto().cdf(2.0)); System.out.println(pareto.getPareto().cdf(2.5)); System.out.println(pareto.getPareto().cdf(3.0)); System.out.println(pareto.getPareto().cdf(10.5)); System.out.println(pareto.getPareto().cdf(100.5)); System.out.println("-------"); } **testPseudoTruncatedPareto()** - Method in class uk.ac.ed.inf.utils.stats.tests.PseudoTruncatedParetoTest: **testRandomDoubleNumber()** - Method in class uk.ac.ed.inf.utils.stats.tests.RandomUtilsTest: **testRandomIntNumber()** - Method in class uk.ac.ed.inf.utils.stats.tests.RandomUtilsTest: **testRandomSubmap()** - Method in class uk.ac.ed.inf.utils.stats.tests.RandomUtilsTest: **testRemoveKeyValue()** - Method in class test.maputils.IndexedOneToManyMapTest: **testRemoveKeyValue()** - Method in class test.maputils.OneToManyMapTest: **testRemoveValue()** - Method in class test.maputils.TableMapTest: **testRowExistsInTable()** - Method in class test.database.TableReaderTest: **testSeries()** - Method in class test.UtilsTest: **testSetFromList()** - Method in class test.ListUtilsTest: **testSimpleElement()** - Method in class test.XmlNodeTest: **testStringContainsPositiveInteger()** - Method in class test.StringUtilsTest: **testStringIsNullOrEmpty()** - Method in class test.ListUtilsTest: **testStringIsNullOrEmpty()** - Method in class test.StringUtilsTest: **testStringMatchesRegExp()** - Method in class test.RegExpUtilsTest: **testStripHtml()** - Method in class test.RegExpUtilsTest: **testSubstituteMatchedString()** - Method in class test.RegExpUtilsTest: **testSurroundWith()** - Method in class test.database.SqlUtilsTest: **testTableColumnsAreSorted()** - Method in class test.database.TableTest: **testTableExistsInDb()** - Method in class test.database.DbReaderTest: **testToString()** - Method in class test.XmlNodeTest: **testTrimList()** - Method in class test.ListUtilsTest: **testTruncatedPareto()** - Method in class uk.ac.ed.inf.utils.stats.tests.TruncatedParetoTest: **THRESHOLD\_100** - Static variable in class uk.ac.ed.inf.utils.stats.tests.TruncatedParetoTest: **tidyDOM(InputStream)** - Static method in class uk.ac.ed.inf.utils.webutils.XMLUtils: JTidy is designed to read-in typical-quality (that is to say, bad) HTML and output something cleaner (you have a choice of options), and also provides a DOM interface for traversing HTML documents that can be fed to an XML parser. **TIMESTAMP\_DOWNLOADED** - Static variable in class uk.ac.ed.inf.utils.database.DbUtils: **TIMESTAMP\_SQL\_DATATYPE** - Static variable in class uk.ac.ed.inf.utils.database.DbUtils: **timestampedComment(String)** - Static method in class uk.ac.ed.inf.utils.TimeUtils: **TimeUtils** - Class in uk.ac.ed.inf.utils: Utilities to manipulate time objects, create timestamps etc. **TimeUtils()** - Constructor for class uk.ac.ed.inf.utils.TimeUtils: **TimeUtilsTest** - Class in test: Class **TimeUtilsTest()** - Constructor for class test.TimeUtilsTest: **toCSV()** - Method in class uk.ac.ed.inf.utils.maputils.OneToManyMap: Get a list of comma separated [key, value] couples. **toReadableString()** - Method in class uk.ac.ed.inf.utils.setutils.Set: **toString()** - Method in class cern.jet.random.Pareto: Returns a String representation of the receiver. **toString()** - Method in class uk.ac.ed.inf.utils.database.TableRow: Returns a line with a comma-separated list of table column names followed by a line with the comma-separated list of values. **toString()** - Method in class uk.ac.ed.inf.utils.diff.Difference: Returns a string representation of this difference. **toString()** - Method in class uk.ac.ed.inf.utils.guiutils.CommandLineMenu: "-n The file containing the network e.g. **toString()** - Method in class uk.ac.ed.inf.utils.guiutils.CommandOption: **toString()** - Method in class uk.ac.ed.inf.utils.maputils.OneToManyMap: **toString()** - Method in class uk.ac.ed.inf.utils.setutils.Set: **toString()** - Method in class uk.ac.ed.inf.utils.webutils.simpledomparser.XmlNode: Full string representation of node and all child nodes. **treeMapsHaveEqualValues(TreeMap<Integer, Character>, TreeMap<Integer, Character>)** - Static method in class uk.ac.ed.inf.utils.maputils.MapUtils: Check if two treeMaps have the same collection of values **trimList(Vector<String>)** - Static method in class uk.ac.ed.inf.utils.VectorUtils: trim all trailing white spaces from strings in a list (beware: null elements become empty strings) **TruncatedPareto** - Class in uk.ac.ed.inf.utils.stats: A utility class to extract from a truncated Pareto (power-law, long-tail) distribution. **TruncatedPareto(double, double, int, int)** - Constructor for class uk.ac.ed.inf.utils.stats.TruncatedPareto: **TruncatedParetoTest** - Class in uk.ac.ed.inf.utils.stats.tests: Class **TruncatedParetoTest()** - Constructor for class uk.ac.ed.inf.utils.stats.tests.TruncatedParetoTest

---


|  |  |  |  |  |  |  |  |  |  |  |
| --- | --- | --- | --- | --- | --- | --- | --- | --- | --- | --- |
| |  |  |  |  |  |  |  |  | | --- | --- | --- | --- | --- | --- | --- | --- | | **Overview** | Package | Class | Use | **Tree** | **Deprecated** | **Index** | **Help** | | |  |
| **PREV LETTER**   **NEXT LETTER** | **FRAMES**    **NO FRAMES**     **All Classes** |


A B C D E F G H I J K L M N O P Q R S T U V W X Y 

---
