# Supplementary material for: EnzML: multi-label prediction of enzyme classes using InterPro signatures
Source: BMC Bioinformatics. 2012 Apr 25;13:61. doi: 10.1186/1471-2105-13-61 (PMC3483700; doi:10.1186/1471-2105-13-61)
Supplement: Addtional file 5 — The Java code to format the data files, evaluate and predict. The file enzml_java_code.tar.gz contains the Java code used to format database data to ARFF and XML formats, to execute cross and train-test (jackknife) evaluations and to record evaluation results to database. More information is included in the readme.txt file and the Javadoc files. The code can be used with a MySQL database. To use a different database software, other JDBC drivers might be required. [file 1471-2105-13-61-S5.gz › java_code/utils/doc/index-files/index-14.html]

N-Index


---


|  |  |  |  |  |  |  |  |  |  |  |
| --- | --- | --- | --- | --- | --- | --- | --- | --- | --- | --- |
| |  |  |  |  |  |  |  |  | | --- | --- | --- | --- | --- | --- | --- | --- | | **Overview** | Package | Class | Use | **Tree** | **Deprecated** | **Index** | **Help** | | |  |
| **PREV LETTER**   **NEXT LETTER** | **FRAMES**    **NO FRAMES**     **All Classes** |


A B C D E F G H I J K L M N O P Q R S T U V W X Y 

---


## **N**

**nextDouble()** - Method in class cern.jet.random.AbstractDistribution: Returns a random number from the distribution. **nextDouble()** - Method in class cern.jet.random.engine.RandomEngine: Returns a 64 bit uniformly distributed random number in the open unit interval `(0.0,1.0)` (excluding 0.0 and 1.0). **nextDouble()** - Method in class cern.jet.random.Pareto: Returns a random number from the distribution. **nextDouble(double, double)** - Method in class cern.jet.random.Pareto: Returns a random number from the distribution; bypasses the internal state. **nextFloat()** - Method in class cern.jet.random.engine.RandomEngine: Returns a 32 bit uniformly distributed random number in the open unit interval `(0.0f,1.0f)` (excluding 0.0f and 1.0f). **nextInt()** - Method in class cern.jet.random.AbstractDistribution: Returns a random number from the distribution; returns (int) Math.round(nextDouble()). **nextInt()** - Method in class cern.jet.random.engine.MersenneTwister: Returns a 32 bit uniformly distributed random number in the closed interval [Integer.MIN\_VALUE,Integer.MAX\_VALUE] (including Integer.MIN\_VALUE and Integer.MAX\_VALUE). **nextInt()** - Method in class cern.jet.random.engine.RandomEngine: Returns a 32 bit uniformly distributed random number in the closed interval [Integer.MIN\_VALUE,Integer.MAX\_VALUE] (including Integer.MIN\_VALUE and Integer.MAX\_VALUE); **nextInt()** - Method in class uk.ac.ed.inf.utils.stats.TruncatedPareto: **nextLong()** - Method in class cern.jet.random.engine.RandomEngine: Returns a 64 bit uniformly distributed random number in the closed interval [Long.MIN\_VALUE,Long.MAX\_VALUE] (including Long.MIN\_VALUE and Long.MAX\_VALUE). **nextPseudoInt()** - Method in class uk.ac.ed.inf.utils.stats.PseudoTruncatedPareto: **nodeHeadToString()** - Method in class uk.ac.ed.inf.utils.webutils.simpledomparser.XmlNode: String representation of the node opening tag **NONE** - Static variable in class uk.ac.ed.inf.utils.diff.Difference: **NULL** - Static variable in class uk.ac.ed.inf.utils.database.SqlUtils: Basic data type: null (the type could not be guessed, eg. **NumberUtils** - Class in uk.ac.ed.inf.utils: Class **NumberUtils()** - Constructor for class uk.ac.ed.inf.utils.NumberUtils: **NumberUtilsTest** - Class in test: Class **NumberUtilsTest()** - Constructor for class test.NumberUtilsTest

---


|  |  |  |  |  |  |  |  |  |  |  |
| --- | --- | --- | --- | --- | --- | --- | --- | --- | --- | --- |
| |  |  |  |  |  |  |  |  | | --- | --- | --- | --- | --- | --- | --- | --- | | **Overview** | Package | Class | Use | **Tree** | **Deprecated** | **Index** | **Help** | | |  |
| **PREV LETTER**   **NEXT LETTER** | **FRAMES**    **NO FRAMES**     **All Classes** |


A B C D E F G H I J K L M N O P Q R S T U V W X Y 

---
