# Supplementary material for: EnzML: multi-label prediction of enzyme classes using InterPro signatures
Source: BMC Bioinformatics. 2012 Apr 25;13:61. doi: 10.1186/1471-2105-13-61 (PMC3483700; doi:10.1186/1471-2105-13-61)
Supplement: Addtional file 5 — The Java code to format the data files, evaluate and predict. The file enzml_java_code.tar.gz contains the Java code used to format database data to ARFF and XML formats, to execute cross and train-test (jackknife) evaluations and to record evaluation results to database. More information is included in the readme.txt file and the Javadoc files. The code can be used with a MySQL database. To use a different database software, other JDBC drivers might be required. [file 1471-2105-13-61-S5.gz › java_code/utils/doc/index-files/index-7.html]

 
 
 
 
 
 
G-Index
 

 

 

 
function windowTitle()
{
    if (location.href.indexOf('is-external=true') == -1) {
        parent.document.title="G-Index";
    }
}
 
 
 

 

 
 


 
   
  
 
 
 
   
 
   
          Overview     
        Package   
        Class   
        Use   
          Tree     
          Deprecated     
       Index    
          Help     
   
 
 
  
 
 
 

 
  
   PREV LETTER   
   NEXT LETTER    
  
    FRAMES     
   NO FRAMES     
  
    All Classes  ');
  }
  //-->
 
 
    All Classes  
 


  
 
 
  
 

 A   B   C   D   E   F   G   H   I   J   K   L   M   N   O   P   Q   R   S   T   U   V   W   X   Y   
    
 G  
 
   gaussian()   - 
Method in class edu.cornell.lassp.houle.RngPack. RandomElement 
 gaussian() uses the Box-Muller algorithm to transform raw()'s into
 gaussian deviates.
   gaussian(double)   - 
Method in class edu.cornell.lassp.houle.RngPack. RandomElement 
  
   generateSupersets()   - 
Method in class uk.ac.ed.inf.utils.setutils. SupersetsManager 
  
   getAddedEnd()   - 
Method in class uk.ac.ed.inf.utils.diff. Difference 
 The point at which the addition ends, if any.
   getAddedStart()   - 
Method in class uk.ac.ed.inf.utils.diff. Difference 
 The point at which the addition starts, if any.
   getAKeyValues(T)   - 
Method in class uk.ac.ed.inf.utils.maputils. IndexedOneToManyMap 
 Get the set of unique values for this key.
   getAKeyValues(T)   - 
Method in class uk.ac.ed.inf.utils.maputils. OneToManyMap 
 Get the set of unique values for this key.
   getAllMatchedSubstring(String, String, boolean)   - 
Static method in class uk.ac.ed.inf.utils. RegExpUtils 
 Returns all regular expression matches in the text (as strings)
   getAllMatchedSubstringsEndpoints(String, String, boolean)   - 
Static method in class uk.ac.ed.inf.utils. RegExpUtils 
 Gets the start and end index of all regular expression matches in the
 text
   getAttributeValue(String)   - 
Method in class uk.ac.ed.inf.utils.webutils.simpledomparser. XmlNode 
 Get the attribute value for the given attribute name.
   getBasicTypeOfDataColumn(ArrayList<String>)   - 
Static method in class uk.ac.ed.inf.utils.database. DbUtils 
 Given a list of strings, it returns a generic data type for the database
 column that could best contain them: INTEGER, DOUBLE, STRING
 
 If the strings passes a parseInteger: type = INTEGER if the strings
 contain digits, 'E' (for exponential), dot, plus, minus: type = DOUBLE If
 the strings contain letters or other characters: type = STRING Note: If
 the string contains only digits, but also intervening white spaces: type
 = STRING
   getChildElements()   - 
Method in class uk.ac.ed.inf.utils.webutils.simpledomparser. XmlNode 
 public Object[] getChildElements() { return m_childElements.toArray(); }
   getChosenOption()   - 
Method in class uk.ac.ed.inf.utils.guiutils. SimpleRadioButtonPanel 
  
   getCollection()   - 
Static method in class test. CollectionUtilsTest 
  
   getColumnAndValueMap()   - 
Method in class uk.ac.ed.inf.utils.database. TableRow 
  
   getColumnName()   - 
Method in class uk.ac.ed.inf.utils.database. TableColumn 
 Gets the column name
   getColumnNames()   - 
Method in class uk.ac.ed.inf.utils.database. Table 
 Gets column names for the table
   getColumns()   - 
Method in class uk.ac.ed.inf.utils.database. Table 
 Get the   TableColumn   list
   getColumns()   - 
Method in class uk.ac.ed.inf.utils.database. TableRow 
  
   getColumnsfrom2DArray(String[][])   - 
Static method in class uk.ac.ed.inf.utils.database. DbUtils 
 Takes a 2 dimensional array of data and returns the columns (as an array
 list of array lists)
   getColumnValues(ResultSet, int, AbstractCollection<T>)   - 
Method in class uk.ac.ed.inf.utils.database. DbReader 
 Get the nth column of a  ResultSet 
   getCommonElements(int[], int[])   - 
Static method in class uk.ac.ed.inf.utils. ArrayUtils 
 Gets the elements present in both lists
   getCommonElements(Object[], Object[])   - 
Static method in class uk.ac.ed.inf.utils. ArrayUtils 
 Gets the elements present in both lists
   getCommonElements(ArrayList, ArrayList)   - 
Static method in class uk.ac.ed.inf.utils. ListUtils 
 Gets the elements present in both lists
   getConnection()   - 
Method in class test.database. DbUtilsTest 
  
   getConnection()   - 
Method in class uk.ac.ed.inf.utils.database. DbConn 
  
   getConnectionParametersString()   - 
Method in class uk.ac.ed.inf.utils.database. DbConn 
 Get the connection log (database driver + url)
   getDatabaseName()   - 
Method in class uk.ac.ed.inf.utils.database. DbConn 
 Gets the database the connection is connected to
   getDatabaseName()   - 
Method in class uk.ac.ed.inf.utils.database. DbManager 
  
   getDatabaseType()   - 
Method in class uk.ac.ed.inf.utils.database. DbConn 
  
   getDataDbManager()   - 
Static method in class test.database. DbManagerTest 
  
   getDataType()   - 
Method in class uk.ac.ed.inf.utils.database. TableColumn 
 Gets the column data type
   getDataTypeForAnArray(ArrayList<String>)   - 
Static method in class uk.ac.ed.inf.utils.database. DbUtils 
 Guesses from data type and length the appropriate SQL type of a column to
 contain the data in the array
   getDateForFileName()   - 
Static method in class uk.ac.ed.inf.utils. TimeUtils 
 getFileTimeStamp returns a date stamp that can be attached to file names
   getDateStamp()   - 
Static method in class uk.ac.ed.inf.utils. TimeUtils 
 Returns a date stamp in the format: 20070117 yyyymmdd
   getDateStrings()   - 
Static method in class uk.ac.ed.inf.utils. TimeUtils 
 getDateStrings()
   getDateTimeMillisecondsStamp()   - 
Static method in class uk.ac.ed.inf.utils. TimeUtils 
 Returns a date-time-milliseconds stamp in the format: 20070117_111847_123
 yyyymmdd_hhmmss_lll
   getDateTimeNanoSecondsStamp()   - 
Static method in class uk.ac.ed.inf.utils. TimeUtils 
 Returns a date-time-milliseconds-nanoseconds stamp in the format:
 20070117_111847_123_3000 yyyymmdd_hhmmss_lll_nnnn (the n digits are not
 the nanoseconds! just a progressive stamp)
   getDateTimeStamp()   - 
Static method in class uk.ac.ed.inf.utils. TimeUtils 
 Returns a date-time stamp in the format: 20070117_111847 yyyymmdd_hhmmss
   getDbConnection()   - 
Method in class uk.ac.ed.inf.utils.database. DbManaged 
  
   getDbConnection()   - 
Method in class uk.ac.ed.inf.utils.database. DbManager 
  
   getDbConnProps()   - 
Static method in class test.database. DbConnPropsTest 
  
   getDbCreator()   - 
Static method in class test.database. DbCreatorTest 
  
   getDbCreator()   - 
Method in class uk.ac.ed.inf.utils.database. DbManager 
  
   getDbManager()   - 
Static method in class test.database. DbManagerTest 
  
   getDbManager()   - 
Method in class uk.ac.ed.inf.utils.database. DbManaged 
  
   getDbMetaData()   - 
Method in class uk.ac.ed.inf.utils.database. DbReader 
  
   getDbReader()   - 
Static method in class test.database. DbReaderTest 
  
   getDbReader()   - 
Method in class uk.ac.ed.inf.utils.database. DbManager 
  
   getDbWriter()   - 
Static method in class test.database. DbWriterTest 
  
   getDbWriter()   - 
Method in class uk.ac.ed.inf.utils.database. DbManager 
  
   getDeletedEnd()   - 
Method in class uk.ac.ed.inf.utils.diff. Difference 
 The point at which the deletion ends, if any.
   getDeletedStart()   - 
Method in class uk.ac.ed.inf.utils.diff. Difference 
 The point at which the deletion starts, if any.
   getDirectoryFiles(String)   - 
Static method in class uk.ac.ed.inf.utils. FileUtils 
 Gets the list of files in a directory From
 http://www.faqs.org/docs/javap/c10/s2.html
   getDirectoryPath(String)   - 
Static method in class uk.ac.ed.inf.utils. FileUtils 
 Get the parent of a full file path (directory + filename)
   getDoubleFromHashmap(HashMap, String)   - 
Static method in class uk.ac.ed.inf.utils.maputils. MapUtils 
 Gets a double from a hashMap
   getDoubleIntervals(Double, Double, Double)   - 
Static method in class uk.ac.ed.inf.utils. Utils 
  
   getDriverName()   - 
Method in class uk.ac.ed.inf.utils.database. DbConn 
  
   getElapsedTime(long, long)   - 
Static method in class uk.ac.ed.inf.utils. TimeUtils 
 Returns a string with the elapsed time in a hour:min:sec.msec format
   getElements()   - 
Method in class uk.ac.ed.inf.utils.setutils. Set 
  
   getElementsSize()   - 
Method in class uk.ac.ed.inf.utils.setutils. Set 
  
   getESummaryXML(String, String)   - 
Static method in class uk.ac.ed.inf.utils. EntrezUtils 
 Gets an XML tree of elements (SimpleElement) from eSummary Entrez
 service.
   getFileNameFromDirectory(String, String)   - 
Static method in class uk.ac.ed.inf.utils. FileUtils 
 Get the first file name in the directory matching the given pattern
 string.
   getFileNameFromFullPath(String)   - 
Static method in class uk.ac.ed.inf.utils. FileUtils 
 Get the filename from a full file path (directory + filename)
   getFileSizeInKb(String)   - 
Static method in class uk.ac.ed.inf.utils. FileUtils 
 Get the file size in Kb
   getFirstMatchedSubstring(String, String)   - 
Static method in class uk.ac.ed.inf.utils. RegExpUtils 
 Gets the first matched substring in the text (dot all mode disabled > the
 dot does not match the \n character)
   getFirstMatchedSubstring(String, String, boolean)   - 
Static method in class uk.ac.ed.inf.utils. RegExpUtils 
 Gets the first matched substring in the text
   getFirstValueOccurrence(Map<String, String>, String)   - 
Static method in class uk.ac.ed.inf.utils.maputils. MapUtils 
 Get all the keys that have the given value in a Map\ 
   getFlag(String, String[])   - 
Static method in class uk.ac.ed.inf.utils.guiutils. OptionUtils 
  
   getFlag(String, String[])   - 
Static method in class uk.ac.ed.inf.utils. Utils 
  
   getFrequencies()   - 
Static method in class uk.ac.ed.inf.utils.stats.tests. StatUtilsTest 
  
   getGunzipInputStream(BufferedInputStream)   - 
Static method in class uk.ac.ed.inf.utils. FileUtils 
  
   getHost()   - 
Method in class uk.ac.ed.inf.utils.database. DbConn 
  
   getHumanReadableTimeStamp()   - 
Static method in class uk.ac.ed.inf.utils. TimeUtils 
 Returns a human readable date-time stamp
   getId()   - 
Method in class uk.ac.ed.inf.utils.setutils. Set 
  
   getIdColumn()   - 
Static method in class uk.ac.ed.inf.utils.database. DbUtils 
 get the definition for an "id" column of type auto-increment (which is
 primary key)
   getIncrements(Object, Object, Double)   - 
Static method in class uk.ac.ed.inf.utils. Utils 
 Returns a vector of numbers, starting from a minimum value, with
 incremental increases until the maximum value.
   getIncrementsFromRange(Integer, Double)   - 
Static method in class uk.ac.ed.inf.utils. Utils 
 Returns a set of increments.
   getIndexedMap(Object[])   - 
Static method in class uk.ac.ed.inf.utils.maputils. MapUtils 
 Get a map with entries composed of: an integer id as key and an entry as
 value
   getInsertSql(TableRow)   - 
Method in class uk.ac.ed.inf.utils.database. TableWriter 
 Returns the sql "insert into" statement for the row
   getInstance(String)   - 
Static method in class uk.ac.ed.inf.utils. LoggerCreator 
  
   getInstance(double, double, int, int, int)   - 
Static method in class uk.ac.ed.inf.utils.stats. PseudoTruncatedParetoSingleton 
  
   getInstance()   - 
Static method in class uk.ac.ed.inf.utils.stats. UniformRandomSingleton 
  
   getIntegerIntervals(Integer, Integer, Double)   - 
Static method in class uk.ac.ed.inf.utils. Utils 
 Returns a vector of numbers, starting from a minimum value, with
 incremental increases until the maximum value.
   getInternalBlocks(String, XmlNode, File)   - 
Static method in class uk.ac.ed.inf.utils.webutils.simpledomparser. XmlSearcher 
 Gets the children of a given xml tag
   getJavaSystemProperties()   - 
Static method in class uk.ac.ed.inf.utils. PropertiesUtils 
 Gets the Java system properties
   getJavaSystemProperties()   - 
Static method in class uk.ac.ed.inf.utils. Utils 
 Gets the Java system properties
   getKeys()   - 
Method in class uk.ac.ed.inf.utils.maputils. OneToManyMap 
  
   getKeys()   - 
Method in class uk.ac.ed.inf.utils.maputils. TableMap 
  
   getKeysStrings(Properties)   - 
Static method in class uk.ac.ed.inf.utils. PropertiesUtils 
 Get the properties keys as strings
   getKeysVectorValuesVector()   - 
Method in class uk.ac.ed.inf.utils.maputils. OneToManyMap 
 Get 1.
   getKeyValuePairSize()   - 
Method in class uk.ac.ed.inf.utils.maputils. TableMap 
 Get the number of key-value pairs in the map
   getKeyValues(String)   - 
Method in class uk.ac.ed.inf.utils.maputils. TableMap 
  
   getKeyValuesPairsSize()   - 
Method in class uk.ac.ed.inf.utils.maputils. OneToManyMap 
 Get the number of unique key-value pairs in the map.
   getListOfDbTables()   - 
Method in class uk.ac.ed.inf.utils.database. DbReader 
 Gets the list of tables for a database
   getListOfValuesString(Vector<String>, String)   - 
Static method in class uk.ac.ed.inf.utils.database. DbUtils 
 Transforms a list of values into a comma separated list
   getLoadDataInfileSql(String, String, String, int, String)   - 
Method in class uk.ac.ed.inf.utils.database. TableWriter 
  
   getLongestCommonSubsequences()   - 
Method in class uk.ac.ed.inf.utils.diff. Diff 
 Returns an array of the longest common subsequences.
   getLongestStringLength(ArrayList<String>)   - 
Static method in class uk.ac.ed.inf.utils. ListUtils 
 Get the length of the longest string in an array
   getManager()   - 
Static method in class test.setutils. SupersetsManagerTest 
  
   getManager()   - 
Method in class uk.ac.ed.inf.utils.database. Managed 
  
   getManager2()   - 
Static method in class test.setutils. SupersetsManagerTest 
  
   getManager3()   - 
Static method in class test.setutils. SupersetsManagerTest 
  
   getMap()   - 
Static method in class test.maputils. MapUtilsTest 
  
   getMap(String)   - 
Method in class uk.ac.ed.inf.utils.database. DbReader 
 Converts a query resultset into a map.
   getMap(String, int, int)   - 
Method in class uk.ac.ed.inf.utils.database. DbReader 
 Converts a query resultset into a map.
   getMap()   - 
Method in class uk.ac.ed.inf.utils.maputils. OneToManyMap 
  
   getMap()   - 
Method in class uk.ac.ed.inf.utils.maputils. TableMap 
 Get the keys to values map
   getMap1()   - 
Static method in class test.maputils. MapUtilsTest 
  
   getMap2()   - 
Static method in class test.maputils. MapUtilsTest 
  
   getMatcher(String, String, boolean)   - 
Static method in class uk.ac.ed.inf.utils. RegExpUtils 
 Get a matcher trained on the given pattern/regular expression and
 pointing at the text of interest.
   getMilliseconds()   - 
Static method in class uk.ac.ed.inf.utils. TimeUtils 
 Returns a milliseconds time stamp
   getMillisecTimeStampForFileName()   - 
Static method in class uk.ac.ed.inf.utils. TimeUtils 
 Date-time stamp (up to milliseconds) that can be attached to file names
   getNameForEntrezGeneId(String)   - 
Static method in class uk.ac.ed.inf.utils. EntrezUtils 
 Get the entrez gene name for an entrez gene id
   getNanoSecondsStamp()   - 
Static method in class uk.ac.ed.inf.utils. TimeUtils 
 Returns a nanoseconds stamp (it's a progressive stamp actually, not the
 actual nanoseconds in desktop-clock style!)
   getNextNanoSecondsStamp(String)   - 
Static method in class uk.ac.ed.inf.utils. TimeUtils 
 Returns a nanoseconds stamp + 1 (it's a progressive stamp actually, not
 the actual nanoseconds in desktop-clock style!)
   getNonEmptyKeys()   - 
Method in class uk.ac.ed.inf.utils.maputils. OneToManyMap 
 Get the set of unique, non-empty-string keys for this map.
   getNonEmptyKeyValuesPairsSize()   - 
Method in class uk.ac.ed.inf.utils.maputils. OneToManyMap 
 Get the number of unique, non-empty key-value pairs in the map.
   getNonEmptyValues()   - 
Method in class uk.ac.ed.inf.utils.maputils. IndexedOneToManyMap 
 Get the set of unique, non-empty-string values for this map.
   getNonEmptyValues()   - 
Method in class uk.ac.ed.inf.utils.maputils. OneToManyMap 
 Get the set of unique, non-empty-string values for this map.
   getNoOfRows()   - 
Method in class uk.ac.ed.inf.utils.database. TableColumn 
 Gets the number of rows in the column
   getNumberedXmlTree()   - 
Static method in class test. XmlNodeTest 
  
   getOffset()   - 
Method in class uk.ac.ed.inf.utils.stats. TruncatedPareto 
  
   getOpenConnection()   - 
Method in class uk.ac.ed.inf.utils.database. DbConn 
  
   getOption(String, String[])   - 
Static method in class uk.ac.ed.inf.utils.guiutils. OptionUtils 
 GetOption(String opt, String[] options) gets an option value out of a
 list of options
   getOption(String, String[])   - 
Static method in class uk.ac.ed.inf.utils. Utils 
 getOption(String opt, String[] options) gets an option value out of a
 list of options
   getPareto()   - 
Method in class uk.ac.ed.inf.utils.stats. TruncatedPareto 
  
   getParetoFrequencies(int)   - 
Method in class uk.ac.ed.inf.utils.stats. TruncatedPareto 
 Extracts numbers from a pareto distribution for the given number of times
 and records the freqencies with which the integers have been extracted
   getPassword()   - 
Method in class uk.ac.ed.inf.utils.database. DbConn 
  
   getPdbSimpleElement()   - 
Static method in class test. XmlNodeTest 
  
   getPdbXmlReader()   - 
Static method in class test. XmlUtilsTest 
  
   getPort()   - 
Method in class uk.ac.ed.inf.utils.database. DbConn 
  
   getPotentialDirectory(File)   - 
Static method in class uk.ac.ed.inf.utils. FileUtils 
 Get a directory out of a path
   getPseudoRandomNumbers()   - 
Method in class uk.ac.ed.inf.utils.stats. PseudoTruncatedPareto 
  
   getPseudoTruncatedPareto()   - 
Method in class uk.ac.ed.inf.utils.stats. PseudoTruncatedParetoSingleton 
  
   getPubMedUrl(String)   - 
Static method in class uk.ac.ed.inf.utils. EntrezUtils 
 Generates pubMed URL adding pubMed ids (as list separated by commas)
   getRandomInt(int)   - 
Static method in class uk.ac.ed.inf.utils.stats. StatUtils 
 Problem: generates a new Random at each call...
   getRandomUtils()   - 
Static method in class uk.ac.ed.inf.utils.stats.tests. RandomUtilsTest 
  
   getReaderFromInputStream(InputStream)   - 
Static method in class uk.ac.ed.inf.utils.webutils. WebUtils 
 Creates a buffered reader from an input stream
   getResultSet(String, DbConn)   - 
Static method in class uk.ac.ed.inf.utils.database. DbUtils 
 Executes an sql query returning a result set
   getResultSetColumnAsVector(ResultSet, int)   - 
Static method in class uk.ac.ed.inf.utils.database. DbUtils 
 Takes a resultSet and returns an array of Strings
   getReverseMap()   - 
Method in class uk.ac.ed.inf.utils.maputils. TableMap 
 Get the values to keys map
   getRowKo()   - 
Static method in class test.database. TableRowTest 
  
   getRowOk()   - 
Static method in class test.database. TableRowTest 
  
   getRowValues(ResultSet, int)   - 
Method in class uk.ac.ed.inf.utils.database. TableReader 
 Get the nth row of a  ResultSet 
   getSeed()   - 
Method in class edu.cornell.lassp.houle.RngPack. Ranecu 
  
   getSelectStarSql(String)   - 
Static method in class uk.ac.ed.inf.utils.database. SqlUtils 
 Generates a select * query text (eg.
   getSelectWhereEqualSql(String, String, String)   - 
Static method in class uk.ac.ed.inf.utils.database. SqlUtils 
 gets sql code for a select on one column of a table, with a where x = y
 condition specific for that column
   getSelectWhereEqualSql(String, String, String, String, String)   - 
Static method in class uk.ac.ed.inf.utils.database. SqlUtils 
 Select on two columns of a table, with two "where" conditions, , one for
 each provided column
   getSelectWhereLikeSql(String, String, String)   - 
Static method in class uk.ac.ed.inf.utils.database. SqlUtils 
 Gets sql code for a select on one column of a table, with a where x like
 y condition specific for that column
   getSelectWhereSql(String, String, String)   - 
Static method in class uk.ac.ed.inf.utils.database. SqlUtils 
 gets sql code for a select on one column of a table, with a where
 condition
   getSeparator()   - 
Method in class uk.ac.ed.inf.utils.setutils. SupersetsManager 
  
   getSet(String, TreeSet<String>)   - 
Static method in class test.setutils. SetTest 
  
   getSetA()   - 
Static method in class test.setutils. SetTest 
  
   getSetAB()   - 
Static method in class test.setutils. SetTest 
  
   getSetABC()   - 
Static method in class test.setutils. SetTest 
  
   getSetAC()   - 
Static method in class test.setutils. SetTest 
  
   getSetAD()   - 
Static method in class test.setutils. SetTest 
  
   getSets()   - 
Method in class uk.ac.ed.inf.utils.setutils. SupersetsManager 
  
   getShortestStringLength(ArrayList<String>)   - 
Static method in class uk.ac.ed.inf.utils. ListUtils 
 Get the length of the shortest string in an array
   getSimpleSelectSql(String, String)   - 
Static method in class uk.ac.ed.inf.utils.database. SqlUtils 
 gets sql code for a select on one column of a table
   getSimpleSerie(Double, Double, int)   - 
Static method in class uk.ac.ed.inf.utils. Utils 
 Gets a series of doubles from a min incrementing by a given increment for
 a number of iterations
   getSqlForSelectLike(String, String, String, String)   - 
Static method in class uk.ac.ed.inf.utils.database. SqlUtils 
 Sql code for a select "LIKE"
   getSqlSelectByIdByRegExp(String, String, String, String, String)   - 
Static method in class uk.ac.ed.inf.utils.database. SqlUtils 
 Generates the sql command for a select of a value corresponding to a
 certain id AND matching a certain regex string
   getSqlSelectByRegExp(String, String, String)   - 
Static method in class uk.ac.ed.inf.utils.database. SqlUtils 
 Generates the sql command for a select matching a certain regex string
   getSqlTimestamp()   - 
Static method in class uk.ac.ed.inf.utils. TimeUtils 
 Gets a sql.Timestamp object of the current time (precise to the second:
 nanoseconds are set to zero)
   getSqlTimestampString()   - 
Static method in class uk.ac.ed.inf.utils. TimeUtils 
  
   getSqlToCreateTable()   - 
Method in class uk.ac.ed.inf.utils.database. TableCreator 
 Get the SQL statement to create the table
   getSqlToDropTable(String)   - 
Method in class uk.ac.ed.inf.utils.database. DbCreator 
 Get sql command to drop table
   getSqlToDropTable(String)   - 
Static method in class uk.ac.ed.inf.utils.database. SqlUtils 
 Get sql to drop table
   getStatement()   - 
Method in class uk.ac.ed.inf.utils.database. DbConn 
 Gets a statement from a new connection
   getStatement()   - 
Method in class uk.ac.ed.inf.utils.database. DbReader 
  
   getStreamFromFileUri(String)   - 
Static method in class uk.ac.ed.inf.utils.webutils. WebUtils 
  
   getStreamFromGzipUrl(String)   - 
Static method in class uk.ac.ed.inf.utils.webutils. WebUtils 
  
   getStreamFromURL(String)   - 
Static method in class uk.ac.ed.inf.utils.webutils. WebUtils 
 Creates an input stream from a URL (gets all the HTML or XML contained in
 the page)
   getString(String[])   - 
Static method in class uk.ac.ed.inf.utils. ArrayUtils 
 Returns a string representation for an array
   getStringFromBufferedReader(BufferedReader)   - 
Static method in class uk.ac.ed.inf.utils. FileUtils 
  
   getStringFromBufferedReader(BufferedReader)   - 
Static method in class uk.ac.ed.inf.utils.webutils. WebUtils 
 Get a string from a buffered reader
   getStringFromFile(String)   - 
Static method in class uk.ac.ed.inf.utils. FileUtils 
 Get all the text in a file as a single string
   getStringFromUrl(String, boolean)   - 
Static method in class uk.ac.ed.inf.utils.webutils. WebUtils 
 Get a string from a URL
   getSubsets()   - 
Method in class uk.ac.ed.inf.utils.setutils. Set 
  
   getSubsetsSize()   - 
Method in class uk.ac.ed.inf.utils.setutils. Set 
  
   getSupersets()   - 
Method in class uk.ac.ed.inf.utils.setutils. SupersetsManager 
  
   getSupersetsCSV()   - 
Method in class uk.ac.ed.inf.utils.setutils. SupersetsManager 
 Get a comma separated list of [superset, subset] couples.
   getSupersetsIds()   - 
Method in class uk.ac.ed.inf.utils.setutils. SupersetsManager 
  
   getSupersetsIdsCSV()   - 
Method in class uk.ac.ed.inf.utils.setutils. SupersetsManager 
 Get a comma separated list of [superset id, subset id] couples.
   getTableColumnsNames()   - 
Method in class uk.ac.ed.inf.utils.database. TableReader 
 Gets the list of columns for a database table (from:
 http://forums.sun.com/thread.jspa?threadID=501222&messageID=2369725)
   getTableCreator()   - 
Method in class uk.ac.ed.inf.utils.database. TableManager 
  
   getTableManager(String)   - 
Method in class uk.ac.ed.inf.utils.database. DbManager 
  
   getTableManager(Table)   - 
Method in class uk.ac.ed.inf.utils.database. DbManager 
  
   getTableManager()   - 
Method in class uk.ac.ed.inf.utils.database. TableManaged 
  
   getTableManager()   - 
Method in class uk.ac.ed.inf.utils.database. TableRow 
  
   getTableManagerWithDbManagerString()   - 
Static method in class test.database. TableManagerTest 
  
   getTableManagerWithDbManagerTable()   - 
Static method in class test.database. TableManagerTest 
  
   getTableName()   - 
Method in class uk.ac.ed.inf.utils.database. Table 
 Get the table name
   getTableName()   - 
Method in class uk.ac.ed.inf.utils.database. TableCreator 
  
   getTableName()   - 
Method in class uk.ac.ed.inf.utils.database. TableManager 
  
   getTableName()   - 
Method in class uk.ac.ed.inf.utils.database. TableReader 
  
   getTableReader(String)   - 
Method in class uk.ac.ed.inf.utils.database. DbReader 
 Object to read from a table
   getTableReader()   - 
Method in class uk.ac.ed.inf.utils.database. TableManager 
  
   getTableRowWhereEqual(String, String)   - 
Method in class uk.ac.ed.inf.utils.database. TableReader 
 Gets the *first* table row where the given column has exactly the given
 value
   getTableRowWhereLike(String, String)   - 
Method in class uk.ac.ed.inf.utils.database. TableReader 
 Gets the *first* table row where the given column has a value similar to
 the given value
   getTableWriter(String)   - 
Method in class uk.ac.ed.inf.utils.database. DbWriter 
 Object to write to a table
   getTableWriter()   - 
Method in class uk.ac.ed.inf.utils.database. TableManager 
  
   getTagName()   - 
Method in class uk.ac.ed.inf.utils.webutils.simpledomparser. XmlNode 
 Get the tag E.g.
   getTest1Table()   - 
Static method in class test.database. TableTest 
  
   getTestHtml()   - 
Static method in class test. RegExpUtilsTest 
  
   getText()   - 
Method in class uk.ac.ed.inf.utils.webutils.simpledomparser. XmlNode 
 Get text for the tag E.g.
   getTextFromChildElement(XmlNode, String, String, File)   - 
Static method in class uk.ac.ed.inf.utils.webutils.simpledomparser. XmlSearcher 
 Checks if child exists and returns its text
   getTimeDownloadedColumn()   - 
Static method in class uk.ac.ed.inf.utils.database. DbUtils 
  
   getTimeOnlyStamp()   - 
Static method in class uk.ac.ed.inf.utils. TimeUtils 
 Returns a time stamp in the format (for 11 am, 18 min, 47 sec): 111847
 hhmmss
   getTimestampColumn()   - 
Static method in class uk.ac.ed.inf.utils.database. DbUtils 
  
   getTimeStampForFileName()   - 
Static method in class uk.ac.ed.inf.utils. TimeUtils 
 Date-time stamp (up to seconds) that can be attached to file names
   getTruncatedPareto100()   - 
Static method in class uk.ac.ed.inf.utils.stats.tests. TruncatedParetoTest 
  
   getTruncationThreshold()   - 
Method in class uk.ac.ed.inf.utils.stats. TruncatedPareto 
  
   getUniformRandomGenerator()   - 
Method in class uk.ac.ed.inf.utils.stats. UniformRandomSingleton 
  
   getUniformRandomSubmap(Map, double)   - 
Static method in class uk.ac.ed.inf.utils.stats. UniformRandomUtils 
 Returns a submap of randomly picked map entries
   getUrl()   - 
Method in class uk.ac.ed.inf.utils.database. DbConn 
  
   getUser()   - 
Method in class uk.ac.ed.inf.utils.database. DbConn 
  
   getValue(String)   - 
Method in class uk.ac.ed.inf.utils.database. TableRow 
 get the value for a certain column
   getValue(Double)   - 
Static method in class uk.ac.ed.inf.utils. NumberUtils 
 Gets a string value usable for SQL DOUBLE column.
   getValues()   - 
Method in class uk.ac.ed.inf.utils.database. TableRow 
  
   getValues()   - 
Method in class uk.ac.ed.inf.utils.maputils. IndexedOneToManyMap 
 Get the set of unique values for this map.
   getValues()   - 
Method in class uk.ac.ed.inf.utils.maputils. OneToManyMap 
 Get the set of unique values for this map.
   getValues()   - 
Method in class uk.ac.ed.inf.utils.maputils. TableMap 
  
   getValuesStrings(Properties)   - 
Static method in class uk.ac.ed.inf.utils. PropertiesUtils 
 Get the properties values as strings
   getXml()   - 
Static method in class test. XmlUtilsTest 
  
   getXml1String()   - 
Static method in class test. XmlNodeTest 
  
   getXml2String()   - 
Static method in class test. XmlNodeTest 
  
   getXml3String()   - 
Static method in class test. XmlNodeTest 
  
   getXml4String()   - 
Static method in class test. XmlNodeTest 
  
   getXml5String()   - 
Static method in class test. XmlNodeTest 
  
   getXml6String()   - 
Static method in class test. XmlNodeTest 
  
   getXml7String()   - 
Static method in class test. XmlNodeTest 
  
   getXmlComment(String)   - 
Static method in class uk.ac.ed.inf.utils.webutils. XMLUtils 
 Returns a string formatted as xml comment:
   getXmlFromHTML(String, String)   - 
Static method in class uk.ac.ed.inf.utils.webutils. XMLUtils 
  
   getXmlFromReader(BufferedReader)   - 
Static method in class uk.ac.ed.inf.utils.webutils. XMLUtils 
 Get the XML as SimpleElement from a URL reader.
   getXmlString()   - 
Static method in class test. XmlNodeTest 
  
   getXmlStringReader()   - 
Static method in class test. XmlUtilsTest 
  
   getXmlTreeFromString(String)   - 
Static method in class uk.ac.ed.inf.utils.webutils.simpledomparser. XmlNode 
  
   getXmlTreeFromUrl(String, boolean)   - 
Static method in class uk.ac.ed.inf.utils.webutils.simpledomparser. XmlNode 
 get XML tree from URL
   GuiUtils   - Class in  uk.ac.ed.inf.utils.guiutils  Gui utilities   GuiUtils()   - 
Constructor for class uk.ac.ed.inf.utils.guiutils. GuiUtils 
  
   gunzipFile(File)   - 
Static method in class uk.ac.ed.inf.utils. FileUtils 
  
   gunzipFile2(File, File)   - 
Static method in class uk.ac.ed.inf.utils. FileUtils 
 Gunzip the input archive.
 
 


 
   
  
 
 
 
   
 
   
          Overview     
        Package   
        Class   
        Use   
          Tree     
          Deprecated     
       Index    
          Help     
   
 
 
  
 
 
 

 
  
   PREV LETTER   
   NEXT LETTER    
  
    FRAMES     
   NO FRAMES     
  
    All Classes  ');
  }
  //-->
 
 
    All Classes  
 


  
 
 
  
 

 A   B   C   D   E   F   G   H   I   J   K   L   M   N   O   P   Q   R   S   T   U   V   W   X   Y   

 
 
