# Supplementary material for: EnzML: multi-label prediction of enzyme classes using InterPro signatures
Source: BMC Bioinformatics. 2012 Apr 25;13:61. doi: 10.1186/1471-2105-13-61 (PMC3483700; doi:10.1186/1471-2105-13-61)
Supplement: Addtional file 5 — The Java code to format the data files, evaluate and predict. The file enzml_java_code.tar.gz contains the Java code used to format database data to ARFF and XML formats, to execute cross and train-test (jackknife) evaluations and to record evaluation results to database. More information is included in the readme.txt file and the Javadoc files. The code can be used with a MySQL database. To use a different database software, other JDBC drivers might be required. [file 1471-2105-13-61-S5.gz › java_code/utils/doc/index-files/index-3.html]

C-Index


---


|  |  |  |  |  |  |  |  |  |  |  |
| --- | --- | --- | --- | --- | --- | --- | --- | --- | --- | --- |
| |  |  |  |  |  |  |  |  | | --- | --- | --- | --- | --- | --- | --- | --- | | **Overview** | Package | Class | Use | **Tree** | **Deprecated** | **Index** | **Help** | | |  |
| **PREV LETTER**   **NEXT LETTER** | **FRAMES**    **NO FRAMES**     **All Classes** |


A B C D E F G H I J K L M N O P Q R S T U V W X Y 

---


## **C**

**calcAverage(double[])** - Static method in class uk.ac.ed.inf.utils.Utils: Calculates the average of the numbers in an array (from web http://forum.java.sun.com/, author JosAH) **calcStandardDeviation(double[])** - Static method in class uk.ac.ed.inf.utils.Utils: Calculates the standard deviation of the elements in an array as root of the variance = [sum( x(i) - mean)^2 )] / (n-1), where x(i) is the ith element, m the mean, n the number of elements **canReadFile(File)** - Static method in class uk.ac.ed.inf.utils.FileUtils: Tests wether the file can be read **canReadFile(String)** - Static method in class uk.ac.ed.inf.utils.FileUtils: Tests whether the file can be read **canReadParent(String)** - Static method in class uk.ac.ed.inf.utils.FileUtils: Checks whether a file directory is readable. **canWriteFile(File)** - Static method in class uk.ac.ed.inf.utils.FileUtils: Tests whether the file can be written **canWriteFile(String)** - Static method in class uk.ac.ed.inf.utils.FileUtils: Tests wether the file can be written **canWriteParent(String)** - Static method in class uk.ac.ed.inf.utils.FileUtils: Checks whether a file directory is writable. **cdf(double)** - Method in class cern.jet.random.Pareto: Returns the cumulative distribution function. **cern.colt** - package cern.colt: **cern.colt.function** - package cern.colt.function: **cern.jet.random** - package cern.jet.random: **cern.jet.random.engine** - package cern.jet.random.engine: **CHAR\_2\_SQL\_DATATYPE** - Static variable in class uk.ac.ed.inf.utils.database.DbUtils: **CHAR\_4\_SQL\_DATATYPE** - Static variable in class uk.ac.ed.inf.utils.database.DbUtils: **checkRow()** - Method in class uk.ac.ed.inf.utils.database.TableRow: **checkRowFields()** - Method in class uk.ac.ed.inf.utils.database.TableRow: checks that the record values are paired to fields existing in the table **checkRowValues()** - Method in class uk.ac.ed.inf.utils.database.TableRow: TODO Not implemented: check that each row value is of the appropriate datatype for the table field... **choiceEvent** - Variable in class uk.ac.ed.inf.utils.guiutils.SimpleRadioButtonPanel: **choose(int)** - Method in class edu.cornell.lassp.houle.RngPack.RandomElement: **choose(int, int)** - Method in class edu.cornell.lassp.houle.RngPack.RandomElement: **classFieldNames(Object)** - Static method in class uk.ac.ed.inf.utils.ReflectionUtils: public static String classFieldsNamesAndValuesString(Object o){ TreeMap map = ReflectionUtils.classFieldsNamesAndValues(o); String fieldsValues = "Fields of " + o.getClass().getName() + "\n"; Set> entries = map.entrySet(); Iterator> it = entries.iterator(); while(it.hasNext()){ Entry entry = it.next(); fieldsValues += entry.getKey() } return fieldsValues; } **classFieldsNamesAndValues(Object)** - Static method in class uk.ac.ed.inf.utils.ReflectionUtils: **cleanSet(Set<String>)** - Static method in class uk.ac.ed.inf.utils.ListUtils: Cleans a set from null and empty elements **ClientHttpRequestUtils** - Class in uk.ac.ed.inf.utils.webutils: Title: Client HTTP Request class **ClientHttpRequestUtils(String)** - Constructor for class uk.ac.ed.inf.utils.webutils.ClientHttpRequestUtils: Creates a new multipart POST HTTP request for a specified URL string **ClientHttpRequestUtils(URL)** - Constructor for class uk.ac.ed.inf.utils.webutils.ClientHttpRequestUtils: Creates a new multipart POST HTTP request for a specified URL **ClientHttpRequestUtils(URLConnection)** - Constructor for class uk.ac.ed.inf.utils.webutils.ClientHttpRequestUtils: Creates a new multipart POST HTTP request on a freshly opened URLConnection **ClockSeed()** - Static method in class edu.cornell.lassp.houle.RngPack.RandomSeedable: Return a long integer seed calculated from the date. **ClockSeed(Date)** - Static method in class edu.cornell.lassp.houle.RngPack.RandomSeedable: Return a long integer seed given a date **clone()** - Method in class cern.colt.PersistentObject: Returns a copy of the receiver. **clone()** - Method in class cern.jet.random.AbstractDistribution: Returns a deep copy of the receiver; the copy will produce identical sequences. **clone()** - Method in class cern.jet.random.engine.MersenneTwister: Returns a copy of the receiver; the copy will produce identical sequences. **clone()** - Method in class edu.cornell.lassp.houle.RngPack.RandomShuffle: Returns a copy of the receiver. **clone()** - Method in class edu.cornell.lassp.houle.RngPack.Ranlux: Returns a copy of the receiver; the copy will produce identical sequences. **clone()** - Method in class edu.cornell.lassp.houle.RngPack.Ranmar: Returns a copy of the receiver; the copy will produce identical sequences. **clone()** - Method in class uk.ac.ed.inf.utils.setutils.Set: **closeConnection()** - Method in class uk.ac.ed.inf.utils.database.DbConn: Close the database connection **closeConnection()** - Method in class uk.ac.ed.inf.utils.database.DbManager: **CollectionUtils** - Class in uk.ac.ed.inf.utils: Class **CollectionUtils()** - Constructor for class uk.ac.ed.inf.utils.CollectionUtils: **CollectionUtilsTest** - Class in test: Class **CollectionUtilsTest()** - Constructor for class test.CollectionUtilsTest: **columnExistsInTable(String)** - Method in class uk.ac.ed.inf.utils.database.TableReader: **columnsToString()** - Method in class uk.ac.ed.inf.utils.database.TableRow: Returns a comma separated string of the values **CommandLineMenu** - Class in uk.ac.ed.inf.utils.guiutils: Stores the options for a command line program. **CommandLineMenu(String, String, ArrayList<CommandOption>, String, String)** - Constructor for class uk.ac.ed.inf.utils.guiutils.CommandLineMenu: **CommandOption** - Class in uk.ac.ed.inf.utils.guiutils: **CommandOption(String, String)** - Constructor for class uk.ac.ed.inf.utils.guiutils.CommandOption: **compare(Object, Object)** - Method in class uk.ac.ed.inf.utils.database.TableColumn: Compares two columns by their name, returns -1 if f1 comes alphabetically before f2, 0 if the two are equal and +1 if f2 comes alphabetically before f1. **compareTo(TableColumn)** - Method in class uk.ac.ed.inf.utils.database.TableColumn: Compares two columns by their name, returns -1 if this column comes alphabetically before f2, 0 if the two are equal and +1 if f2 comes alphabetically before this column. **compareTo(Set)** - Method in class uk.ac.ed.inf.utils.setutils.Set: **connectionIsValid()** - Method in class uk.ac.ed.inf.utils.database.DbConn: **connectionIsValid(Connection)** - Static method in class uk.ac.ed.inf.utils.database.DbConn: Checks that the connection is not null **connectionIsValid()** - Method in class uk.ac.ed.inf.utils.database.DbManager: **contains(Set)** - Method in class uk.ac.ed.inf.utils.setutils.Set: **containsKey(T)** - Method in class uk.ac.ed.inf.utils.maputils.OneToManyMap: Checks if the map contains a key **containsKey(String)** - Method in class uk.ac.ed.inf.utils.maputils.TableMap: **containsKeyValue(T, U)** - Method in class uk.ac.ed.inf.utils.maputils.IndexedOneToManyMap: Checks if the map contains a specific key-value pair **containsKeyValue(T, U)** - Method in class uk.ac.ed.inf.utils.maputils.OneToManyMap: Checks if the map contains a specific key-value pair **containsKeyValue(String, String)** - Method in class uk.ac.ed.inf.utils.maputils.TableMap: **containsPositiveInteger(String)** - Static method in class uk.ac.ed.inf.utils.StringUtils: Returns true if the string contains a parsable integer **containsValue(U)** - Method in class uk.ac.ed.inf.utils.maputils.IndexedOneToManyMap: Checks if the map contains a value **containsValue(U)** - Method in class uk.ac.ed.inf.utils.maputils.OneToManyMap: Checks if the map contains a value **containsValue(String)** - Method in class uk.ac.ed.inf.utils.maputils.TableMap: **countValues(Collection, Object)** - Static method in class uk.ac.ed.inf.utils.CollectionUtils: Counts the occurrences of a given value in a collection (using the 'equals' method) **createConnection()** - Method in class uk.ac.ed.inf.utils.database.DbConn: Creates a new connection to the database **createSupersets()** - Method in class uk.ac.ed.inf.utils.setutils.SupersetsManager: **createTable()** - Method in class uk.ac.ed.inf.utils.database.TableCreator: Creates the database table for this `Table` object

---


|  |  |  |  |  |  |  |  |  |  |  |
| --- | --- | --- | --- | --- | --- | --- | --- | --- | --- | --- |
| |  |  |  |  |  |  |  |  | | --- | --- | --- | --- | --- | --- | --- | --- | | **Overview** | Package | Class | Use | **Tree** | **Deprecated** | **Index** | **Help** | | |  |
| **PREV LETTER**   **NEXT LETTER** | **FRAMES**    **NO FRAMES**     **All Classes** |


A B C D E F G H I J K L M N O P Q R S T U V W X Y 

---
