# Supplementary material for: EnzML: multi-label prediction of enzyme classes using InterPro signatures
Source: BMC Bioinformatics. 2012 Apr 25;13:61. doi: 10.1186/1471-2105-13-61 (PMC3483700; doi:10.1186/1471-2105-13-61)
Supplement: Addtional file 5 — The Java code to format the data files, evaluate and predict. The file enzml_java_code.tar.gz contains the Java code used to format database data to ARFF and XML formats, to execute cross and train-test (jackknife) evaluations and to record evaluation results to database. More information is included in the readme.txt file and the Javadoc files. The code can be used with a MySQL database. To use a different database software, other JDBC drivers might be required. [file 1471-2105-13-61-S5.gz › java_code/utils/doc/index-files/index-22.html]

V-Index


---


|  |  |  |  |  |  |  |  |  |  |  |
| --- | --- | --- | --- | --- | --- | --- | --- | --- | --- | --- |
| |  |  |  |  |  |  |  |  | | --- | --- | --- | --- | --- | --- | --- | --- | | **Overview** | Package | Class | Use | **Tree** | **Deprecated** | **Index** | **Help** | | |  |
| **PREV LETTER**   **NEXT LETTER** | **FRAMES**    **NO FRAMES**     **All Classes** |


A B C D E F G H I J K L M N O P Q R S T U V W X Y 

---


## **V**

**VAL1** - Static variable in class test.Data: **VAL2** - Static variable in class test.Data: **VAL3** - Static variable in class test.Data: **VALA** - Static variable in class test.Data: **VALB** - Static variable in class test.Data: **valueExistsInTable(String, String)** - Method in class uk.ac.ed.inf.utils.database.TableReader: Checks whether a certain row value exists in the given table. **values()** - Static method in class test.Data: **values2()** - Static method in class test.Data: **valuesExistsInTable(String, String, String, String)** - Method in class uk.ac.ed.inf.utils.database.TableReader: Checks whether a row with the two given values exists in the given table. **valuesToString()** - Method in class uk.ac.ed.inf.utils.database.TableRow: Returns a comma separated string of the values **VARCHAR\_90\_SQL\_DATATYPE** - Static variable in class uk.ac.ed.inf.utils.database.DbUtils: **vectorFrequencies(Vector<Integer>)** - Static method in class uk.ac.ed.inf.utils.stats.StatUtils: Records the freqencies with which the integers appear in the array **vectorFromList(ArrayList<String>)** - Static method in class uk.ac.ed.inf.utils.ListUtils: **VectorUtils** - Class in uk.ac.ed.inf.utils: **VectorUtils()** - Constructor for class uk.ac.ed.inf.utils.VectorUtils

---


|  |  |  |  |  |  |  |  |  |  |  |
| --- | --- | --- | --- | --- | --- | --- | --- | --- | --- | --- |
| |  |  |  |  |  |  |  |  | | --- | --- | --- | --- | --- | --- | --- | --- | | **Overview** | Package | Class | Use | **Tree** | **Deprecated** | **Index** | **Help** | | |  |
| **PREV LETTER**   **NEXT LETTER** | **FRAMES**    **NO FRAMES**     **All Classes** |


A B C D E F G H I J K L M N O P Q R S T U V W X Y 

---
