# Supplementary material for: EnzML: multi-label prediction of enzyme classes using InterPro signatures
Source: BMC Bioinformatics. 2012 Apr 25;13:61. doi: 10.1186/1471-2105-13-61 (PMC3483700; doi:10.1186/1471-2105-13-61)
Supplement: Addtional file 5 — The Java code to format the data files, evaluate and predict. The file enzml_java_code.tar.gz contains the Java code used to format database data to ARFF and XML formats, to execute cross and train-test (jackknife) evaluations and to record evaluation results to database. More information is included in the readme.txt file and the Javadoc files. The code can be used with a MySQL database. To use a different database software, other JDBC drivers might be required. [file 1471-2105-13-61-S5.gz › java_code/utils/doc/index-files/index-6.html]

F-Index


---


|  |  |  |  |  |  |  |  |  |  |  |
| --- | --- | --- | --- | --- | --- | --- | --- | --- | --- | --- |
| |  |  |  |  |  |  |  |  | | --- | --- | --- | --- | --- | --- | --- | --- | | **Overview** | Package | Class | Use | **Tree** | **Deprecated** | **Index** | **Help** | | |  |
| **PREV LETTER**   **NEXT LETTER** | **FRAMES**    **NO FRAMES**     **All Classes** |


A B C D E F G H I J K L M N O P Q R S T U V W X Y 

---


## **F**

**fileChooserWithPreMessage(String, String)** - Static method in class uk.ac.ed.inf.utils.guiutils.GuiUtils: 1. **fileNotWritablePopUp(String)** - Static method in class uk.ac.ed.inf.utils.FileUtils: Error Pop-up: file not writable **fileToArrayOfArrays(String, String, boolean)** - Static method in class uk.ac.ed.inf.utils.FileUtils: Reads a special-character-separated file (eg comma or tab separated) and creates an array of rows-columns. **fileToArrayOfLines(File)** - Static method in class uk.ac.ed.inf.utils.FileUtils: Reads a file and adds the lines to an array, adapted from: http://www.computing.net/programming/wwwboard/forum/12276.html **fileToArrayOfLines(String)** - Static method in class uk.ac.ed.inf.utils.FileUtils: Reads a file and adds the lines to an array, adapted from: http://www.computing.net/programming/wwwboard/forum/12276.html **fileTokensToArray(String, String)** - Static method in class uk.ac.ed.inf.utils.FileUtils: Reads a file and adds the tokens (identified by regular expressions) to an array (e.g. **FileUtils** - Class in uk.ac.ed.inf.utils: Utils to read, write files etc. **FileUtils()** - Constructor for class uk.ac.ed.inf.utils.FileUtils: **FileUtilsTest** - Class in test: Class **FileUtilsTest()** - Constructor for class test.FileUtilsTest: **formatPercentOneFraction(double)** - Static method in class uk.ac.ed.inf.utils.NumberUtils: Takes a double in percent returns a String with one fraction 0.85842 -> 85.8% **from1DigitTo2DigitsDate(String)** - Static method in class uk.ac.ed.inf.utils.TimeUtils: Returns a day or month number in 2 digits format. **fromArrayListToVector(ArrayList<String>)** - Static method in class uk.ac.ed.inf.utils.CollectionUtils: **fromArrayofArraysToColumnVector(String[][], int)** - Static method in class uk.ac.ed.inf.utils.ArrayUtils: Converts a column of an array of arrays into a vector **fromArrayOfArraysToFile(ArrayList<ArrayList<String>>, String, String)** - Static method in class uk.ac.ed.inf.utils.FileUtils: Writes a special-character-separated file (eg comma or tab separated) and fills it from an array of rows-columns. **fromArrayToArrayList(Object[])** - Static method in class uk.ac.ed.inf.utils.ArrayUtils: Creates an arrayList from an array **fromArrayToSet(String[])** - Static method in class uk.ac.ed.inf.utils.SetUtils: **fromArrayToVector(Object[])** - Static method in class uk.ac.ed.inf.utils.ArrayUtils: Creates a vector from an array **fromBigSetToVector(Set<String>)** - Static method in class uk.ac.ed.inf.utils.CollectionUtils: Deletes each set entry after writing it to vector, to limit memory use. **fromDMYYYtoYYYYMMDDdate(String, String, String)** - Static method in class uk.ac.ed.inf.utils.TimeUtils: Converts from D/M/YYYY date (6/19/2006) to YYYY-MM-DD date (2006-06-19) **fromDOMtoFile(Document, String)** - Static method in class uk.ac.ed.inf.utils.webutils.XMLUtils: From JTidy DOM to file **fromFilePathToBufferedReader(String)** - Static method in class uk.ac.ed.inf.utils.FileUtils: From file path to bufferedReader **fromFrequencyToPercOfFrequency(TreeMap<Integer, Integer>)** - Static method in class uk.ac.ed.inf.utils.stats.StatUtils: Converts a frequency from number of events to percentage over the total number of exctractions **fromInputStreamToString(InputStream)** - Static method in class uk.ac.ed.inf.utils.StringUtils: **fromSetToVector(Set<String>)** - Static method in class uk.ac.ed.inf.utils.CollectionUtils: **fromStringArrayToArrayList(String[])** - Static method in class uk.ac.ed.inf.utils.ArrayUtils: Creates an arrayList from an array **fromStringToSet(String, String)** - Static method in class uk.ac.ed.inf.utils.SetUtils: **fromTwoVectorsToMap(Vector<String>, Vector<String>)** - Static method in class uk.ac.ed.inf.utils.maputils.MapUtils: Creates an hashmap from 2 vectors.

---


|  |  |  |  |  |  |  |  |  |  |  |
| --- | --- | --- | --- | --- | --- | --- | --- | --- | --- | --- |
| |  |  |  |  |  |  |  |  | | --- | --- | --- | --- | --- | --- | --- | --- | | **Overview** | Package | Class | Use | **Tree** | **Deprecated** | **Index** | **Help** | | |  |
| **PREV LETTER**   **NEXT LETTER** | **FRAMES**    **NO FRAMES**     **All Classes** |


A B C D E F G H I J K L M N O P Q R S T U V W X Y 

---
