# Supplementary material for: EnzML: multi-label prediction of enzyme classes using InterPro signatures
Source: BMC Bioinformatics. 2012 Apr 25;13:61. doi: 10.1186/1471-2105-13-61 (PMC3483700; doi:10.1186/1471-2105-13-61)
Supplement: Addtional file 5 — The Java code to format the data files, evaluate and predict. The file enzml_java_code.tar.gz contains the Java code used to format database data to ARFF and XML formats, to execute cross and train-test (jackknife) evaluations and to record evaluation results to database. More information is included in the readme.txt file and the Javadoc files. The code can be used with a MySQL database. To use a different database software, other JDBC drivers might be required. [file 1471-2105-13-61-S5.gz › java_code/utils/doc/index-files/index-24.html]

X-Index


---


|  |  |  |  |  |  |  |  |  |  |  |
| --- | --- | --- | --- | --- | --- | --- | --- | --- | --- | --- |
| |  |  |  |  |  |  |  |  | | --- | --- | --- | --- | --- | --- | --- | --- | | **Overview** | Package | Class | Use | **Tree** | **Deprecated** | **Index** | **Help** | | |  |
| **PREV LETTER**   **NEXT LETTER** | **FRAMES**    **NO FRAMES**     **All Classes** |


A B C D E F G H I J K L M N O P Q R S T U V W X Y 

---


## **X**

**xmlElementToString(XmlNode)** - Static method in class uk.ac.ed.inf.utils.EntrezUtils: Test method to print to string a single Entrez eSummary xml element **XmlNode** - Class in uk.ac.ed.inf.utils.webutils.simpledomparser: `SimpleElement` is the only node type for simplified DOM model. **XmlNode(String)** - Constructor for class uk.ac.ed.inf.utils.webutils.simpledomparser.XmlNode: **XmlNodeTest** - Class in test: Class **XmlNodeTest()** - Constructor for class test.XmlNodeTest: **XmlSearcher** - Class in uk.ac.ed.inf.utils.webutils.simpledomparser: Executes a search over a SimpleElement (an XML tree) **XmlSearcher()** - Constructor for class uk.ac.ed.inf.utils.webutils.simpledomparser.XmlSearcher: **XmlSearcherTest** - Class in test: Class **XmlSearcherTest()** - Constructor for class test.XmlSearcherTest: **XMLUtils** - Class in uk.ac.ed.inf.utils.webutils: XML utilities. **XMLUtils()** - Constructor for class uk.ac.ed.inf.utils.webutils.XMLUtils: **XmlUtilsTest** - Class in test: Class **XmlUtilsTest()** - Constructor for class test.XmlUtilsTest

---


|  |  |  |  |  |  |  |  |  |  |  |
| --- | --- | --- | --- | --- | --- | --- | --- | --- | --- | --- |
| |  |  |  |  |  |  |  |  | | --- | --- | --- | --- | --- | --- | --- | --- | | **Overview** | Package | Class | Use | **Tree** | **Deprecated** | **Index** | **Help** | | |  |
| **PREV LETTER**   **NEXT LETTER** | **FRAMES**    **NO FRAMES**     **All Classes** |


A B C D E F G H I J K L M N O P Q R S T U V W X Y 

---
