# Supplementary material for: EnzML: multi-label prediction of enzyme classes using InterPro signatures
Source: BMC Bioinformatics. 2012 Apr 25;13:61. doi: 10.1186/1471-2105-13-61 (PMC3483700; doi:10.1186/1471-2105-13-61)
Supplement: Addtional file 5 — The Java code to format the data files, evaluate and predict. The file enzml_java_code.tar.gz contains the Java code used to format database data to ARFF and XML formats, to execute cross and train-test (jackknife) evaluations and to record evaluation results to database. More information is included in the readme.txt file and the Javadoc files. The code can be used with a MySQL database. To use a different database software, other JDBC drivers might be required. [file 1471-2105-13-61-S5.gz › java_code/utils/doc/index-files/index-16.html]

P-Index


---


|  |  |  |  |  |  |  |  |  |  |  |
| --- | --- | --- | --- | --- | --- | --- | --- | --- | --- | --- |
| |  |  |  |  |  |  |  |  | | --- | --- | --- | --- | --- | --- | --- | --- | | **Overview** | Package | Class | Use | **Tree** | **Deprecated** | **Index** | **Help** | | |  |
| **PREV LETTER**   **NEXT LETTER** | **FRAMES**    **NO FRAMES**     **All Classes** |


A B C D E F G H I J K L M N O P Q R S T U V W X Y 

---


## **P**

**Pareto** - Class in cern.jet.random: Pareto Distribution. **Pareto(double, double, RandomElement)** - Constructor for class cern.jet.random.Pareto: Constructs a Pareto distribution. **paretoFrequencies(Pareto, int)** - Static method in class uk.ac.ed.inf.utils.stats.StatUtils: Extracts numbers from a pareto distribution for the given number of times and records the freqencies with which the integers have been extracted **ParetoTest** - Class in uk.ac.ed.inf.utils.stats.tests: Class **ParetoTest()** - Constructor for class uk.ac.ed.inf.utils.stats.tests.ParetoTest: **parse(Reader)** - Method in class uk.ac.ed.inf.utils.webutils.simpledomparser.SimpleDOMParser: **parseAndGetSimpleElementTest()** - Static method in class test.SimpleDOMParserTest: **PASSWORD\_PROP** - Static variable in class uk.ac.ed.inf.utils.database.DbConn: **PathUtils** - Class in uk.ac.ed.inf.utils: Paths to utilities folders and files **PathUtils()** - Constructor for class uk.ac.ed.inf.utils.PathUtils: **pdf(double)** - Method in class cern.jet.random.Pareto: Returns the probability distribution function. **PersistentObject** - Class in cern.colt: This empty class is the common root for all persistent capable classes. **PORT\_PROP** - Static variable in class uk.ac.ed.inf.utils.database.DbConn: **post()** - Method in class uk.ac.ed.inf.utils.webutils.ClientHttpRequestUtils: posts the requests to the server, with all the cookies and parameters that were added **post(Map)** - Method in class uk.ac.ed.inf.utils.webutils.ClientHttpRequestUtils: posts the requests to the server, with all the cookies and parameters that were added before (if any), and with parameters that are passed in the argument **post(Map, Map)** - Method in class uk.ac.ed.inf.utils.webutils.ClientHttpRequestUtils: posts the requests to the server, with all the cookies and parameters that were added before (if any), and with cookies and parameters that are passed in the arguments **post(Object[])** - Method in class uk.ac.ed.inf.utils.webutils.ClientHttpRequestUtils: posts the requests to the server, with all the cookies and parameters that were added before (if any), and with parameters that are passed in the argument **post(String, Object)** - Method in class uk.ac.ed.inf.utils.webutils.ClientHttpRequestUtils: post the POST request to the server, with the specified parameter **post(String, Object, String, Object)** - Method in class uk.ac.ed.inf.utils.webutils.ClientHttpRequestUtils: post the POST request to the server, with the specified parameters **post(String, Object, String, Object, String, Object)** - Method in class uk.ac.ed.inf.utils.webutils.ClientHttpRequestUtils: post the POST request to the server, with the specified parameters **post(String, Object, String, Object, String, Object, String, Object)** - Method in class uk.ac.ed.inf.utils.webutils.ClientHttpRequestUtils: post the POST request to the server, with the specified parameters **post(String[], Object[])** - Method in class uk.ac.ed.inf.utils.webutils.ClientHttpRequestUtils: posts the requests to the server, with all the cookies and parameters that were added before (if any), and with cookies and parameters that are passed in the arguments **post(URL, Map)** - Static method in class uk.ac.ed.inf.utils.webutils.ClientHttpRequestUtils: posts a new request to specified URL, with parameters that are passed in the argument **post(URL, Map, Map)** - Static method in class uk.ac.ed.inf.utils.webutils.ClientHttpRequestUtils: posts a new request to specified URL, with cookies and parameters that are passed in the argument **post(URL, Object[])** - Static method in class uk.ac.ed.inf.utils.webutils.ClientHttpRequestUtils: posts a new request to specified URL, with parameters that are passed in the argument **post(URL, String, Object)** - Static method in class uk.ac.ed.inf.utils.webutils.ClientHttpRequestUtils: post the POST request specified URL, with the specified parameter **post(URL, String, Object, String, Object)** - Static method in class uk.ac.ed.inf.utils.webutils.ClientHttpRequestUtils: post the POST request to specified URL, with the specified parameters **post(URL, String, Object, String, Object, String, Object)** - Static method in class uk.ac.ed.inf.utils.webutils.ClientHttpRequestUtils: post the POST request to specified URL, with the specified parameters **post(URL, String, Object, String, Object, String, Object, String, Object)** - Static method in class uk.ac.ed.inf.utils.webutils.ClientHttpRequestUtils: post the POST request to specified URL, with the specified parameters **post(URL, String[], Object[])** - Static method in class uk.ac.ed.inf.utils.webutils.ClientHttpRequestUtils: posts a new request to specified URL, with cookies and parameters that are passed in the argument **powlaw(double, double)** - Method in class edu.cornell.lassp.houle.RngPack.RandomElement: generate a power-law distribution with exponent `alpha` and lower cutoff `cut` **printAllPaths(File)** - Static method in class uk.ac.ed.inf.utils.FileUtils: Test method to print all absolute/relative paths of a file **printArray(String[])** - Static method in class uk.ac.ed.inf.utils.ArrayUtils: Prints an array (String[]) **printArrayOfArrays(String[][])** - Static method in class uk.ac.ed.inf.utils.ArrayUtils: Prints an array of arrays (String[][]) **printArrayOfObjects(Object[])** - Static method in class uk.ac.ed.inf.utils.ArrayUtils: Prints an array of objects **printFromReader(BufferedReader)** - Static method in class uk.ac.ed.inf.utils.FileUtils: Prints all lines from a reader. **printList(List)** - Static method in class uk.ac.ed.inf.utils.ListUtils: Prints an array as a list of 'index' + 'array element' (with a header containing the size) **printListsOfLists(List<List>)** - Static method in class uk.ac.ed.inf.utils.ListUtils: Prints lists of lists **printMap(Map)** - Static method in class uk.ac.ed.inf.utils.maputils.MapUtils: Prints an HashMap as a list of index + key + value **printResultsSet(ResultSet)** - Static method in class uk.ac.ed.inf.utils.database.DbUtils: Prints the results of a query **printTimestampedComment(String)** - Static method in class uk.ac.ed.inf.utils.TimeUtils: Writes to System.out a timestamp + tab + the comment. **PropertiesUtils** - Class in uk.ac.ed.inf.utils: Utilities to manipulate Java properties and properties text files **PropertiesUtils()** - Constructor for class uk.ac.ed.inf.utils.PropertiesUtils: **PseudoTruncatedPareto** - Class in uk.ac.ed.inf.utils.stats: A truncated pareto distribution which stores a big lists of power-law extracted random numbers. **PseudoTruncatedPareto(double, double, int, int, int)** - Constructor for class uk.ac.ed.inf.utils.stats.PseudoTruncatedPareto: **PseudoTruncatedParetoSingleton** - Class in uk.ac.ed.inf.utils.stats: Class **PseudoTruncatedParetoTest** - Class in uk.ac.ed.inf.utils.stats.tests: Class **PseudoTruncatedParetoTest()** - Constructor for class uk.ac.ed.inf.utils.stats.tests.PseudoTruncatedParetoTest: **put(String, String)** - Method in class uk.ac.ed.inf.utils.database.TableRow: Puts an entry in the table record map

---


|  |  |  |  |  |  |  |  |  |  |  |
| --- | --- | --- | --- | --- | --- | --- | --- | --- | --- | --- |
| |  |  |  |  |  |  |  |  | | --- | --- | --- | --- | --- | --- | --- | --- | | **Overview** | Package | Class | Use | **Tree** | **Deprecated** | **Index** | **Help** | | |  |
| **PREV LETTER**   **NEXT LETTER** | **FRAMES**    **NO FRAMES**     **All Classes** |


A B C D E F G H I J K L M N O P Q R S T U V W X Y 

---
