# Supplementary material for: EnzML: multi-label prediction of enzyme classes using InterPro signatures
Source: BMC Bioinformatics. 2012 Apr 25;13:61. doi: 10.1186/1471-2105-13-61 (PMC3483700; doi:10.1186/1471-2105-13-61)
Supplement: Addtional file 5 — The Java code to format the data files, evaluate and predict. The file enzml_java_code.tar.gz contains the Java code used to format database data to ARFF and XML formats, to execute cross and train-test (jackknife) evaluations and to record evaluation results to database. More information is included in the readme.txt file and the Javadoc files. The code can be used with a MySQL database. To use a different database software, other JDBC drivers might be required. [file 1471-2105-13-61-S5.gz › java_code/utils/doc/index-files/index-15.html]

O-Index


---


|  |  |  |  |  |  |  |  |  |  |  |
| --- | --- | --- | --- | --- | --- | --- | --- | --- | --- | --- |
| |  |  |  |  |  |  |  |  | | --- | --- | --- | --- | --- | --- | --- | --- | | **Overview** | Package | Class | Use | **Tree** | **Deprecated** | **Index** | **Help** | | |  |
| **PREV LETTER**   **NEXT LETTER** | **FRAMES**    **NO FRAMES**     **All Classes** |


A B C D E F G H I J K L M N O P Q R S T U V W X Y 

---


## **O**

**OneToManyMap**<T,U> - Class in uk.ac.ed.inf.utils.maputils: A map containing for each key a set of values. **OneToManyMap()** - Constructor for class uk.ac.ed.inf.utils.maputils.OneToManyMap: Initialise an empty map **OneToManyMap(Vector<T>, Vector<U>)** - Constructor for class uk.ac.ed.inf.utils.maputils.OneToManyMap: Initialise with a list of key-value pairs **oneToManyMap1()** - Static method in class test.maputils.IndexedOneToManyMapTest: **oneToManyMap1()** - Static method in class test.maputils.OneToManyMapTest: **oneToManyMap2()** - Static method in class test.maputils.IndexedOneToManyMapTest: **oneToManyMap2()** - Static method in class test.maputils.OneToManyMapTest: **oneToManyMapEmptyKeyValue()** - Static method in class test.maputils.IndexedOneToManyMapTest: **oneToManyMapEmptyKeyValue()** - Static method in class test.maputils.OneToManyMapTest: **OneToManyMapTest** - Class in test.maputils: Class **OneToManyMapTest()** - Constructor for class test.maputils.OneToManyMapTest: **OptionUtils** - Class in uk.ac.ed.inf.utils.guiutils: Utilities to manipulate command line options, main args etc. **OptionUtils()** - Constructor for class uk.ac.ed.inf.utils.guiutils.OptionUtils

---


|  |  |  |  |  |  |  |  |  |  |  |
| --- | --- | --- | --- | --- | --- | --- | --- | --- | --- | --- |
| |  |  |  |  |  |  |  |  | | --- | --- | --- | --- | --- | --- | --- | --- | | **Overview** | Package | Class | Use | **Tree** | **Deprecated** | **Index** | **Help** | | |  |
| **PREV LETTER**   **NEXT LETTER** | **FRAMES**    **NO FRAMES**     **All Classes** |


A B C D E F G H I J K L M N O P Q R S T U V W X Y 

---
