# Supplementary material for: EnzML: multi-label prediction of enzyme classes using InterPro signatures
Source: BMC Bioinformatics. 2012 Apr 25;13:61. doi: 10.1186/1471-2105-13-61 (PMC3483700; doi:10.1186/1471-2105-13-61)
Supplement: Addtional file 5 — The Java code to format the data files, evaluate and predict. The file enzml_java_code.tar.gz contains the Java code used to format database data to ARFF and XML formats, to execute cross and train-test (jackknife) evaluations and to record evaluation results to database. More information is included in the readme.txt file and the Javadoc files. The code can be used with a MySQL database. To use a different database software, other JDBC drivers might be required. [file 1471-2105-13-61-S5.gz › java_code/utils/doc/index-files/index-4.html]

D-Index


---


|  |  |  |  |  |  |  |  |  |  |  |
| --- | --- | --- | --- | --- | --- | --- | --- | --- | --- | --- |
| |  |  |  |  |  |  |  |  | | --- | --- | --- | --- | --- | --- | --- | --- | | **Overview** | Package | Class | Use | **Tree** | **Deprecated** | **Index** | **Help** | | |  |
| **PREV LETTER**   **NEXT LETTER** | **FRAMES**    **NO FRAMES**     **All Classes** |


A B C D E F G H I J K L M N O P Q R S T U V W X Y 

---


## **D**

**Data** - Class in test: Class **Data()** - Constructor for class test.Data: **DATA\_DATABASE\_CONNECTION** - Static variable in class test.database.DbManagerTest: **DATA\_DB\_CONN\_PATH** - Static variable in class uk.ac.ed.inf.utils.PathUtils: **DATABASE\_NAME\_PROP** - Static variable in class uk.ac.ed.inf.utils.database.DbConn: **DATABASE\_TYPE\_PROP** - Static variable in class uk.ac.ed.inf.utils.database.DbConn: **DAY** - Static variable in class uk.ac.ed.inf.utils.TimeUtils: **DB\_UTILS\_HOME** - Static variable in class uk.ac.ed.inf.utils.PathUtils: **DbConn** - Class in uk.ac.ed.inf.utils.database: A DbConnectionProperties object contains the properties needed to connect to a database through JDBC. **DbConn(String)** - Constructor for class uk.ac.ed.inf.utils.database.DbConn: Constructor: takes connection parameters from the specified properties file **DbConnPropsTest** - Class in test.database: Class **DbConnPropsTest()** - Constructor for class test.database.DbConnPropsTest: **DbCreator** - Class in uk.ac.ed.inf.utils.database: Class **DbCreator(DbManager)** - Constructor for class uk.ac.ed.inf.utils.database.DbCreator: **DbCreatorTest** - Class in test.database: Class **DbCreatorTest()** - Constructor for class test.database.DbCreatorTest: **DbManaged** - Class in uk.ac.ed.inf.utils.database: Managed is a class which has a `DbManager` (an object that coordinates its database activities) **DbManaged(DbManager)** - Constructor for class uk.ac.ed.inf.utils.database.DbManaged: **DbManager** - Class in uk.ac.ed.inf.utils.database: A database manager provides object to create a database, write and read from it. **DbManager(String)** - Constructor for class uk.ac.ed.inf.utils.database.DbManager: Constructor 1: creates the database connection and the classes for reading/writing to database **DbManagerTest** - Class in test.database: Class **DbManagerTest()** - Constructor for class test.database.DbManagerTest: **DbReader**<T,U> - Class in uk.ac.ed.inf.utils.database: Class to read from a database. **DbReader(DbManager)** - Constructor for class uk.ac.ed.inf.utils.database.DbReader: **DbReaderTest** - Class in test.database: Class **DbReaderTest()** - Constructor for class test.database.DbReaderTest: **DbUtils** - Class in uk.ac.ed.inf.utils.database: Database utilities (to create sql code etc.) **DbUtils()** - Constructor for class uk.ac.ed.inf.utils.database.DbUtils: **DbUtilsTest** - Class in test.database: Class **DbUtilsTest()** - Constructor for class test.database.DbUtilsTest: **DbWriter** - Class in uk.ac.ed.inf.utils.database: To write to a database table **DbWriter(DbManager)** - Constructor for class uk.ac.ed.inf.utils.database.DbWriter: **DbWriterTest** - Class in test.database: Class **DbWriterTest()** - Constructor for class test.database.DbWriterTest: **DEFAULT\_SEED** - Static variable in class cern.jet.random.engine.MersenneTwister: **DEFSEED** - Static variable in class edu.cornell.lassp.houle.RngPack.Ranmar: Default seed. **DEFSEED1** - Static variable in class edu.cornell.lassp.houle.RngPack.Ranecu: default iseed1 = 12345 **DEFSEED2** - Static variable in class edu.cornell.lassp.houle.RngPack.Ranecu: default iseed2 = 67890 **deleteFile(String)** - Static method in class uk.ac.ed.inf.utils.FileUtils: Delete a file **deleteKeyValue(T, U)** - Method in class uk.ac.ed.inf.utils.maputils.OneToManyMap: **deleteKeyValue(String, String)** - Method in class uk.ac.ed.inf.utils.maputils.TableMap: Delete a key-value pair from the one to many map **depthFirstSearch(XmlNode, String, String, File)** - Static method in class uk.ac.ed.inf.utils.webutils.simpledomparser.XmlSearcher: Searches this tree in a depth first mode and returns the first node whose tag, text or attribute value matches the given string. **depthFirstSearch(XmlNode, String, String, File, int, int)** - Static method in class uk.ac.ed.inf.utils.webutils.simpledomparser.XmlSearcher: Searches this tree in a depth first mode and returns the first node whose tag, text or attribute value matches the given string. **depthFirstSearchOfParent(XmlNode, String, String)** - Static method in class uk.ac.ed.inf.utils.webutils.simpledomparser.XmlSearcher: **depthFirstSearchOfParent(XmlNode, String, String, File, int, int)** - Static method in class uk.ac.ed.inf.utils.webutils.simpledomparser.XmlSearcher: TODO duplicated code! depthFirstSearch ~ depthFirstSearchOfParent Searches this tree in a depth first mode and returns the \*parent\* of the first node whose tag, text or attribute value matches the given string. **Diff** - Class in uk.ac.ed.inf.utils.diff: Compares two collections, returning a list of the additions, changes, and deletions between them. **Diff(Collection, Collection)** - Constructor for class uk.ac.ed.inf.utils.diff.Diff: Constructs the Diff object for the two collections, using the default comparison mechanism between the objects, such as `equals` and `compareTo`. **Diff(Collection, Collection, Comparator)** - Constructor for class uk.ac.ed.inf.utils.diff.Diff: Constructs the Diff object for the two collections, using the given comparator. **Diff(Object[], Object[])** - Constructor for class uk.ac.ed.inf.utils.diff.Diff: Constructs the Diff object for the two arrays, using the default comparison mechanism between the objects, such as `equals` and `compareTo`. **Diff(Object[], Object[], Comparator)** - Constructor for class uk.ac.ed.inf.utils.diff.Diff: Constructs the Diff object for the two arrays, using the given comparator. **diff()** - Method in class uk.ac.ed.inf.utils.diff.Diff: Runs diff and returns the results. **Difference** - Class in uk.ac.ed.inf.utils.diff: Represents a difference, as used in `Diff`. **Difference(int, int, int, int)** - Constructor for class uk.ac.ed.inf.utils.diff.Difference: Creates the difference for the given start and end points for the deletion and addition. **DOUBLE** - Static variable in class uk.ac.ed.inf.utils.database.SqlUtils: Basic data type: double (passes without error a parseDouble action) **DOUBLE\_QUOTE\_CHARACTER** - Static variable in class uk.ac.ed.inf.utils.database.DbUtils: **DOUBLE\_SQL\_DATATYPE** - Static variable in class uk.ac.ed.inf.utils.database.DbUtils: **DoubleFunction** - Interface in cern.colt.function: Interface that represents a function object: a function that takes a single argument and returns a single value. **doubleTo2DecimalsString(Double)** - Static method in class uk.ac.ed.inf.utils.NumberUtils: Get a percentage representation of a double **dropCreateTable()** - Method in class uk.ac.ed.inf.utils.database.TableCreator: Drops and creates table corresponding to this Table object. **dropTable(String)** - Method in class uk.ac.ed.inf.utils.database.DbCreator: Drops the database table. **dropTable()** - Method in class uk.ac.ed.inf.utils.database.TableCreator: Drops the database table for this Table object.

---


|  |  |  |  |  |  |  |  |  |  |  |
| --- | --- | --- | --- | --- | --- | --- | --- | --- | --- | --- |
| |  |  |  |  |  |  |  |  | | --- | --- | --- | --- | --- | --- | --- | --- | | **Overview** | Package | Class | Use | **Tree** | **Deprecated** | **Index** | **Help** | | |  |
| **PREV LETTER**   **NEXT LETTER** | **FRAMES**    **NO FRAMES**     **All Classes** |


A B C D E F G H I J K L M N O P Q R S T U V W X Y 

---
