# Supplementary material for: EnzML: multi-label prediction of enzyme classes using InterPro signatures
Source: BMC Bioinformatics. 2012 Apr 25;13:61. doi: 10.1186/1471-2105-13-61 (PMC3483700; doi:10.1186/1471-2105-13-61)
Supplement: Addtional file 5 — The Java code to format the data files, evaluate and predict. The file enzml_java_code.tar.gz contains the Java code used to format database data to ARFF and XML formats, to execute cross and train-test (jackknife) evaluations and to record evaluation results to database. More information is included in the readme.txt file and the Javadoc files. The code can be used with a MySQL database. To use a different database software, other JDBC drivers might be required. [file 1471-2105-13-61-S5.gz › java_code/utils/doc/index-files/index-12.html]

L-Index


---


|  |  |  |  |  |  |  |  |  |  |  |
| --- | --- | --- | --- | --- | --- | --- | --- | --- | --- | --- |
| |  |  |  |  |  |  |  |  | | --- | --- | --- | --- | --- | --- | --- | --- | | **Overview** | Package | Class | Use | **Tree** | **Deprecated** | **Index** | **Help** | | |  |
| **PREV LETTER**   **NEXT LETTER** | **FRAMES**    **NO FRAMES**     **All Classes** |


A B C D E F G H I J K L M N O P Q R S T U V W X Y 

---


## **L**

**list()** - Static method in class test.ListUtilsTest: **listFromVector(Vector<String>)** - Static method in class uk.ac.ed.inf.utils.VectorUtils: **ListUtils** - Class in uk.ac.ed.inf.utils: Utilities to manipulate lists **ListUtils()** - Constructor for class uk.ac.ed.inf.utils.ListUtils: **ListUtilsTest** - Class in test: Class **ListUtilsTest()** - Constructor for class test.ListUtilsTest: **log(Object)** - Static method in class uk.ac.ed.inf.utils.LogUtils: **log(Object, String)** - Static method in class uk.ac.ed.inf.utils.LogUtils: **logAndPrint(File, String)** - Static method in class uk.ac.ed.inf.utils.LogUtils: Logs some text to file AND prints it to screen. **LoggerCreator** - Class in uk.ac.ed.inf.utils: **LogUtils** - Class in uk.ac.ed.inf.utils: Utilities to log error messages **LogUtils()** - Constructor for class uk.ac.ed.inf.utils.LogUtils: **lxdflt** - Static variable in class edu.cornell.lassp.houle.RngPack.Ranlux: Default luxury level: `lxdflt=3`

---


|  |  |  |  |  |  |  |  |  |  |  |
| --- | --- | --- | --- | --- | --- | --- | --- | --- | --- | --- |
| |  |  |  |  |  |  |  |  | | --- | --- | --- | --- | --- | --- | --- | --- | | **Overview** | Package | Class | Use | **Tree** | **Deprecated** | **Index** | **Help** | | |  |
| **PREV LETTER**   **NEXT LETTER** | **FRAMES**    **NO FRAMES**     **All Classes** |


A B C D E F G H I J K L M N O P Q R S T U V W X Y 

---
