# Supplementary material for: EnzML: multi-label prediction of enzyme classes using InterPro signatures
Source: BMC Bioinformatics. 2012 Apr 25;13:61. doi: 10.1186/1471-2105-13-61 (PMC3483700; doi:10.1186/1471-2105-13-61)
Supplement: Addtional file 5 — The Java code to format the data files, evaluate and predict. The file enzml_java_code.tar.gz contains the Java code used to format database data to ARFF and XML formats, to execute cross and train-test (jackknife) evaluations and to record evaluation results to database. More information is included in the readme.txt file and the Javadoc files. The code can be used with a MySQL database. To use a different database software, other JDBC drivers might be required. [file 1471-2105-13-61-S5.gz › java_code/utils/doc/index-files/index-21.html]

U-Index


---


|  |  |  |  |  |  |  |  |  |  |  |
| --- | --- | --- | --- | --- | --- | --- | --- | --- | --- | --- |
| |  |  |  |  |  |  |  |  | | --- | --- | --- | --- | --- | --- | --- | --- | | **Overview** | Package | Class | Use | **Tree** | **Deprecated** | **Index** | **Help** | | |  |
| **PREV LETTER**   **NEXT LETTER** | **FRAMES**    **NO FRAMES**     **All Classes** |


A B C D E F G H I J K L M N O P Q R S T U V W X Y 

---


## **U**

**uk.ac.ed.inf.utils** - package uk.ac.ed.inf.utils: **uk.ac.ed.inf.utils.database** - package uk.ac.ed.inf.utils.database: **uk.ac.ed.inf.utils.diff** - package uk.ac.ed.inf.utils.diff: **uk.ac.ed.inf.utils.guiutils** - package uk.ac.ed.inf.utils.guiutils: **uk.ac.ed.inf.utils.maputils** - package uk.ac.ed.inf.utils.maputils: **uk.ac.ed.inf.utils.setutils** - package uk.ac.ed.inf.utils.setutils: **uk.ac.ed.inf.utils.stats** - package uk.ac.ed.inf.utils.stats: **uk.ac.ed.inf.utils.stats.tests** - package uk.ac.ed.inf.utils.stats.tests: **uk.ac.ed.inf.utils.webutils** - package uk.ac.ed.inf.utils.webutils: **uk.ac.ed.inf.utils.webutils.simpledomparser** - package uk.ac.ed.inf.utils.webutils.simpledomparser: **uniform(double, double)** - Method in class edu.cornell.lassp.houle.RngPack.RandomElement: **UniformRandomSingleton** - Class in uk.ac.ed.inf.utils.stats: Class **UniformRandomSingletonTest** - Class in uk.ac.ed.inf.utils.stats.tests: Class **UniformRandomSingletonTest()** - Constructor for class uk.ac.ed.inf.utils.stats.tests.UniformRandomSingletonTest: **UniformRandomUtils** - Class in uk.ac.ed.inf.utils.stats: Utilities for extracting with uniform random distribution **UniformRandomUtils()** - Constructor for class uk.ac.ed.inf.utils.stats.UniformRandomUtils: **UniformRandomUtilsTest** - Class in uk.ac.ed.inf.utils.stats.tests: Class **UniformRandomUtilsTest()** - Constructor for class uk.ac.ed.inf.utils.stats.tests.UniformRandomUtilsTest: **USER\_PROP** - Static variable in class uk.ac.ed.inf.utils.database.DbConn: **Utils** - Class in uk.ac.ed.inf.utils: Various utilities to manipulate vectors of numbers, get a timestamp etc. **Utils()** - Constructor for class uk.ac.ed.inf.utils.Utils: **UTILS\_DATA** - Static variable in class uk.ac.ed.inf.utils.PathUtils: **UTILS\_HOME** - Static variable in class uk.ac.ed.inf.utils.PathUtils: **UtilsTest** - Class in test: Class **UtilsTest()** - Constructor for class test.UtilsTest

---


|  |  |  |  |  |  |  |  |  |  |  |
| --- | --- | --- | --- | --- | --- | --- | --- | --- | --- | --- |
| |  |  |  |  |  |  |  |  | | --- | --- | --- | --- | --- | --- | --- | --- | | **Overview** | Package | Class | Use | **Tree** | **Deprecated** | **Index** | **Help** | | |  |
| **PREV LETTER**   **NEXT LETTER** | **FRAMES**    **NO FRAMES**     **All Classes** |


A B C D E F G H I J K L M N O P Q R S T U V W X Y 

---
