# Supplementary material for: EnzML: multi-label prediction of enzyme classes using InterPro signatures
Source: BMC Bioinformatics. 2012 Apr 25;13:61. doi: 10.1186/1471-2105-13-61 (PMC3483700; doi:10.1186/1471-2105-13-61)
Supplement: Addtional file 5 — The Java code to format the data files, evaluate and predict. The file enzml_java_code.tar.gz contains the Java code used to format database data to ARFF and XML formats, to execute cross and train-test (jackknife) evaluations and to record evaluation results to database. More information is included in the readme.txt file and the Javadoc files. The code can be used with a MySQL database. To use a different database software, other JDBC drivers might be required. [file 1471-2105-13-61-S5.gz › java_code/utils/doc/index-files/index-18.html]

R-Index


---


|  |  |  |  |  |  |  |  |  |  |  |
| --- | --- | --- | --- | --- | --- | --- | --- | --- | --- | --- |
| |  |  |  |  |  |  |  |  | | --- | --- | --- | --- | --- | --- | --- | --- | | **Overview** | Package | Class | Use | **Tree** | **Deprecated** | **Index** | **Help** | | |  |
| **PREV LETTER**   **NEXT LETTER** | **FRAMES**    **NO FRAMES**     **All Classes** |


A B C D E F G H I J K L M N O P Q R S T U V W X Y 

---


## **R**

**RandomApp** - Class in edu.cornell.lassp.houle.RngPack: RandomApp is a simple application that demonstrates the use of RngPack. **RandomApp()** - Constructor for class edu.cornell.lassp.houle.RngPack.RandomApp: **RandomElement** - Class in edu.cornell.lassp.houle.RngPack: RandomElement is an abstract class that encapsulates uniform random number generators. **RandomElement()** - Constructor for class edu.cornell.lassp.houle.RngPack.RandomElement: **RandomEngine** - Class in cern.jet.random.engine: Abstract base class for uniform pseudo-random number generating engines. **RandomJava** - Class in edu.cornell.lassp.houle.RngPack: RandomJava is a class wrapper for the `Math.random()` generator that comes with Java. **RandomJava()** - Constructor for class edu.cornell.lassp.houle.RngPack.RandomJava: **RandomSeedable** - Class in edu.cornell.lassp.houle.RngPack: `RandomSeedable` is an abstract class that extends the `RandomElement` class to include the ability to automatically generate a valid `long` seed from the clock. **RandomSeedable()** - Constructor for class edu.cornell.lassp.houle.RngPack.RandomSeedable: **RandomShuffle** - Class in edu.cornell.lassp.houle.RngPack: RandomShuffle uses one random number generator to shuffle the numbers produced by another to obliterate sequential correlations. **RandomShuffle(RandomElement, RandomElement, int)** - Constructor for class edu.cornell.lassp.houle.RngPack.RandomShuffle: **RandomUtilsTest** - Class in uk.ac.ed.inf.utils.stats.tests: Class **RandomUtilsTest()** - Constructor for class uk.ac.ed.inf.utils.stats.tests.RandomUtilsTest: **Ranecu** - Class in edu.cornell.lassp.houle.RngPack: Ranecu is an advanced multiplicative linear congruential random number generator with a period of aproximately 1018. **Ranecu()** - Constructor for class edu.cornell.lassp.houle.RngPack.Ranecu: Initialize RANECU with the default seeds from James. **Ranecu(Date)** - Constructor for class edu.cornell.lassp.houle.RngPack.Ranecu: **Ranecu(int, int)** - Constructor for class edu.cornell.lassp.houle.RngPack.Ranecu: Initialize RANECU with two specified integer seeds. **Ranecu(long)** - Constructor for class edu.cornell.lassp.houle.RngPack.Ranecu: **Ranlux** - Class in edu.cornell.lassp.houle.RngPack: RANLUX is an advanced pseudo-random number generator based on the RCARRY algorithm proposed in 1991 by Marsaglia and Zaman. **Ranlux()** - Constructor for class edu.cornell.lassp.houle.RngPack.Ranlux: Default initialization of RANLUX. **Ranlux(Date)** - Constructor for class edu.cornell.lassp.houle.RngPack.Ranlux: Initialize RANLUX with default luxury level and a Date object. **Ranlux(int)** - Constructor for class edu.cornell.lassp.houle.RngPack.Ranlux: Initialize RANLUX with default luxury level and a specified seed. **Ranlux(int, Date)** - Constructor for class edu.cornell.lassp.houle.RngPack.Ranlux: Initialize RANLUX with specified luxury level and a Date object. **Ranlux(int, int)** - Constructor for class edu.cornell.lassp.houle.RngPack.Ranlux: Initialize RANLUX with specified luxury level and seed. **Ranlux(int, long)** - Constructor for class edu.cornell.lassp.houle.RngPack.Ranlux: Initialize RANLUX with specified luxury level and seed. **Ranlux(long)** - Constructor for class edu.cornell.lassp.houle.RngPack.Ranlux: Initialize RANLUX with default luxury level and a specified seed. **Ranmar** - Class in edu.cornell.lassp.houle.RngPack: RANMAR is a lagged Fibonacci generator proposed by Marsaglia and Zaman and is a good research grade generator. **Ranmar()** - Constructor for class edu.cornell.lassp.houle.RngPack.Ranmar: Initialize Ranmar with a default seed taken from Marsaglia and Zaman's paper. **Ranmar(Date)** - Constructor for class edu.cornell.lassp.houle.RngPack.Ranmar: Seed RANMAR from the clock. **Ranmar(int)** - Constructor for class edu.cornell.lassp.houle.RngPack.Ranmar: Initialize Ranmar with a specified integer seed **Ranmar(long)** - Constructor for class edu.cornell.lassp.houle.RngPack.Ranmar: Initialize Ranmar with a specified long seed **raw()** - Method in class cern.jet.random.engine.RandomEngine: Returns a 32 bit uniformly distributed random number in the open unit interval `(0.0,1.0)` (excluding 0.0 and 1.0). **raw()** - Method in class edu.cornell.lassp.houle.RngPack.RandomElement: Returns a uniformly distributed pseudo-random number. **raw(double[])** - Method in class edu.cornell.lassp.houle.RngPack.RandomElement: Fill an entire array with doubles. **raw(double[], int)** - Method in class edu.cornell.lassp.houle.RngPack.RandomElement: Fill part or all of an array with doubles. **raw()** - Method in class edu.cornell.lassp.houle.RngPack.RandomJava: Wrapper for `Math.random().` **raw()** - Method in class edu.cornell.lassp.houle.RngPack.RandomShuffle: The generator. **raw()** - Method in class edu.cornell.lassp.houle.RngPack.Ranecu: **raw(double[], int)** - Method in class edu.cornell.lassp.houle.RngPack.Ranecu: This is an inline version that returns an array of doubles for speed. **raw()** - Method in class edu.cornell.lassp.houle.RngPack.Ranlux: Returns a uniformly distributed pseudo-random double in the range (0,1). **raw()** - Method in class edu.cornell.lassp.houle.RngPack.Ranmar: Returns a uniformly distributed pseudo-random double in the range (0,1). **raw(double[], int)** - Method in class edu.cornell.lassp.houle.RngPack.Ranmar: A version of the generator for filling arrays, inlined for speed **readConnectionProperties(String)** - Method in class uk.ac.ed.inf.utils.database.DbConn: Reads the connection properties from the java properties file specified in the path **readLocalProperties(String)** - Static method in class uk.ac.ed.inf.utils.PropertiesUtils: Reads properties that inherit from three locations. **readLocalProperties(String)** - Static method in class uk.ac.ed.inf.utils.Utils: Reads properties that inherit from three locations. **readProperties(String)** - Static method in class uk.ac.ed.inf.utils.PropertiesUtils: Reads properties Code adapted from Weka: http://www.cs.waikato.ac.nz/ml/weka/ Ian H. **readProperties(String)** - Static method in class uk.ac.ed.inf.utils.Utils: Reads properties Code adapted from Weka: http://www.cs.waikato.ac.nz/ml/weka/ Ian H. **redirectStdErr(File)** - Static method in class uk.ac.ed.inf.utils.LogUtils: Redirect stderr to file **ReflectionUtils** - Class in uk.ac.ed.inf.utils: Class **ReflectionUtils()** - Constructor for class uk.ac.ed.inf.utils.ReflectionUtils: **ReflectionUtilsTest** - Class in test: Class **ReflectionUtilsTest()** - Constructor for class test.ReflectionUtilsTest: **refreshConnection()** - Method in class uk.ac.ed.inf.utils.database.DbManager: **RegExpUtils** - Class in uk.ac.ed.inf.utils: **RegExpUtils()** - Constructor for class uk.ac.ed.inf.utils.RegExpUtils: **RegExpUtilsTest** - Class in test: Class **RegExpUtilsTest()** - Constructor for class test.RegExpUtilsTest: **removeAttribute(String)** - Method in class uk.ac.ed.inf.utils.webutils.simpledomparser.XmlNode: remove an attribute by name **removeChildNode(XmlNode)** - Method in class uk.ac.ed.inf.utils.webutils.simpledomparser.XmlNode: Remove a child element **ResultSetUtils** - Class in uk.ac.ed.inf.utils.database: Class **ResultSetUtils()** - Constructor for class uk.ac.ed.inf.utils.database.ResultSetUtils: **resultsSetToString(ResultSet)** - Static method in class uk.ac.ed.inf.utils.database.DbUtils: Generates a string for the results of a query **REVERSED\_PRIME\_CHARACTER** - Static variable in class uk.ac.ed.inf.utils.database.DbUtils: **roundDouble(double, int)** - Static method in class uk.ac.ed.inf.utils.Utils: Rounds a number to just n decimal digits **runAnalyzeTable()** - Method in class uk.ac.ed.inf.utils.database.DbManager: Runs the 'ANALYZE TABLE tablename' SQL command for all the tables in a mysql database.

---


|  |  |  |  |  |  |  |  |  |  |  |
| --- | --- | --- | --- | --- | --- | --- | --- | --- | --- | --- |
| |  |  |  |  |  |  |  |  | | --- | --- | --- | --- | --- | --- | --- | --- | | **Overview** | Package | Class | Use | **Tree** | **Deprecated** | **Index** | **Help** | | |  |
| **PREV LETTER**   **NEXT LETTER** | **FRAMES**    **NO FRAMES**     **All Classes** |


A B C D E F G H I J K L M N O P Q R S T U V W X Y 

---
