# Supplementary material for: EnzML: multi-label prediction of enzyme classes using InterPro signatures
Source: BMC Bioinformatics. 2012 Apr 25;13:61. doi: 10.1186/1471-2105-13-61 (PMC3483700; doi:10.1186/1471-2105-13-61)
Supplement: Addtional file 5 — The Java code to format the data files, evaluate and predict. The file enzml_java_code.tar.gz contains the Java code used to format database data to ARFF and XML formats, to execute cross and train-test (jackknife) evaluations and to record evaluation results to database. More information is included in the readme.txt file and the Javadoc files. The code can be used with a MySQL database. To use a different database software, other JDBC drivers might be required. [file 1471-2105-13-61-S5.gz › java_code/utils/doc/index-files/index-9.html]

I-Index


---


|  |  |  |  |  |  |  |  |  |  |  |
| --- | --- | --- | --- | --- | --- | --- | --- | --- | --- | --- |
| |  |  |  |  |  |  |  |  | | --- | --- | --- | --- | --- | --- | --- | --- | | **Overview** | Package | Class | Use | **Tree** | **Deprecated** | **Index** | **Help** | | |  |
| **PREV LETTER**   **NEXT LETTER** | **FRAMES**    **NO FRAMES**     **All Classes** |


A B C D E F G H I J K L M N O P Q R S T U V W X Y 

---


## **I**

**ID** - Static variable in class uk.ac.ed.inf.utils.database.DbUtils: **IndexedOneToManyMap**<T,U> - Class in uk.ac.ed.inf.utils.maputils: A one-to-many map that uses an internal integer index to save memory. **IndexedOneToManyMap()** - Constructor for class uk.ac.ed.inf.utils.maputils.IndexedOneToManyMap: Initialise an empty map **IndexedOneToManyMap(Vector<T>, Vector<U>)** - Constructor for class uk.ac.ed.inf.utils.maputils.IndexedOneToManyMap: Initialise with a list of key-value pairs **IndexedOneToManyMapTest** - Class in test.maputils: Class **IndexedOneToManyMapTest()** - Constructor for class test.maputils.IndexedOneToManyMapTest: **Initialisable** - Interface in uk.ac.ed.inf.utils: A class having a boolean variable telling whether the initialisation has completed correctly **initialisationIsCorrect()** - Method in interface uk.ac.ed.inf.utils.Initialisable: **initialisationIsCorrect()** - Method in class uk.ac.ed.inf.utils.Initialised: **initialise()** - Method in class uk.ac.ed.inf.utils.maputils.IndexedOneToManyMap: public TreeMap> getIndexedMap() { return m\_map; } **initialise()** - Method in class uk.ac.ed.inf.utils.maputils.OneToManyMap: **Initialised** - Class in uk.ac.ed.inf.utils: **Initialised()** - Constructor for class uk.ac.ed.inf.utils.Initialised: **insert(DbConn, String)** - Static method in class uk.ac.ed.inf.utils.database.DbUtils: Executes sql insert and returns whether the insert was successful. **insertReturnsKey(DbConn, String)** - Static method in class uk.ac.ed.inf.utils.database.DbUtils: Executes sql insert and returns the auto generated key for the first inserted row. **insertReturnsKey(TableRow)** - Method in class uk.ac.ed.inf.utils.database.TableWriter: Executes an sql 'insert into' operation. **INTEGER** - Static variable in class uk.ac.ed.inf.utils.database.SqlUtils: Basic data type: integer (passes without error a parseInteger action) **INTEGER\_SQL\_DATATYPE** - Static variable in class uk.ac.ed.inf.utils.database.DbUtils: **interactiveError(boolean, String)** - Static method in class uk.ac.ed.inf.utils.guiutils.GuiUtils: Emits a popup if the mode is interactive or writes to System.err if non interactive **interactiveMessage(boolean, String)** - Static method in class uk.ac.ed.inf.utils.guiutils.GuiUtils: Emits a popup if the mode is interactive or writes to System.out if non interactive **IntFunction** - Interface in cern.colt.function: Interface that represents a function object: a function that takes a single argument and returns a single value. **isCorrect()** - Method in class uk.ac.ed.inf.utils.database.TableRow: **isDash(String)** - Static method in class uk.ac.ed.inf.utils.StringUtils: check if the string is a dash **isEmpty(ResultSet)** - Method in class uk.ac.ed.inf.utils.database.TableReader: Returns true if the resultset is null or empty. **isNullOrEmpty(String)** - Static method in class uk.ac.ed.inf.utils.ListUtils: tells if a string is null or empty **isNullOrEmpty(Vector<String>)** - Static method in class uk.ac.ed.inf.utils.ListUtils: tells if a list is null or empty **isPrimaryKey()** - Method in class uk.ac.ed.inf.utils.database.TableColumn: Gets the column primary key status **isSubsetOfSuperset(TreeSet<String>, TreeSet<String>)** - Static method in class uk.ac.ed.inf.utils.SetUtils: **isValidConnection(Connection)** - Static method in class uk.ac.ed.inf.utils.database.DbUtils: Test Validity of JDBC Installation. **iterator1()** - Static method in class test.RegExpUtilsTest: **iterator2()** - Static method in class test.RegExpUtilsTest

---


|  |  |  |  |  |  |  |  |  |  |  |
| --- | --- | --- | --- | --- | --- | --- | --- | --- | --- | --- |
| |  |  |  |  |  |  |  |  | | --- | --- | --- | --- | --- | --- | --- | --- | | **Overview** | Package | Class | Use | **Tree** | **Deprecated** | **Index** | **Help** | | |  |
| **PREV LETTER**   **NEXT LETTER** | **FRAMES**    **NO FRAMES**     **All Classes** |


A B C D E F G H I J K L M N O P Q R S T U V W X Y 

---
