# Supplementary material for: EnzML: multi-label prediction of enzyme classes using InterPro signatures
Source: BMC Bioinformatics. 2012 Apr 25;13:61. doi: 10.1186/1471-2105-13-61 (PMC3483700; doi:10.1186/1471-2105-13-61)
Supplement: Addtional file 5 — The Java code to format the data files, evaluate and predict. The file enzml_java_code.tar.gz contains the Java code used to format database data to ARFF and XML formats, to execute cross and train-test (jackknife) evaluations and to record evaluation results to database. More information is included in the readme.txt file and the Javadoc files. The code can be used with a MySQL database. To use a different database software, other JDBC drivers might be required. [file 1471-2105-13-61-S5.gz › java_code/utils/doc/index-files/index-5.html]

E-Index


---


|  |  |  |  |  |  |  |  |  |  |  |
| --- | --- | --- | --- | --- | --- | --- | --- | --- | --- | --- |
| |  |  |  |  |  |  |  |  | | --- | --- | --- | --- | --- | --- | --- | --- | | **Overview** | Package | Class | Use | **Tree** | **Deprecated** | **Index** | **Help** | | |  |
| **PREV LETTER**   **NEXT LETTER** | **FRAMES**    **NO FRAMES**     **All Classes** |


A B C D E F G H I J K L M N O P Q R S T U V W X Y 

---


## **E**

**edu.cornell.lassp.houle.RngPack** - package edu.cornell.lassp.houle.RngPack: **elementExistsInTable(String, String, String, DbConn)** - Static method in class uk.ac.ed.inf.utils.database.DbUtils: Get if an element exists in a table column **elementsExistsInTable(String, String, String, String, String, DbConn)** - Static method in class uk.ac.ed.inf.utils.database.DbUtils: Get if two elements exists in the same table row **elementsToString()** - Method in class uk.ac.ed.inf.utils.setutils.Set: **emptyKeys()** - Static method in class test.Data: **emptyValue()** - Static method in class test.Data: **END\_TIMESTAMP** - Static variable in class uk.ac.ed.inf.utils.database.DbUtils: **EntrezUtils** - Class in uk.ac.ed.inf.utils: Utilities for Entrez services. **EntrezUtils()** - Constructor for class uk.ac.ed.inf.utils.EntrezUtils: **equals(TableColumn)** - Method in class uk.ac.ed.inf.utils.database.TableColumn: Compares two columns by their name, returns true if the two columns have the same name, false otherwise. **equals(Object)** - Method in class uk.ac.ed.inf.utils.diff.Difference: Compares this object to the other for equality. **error(Object)** - Static method in class uk.ac.ed.inf.utils.LogUtils: **error(Object, String)** - Static method in class uk.ac.ed.inf.utils.LogUtils: **error(String)** - Static method in class uk.ac.ed.inf.utils.LogUtils: **errorPopUp(String)** - Static method in class uk.ac.ed.inf.utils.guiutils.GuiUtils: Error pop-up window with message (icon: stop) **eSummaryToString(XmlNode)** - Static method in class uk.ac.ed.inf.utils.EntrezUtils: Test method to print an an Entrez eSummary xml report **evaluateCondition(XmlNode, String)** - Static method in class uk.ac.ed.inf.utils.webutils.simpledomparser.XmlSearcher: Check whether the desired string is either a tag or text for this element. **evaluateCondition(XmlNode, String, String)** - Static method in class uk.ac.ed.inf.utils.webutils.simpledomparser.XmlSearcher: Check whether the desired string is either a tag, text or an attribute \*value\* for this element. **executeQuery(String)** - Method in class uk.ac.ed.inf.utils.database.DbReader: Executes an sql query returning a result set. **executeQuery(String)** - Method in class uk.ac.ed.inf.utils.database.DbWriter: Executes any SQL update which returns some results. **executeQueryReturn1Id(String)** - Method in class uk.ac.ed.inf.utils.database.DbWriter: Executes an SQL update (an individual row insert) and returns the auto-generated key (identifier) of the first inserted row. **executeQueryReturnIds(String)** - Method in class uk.ac.ed.inf.utils.database.DbWriter: Executes an SQL update (usually a row insert) which returns the identifier(s) of the inserted rows. **executeUpdate(String)** - Method in class uk.ac.ed.inf.utils.database.DbWriter: Executes any SQL update which does not return results (create/drop table etc) **existsInTable(String, DbConn)** - Static method in class uk.ac.ed.inf.utils.database.DbUtils: **extractInteger(int)** - Static method in class uk.ac.ed.inf.utils.stats.UniformRandomUtils: Returns a random integer between 0 and (size-1) **extractMapEntry(Map, int)** - Static method in class uk.ac.ed.inf.utils.maputils.MapUtils: Extracts a random entry (key-value association) from a map **extractUniformRandomMapEntry(Map)** - Static method in class uk.ac.ed.inf.utils.stats.UniformRandomUtils: Extracts a random entry (key-value association) from a map

---


|  |  |  |  |  |  |  |  |  |  |  |
| --- | --- | --- | --- | --- | --- | --- | --- | --- | --- | --- |
| |  |  |  |  |  |  |  |  | | --- | --- | --- | --- | --- | --- | --- | --- | | **Overview** | Package | Class | Use | **Tree** | **Deprecated** | **Index** | **Help** | | |  |
| **PREV LETTER**   **NEXT LETTER** | **FRAMES**    **NO FRAMES**     **All Classes** |


A B C D E F G H I J K L M N O P Q R S T U V W X Y 

---
