# Supplementary material for: EnzML: multi-label prediction of enzyme classes using InterPro signatures
Source: BMC Bioinformatics. 2012 Apr 25;13:61. doi: 10.1186/1471-2105-13-61 (PMC3483700; doi:10.1186/1471-2105-13-61)
Supplement: Addtional file 5 — The Java code to format the data files, evaluate and predict. The file enzml_java_code.tar.gz contains the Java code used to format database data to ARFF and XML formats, to execute cross and train-test (jackknife) evaluations and to record evaluation results to database. More information is included in the readme.txt file and the Javadoc files. The code can be used with a MySQL database. To use a different database software, other JDBC drivers might be required. [file 1471-2105-13-61-S5.gz › java_code/utils/doc/index-files/index-19.html]

S-Index


---


|  |  |  |  |  |  |  |  |  |  |  |
| --- | --- | --- | --- | --- | --- | --- | --- | --- | --- | --- |
| |  |  |  |  |  |  |  |  | | --- | --- | --- | --- | --- | --- | --- | --- | | **Overview** | Package | Class | Use | **Tree** | **Deprecated** | **Index** | **Help** | | |  |
| **PREV LETTER**   **NEXT LETTER** | **FRAMES**    **NO FRAMES**     **All Classes** |


A B C D E F G H I J K L M N O P Q R S T U V W X Y 

---


## **S**

**SECOND** - Static variable in class uk.ac.ed.inf.utils.TimeUtils: **Set** - Class in uk.ac.ed.inf.utils.setutils: A set and its subsets. **Set(String, TreeSet<String>, SupersetsManager)** - Constructor for class uk.ac.ed.inf.utils.setutils.Set: **Set(String, TreeSet<String>, SupersetsManager, TreeSet<Set>)** - Constructor for class uk.ac.ed.inf.utils.setutils.Set: **setAdded(int)** - Method in class uk.ac.ed.inf.utils.diff.Difference: Sets the point as added. **setAttribute(String, String)** - Method in class uk.ac.ed.inf.utils.webutils.simpledomparser.XmlNode: **setChosenOption(String)** - Method in class uk.ac.ed.inf.utils.guiutils.SimpleRadioButtonPanel: **setCookie(String, String)** - Method in class uk.ac.ed.inf.utils.webutils.ClientHttpRequestUtils: adds a cookie to the requst **setCookies(Map)** - Method in class uk.ac.ed.inf.utils.webutils.ClientHttpRequestUtils: adds cookies to the request **setCookies(String[])** - Method in class uk.ac.ed.inf.utils.webutils.ClientHttpRequestUtils: adds cookies to the request **setDeleted(int)** - Method in class uk.ac.ed.inf.utils.diff.Difference: Sets the point as deleted. **setDiag(boolean)** - Method in class edu.cornell.lassp.houle.RngPack.Ranlux: Turns diagnostic messages on and off. **setFromList(ArrayList<String>)** - Static method in class uk.ac.ed.inf.utils.ListUtils: create a set (elements appear only once) from a list **setFromObjectsToString(TreeSet<Object>)** - Static method in class uk.ac.ed.inf.utils.Utils: **setInitialisationIsCorrect(boolean)** - Method in interface uk.ac.ed.inf.utils.Initialisable: **setInitialisationIsCorrect(boolean)** - Method in class uk.ac.ed.inf.utils.Initialised: **setIsPrimaryKey(boolean)** - Method in class uk.ac.ed.inf.utils.database.TableColumn: Sets the column primary key status **setManager(Manager)** - Method in class uk.ac.ed.inf.utils.database.Managed: **setParameter(String, File)** - Method in class uk.ac.ed.inf.utils.webutils.ClientHttpRequestUtils: adds a file parameter to the request **setParameter(String, Object)** - Method in class uk.ac.ed.inf.utils.webutils.ClientHttpRequestUtils: adds a parameter to the request; if the parameter is a File, the file is uploaded, otherwise the string value of the parameter is passed in the request **setParameter(String, String)** - Method in class uk.ac.ed.inf.utils.webutils.ClientHttpRequestUtils: adds a string parameter to the request **setParameter(String, String, InputStream)** - Method in class uk.ac.ed.inf.utils.webutils.ClientHttpRequestUtils: adds a file parameter to the request **setParameters(Map)** - Method in class uk.ac.ed.inf.utils.webutils.ClientHttpRequestUtils: adds parameters to the request **setParameters(Object[])** - Method in class uk.ac.ed.inf.utils.webutils.ClientHttpRequestUtils: adds parameters to the request **setState(double, double)** - Method in class cern.jet.random.Pareto: Sets the parameters. **setTagName(String)** - Method in class uk.ac.ed.inf.utils.webutils.simpledomparser.XmlNode: **SetTest** - Class in test.setutils: Class **SetTest()** - Constructor for class test.setutils.SetTest: **setText(String)** - Method in class uk.ac.ed.inf.utils.webutils.simpledomparser.XmlNode: **setUp()** - Method in class test.database.DbConnPropsTest: the database connection properties file for test (database = test) **setUp()** - Method in class test.database.DbCreatorTest: **setUp()** - Method in class test.database.DbManagerTest: **setUp()** - Method in class test.database.DbReaderTest: **setUp()** - Method in class test.database.DbWriterTest: **setUp()** - Method in class test.database.SqlUtilsTest: **setUp()** - Method in class test.database.TableCreatorTest: **setUp()** - Method in class test.database.TableManagerTest: **setUp()** - Method in class test.database.TableReaderTest: **setUp()** - Method in class test.database.TableRowTest: **setUp()** - Method in class test.database.TableTest: **setUp()** - Method in class test.database.TableWriterTest: **setUp()** - Method in class test.FileUtilsTest: **setUp()** - Method in class test.NumberUtilsTest: **setUp()** - Method in class test.SimpleDOMParserTest: **setUp()** - Method in class test.WebUtilsTest: **setUp()** - Method in class test.XmlNodeTest: **setUp()** - Method in class test.XmlSearcherTest: **setUp()** - Method in class test.XmlUtilsTest: **SetUtils** - Class in uk.ac.ed.inf.utils: Class **SetUtils()** - Constructor for class uk.ac.ed.inf.utils.SetUtils: **similarElementExistsInTable(String, String, String, DbConn)** - Static method in class uk.ac.ed.inf.utils.database.DbUtils: **simpleDirectoryChooser()** - Static method in class uk.ac.ed.inf.utils.guiutils.GuiUtils: Returns the directory chosen by the user in a file chooser window. **SimpleDOMParser** - Class in uk.ac.ed.inf.utils.webutils.simpledomparser: `SimpleDOMParser` is a highly-simplified XML DOM parser. **SimpleDOMParser()** - Constructor for class uk.ac.ed.inf.utils.webutils.simpledomparser.SimpleDOMParser: **SimpleDOMParserTest** - Class in test: Class **SimpleDOMParserTest()** - Constructor for class test.SimpleDOMParserTest: **simpleFileChooser()** - Static method in class uk.ac.ed.inf.utils.guiutils.GuiUtils: Returns the file chosen by the user in a file chooser window. **simpleInputDialog(String, String)** - Static method in class uk.ac.ed.inf.utils.guiutils.GuiUtils: Creates a simple input pop-up **simpleMessagePopUp(String)** - Static method in class uk.ac.ed.inf.utils.guiutils.GuiUtils: Pop-up window with message (icon: information > "i") **SimpleRadioButtonPanel** - Class in uk.ac.ed.inf.utils.guiutils: **SimpleRadioButtonPanel(String[], String[])** - Constructor for class uk.ac.ed.inf.utils.guiutils.SimpleRadioButtonPanel: Generates a simple radio button popup **SINGLE\_QUOTE\_CHARACTER** - Static variable in class uk.ac.ed.inf.utils.database.DbUtils: **size()** - Method in class uk.ac.ed.inf.utils.maputils.OneToManyMap: **splitTextInLines(String)** - Static method in class uk.ac.ed.inf.utils.RegExpUtils: I am splitting the String based on a regular expression which looks for carriage return (\r) or line feed (\n) or carriage return immediately followed by line feed. **SqlUtils** - Class in uk.ac.ed.inf.utils.database: Class **SqlUtils()** - Constructor for class uk.ac.ed.inf.utils.database.SqlUtils: **SqlUtilsTest** - Class in test.database: Class **SqlUtilsTest()** - Constructor for class test.database.SqlUtilsTest: **START\_TIMESTAMP** - Static variable in class uk.ac.ed.inf.utils.database.DbUtils: **staticNextDouble(double, double)** - Static method in class cern.jet.random.Pareto: Returns a random number from the distribution with the given scale = k, and shape = alpha. **StatUtils** - Class in uk.ac.ed.inf.utils.stats: Utilities to print simple histograms, percent of frequency etc **StatUtils()** - Constructor for class uk.ac.ed.inf.utils.stats.StatUtils: **StatUtilsTest** - Class in uk.ac.ed.inf.utils.stats.tests: Class **StatUtilsTest()** - Constructor for class uk.ac.ed.inf.utils.stats.tests.StatUtilsTest: **STRING** - Static variable in class uk.ac.ed.inf.utils.database.SqlUtils: Basic data type: string (anything that is not an integer or a double...) **stringIsNullOrEmpty(String)** - Static method in class uk.ac.ed.inf.utils.StringUtils: **stringMatchesRegExp(String, String, boolean)** - Static method in class uk.ac.ed.inf.utils.RegExpUtils: matches a string against a regular expression (gives true if there is at least a partial match) **stringToArrayOfArrays(String, String)** - Static method in class uk.ac.ed.inf.utils.ArrayUtils: Reads a special-character-separated string (eg comma or tab separated) and creates an array of rows-columns. **StringUtils** - Class in uk.ac.ed.inf.utils: Class **StringUtils()** - Constructor for class uk.ac.ed.inf.utils.StringUtils: **StringUtilsTest** - Class in test: Class **StringUtilsTest()** - Constructor for class test.StringUtilsTest: **stripXmlHtmlTags(String)** - Static method in class uk.ac.ed.inf.utils.RegExpUtils: Strips all tags from xml or html code **substituteMatchedString(String, String, String)** - Static method in class uk.ac.ed.inf.utils.RegExpUtils: Substitute an old string with a new string if the old string matches the given regular expression **suite()** - Static method in class test.AllDatabaseUtilsTests: **suite()** - Static method in class test.AllUtilsTests: **suite()** - Static method in class uk.ac.ed.inf.utils.stats.tests.AllStatsUtilsTests: **SupersetsManager** - Class in uk.ac.ed.inf.utils.setutils: A class to identify the unique supersets among sets of interpro signatures. **SupersetsManager(String, DbManager, String)** - Constructor for class uk.ac.ed.inf.utils.setutils.SupersetsManager: **SupersetsManager(TreeMap<String, String>, String)** - Constructor for class uk.ac.ed.inf.utils.setutils.SupersetsManager: **SupersetsManagerTest** - Class in test.setutils: Class **SupersetsManagerTest()** - Constructor for class test.setutils.SupersetsManagerTest: **surroundWith(String, String)** - Static method in class uk.ac.ed.inf.utils.database.SqlUtils: Adds a surrounding string (usually an apex or similar) as prefix and suffix of the given text.

---


|  |  |  |  |  |  |  |  |  |  |  |
| --- | --- | --- | --- | --- | --- | --- | --- | --- | --- | --- |
| |  |  |  |  |  |  |  |  | | --- | --- | --- | --- | --- | --- | --- | --- | | **Overview** | Package | Class | Use | **Tree** | **Deprecated** | **Index** | **Help** | | |  |
| **PREV LETTER**   **NEXT LETTER** | **FRAMES**    **NO FRAMES**     **All Classes** |


A B C D E F G H I J K L M N O P Q R S T U V W X Y 

---
