# Supplementary material for: EnzML: multi-label prediction of enzyme classes using InterPro signatures
Source: BMC Bioinformatics. 2012 Apr 25;13:61. doi: 10.1186/1471-2105-13-61 (PMC3483700; doi:10.1186/1471-2105-13-61)
Supplement: Addtional file 5 — The Java code to format the data files, evaluate and predict. The file enzml_java_code.tar.gz contains the Java code used to format database data to ARFF and XML formats, to execute cross and train-test (jackknife) evaluations and to record evaluation results to database. More information is included in the readme.txt file and the Javadoc files. The code can be used with a MySQL database. To use a different database software, other JDBC drivers might be required. [file 1471-2105-13-61-S5.gz › java_code/ecmulan/doc/index-files/index-6.html]

G-Index


---


|  |  |  |  |  |  |  |  |  |  |  |
| --- | --- | --- | --- | --- | --- | --- | --- | --- | --- | --- |
| |  |  |  |  |  |  |  |  | | --- | --- | --- | --- | --- | --- | --- | --- | | **Overview** | Package | Class | Use | **Tree** | **Deprecated** | **Index** | **Help** | | |  |
| **PREV LETTER**   **NEXT LETTER** | **FRAMES**    **NO FRAMES**     **All Classes** |


A C D E F G I L M S T U W 

---


## **G**

**generateEcNumber(String)** - Static method in class uk.ac.ed.inf.ec.EcNumberGenerator: **get0dashEcA()** - Static method in class uk.ac.ed.inf.ec.test.EcNumberTest: **get0dashEcB()** - Static method in class uk.ac.ed.inf.ec.test.EcNumberTest: **get1dashEc()** - Static method in class uk.ac.ed.inf.ec.test.EcNumberTest: **get2dashEc()** - Static method in class uk.ac.ed.inf.ec.test.EcNumberTest: **get3dashEc()** - Static method in class uk.ac.ed.inf.ec.test.EcNumberTest: **get4dashEc()** - Static method in class uk.ac.ed.inf.ec.test.EcNumberTest: **GET\_EC\_QUERY\_1** - Static variable in class uk.ac.ed.inf.ec.test.EcDbReaderTest: **GET\_EC\_QUERY\_2** - Static variable in class uk.ac.ed.inf.ec.test.EcDbReaderTest: **getAncestorsStrings()** - Method in class uk.ac.ed.inf.ec.EcNumber: Get a complete hierarchy of ancestor for the ec number. **getBlocks()** - Method in class uk.ac.ed.inf.ec.EcNumber: **getDbReader()** - Method in class uk.ac.ed.inf.ec.EcFullXmlCreator: **getEcDbWriter()** - Static method in class uk.ac.ed.inf.ec.test.EcDbWriterTest: **getEcList()** - Method in class uk.ac.ed.inf.ec.EcDbReader: **getEcNumbers()** - Method in class uk.ac.ed.inf.ec.EcFullXmlCreator: **getEcSqlQuery()** - Method in class uk.ac.ed.inf.ec.EcDbReader: **getEcString()** - Method in class uk.ac.ed.inf.ec.EcNumber: **getEcStringFromBlocks(String[])** - Static method in class uk.ac.ed.inf.ec.EcNumber: **getEcTable()** - Method in class uk.ac.ed.inf.ec.EcDbWriter: Creates the table for the ec numbers and ancestors **getHierarchyLevel()** - Method in class uk.ac.ed.inf.ec.EcNumber: **getMulanLabel()** - Static method in class uk.ac.ed.inf.ec.test.MulanLabelTest: **getMulanLabelXml()** - Static method in class uk.ac.ed.inf.ec.test.MulanXmlTest: **getParent()** - Method in class uk.ac.ed.inf.ec.EcNumber: **getParentString()** - Method in class uk.ac.ed.inf.ec.EcNumber: **getRoot()** - Method in class uk.ac.ed.inf.ec.MulanXml: **getSet()** - Static method in class uk.ac.ed.inf.ec.test.EcFullXmlCreatorTest: **getTableManager()** - Method in class uk.ac.ed.inf.ec.EcDbWriter: **getXmlCreator0dashA()** - Static method in class uk.ac.ed.inf.ec.test.EcFullXmlCreatorTest: **getXmlCreator1dash()** - Static method in class uk.ac.ed.inf.ec.test.EcFullXmlCreatorTest: **getXmlCreator2dash()** - Static method in class uk.ac.ed.inf.ec.test.EcFullXmlCreatorTest: **getXmlCreator3dash()** - Static method in class uk.ac.ed.inf.ec.test.EcFullXmlCreatorTest: **getXmlCreator3EcNumbers()** - Static method in class uk.ac.ed.inf.ec.test.EcFullXmlCreatorTest: **getXmlCreator4dash()** - Static method in class uk.ac.ed.inf.ec.test.EcFullXmlCreatorTest: **getXmlCreator5()** - Static method in class uk.ac.ed.inf.ec.test.EcMulanXmlCreatorTest: **getXmlCreatorWithDbConn()** - Static method in class uk.ac.ed.inf.ec.test.EcFullXmlCreatorTest: **getXmlString()** - Method in class uk.ac.ed.inf.ec.EcFullXmlCreator: Adds a log to the xml file (timestamp, database and query to the data etc.)

---


|  |  |  |  |  |  |  |  |  |  |  |
| --- | --- | --- | --- | --- | --- | --- | --- | --- | --- | --- |
| |  |  |  |  |  |  |  |  | | --- | --- | --- | --- | --- | --- | --- | --- | | **Overview** | Package | Class | Use | **Tree** | **Deprecated** | **Index** | **Help** | | |  |
| **PREV LETTER**   **NEXT LETTER** | **FRAMES**    **NO FRAMES**     **All Classes** |


A C D E F G I L M S T U W 

---
