# Supplementary material for: EnzML: multi-label prediction of enzyme classes using InterPro signatures
Source: BMC Bioinformatics. 2012 Apr 25;13:61. doi: 10.1186/1471-2105-13-61 (PMC3483700; doi:10.1186/1471-2105-13-61)
Supplement: Addtional file 5 — The Java code to format the data files, evaluate and predict. The file enzml_java_code.tar.gz contains the Java code used to format database data to ARFF and XML formats, to execute cross and train-test (jackknife) evaluations and to record evaluation results to database. More information is included in the readme.txt file and the Javadoc files. The code can be used with a MySQL database. To use a different database software, other JDBC drivers might be required. [file 1471-2105-13-61-S5.gz › java_code/ecmulan/doc/index-files/index-10.html]

S-Index


---


|  |  |  |  |  |  |  |  |  |  |  |
| --- | --- | --- | --- | --- | --- | --- | --- | --- | --- | --- |
| |  |  |  |  |  |  |  |  | | --- | --- | --- | --- | --- | --- | --- | --- | | **Overview** | Package | Class | Use | **Tree** | **Deprecated** | **Index** | **Help** | | |  |
| **PREV LETTER**   **NEXT LETTER** | **FRAMES**    **NO FRAMES**     **All Classes** |


A C D E F G I L M S T U W 

---


## **S**

**saveToFile(String)** - Method in class uk.ac.ed.inf.ec.EcFullXmlCreator: **setUp()** - Method in class uk.ac.ed.inf.ec.test.EcDbWriterTest: **setUp()** - Method in class uk.ac.ed.inf.ec.test.EcFullXmlCreatorTest: **setUp()** - Method in class uk.ac.ed.inf.ec.test.EcMulanXmlCreatorTest: **setUp()** - Method in class uk.ac.ed.inf.ec.test.EcNumberGeneratorTest: **setUp()** - Method in class uk.ac.ed.inf.ec.test.MulanLabelTest: **setUp()** - Method in class uk.ac.ed.inf.ec.test.MulanXmlTest: **suite()** - Static method in class uk.ac.ed.inf.ec.test.AllTests

---


|  |  |  |  |  |  |  |  |  |  |  |
| --- | --- | --- | --- | --- | --- | --- | --- | --- | --- | --- |
| |  |  |  |  |  |  |  |  | | --- | --- | --- | --- | --- | --- | --- | --- | | **Overview** | Package | Class | Use | **Tree** | **Deprecated** | **Index** | **Help** | | |  |
| **PREV LETTER**   **NEXT LETTER** | **FRAMES**    **NO FRAMES**     **All Classes** |


A C D E F G I L M S T U W 

---
