# Supplementary material for: EnzML: multi-label prediction of enzyme classes using InterPro signatures
Source: BMC Bioinformatics. 2012 Apr 25;13:61. doi: 10.1186/1471-2105-13-61 (PMC3483700; doi:10.1186/1471-2105-13-61)
Supplement: Addtional file 5 — The Java code to format the data files, evaluate and predict. The file enzml_java_code.tar.gz contains the Java code used to format database data to ARFF and XML formats, to execute cross and train-test (jackknife) evaluations and to record evaluation results to database. More information is included in the readme.txt file and the Javadoc files. The code can be used with a MySQL database. To use a different database software, other JDBC drivers might be required. [file 1471-2105-13-61-S5.gz › java_code/ecmulan/doc/index-files/index-4.html]

E-Index


---


|  |  |  |  |  |  |  |  |  |  |  |
| --- | --- | --- | --- | --- | --- | --- | --- | --- | --- | --- |
| |  |  |  |  |  |  |  |  | | --- | --- | --- | --- | --- | --- | --- | --- | | **Overview** | Package | Class | Use | **Tree** | **Deprecated** | **Index** | **Help** | | |  |
| **PREV LETTER**   **NEXT LETTER** | **FRAMES**    **NO FRAMES**     **All Classes** |


A C D E F G I L M S T U W 

---


## **E**

**EC\_DATA\_TYPE** - Static variable in class uk.ac.ed.inf.ec.EcDbWriter: the sql data type for ec numbers **EC\_FIELD\_NAME** - Static variable in class uk.ac.ed.inf.ec.EcDbWriter: the name of the field to contain the ec number **EC\_TABLE\_NAME** - Static variable in class uk.ac.ed.inf.ec.EcDbWriter: the name of the table for ec and ancestors **EcDbReader** - Class in uk.ac.ed.inf.ec: Reads the full list of Enzyme Commission numbers from database **EcDbReader(DbManager, String)** - Constructor for class uk.ac.ed.inf.ec.EcDbReader: **EcDbReaderTest** - Class in uk.ac.ed.inf.ec.test: Class **EcDbReaderTest()** - Constructor for class uk.ac.ed.inf.ec.test.EcDbReaderTest: **EcDbWriter** - Class in uk.ac.ed.inf.ec: Given a database and a list of Enzyme commission numbers, writes a 2 columns table containing: in column 1: the Ec number, in column 2: all the ancestors of that EC number, including itself. **EcDbWriter(DbManager, String)** - Constructor for class uk.ac.ed.inf.ec.EcDbWriter: Reads ec numbers from database and writes them and their ancestors to a new table **EcDbWriterTest** - Class in uk.ac.ed.inf.ec.test: Class **EcDbWriterTest()** - Constructor for class uk.ac.ed.inf.ec.test.EcDbWriterTest: **EcFullXmlCreator** - Class in uk.ac.ed.inf.ec: Creates a full XML hierarchical representation of Enzyme Commission numbers in Mulan format. **EcFullXmlCreator(String, String)** - Constructor for class uk.ac.ed.inf.ec.EcFullXmlCreator: Get EC numbers from database **EcFullXmlCreator(TreeSet<String>)** - Constructor for class uk.ac.ed.inf.ec.EcFullXmlCreator: Get EC numbers from list **EcFullXmlCreatorTest** - Class in uk.ac.ed.inf.ec.test: Class **EcFullXmlCreatorTest()** - Constructor for class uk.ac.ed.inf.ec.test.EcFullXmlCreatorTest: **EcMulanXmlCreator** - Class in uk.ac.ed.inf.ec: Creates a full XML hierarchical representation of Enzyme Commission numbers in Mulan format. **EcMulanXmlCreator(String, String)** - Constructor for class uk.ac.ed.inf.ec.EcMulanXmlCreator: **EcMulanXmlCreator(TreeSet<String>)** - Constructor for class uk.ac.ed.inf.ec.EcMulanXmlCreator: **EcMulanXmlCreatorTest** - Class in uk.ac.ed.inf.ec.test: Class **EcMulanXmlCreatorTest()** - Constructor for class uk.ac.ed.inf.ec.test.EcMulanXmlCreatorTest: **EcNumber** - Class in uk.ac.ed.inf.ec: A class representing an Enzyme Commission (EC) number. **EcNumber(String)** - Constructor for class uk.ac.ed.inf.ec.EcNumber: **EcNumberGenerator** - Class in uk.ac.ed.inf.ec: Given a string such as 1.2.3.4 or 1.2.-.-.- checks if it is a valid Enzyme commission number and generates the Java EcNumber object (including its parent EC numbers) **EcNumberGenerator(String)** - Constructor for class uk.ac.ed.inf.ec.EcNumberGenerator: **EcNumberGeneratorTest** - Class in uk.ac.ed.inf.ec.test: Class **EcNumberGeneratorTest()** - Constructor for class uk.ac.ed.inf.ec.test.EcNumberGeneratorTest: **EcNumberTest** - Class in uk.ac.ed.inf.ec.test: Class **EcNumberTest()** - Constructor for class uk.ac.ed.inf.ec.test.EcNumberTest

---


|  |  |  |  |  |  |  |  |  |  |  |
| --- | --- | --- | --- | --- | --- | --- | --- | --- | --- | --- |
| |  |  |  |  |  |  |  |  | | --- | --- | --- | --- | --- | --- | --- | --- | | **Overview** | Package | Class | Use | **Tree** | **Deprecated** | **Index** | **Help** | | |  |
| **PREV LETTER**   **NEXT LETTER** | **FRAMES**    **NO FRAMES**     **All Classes** |


A C D E F G I L M S T U W 

---
