# Supplementary material for: EnzML: multi-label prediction of enzyme classes using InterPro signatures
Source: BMC Bioinformatics. 2012 Apr 25;13:61. doi: 10.1186/1471-2105-13-61 (PMC3483700; doi:10.1186/1471-2105-13-61)
Supplement: Addtional file 5 — The Java code to format the data files, evaluate and predict. The file enzml_java_code.tar.gz contains the Java code used to format database data to ARFF and XML formats, to execute cross and train-test (jackknife) evaluations and to record evaluation results to database. More information is included in the readme.txt file and the Javadoc files. The code can be used with a MySQL database. To use a different database software, other JDBC drivers might be required. [file 1471-2105-13-61-S5.gz › java_code/ecmulan/doc/index-files/index-9.html]

M-Index


---


|  |  |  |  |  |  |  |  |  |  |  |
| --- | --- | --- | --- | --- | --- | --- | --- | --- | --- | --- |
| |  |  |  |  |  |  |  |  | | --- | --- | --- | --- | --- | --- | --- | --- | | **Overview** | Package | Class | Use | **Tree** | **Deprecated** | **Index** | **Help** | | |  |
| **PREV LETTER**   **NEXT LETTER** | **FRAMES**    **NO FRAMES**     **All Classes** |


A C D E F G I L M S T U W 

---


## **M**

**m\_ecSqlQuery** - Variable in class uk.ac.ed.inf.ec.EcDbReader: **m\_root** - Variable in class uk.ac.ed.inf.ec.MulanXml: the xml tree root **main(String[])** - Static method in class uk.ac.ed.inf.ec.EcDbWriter: Main for recreating table **main(String[])** - Static method in class uk.ac.ed.inf.ec.EcFullXmlCreator: **main(String[])** - Static method in class uk.ac.ed.inf.ec.EcMulanXmlCreator: **main(String[])** - Static method in class uk.ac.ed.inf.ec.test.AllTests: **MAX\_LEVEL1\_CLASS** - Static variable in class uk.ac.ed.inf.ec.EcNumberGenerator: maximum value for level 1 class: currently 6. **MAX\_LEVEL1\_REGEXP** - Static variable in class uk.ac.ed.inf.ec.EcNumberGenerator: regexp from 1 to 6 **MAX\_LEVEL2\_CLASS** - Static variable in class uk.ac.ed.inf.ec.EcNumberGenerator: maximum value for level 2 class: 99 in this implementation. **MAX\_LEVEL2\_REGEXP** - Static variable in class uk.ac.ed.inf.ec.EcNumberGenerator: regexp from 0 to 99 **MAX\_LEVEL3\_CLASS** - Static variable in class uk.ac.ed.inf.ec.EcNumberGenerator: maximum value for level 3 class: 99 in this implementation. **MAX\_LEVEL3\_REGEXP** - Static variable in class uk.ac.ed.inf.ec.EcNumberGenerator: regexp from 0 to 99 **MAX\_LEVEL4\_CLASS** - Static variable in class uk.ac.ed.inf.ec.EcNumberGenerator: maximum value for level 4 class: 999 in this implementation. **MAX\_LEVEL4\_REGEXP** - Static variable in class uk.ac.ed.inf.ec.EcNumberGenerator: regexp from 0 to 999 **MULAN\_XML\_ROOT\_TAG** - Static variable in class uk.ac.ed.inf.ec.MulanXml: **MULAN\_XML\_ROOT\_TAG\_ATTRIBUTE\_NAME** - Static variable in class uk.ac.ed.inf.ec.MulanXml: **MULAN\_XML\_ROOT\_TAG\_ATTRIBUTE\_VALUE** - Static variable in class uk.ac.ed.inf.ec.MulanXml: **MulanLabel** - Class in uk.ac.ed.inf.ec: A node in the Mulan XML (a label for machine learning) \* **MulanLabel(String)** - Constructor for class uk.ac.ed.inf.ec.MulanLabel: **MulanLabelTest** - Class in uk.ac.ed.inf.ec.test: Class **MulanLabelTest()** - Constructor for class uk.ac.ed.inf.ec.test.MulanLabelTest: **MulanXml** - Class in uk.ac.ed.inf.ec: Generates an XML file for labels in the Mulan format http://mulan.sourceforge.net/ http://mlkd.csd.auth.gr/multilabel.html **MulanXml()** - Constructor for class uk.ac.ed.inf.ec.MulanXml: **MulanXmlTest** - Class in uk.ac.ed.inf.ec.test: Test Generates an XML file for labels in the Mulan format http://mulan.sourceforge.net/ http://mlkd.csd.auth.gr/multilabel.html **MulanXmlTest()** - Constructor for class uk.ac.ed.inf.ec.test.MulanXmlTest

---


|  |  |  |  |  |  |  |  |  |  |  |
| --- | --- | --- | --- | --- | --- | --- | --- | --- | --- | --- |
| |  |  |  |  |  |  |  |  | | --- | --- | --- | --- | --- | --- | --- | --- | | **Overview** | Package | Class | Use | **Tree** | **Deprecated** | **Index** | **Help** | | |  |
| **PREV LETTER**   **NEXT LETTER** | **FRAMES**    **NO FRAMES**     **All Classes** |


A C D E F G I L M S T U W 

---
