# Supplementary material for: EnzML: multi-label prediction of enzyme classes using InterPro signatures
Source: BMC Bioinformatics. 2012 Apr 25;13:61. doi: 10.1186/1471-2105-13-61 (PMC3483700; doi:10.1186/1471-2105-13-61)
Supplement: Addtional file 5 — The Java code to format the data files, evaluate and predict. The file enzml_java_code.tar.gz contains the Java code used to format database data to ARFF and XML formats, to execute cross and train-test (jackknife) evaluations and to record evaluation results to database. More information is included in the readme.txt file and the Javadoc files. The code can be used with a MySQL database. To use a different database software, other JDBC drivers might be required. [file 1471-2105-13-61-S5.gz › java_code/enzml2011/doc/test/dataharness/package-tree.html]

test.dataharness Class Hierarchy


---


|  |  |  |  |  |  |  |  |  |  |  |
| --- | --- | --- | --- | --- | --- | --- | --- | --- | --- | --- |
| |  |  |  |  |  |  |  |  | | --- | --- | --- | --- | --- | --- | --- | --- | | **Overview** | **Package** | Class | Use | **Tree** | **Deprecated** | **Index** | **Help** | | |  |
| **PREV**   **NEXT** | **FRAMES**    **NO FRAMES**     **All Classes** |


---


## Hierarchy For Package test.dataharness

**Package Hierarchies:**: All Packages

---

## Class Hierarchy

- java.lang.Object
  - test.dataharness.**AllDataTests**- junit.framework.Assert
      - junit.framework.TestCase (implements junit.framework.Test)
        - test.dataharness.**ArffPropsFilesTest**- test.dataharness.**ArffPropsOneTest**- test.dataharness.**ArffPropsQueriesOneTest**- test.dataharness.**ArffPropsQueriesTest**- test.dataharness.**ArffPropsQueriesTwoTest**- test.dataharness.**ArffPropsTwoTest**- test.dataharness.**CreateDataTable**
                      - test.dataharness.**DataTableOneTest**- test.dataharness.**DataTableThreeTest**- test.dataharness.**DataTableTwoTest**- test.dataharness.**DatabaseTest**- test.dataharness.**TestProjectParametersTest**- test.dataharness.**DataOne**- test.dataharness.**DataTwo**- uk.ac.ed.inf.enzml.**ProjectParameters**
            - test.dataharness.**TestProjectParameters**

---


|  |  |  |  |  |  |  |  |  |  |  |
| --- | --- | --- | --- | --- | --- | --- | --- | --- | --- | --- |
| |  |  |  |  |  |  |  |  | | --- | --- | --- | --- | --- | --- | --- | --- | | **Overview** | **Package** | Class | Use | **Tree** | **Deprecated** | **Index** | **Help** | | |  |
| **PREV**   **NEXT** | **FRAMES**    **NO FRAMES**     **All Classes** |


---
