# Supplementary material for: EnzML: multi-label prediction of enzyme classes using InterPro signatures
Source: BMC Bioinformatics. 2012 Apr 25;13:61. doi: 10.1186/1471-2105-13-61 (PMC3483700; doi:10.1186/1471-2105-13-61)
Supplement: Addtional file 5 — The Java code to format the data files, evaluate and predict. The file enzml_java_code.tar.gz contains the Java code used to format database data to ARFF and XML formats, to execute cross and train-test (jackknife) evaluations and to record evaluation results to database. More information is included in the readme.txt file and the Javadoc files. The code can be used with a MySQL database. To use a different database software, other JDBC drivers might be required. [file 1471-2105-13-61-S5.gz › java_code/enzml2011/doc/test/dataharness/package-summary.html]

test.dataharness


---


|  |  |  |  |  |  |  |  |  |  |  |
| --- | --- | --- | --- | --- | --- | --- | --- | --- | --- | --- |
| |  |  |  |  |  |  |  |  | | --- | --- | --- | --- | --- | --- | --- | --- | | **Overview** | **Package** | Class | **Use** | **Tree** | **Deprecated** | **Index** | **Help** | | |  |
| **PREV PACKAGE**   **NEXT PACKAGE** | **FRAMES**    **NO FRAMES**     **All Classes** |


---

## Package test.dataharness

| **Class Summary** | |
| --- | --- |
| **AllDataTests** | Data harness for arff file generation and learning tests. |
| **ArffPropsFilesTest** | Test the ARFF properties files used in the test harness and their content. |
| **ArffPropsOneTest** | Get the arff data properties from file |
| **ArffPropsQueriesOneTest** | Class |
| **ArffPropsQueriesTest** | Checks the results of the queries contained in the arff properties files |
| **ArffPropsQueriesTwoTest** | Class |
| **ArffPropsTwoTest** | Class |
| **CreateDataTable** | Create a table for test instances (and their attributes and classes) |
| **DatabaseTest** | Check the test database |
| **DataOne** | Class |
| **DataTableOneTest** | Basic data table containing 3 columns with: instances, attributes, classes. |
| **DataTableThreeTest** |  |
| **DataTableTwoTest** |  |
| **DataTwo** | Class |
| **TestProjectParameters** |  |
| **TestProjectParametersTest** | Class |

---


|  |  |  |  |  |  |  |  |  |  |  |
| --- | --- | --- | --- | --- | --- | --- | --- | --- | --- | --- |
| |  |  |  |  |  |  |  |  | | --- | --- | --- | --- | --- | --- | --- | --- | | **Overview** | **Package** | Class | **Use** | **Tree** | **Deprecated** | **Index** | **Help** | | |  |
| **PREV PACKAGE**   **NEXT PACKAGE** | **FRAMES**    **NO FRAMES**     **All Classes** |


---
