# Supplementary material for: EnzML: multi-label prediction of enzyme classes using InterPro signatures
Source: BMC Bioinformatics. 2012 Apr 25;13:61. doi: 10.1186/1471-2105-13-61 (PMC3483700; doi:10.1186/1471-2105-13-61)
Supplement: Addtional file 5 — The Java code to format the data files, evaluate and predict. The file enzml_java_code.tar.gz contains the Java code used to format database data to ARFF and XML formats, to execute cross and train-test (jackknife) evaluations and to record evaluation results to database. More information is included in the readme.txt file and the Javadoc files. The code can be used with a MySQL database. To use a different database software, other JDBC drivers might be required. [file 1471-2105-13-61-S5.gz › java_code/enzml2011/doc/test/dataharness/TestProjectParameters.html]

TestProjectParameters


---


|  |  |  |  |  |  |  |  |  |  |  |
| --- | --- | --- | --- | --- | --- | --- | --- | --- | --- | --- |
| |  |  |  |  |  |  |  |  | | --- | --- | --- | --- | --- | --- | --- | --- | | **Overview** | **Package** | **Class** | **Use** | **Tree** | **Deprecated** | **Index** | **Help** | | |  |
| **PREV CLASS**   **NEXT CLASS** | **FRAMES**    **NO FRAMES**     **All Classes** |
| SUMMARY: NESTED | FIELD | CONSTR | METHOD | DETAIL: FIELD | CONSTR | METHOD |


---


## test.dataharness Class TestProjectParameters

```
java.lang.Object
  uk.ac.ed.inf.enzml.ProjectParameters
      test.dataharness.TestProjectParameters
```

---

``` public class TestProjectParameters extends ProjectParameters ```

---

| **Field Summary** | |
| --- | --- |
| `static java.lang.String` | `FILTER_OLD_TEST_SET` |
| `static java.lang.String` | `FILTER_TRAIN_SET` |
| `static java.lang.String` | `INSTANCE_1_2` |
| `static java.lang.String` | `INSTANCE_1_2_EMPTY` |
| `static java.lang.String` | `INSTANCE_3` |
| `static java.lang.String` | `TEST_ARFF_DB_PROPS` |
| `static java.lang.String` | `TEST_ARFF_FILES_PATH` |
| `static java.lang.String` | `TEST_ARFF_PROPS_1` |
| `static java.lang.String` | `TEST_ARFF_PROPS_2` |
| `static java.lang.String` | `TEST_ARFF_PROPS_NO_XML` |
| `static java.lang.String` | `TEST_ARFF_PROPS_PATH` |
| `static java.lang.String` | `TEST_BASE_DATA_PATH` |
| `static java.lang.String` | `TEST_BASE_PATH` |
| `static java.lang.String` | `TEST_DATA_DIR` |
| `static java.lang.String` | `TEST_DB_CONN_PROPERTIES` |
| `static java.lang.String` | `TEST_MACHINE_LEARNING_PROPS` |
| `static java.lang.String` | `TEST_ML_PROPS_PATH` |
| `static java.lang.String` | `TEST_MODEL_FOLDER` |
| `static java.lang.String` | `TEST_PREDICTIONS_FOLDER` |
| `static java.lang.String` | `TEST_PROPS_PATH` |
| `static java.lang.String` | `TEST_RESULTS_FOLDER` |

| **Fields inherited from class uk.ac.ed.inf.enzml.ProjectParameters** |
| --- |
| `ARFF_PROPS_ARCHAEA, ARFF_PROPS_BACTERIA, ARFF_PROPS_EUKARIA, ARFF_PROPS_FUNGI, ARFF_PROPS_HUMAN, ARFF_PROPS_INVERTEBRATES, ARFF_PROPS_NOT_HUMAN, ARFF_PROPS_PATH, ARFF_PROPS_PLANTS, ARFF_PROPS_RANDOM_100, ARFF_PROPS_RANDOM_ARCHAEA, ARFF_PROPS_RANDOM_BACTERIA, ARFF_PROPS_RANDOM_EUKARIA, ARFF_PROPS_RANDOM_FUNGI, ARFF_PROPS_RANDOM_INVERTEBRATES, ARFF_PROPS_RANDOM_PLANTS, ARFF_PROPS_RANDOM_VERTEBRATES, ARFF_PROPS_SWISSKEGG, ARFF_PROPS_TREMBL_KEGG, ARFF_PROPS_VERTEBRATES, ARFF_TABLE, AUTHOR, MACHINE_LEARNING_LOCAL_DB_PROPS, MACHINE_LEARNING_PROPS, MACHINE_LEARNING_REMOTE_DB_PROPS, MODELS_PATH, PREDICTIONS_PATH, PROJECT_NAME, RESULTS_DIRECTORY, SOFTWARE_PROJECT_NAME, VERSION, WEB_PAGE` |


| **Constructor Summary** | |
| --- | --- |
| `TestProjectParameters()` |


| **Method Summary** | |
| --- | --- |

| **Methods inherited from class java.lang.Object** |
| --- |
| `equals, getClass, hashCode, notify, notifyAll, toString, wait, wait, wait` |

| **Field Detail** |
| --- |

### TEST\_BASE\_PATH

```
public static final java.lang.String TEST_BASE_PATH
```

**See Also:**: Constant Field Values

---


### TEST\_DATA\_DIR

```
public static final java.lang.String TEST_DATA_DIR
```

**See Also:**: Constant Field Values

---


### TEST\_BASE\_DATA\_PATH

```
public static final java.lang.String TEST_BASE_DATA_PATH
```

**See Also:**: Constant Field Values

---


### TEST\_PREDICTIONS\_FOLDER

```
public static final java.lang.String TEST_PREDICTIONS_FOLDER
```

**See Also:**: Constant Field Values

---


### TEST\_PROPS\_PATH

```
public static final java.lang.String TEST_PROPS_PATH
```

**See Also:**: Constant Field Values

---


### TEST\_ARFF\_FILES\_PATH

```
public static final java.lang.String TEST_ARFF_FILES_PATH
```

**See Also:**: Constant Field Values

---


### TEST\_ARFF\_PROPS\_PATH

```
public static final java.lang.String TEST_ARFF_PROPS_PATH
```

**See Also:**: Constant Field Values

---


### TEST\_ML\_PROPS\_PATH

```
public static final java.lang.String TEST_ML_PROPS_PATH
```

**See Also:**: Constant Field Values

---


### TEST\_ARFF\_PROPS\_NO\_XML

```
public static final java.lang.String TEST_ARFF_PROPS_NO_XML
```

**See Also:**: Constant Field Values

---


### TEST\_MACHINE\_LEARNING\_PROPS

```
public static final java.lang.String TEST_MACHINE_LEARNING_PROPS
```

**See Also:**: Constant Field Values

---


### TEST\_ARFF\_PROPS\_1

```
public static final java.lang.String TEST_ARFF_PROPS_1
```

**See Also:**: Constant Field Values

---


### TEST\_ARFF\_PROPS\_2

```
public static final java.lang.String TEST_ARFF_PROPS_2
```

**See Also:**: Constant Field Values

---


### TEST\_ARFF\_DB\_PROPS

```
public static final java.lang.String TEST_ARFF_DB_PROPS
```

**See Also:**: Constant Field Values

---


### TEST\_DB\_CONN\_PROPERTIES

```
public static final java.lang.String TEST_DB_CONN_PROPERTIES
```

**See Also:**: Constant Field Values

---


### TEST\_RESULTS\_FOLDER

```
public static final java.lang.String TEST_RESULTS_FOLDER
```

**See Also:**: Constant Field Values

---


### TEST\_MODEL\_FOLDER

```
public static final java.lang.String TEST_MODEL_FOLDER
```

**See Also:**: Constant Field Values

---


### FILTER\_OLD\_TEST\_SET

```
public static final java.lang.String FILTER_OLD_TEST_SET
```

**See Also:**: Constant Field Values

---


### FILTER\_TRAIN\_SET

```
public static final java.lang.String FILTER_TRAIN_SET
```

**See Also:**: Constant Field Values

---


### INSTANCE\_1\_2

```
public static final java.lang.String INSTANCE_1_2
```

**See Also:**: Constant Field Values

---


### INSTANCE\_1\_2\_EMPTY

```
public static final java.lang.String INSTANCE_1_2_EMPTY
```

**See Also:**: Constant Field Values

---


### INSTANCE\_3

```
public static final java.lang.String INSTANCE_3
```

**See Also:**: Constant Field Values


| **Constructor Detail** |
| --- |

### TestProjectParameters

```
public TestProjectParameters()
```


---


|  |  |  |  |  |  |  |  |  |  |  |
| --- | --- | --- | --- | --- | --- | --- | --- | --- | --- | --- |
| |  |  |  |  |  |  |  |  | | --- | --- | --- | --- | --- | --- | --- | --- | | **Overview** | **Package** | **Class** | **Use** | **Tree** | **Deprecated** | **Index** | **Help** | | |  |
| **PREV CLASS**   **NEXT CLASS** | **FRAMES**    **NO FRAMES**     **All Classes** |
| SUMMARY: NESTED | FIELD | CONSTR | METHOD | DETAIL: FIELD | CONSTR | METHOD |


---
