# Supplementary material for: EnzML: multi-label prediction of enzyme classes using InterPro signatures
Source: BMC Bioinformatics. 2012 Apr 25;13:61. doi: 10.1186/1471-2105-13-61 (PMC3483700; doi:10.1186/1471-2105-13-61)
Supplement: Addtional file 5 — The Java code to format the data files, evaluate and predict. The file enzml_java_code.tar.gz contains the Java code used to format database data to ARFF and XML formats, to execute cross and train-test (jackknife) evaluations and to record evaluation results to database. More information is included in the readme.txt file and the Javadoc files. The code can be used with a MySQL database. To use a different database software, other JDBC drivers might be required. [file 1471-2105-13-61-S5.gz › java_code/enzml2011/doc/test/dataharness/DataTwo.html]

DataTwo


---


|  |  |  |  |  |  |  |  |  |  |  |
| --- | --- | --- | --- | --- | --- | --- | --- | --- | --- | --- |
| |  |  |  |  |  |  |  |  | | --- | --- | --- | --- | --- | --- | --- | --- | | **Overview** | **Package** | **Class** | **Use** | **Tree** | **Deprecated** | **Index** | **Help** | | |  |
| **PREV CLASS**   **NEXT CLASS** | **FRAMES**    **NO FRAMES**     **All Classes** |
| SUMMARY: NESTED | FIELD | CONSTR | METHOD | DETAIL: FIELD | CONSTR | METHOD |


---


## test.dataharness Class DataTwo

```
java.lang.Object
  test.dataharness.DataTwo
```

---

``` public class DataTwo extends java.lang.Object ```

Class

**Version:**
:   25 Feb 2011

**Author:**
:   Luna De Ferrari luna.deferrari-at-ed.ac.uk

---

| **Field Summary** | |
| --- | --- |
| `static java.lang.String` | `arffEmptyInstance` |
| `static java.lang.String` | `arffInstance1` |
| `static java.lang.String` | `arffInstance1b` |
| `static java.lang.String` | `arffInstance2` |
| `static java.lang.String` | `arffInstance4` |
| `static java.lang.String` | `arffInstanceAttVoid` |
| `static java.lang.String` | `arffInstanceClassVoid` |
| `static java.lang.String` | `DATASET_NAME` |
| `static java.lang.String` | `INST1` |
| `static java.lang.String` | `INST2` |
| `static java.lang.String` | `INST3` |
| `static java.lang.String` | `INST4` |
| `static int` | `NUMBER_OF_INSTANCES_IN_TEST_ARFF` |


| **Constructor Summary** | |
| --- | --- |
| `DataTwo()` |


| **Method Summary** | |
| --- | --- |
| `static uk.ac.ed.inf.utils.maputils.OneToManyMap<java.lang.String,java.lang.String>` | `getInstanceAttributeMap()` |
| `static uk.ac.ed.inf.utils.maputils.OneToManyMap<java.lang.String,java.lang.String>` | `getInstanceClassMap()` |

| **Methods inherited from class java.lang.Object** |
| --- |
| `equals, getClass, hashCode, notify, notifyAll, toString, wait, wait, wait` |

| **Field Detail** |
| --- |

### NUMBER\_OF\_INSTANCES\_IN\_TEST\_ARFF

```
public static int NUMBER_OF_INSTANCES_IN_TEST_ARFF
```

---


### arffInstance1

```
public static final java.lang.String arffInstance1
```

**See Also:**: Constant Field Values

---


### arffInstance1b

```
public static final java.lang.String arffInstance1b
```

**See Also:**: Constant Field Values

---


### arffInstance2

```
public static final java.lang.String arffInstance2
```

**See Also:**: Constant Field Values

---


### arffInstance4

```
public static final java.lang.String arffInstance4
```

**See Also:**: Constant Field Values

---


### arffEmptyInstance

```
public static final java.lang.String arffEmptyInstance
```

**See Also:**: Constant Field Values

---


### arffInstanceClassVoid

```
public static final java.lang.String arffInstanceClassVoid
```

**See Also:**: Constant Field Values

---


### arffInstanceAttVoid

```
public static final java.lang.String arffInstanceAttVoid
```

**See Also:**: Constant Field Values

---


### INST1

```
public static final java.lang.String INST1
```

**See Also:**: Constant Field Values

---


### INST2

```
public static final java.lang.String INST2
```

**See Also:**: Constant Field Values

---


### INST3

```
public static final java.lang.String INST3
```

**See Also:**: Constant Field Values

---


### INST4

```
public static final java.lang.String INST4
```

**See Also:**: Constant Field Values

---


### DATASET\_NAME

```
public static final java.lang.String DATASET_NAME
```

**See Also:**: Constant Field Values


| **Constructor Detail** |
| --- |

### DataTwo

```
public DataTwo()
```


| **Method Detail** |
| --- |

### getInstanceAttributeMap

```
public static uk.ac.ed.inf.utils.maputils.OneToManyMap<java.lang.String,java.lang.String> getInstanceAttributeMap()
```

---


### getInstanceClassMap

```
public static uk.ac.ed.inf.utils.maputils.OneToManyMap<java.lang.String,java.lang.String> getInstanceClassMap()
```


---


|  |  |  |  |  |  |  |  |  |  |  |
| --- | --- | --- | --- | --- | --- | --- | --- | --- | --- | --- |
| |  |  |  |  |  |  |  |  | | --- | --- | --- | --- | --- | --- | --- | --- | | **Overview** | **Package** | **Class** | **Use** | **Tree** | **Deprecated** | **Index** | **Help** | | |  |
| **PREV CLASS**   **NEXT CLASS** | **FRAMES**    **NO FRAMES**     **All Classes** |
| SUMMARY: NESTED | FIELD | CONSTR | METHOD | DETAIL: FIELD | CONSTR | METHOD |


---
