# Supplementary material for: EnzML: multi-label prediction of enzyme classes using InterPro signatures
Source: BMC Bioinformatics. 2012 Apr 25;13:61. doi: 10.1186/1471-2105-13-61 (PMC3483700; doi:10.1186/1471-2105-13-61)
Supplement: Addtional file 5 — The Java code to format the data files, evaluate and predict. The file enzml_java_code.tar.gz contains the Java code used to format database data to ARFF and XML formats, to execute cross and train-test (jackknife) evaluations and to record evaluation results to database. More information is included in the readme.txt file and the Javadoc files. The code can be used with a MySQL database. To use a different database software, other JDBC drivers might be required. [file 1471-2105-13-61-S5.gz › java_code/enzml2011/doc/test/dataharness/DataTableTwoTest.html]

DataTableTwoTest


---


|  |  |  |  |  |  |  |  |  |  |  |
| --- | --- | --- | --- | --- | --- | --- | --- | --- | --- | --- |
| |  |  |  |  |  |  |  |  | | --- | --- | --- | --- | --- | --- | --- | --- | | **Overview** | **Package** | **Class** | **Use** | **Tree** | **Deprecated** | **Index** | **Help** | | |  |
| **PREV CLASS**   **NEXT CLASS** | **FRAMES**    **NO FRAMES**     **All Classes** |
| SUMMARY: NESTED | FIELD | CONSTR | METHOD | DETAIL: FIELD | CONSTR | METHOD |


---


## test.dataharness Class DataTableTwoTest

```
java.lang.Object
  junit.framework.Assert
      junit.framework.TestCase
          test.dataharness.CreateDataTable
              test.dataharness.DataTableTwoTest
```

**All Implemented Interfaces:**: junit.framework.Test

---

``` public class DataTableTwoTest extends CreateDataTable ```

---

| **Field Summary** | |
| --- | --- |
| `static java.lang.String` | `TABLE_TWO_NAME` |

| **Fields inherited from class test.dataharness.CreateDataTable** |
| --- |
| `ATTRIBUTE_FIELD, CLASS_FIELD, INSTANCE_FIELD` |


| **Constructor Summary** | |
| --- | --- |
| `DataTableTwoTest()` |


| **Method Summary** | |
| --- | --- |
| `java.lang.String` | `getInsertDataSqlStatement()` |
| `void` | `setTableName()` |
| `void` | `testTableContent()` |

| **Methods inherited from class test.dataharness.CreateDataTable** |
| --- |
| `createTestTable, getTableName, getTableReader, initialise, setTableName, testTableInstanceAttribute, testTableInstanceClass` |

| **Methods inherited from class junit.framework.TestCase** |
| --- |
| `countTestCases, getName, run, run, runBare, setName, toString` |

| **Methods inherited from class junit.framework.Assert** |
| --- |
| `assertEquals, assertEquals, assertEquals, assertEquals, assertEquals, assertEquals, assertEquals, assertEquals, assertEquals, assertEquals, assertEquals, assertEquals, assertEquals, assertEquals, assertEquals, assertEquals, assertEquals, assertEquals, assertEquals, assertEquals, assertFalse, assertFalse, assertNotNull, assertNotNull, assertNotSame, assertNotSame, assertNull, assertNull, assertSame, assertSame, assertTrue, assertTrue, fail, fail, failNotEquals, failNotSame, failSame, format` |

| **Methods inherited from class java.lang.Object** |
| --- |
| `equals, getClass, hashCode, notify, notifyAll, wait, wait, wait` |

| **Field Detail** |
| --- |

### TABLE\_TWO\_NAME

```
public static java.lang.String TABLE_TWO_NAME
```


| **Constructor Detail** |
| --- |

### DataTableTwoTest

```
public DataTableTwoTest()
```


| **Method Detail** |
| --- |

### getInsertDataSqlStatement

```
public java.lang.String getInsertDataSqlStatement()
```

:   **Overrides:**: `getInsertDataSqlStatement` in class `CreateDataTable`

---


### setTableName

```
public void setTableName()
```

---


### testTableContent

```
public void testTableContent()
```


---


|  |  |  |  |  |  |  |  |  |  |  |
| --- | --- | --- | --- | --- | --- | --- | --- | --- | --- | --- |
| |  |  |  |  |  |  |  |  | | --- | --- | --- | --- | --- | --- | --- | --- | | **Overview** | **Package** | **Class** | **Use** | **Tree** | **Deprecated** | **Index** | **Help** | | |  |
| **PREV CLASS**   **NEXT CLASS** | **FRAMES**    **NO FRAMES**     **All Classes** |
| SUMMARY: NESTED | FIELD | CONSTR | METHOD | DETAIL: FIELD | CONSTR | METHOD |


---
