# Supplementary material for: EnzML: multi-label prediction of enzyme classes using InterPro signatures
Source: BMC Bioinformatics. 2012 Apr 25;13:61. doi: 10.1186/1471-2105-13-61 (PMC3483700; doi:10.1186/1471-2105-13-61)
Supplement: Addtional file 5 — The Java code to format the data files, evaluate and predict. The file enzml_java_code.tar.gz contains the Java code used to format database data to ARFF and XML formats, to execute cross and train-test (jackknife) evaluations and to record evaluation results to database. More information is included in the readme.txt file and the Javadoc files. The code can be used with a MySQL database. To use a different database software, other JDBC drivers might be required. [file 1471-2105-13-61-S5.gz › java_code/enzml2011/doc/test/dataharness/DataOne.html]

DataOne


---


|  |  |  |  |  |  |  |  |  |  |  |
| --- | --- | --- | --- | --- | --- | --- | --- | --- | --- | --- |
| |  |  |  |  |  |  |  |  | | --- | --- | --- | --- | --- | --- | --- | --- | | **Overview** | **Package** | **Class** | **Use** | **Tree** | **Deprecated** | **Index** | **Help** | | |  |
| **PREV CLASS**   **NEXT CLASS** | **FRAMES**    **NO FRAMES**     **All Classes** |
| SUMMARY: NESTED | FIELD | CONSTR | METHOD | DETAIL: FIELD | CONSTR | METHOD |


---


## test.dataharness Class DataOne

```
java.lang.Object
  test.dataharness.DataOne
```

---

``` public class DataOne extends java.lang.Object ```

Class

**Version:**
:   25 Feb 2011

**Author:**
:   Luna De Ferrari luna.deferrari-at-ed.ac.uk

---

| **Field Summary** | |
| --- | --- |
| `static java.lang.String` | `arffEmptyInstance` |
| `static java.lang.String` | `arffInstance1` |
| `static java.lang.String` | `arffInstance1b` |
| `static java.lang.String` | `arffInstance2` |
| `static java.lang.String` | `arffInstance4` |
| `static java.lang.String` | `arffInstanceAttVoid` |
| `static java.lang.String` | `arffInstanceClassVoid` |
| `static java.lang.String` | `ATT1` |
| `static java.lang.String` | `ATT1_TO_STRING` |
| `static java.lang.String` | `ATT2` |
| `static java.lang.String` | `ATT2_TO_STRING` |
| `static java.lang.String` | `ATT3` |
| `static java.lang.String` | `ATT3_TO_STRING` |
| `static java.lang.String` | `ATT4` |
| `static java.lang.String` | `ATT4_TO_STRING` |
| `static java.lang.String` | `ATT5` |
| `static java.lang.String` | `ATT5_TO_STRING` |
| `static java.lang.String` | `CLASS_TO_STRING` |
| `static java.lang.String` | `CLASS1` |
| `static java.lang.String` | `CLASS2` |
| `static java.lang.String` | `CLASS3` |
| `static java.lang.String` | `CLASS4` |
| `static java.lang.String` | `DATASET_NAME` |
| `static java.lang.String` | `INST1` |
| `static java.lang.String` | `INST2` |
| `static java.lang.String` | `INST3` |
| `static java.lang.String` | `INST4` |
| `static java.lang.String` | `INSTATTVOID` |
| `static java.lang.String` | `INSTCLASSATTVOID` |
| `static java.lang.String` | `INSTCLASSVOID` |
| `static int` | `NUMBER_OF_INSTANCES_IN_TEST_ARFF` |


| **Constructor Summary** | |
| --- | --- |
| `DataOne()` |


| **Method Summary** | |
| --- | --- |
| `static java.util.Vector<java.lang.String>` | `attributesColumn()` |
| `static java.util.Vector<java.lang.String>` | `attributesValues()`             Results of a select distinct instance, attribute query |
| `static java.util.Vector<java.lang.String>` | `classColumn()` |
| `static java.util.Vector<java.lang.String>` | `classValues()`             Results of a select distinct instance, class query |
| `static java.util.Vector<java.lang.String>` | `instancesColumn()` |
| `static java.util.Vector<java.lang.String>` | `instancesValues()`             Results of a select distinct instance, class query |

| **Methods inherited from class java.lang.Object** |
| --- |
| `equals, getClass, hashCode, notify, notifyAll, toString, wait, wait, wait` |

| **Field Detail** |
| --- |

### NUMBER\_OF\_INSTANCES\_IN\_TEST\_ARFF

```
public static int NUMBER_OF_INSTANCES_IN_TEST_ARFF
```

---


### arffInstance1

```
public static final java.lang.String arffInstance1
```

**See Also:**: Constant Field Values

---


### arffInstance1b

```
public static final java.lang.String arffInstance1b
```

**See Also:**: Constant Field Values

---


### arffInstance2

```
public static final java.lang.String arffInstance2
```

**See Also:**: Constant Field Values

---


### arffInstance4

```
public static final java.lang.String arffInstance4
```

**See Also:**: Constant Field Values

---


### arffEmptyInstance

```
public static final java.lang.String arffEmptyInstance
```

**See Also:**: Constant Field Values

---


### arffInstanceClassVoid

```
public static final java.lang.String arffInstanceClassVoid
```

**See Also:**: Constant Field Values

---


### arffInstanceAttVoid

```
public static final java.lang.String arffInstanceAttVoid
```

**See Also:**: Constant Field Values

---


### ATT1

```
public static final java.lang.String ATT1
```

**See Also:**: Constant Field Values

---


### ATT1\_TO\_STRING

```
public static final java.lang.String ATT1_TO_STRING
```

**See Also:**: Constant Field Values

---


### ATT2

```
public static final java.lang.String ATT2
```

**See Also:**: Constant Field Values

---


### ATT2\_TO\_STRING

```
public static final java.lang.String ATT2_TO_STRING
```

**See Also:**: Constant Field Values

---


### ATT3

```
public static final java.lang.String ATT3
```

**See Also:**: Constant Field Values

---


### ATT3\_TO\_STRING

```
public static final java.lang.String ATT3_TO_STRING
```

**See Also:**: Constant Field Values

---


### ATT4

```
public static final java.lang.String ATT4
```

**See Also:**: Constant Field Values

---


### ATT4\_TO\_STRING

```
public static final java.lang.String ATT4_TO_STRING
```

**See Also:**: Constant Field Values

---


### ATT5

```
public static final java.lang.String ATT5
```

**See Also:**: Constant Field Values

---


### ATT5\_TO\_STRING

```
public static final java.lang.String ATT5_TO_STRING
```

**See Also:**: Constant Field Values

---


### CLASS\_TO\_STRING

```
public static final java.lang.String CLASS_TO_STRING
```

**See Also:**: Constant Field Values

---


### CLASS1

```
public static final java.lang.String CLASS1
```

**See Also:**: Constant Field Values

---


### CLASS2

```
public static final java.lang.String CLASS2
```

**See Also:**: Constant Field Values

---


### CLASS3

```
public static final java.lang.String CLASS3
```

**See Also:**: Constant Field Values

---


### CLASS4

```
public static final java.lang.String CLASS4
```

**See Also:**: Constant Field Values

---


### INST1

```
public static final java.lang.String INST1
```

**See Also:**: Constant Field Values

---


### INST2

```
public static final java.lang.String INST2
```

**See Also:**: Constant Field Values

---


### INST3

```
public static final java.lang.String INST3
```

**See Also:**: Constant Field Values

---


### INST4

```
public static final java.lang.String INST4
```

**See Also:**: Constant Field Values

---


### INSTCLASSATTVOID

```
public static final java.lang.String INSTCLASSATTVOID
```

**See Also:**: Constant Field Values

---


### INSTCLASSVOID

```
public static final java.lang.String INSTCLASSVOID
```

**See Also:**: Constant Field Values

---


### INSTATTVOID

```
public static final java.lang.String INSTATTVOID
```

**See Also:**: Constant Field Values

---


### DATASET\_NAME

```
public static final java.lang.String DATASET_NAME
```

**See Also:**: Constant Field Values


| **Constructor Detail** |
| --- |

### DataOne

```
public DataOne()
```


| **Method Detail** |
| --- |

### attributesColumn

```
public static java.util.Vector<java.lang.String> attributesColumn()
```

---


### attributesValues

```
public static java.util.Vector<java.lang.String> attributesValues()
```

:   Results of a select distinct instance, attribute query

    :   **Returns:**

---


### classColumn

```
public static java.util.Vector<java.lang.String> classColumn()
```

---


### classValues

```
public static java.util.Vector<java.lang.String> classValues()
```

:   Results of a select distinct instance, class query

    :   **Returns:**

---


### instancesColumn

```
public static java.util.Vector<java.lang.String> instancesColumn()
```

---


### instancesValues

```
public static java.util.Vector<java.lang.String> instancesValues()
```

:   Results of a select distinct instance, class query

    :   **Returns:**


---


|  |  |  |  |  |  |  |  |  |  |  |
| --- | --- | --- | --- | --- | --- | --- | --- | --- | --- | --- |
| |  |  |  |  |  |  |  |  | | --- | --- | --- | --- | --- | --- | --- | --- | | **Overview** | **Package** | **Class** | **Use** | **Tree** | **Deprecated** | **Index** | **Help** | | |  |
| **PREV CLASS**   **NEXT CLASS** | **FRAMES**    **NO FRAMES**     **All Classes** |
| SUMMARY: NESTED | FIELD | CONSTR | METHOD | DETAIL: FIELD | CONSTR | METHOD |


---
