# Supplementary material for: EnzML: multi-label prediction of enzyme classes using InterPro signatures
Source: BMC Bioinformatics. 2012 Apr 25;13:61. doi: 10.1186/1471-2105-13-61 (PMC3483700; doi:10.1186/1471-2105-13-61)
Supplement: Addtional file 9 — Prediction errors analysis. The PDF file predictions.pdf contains a brief analysis of the most common prediction errors when training on SwissProtKEGG and testing on E. coli (all strains). It also contains separate accuracy results for each main EC class. [file 1471-2105-13-61-S9.pdf]

BMC Bioinformatics

EnzML: Multi-label prediction of enzyme classes using InterPro signatures

Luna De Ferrari, Stuart Aitken , Jano van Hemert and Igor Goryanin

Analysis of prediction errors

Predictions obtained by training on the SwissProt-join-KEGG dataset (minus all E. Coli strains) and predicting the E. Coli (all strains) dataset.

The figure below represents the predictive accuracy by main EC class.
The highest accuracy is achieved for classes EC 6 and EC2, while the lowest accuracy is recorded for classes EC 1 and EC 4.

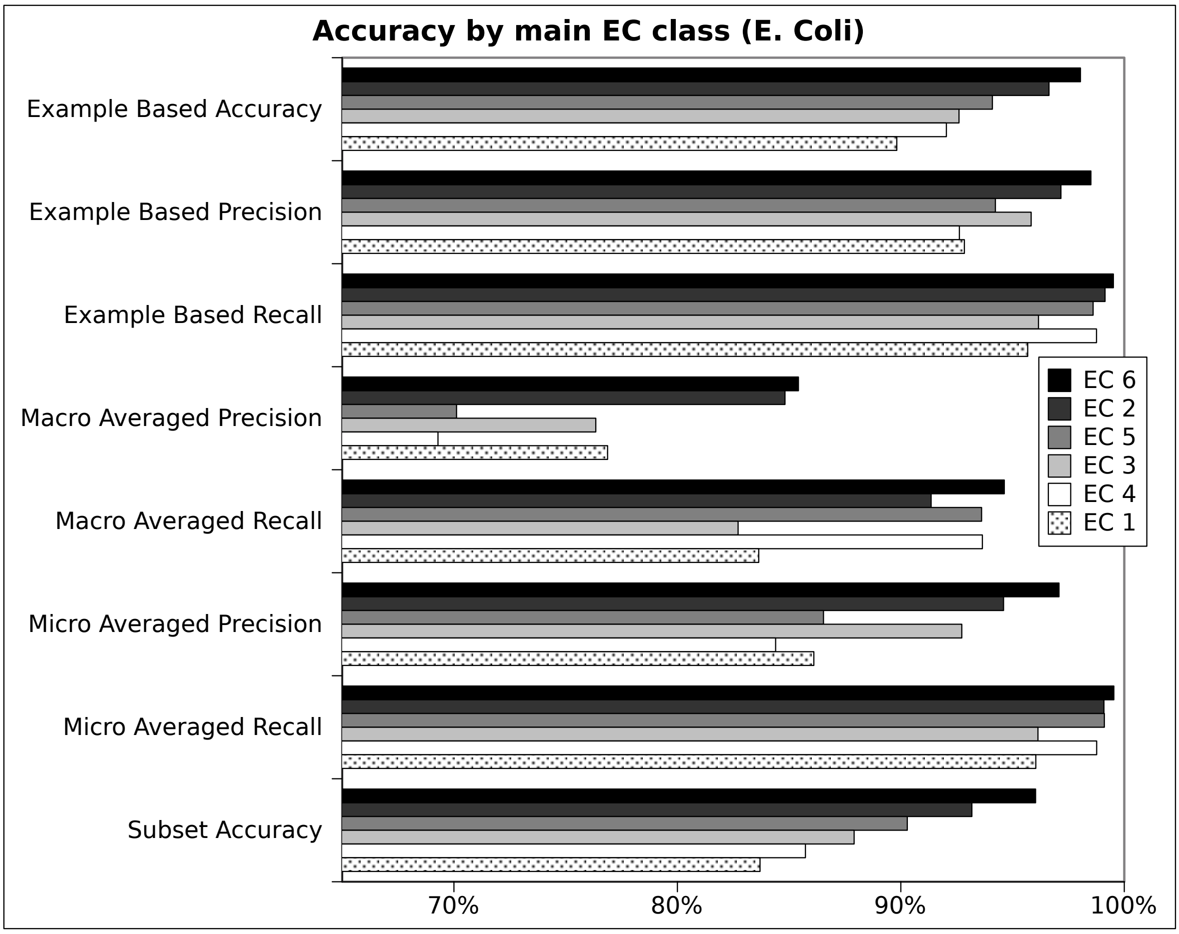

Most affected EC classes

The table above shows that errors are not homogeneously distributed among EC classes. Classes EC 5 and 6 (and no EC) are not affected by a high number of errors despite being the most frequent, while classes EC 1 to EC 4 are more affected.

The table below shows the most common kind of mistakes by main destination EC (wrong EC) and source EC (true EC). The most common mistake is the attribution of a class EC 3 to 85 proteins that are in fact non-enzymes.

Wrong EC	***True EC***	***Errors***	***% of errors***
EC 3	no EC	85	24%
EC 2	no EC	78	22%
no EC	EC 3	52	14%
EC 1	no EC	42	12%
EC 4	EC 5	16	4%
no EC	EC 1	13	4%
EC 6	EC 2	12	3%
EC 4	no EC	12	3%
EC 4	EC 2	11	3%
EC 2	EC 3	7	2%
no EC	EC 2	6	2%
EC 3	EC 5	4	1%
EC 5	no EC	4	1%
EC 4	EC 3	4	1%
EC 2	EC 4	4	1%
EC 3	EC 4	4	1%
EC 1	EC 5	3	1%
no EC	EC 6	2	0.6%

The most common error for the classifier was the classification as non-enzymes of sixteen EC 3.6.3.33 enzymes (Vitamin B12-transporting ATPase). The second most common error was to classify as EC 2.5.1.18 eleven proteins that are in fact non-enzymes in SwissProt and KEGG. But the vast majority of errors is spread across a variety of EC classes.
